# Spiroconjugated Tetraaminospirenes as Donors in Color‐Tunable Charge‐Transfer Emitters with Donor‐Acceptor Structure

**DOI:** 10.1002/chem.202104150

**Published:** 2021-12-16

**Authors:** David C. Grenz, Daniel Rose, Jan S. Wössner, Jennifer Wilbuer, Florin Adler, Mathias Hermann, Chin‐Yiu Chan, Chihaya Adachi, Birgit Esser

**Affiliations:** ^1^ Institute for Organic Chemistry University of Freiburg Albertstraße 21 79104 Freiburg Germany; ^2^ Center for Organic Photonics and Electronics Research OPERA Kyushu University 744 Motooka, Nishi 819-0395 Fukuoka Japan; ^3^ Institute for Organic Chemistry and Biochemistry University of Bonn Gerhard-Domagk-Str. 1 53121 Bonn Germany; ^4^ Freiburg Materials Research Center University of Freiburg Stefan-Meier-Str. 21 79104 Freiburg Germany; ^5^ Freiburg Center for Interactive Materials and Bioinspired Technologies University of Freiburg Georges-Köhler-Allee 105 79110 Freiburg Germany

**Keywords:** amines, charge transfer, donor-acceptor systems, fluorescence, spiro compounds

## Abstract

Charge‐transfer emitters are attractive due to their color tunability and potentially high photoluminescence quantum yields (PLQYs). We herein present tetraaminospirenes as donor moieties, which, in combination with a variety of acceptors, furnished 12 charge‐transfer emitters with a range of emission colors and PLQYs of up to 99 %. The spatial separation of their frontier molecular orbitals was obtained through careful structural design, and two DA structures were confirmed by X‐ray crystallography. A range of photophysical measurements supported by DFT calculations shed light on the optoelectronic properties of this new family of spiro‐NN‐donor‐acceptor dyes.

## Graphical abstract

**Emitters go spiro**: Using spiroconjugated tetraamines as a novel type of donor and a range of different acceptors in three different modes of connection, 12 donor‐acceptor compounds are synthesized. Their twisted structures lead to a spatial separation of the HOMOs and LUMOs. Photophysical measurements show tunable emission colors for these donor‐acceptor compounds, and photoluminescence quantum yields of up to 99 % in solution are obtained.

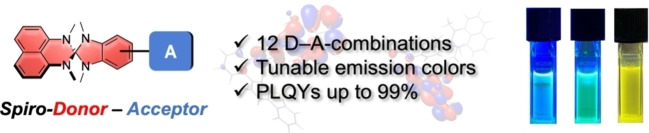

## Introduction

**Figure 1:**
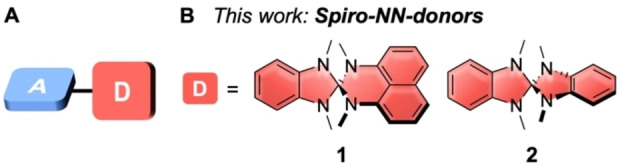
A) General structure of a small organic emitter with donor‐acceptor (DA)‐subunits; B) Structures of the spiro‐NN‐donors **1** and **2** used in this study.

**Figure 2:**
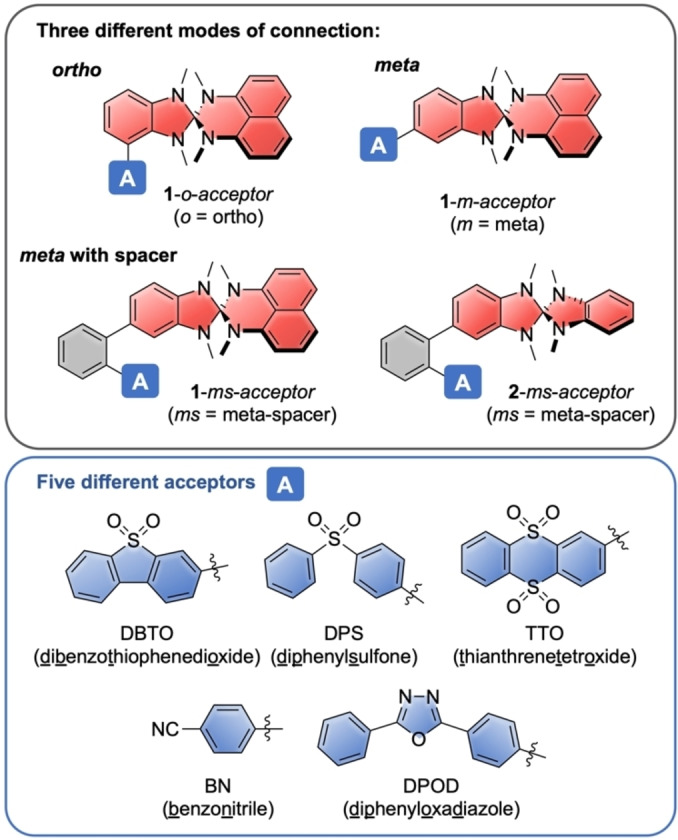
Three different modes of connection between the heterospirane donors **1** and **2** and acceptors used in this study.

Small molecules with donor‐acceptor (DA) character are attractive as active materials for optoelectronic applications. They are used in organic light‐emitting diodes (OLEDs), dye‐sensitized solar cells, as fluorescence‐based sensors, UV absorbers and nonlinear optical materials, to name a few.[^1^, ^2^, ^3^, ^4^, ^5^, ^6^, ^7^, ^8^, ^9^] In DA‐compounds, photoexcitation can lead to an intramolecular charge transfer (CT) of an electron from the donor to the acceptor part of the molecule.^[10]^ Introducing spiro units can be beneficial in organic DA‐type emitters. Due to their rigid three‐dimensional structure π‐π‐stacking and excimer formation can be effectively suppressed, since they often form non‐crystalline, amorphous structures in the solid‐state.[^11^, ^12^] In OLED emitters, such interactions can lead to significant redshifts in photoluminescence and electroluminescence, making them less practicable for commercial use.^[13]^ Another interesting feature in spiro‐compounds is the possibility of spiroconjugation,[^14^, ^15^] defined as the homoconjugative through‐space interaction between two π‐systems arranged perpendicular to each other and connected by a common tetrahedral atom.[^16^, ^17^, ^18^, ^19^] Its extent depends on the orbital overlap between the two π‐systems and hence on the substitution pattern around the spiro center.^[20]^ Several examples of spiro‐conjugated CT emitters have been reported to date, where donor and acceptor are separated by a spiro center.[^14^, ^21^, ^22^, ^23^, ^24^, ^25^, ^26^, ^27^, ^28^, ^29^] We recently reported on spiro‐donor‐σ‐acceptor dyes, with 1,3‐indandione and derivatives thereof as acceptors and aromatic methylated diamines, amino thiols, aromatic thiols, or aromatic alcohols as donor units.[^30^, ^31^] Although intramolecular CT was observed in the absorbance spectra, these dyes did not show fluorescence due to fast vibrational energy deactivation of the excited states. We herein report the first use of heterospiranes **1** and **2** as donors for charge‐transfer dyes with DA‐character (Figure 1). Heterospirane **2** was first reported in 1968 by Quast and Schmitt,^[32]^ and naphthyl‐based **1** in 1986 by Gleiter and Uschmann.^[33]^ Through photo‐electron spectroscopic investigations, Gleiter et al. attested strong spiro‐interactions in **2** due to significant orbital overlap between the two halves of the molecule.[^33^, ^34^] We combined these donors **1** and **2** with five different acceptors and used three modes of connection between donor and acceptor (Figure 2).

The type of connection between the donor and acceptor can significantly influence the optoelectronic properties of the CT‐dyes. For naphthyl‐based donor **1**, all three connection modes were employed, in which the acceptors are either connected “ortho” to the spiro unit (**1**‐*o*‐acceptor), “meta” (**1**‐*m*‐acceptor), or via a 1,2‐phenylene spacer (**1**‐*ms*‐acceptor). With donor **2**, only this third connection mode via the phenylene spacer was employed in DA compounds **2**‐*ms*‐acceptor. As acceptors, we chose dibenzothiophenedioxide (DBTO), diphenylsulfone (DPS), thianthrenetetroxide (TTO), benzonitrile (BN), and diphenyloxadiazole (DPOD) (Figure 2).

Since mono‐halogenated derivatives of heterospiranes **1** and **2** were unknown prior to this study, we developed synthetic strategies to these functional building blocks, which we employed in Suzuki‐Miyaura cross‐coupling reactions to furnish the resulting DA compounds. The structures of two DA compounds were confirmed by X‐ray crystallography. The resulting 12 in total spiro‐NN‐DA compounds showed strong photoluminescence both in solution and in thin films. We investigated in particular the influence of the connection mode between donor and acceptor on the photophysical properties and found the meta‐connection to result in the highest photoluminescence quantum yields of up to 99 %.

## Results and Discussion

### Syntheses

**Scheme 1:**
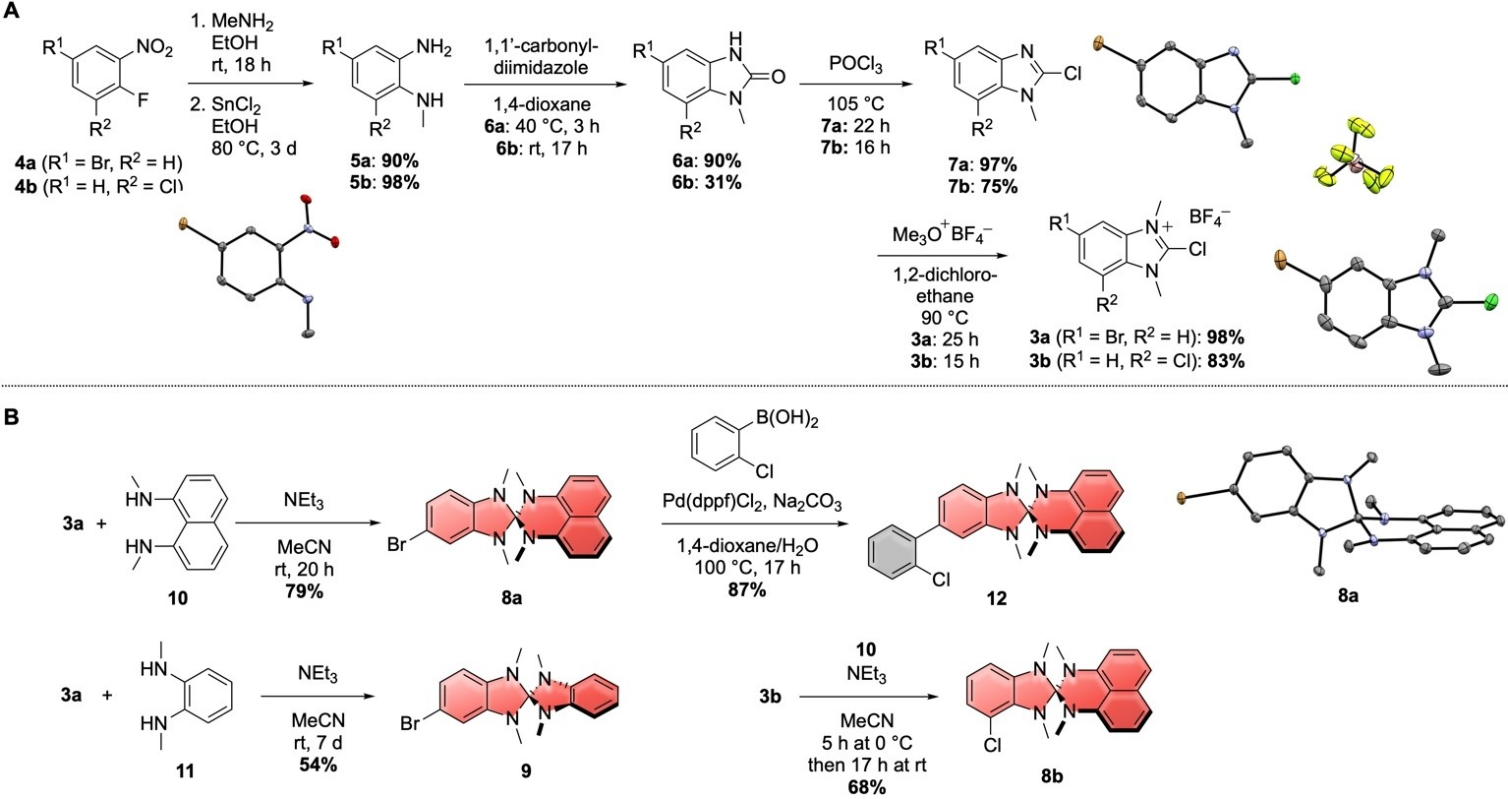
A) Synthesis of chlorobenzimidazolium salts **3** **a** and **3** **b** with molecular structures of 4‐bromo‐*N*‐methyl‐2‐nitroaniline, **7** **a** and **3** **a** in the solid state^a^; B) Synthesis of halogenated heterospiranes **8** **a**, **8** **a**, **9** and **12** with molecular structure of **8** **a** in the solid state^a^ (^a^ displacement ellipsoids are shown at the 50 % probability level; hydrogen atoms are omitted for clarity).

The synthesis of the mono‐halogenated derivatives of heterospiranes **1** and **2**, namely **8** **a** and **8** **b**, **9** and **12**, are shown in Scheme 1. The heterospirane frameworks are typically synthesized through condensation of a 2‐chlorobenzimidazolium salt and methylated *o*‐phenylenediamine or 1,8‐naphthyldiamine, thereby constructing the spiro center.[^32^, ^33^]

To obtain mono‐halogenated derivatives of these heterospiranes as functional building blocks, which can be employed in Pd‐catalyzed cross‐coupling reactions, we developed synthetic routes to the halogenated chlorobenzimidazolium salts **3** **a** and **3** **b** (Scheme 1A). Their syntheses commenced from 4‐bromo‐1‐fluoro‐2‐nitrobenzene (**4** **a**) and 1‐chloro‐2‐fluoro‐3‐nitrobenzene (**4** **b**) by nucleophilic aromatic substitution of the fluoride with methylamine, followed by reduction of the nitro‐ to an amino group with tin chloride. This furnished **5** **a** and **5** **b** in excellent yields of 90 % and 98 % over two steps, respectively. The following condensation with 1,1’‐carbonyldimidazole afforded dihydrobenzimidazolones **6** **a** and **6** **b**. Reaction with phosphoryl chloride led to imidoyl chlorides **7** **a** and **7** **b**, which were methylated with trimethyloxonium tetrafluoroborate, providing the halogenated chlorobenzimidazolium salts **3** **a** and **3** **b**. With 77 %, the overall yield for **3** **a** over five steps from **4** **a** is high, while the lower overall 19 % yield for **3** **b** from **4** **b** was mostly due to the poor yield in the condensation reaction of **5** **b** with 1,1’‐carbonyldimidazole. The molecular structures of intermediate 4‐bromo‐*N*‐methyl‐2‐nitroaniline, **7** **a**, as well as **3** **a** in the solid state were characterized by X‐ray crystallography (see structures in Scheme 1A).

For the synthesis of “meta”‐brominated heterospiranes **8** **a** and **9**, the benzimidazolium salt **3** **a** was reacted with naphthyl‐based diamine **10** and phenylene diamine **11**, respectively, using triethylamine as base in acetonitrile (Scheme 1B). For *N* ^1^,*N* ^8^‐dimethylnaphthalene‐1,8‐diamine (**10**), we developed an improved four‐step synthetic route from 1,8‐diaminonaphthalene, shown in the Supporting Information, and **11** was synthesized according to the literature.^[35]^ Due to the air and thermal sensitivity of diamines **10** and **11** this reaction had to be performed with degassed solvents at room temperature. Furthermore, the correct ratio of the solvents acetonitrile/triethylamine was critical to the yields of **8** **a** and **9**. Suzuki‐Miyaura‐coupling of **8** **a** with (2‐chlorophenyl)boronic acid allowed introducing the 1,2‐phenylene spacer unit in **12**. “Ortho”‐chlorinated **8** **b** was obtained in a similar manner by condensation of benzimidazolium salt **3** **b** with *N* ^1^,*N* ^8^‐dimethylnaphthalene‐1,8‐diamine (**10**) in a good yield of 68 %. The molecular structure of **8** **a** in the solid‐state was confirmed by X‐ray diffraction analysis (see Scheme 1B).

**Scheme 2:**
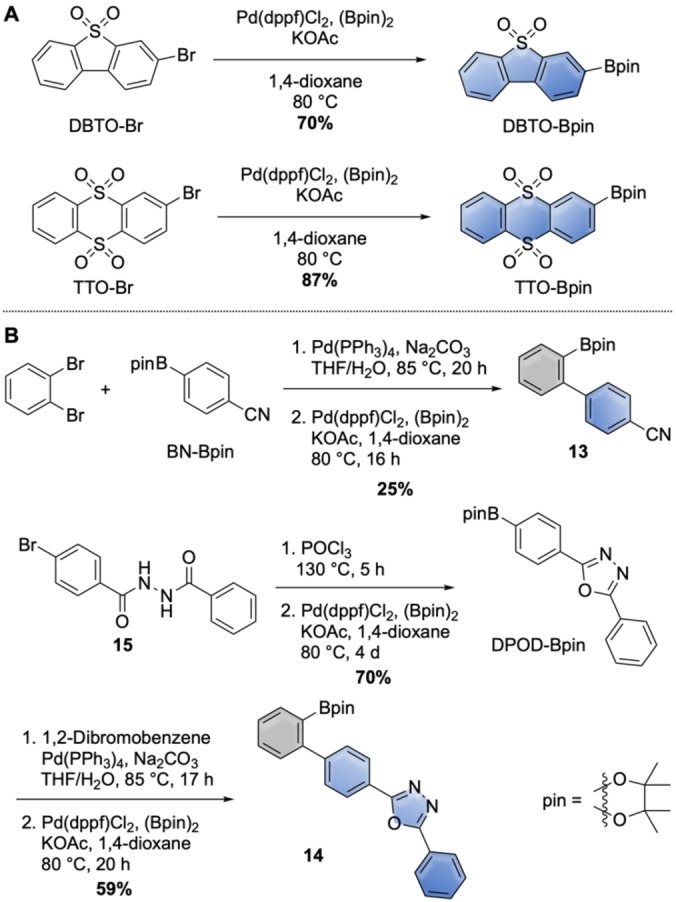
Synthesis of borylated acceptors DBTO‐Bpin, TTO‐Bpin, **13**, and **14**.

To obtain the borylated acceptors A‐Bpin with A=DBTO, DPS and TTO required for the cross‐coupling reactions with the heterospiranes, we performed Miyaura‐borylations of the respective bromides. This is shown in Scheme 2A for DBTO−Br and TTO−Br (for their synthesis, see Supporting Information)^[36]^ or has been reported in the literature for DPS‐Bpin.^[37]^ Benzonitrile‐based acceptor **13** with attached 1,2‐phenylene spacer was afforded through Suzuki‐Miyaura‐coupling of 1,2‐dibromobenzene with benzonitrile‐boronic ester BN‐Bpin followed by Miyaura‐borylation of the remaining aryl bromide (Scheme 2B). For DPOD‐based acceptor **14**, the reaction of *N*′‐benzoyl‐4‐bromobenzohydrazide (**15**) with POCl_3_ afforded the corresponding oxadiazole, which was subjected to Miyaura borylation conditions to furnish DPOD‐Bpin. Suzuki‐Miyaura‐coupling with 1,2‐dibromobenzene followed by Miyaura‐borylation of the remaining aryl bromide furnished DPOD‐based **14** with an incorporated 1,2‐phenylene spacer in good overall yield.

**Scheme 3:**
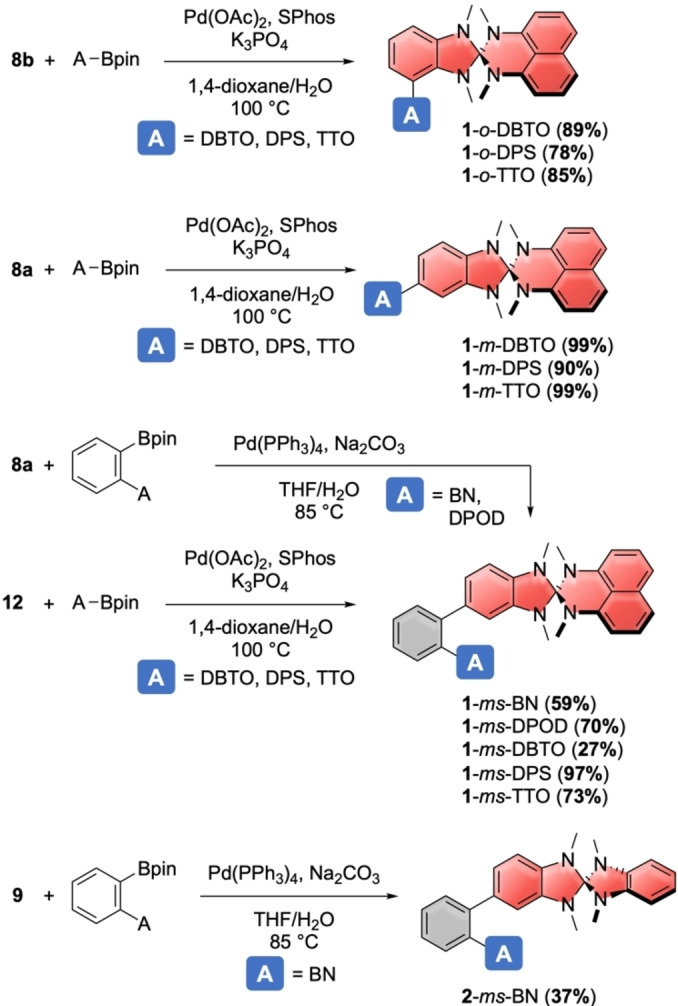
Synthesis of spiro‐NN‐DA‐compounds.

With the halogenated heterospiranes and borylated acceptors in hand, we next synthesized 12 DA‐compounds through Suzuki‐Miyaura‐cross‐coupling reactions, as shown in Scheme 3. For the “ortho”‐connection to the heterospirane donor **1**, we attached acceptors DBTO, DPS, and TTO to chloro‐heterospirane **8** **b**, furnishing **1**‐*o*‐DBTO, **1**‐*o*‐DPS, and **1**‐*o*‐TTO in good yields. The same acceptors were used in the direct “meta”‐connection to this donor by coupling to bromo‐heterospirane **8** **a**, yielding **1**‐*m*‐DBTO, **1**‐*m*‐DPS, and **1**‐*m*‐TTO in excellent yields.

To incorporate the 1,2‐phenylene spacer in the *meta*‐position of the heterospirane donors, we employed two different strategies. For acceptors BN and DPOD, the spacer was first attached to the acceptor unit BN or DPOD (see Scheme 2) and then cross‐coupled with bromo‐heterospirane **8** **a** to furnish **1**‐*ms*‐BN and **1**‐*ms*‐DPOD. The same protocol was followed using benzo‐based heterospirane **9** for the benzonitrile acceptor and afforded DA‐compound **2**‐*ms*‐BN. In the second strategy, we used the 1,2‐phenylene‐extended heterospirane **12** in Suzuki‐Miyaura‐couplings with acceptors A‐Bpin, resulting in DA‐compounds **1**‐*ms*‐DBTO, **1**‐*ms*‐DPS, and **1**‐*ms*‐TTO.

### Structural properties

**Figure 3:**
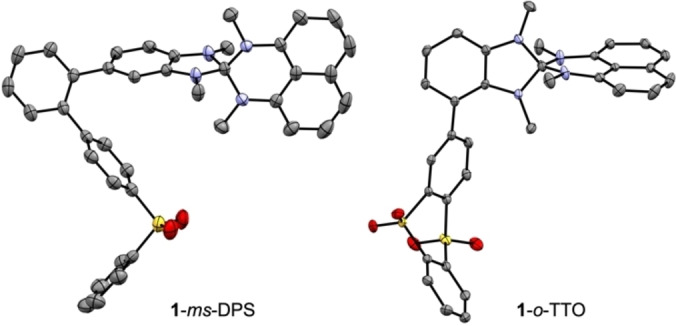
Molecular structures of **1**‐*ms*‐DPS and **1**‐*o*‐TTO in the solid‐state (displacement ellipsoids are shown at the 30 % (**1**‐*ms*‐DPS) or 50 % (**1**‐*o*‐TTO) probability level; hydrogen atoms are omitted for clarity).

The layering of dichloromethane solutions with *n*‐pentane provided single crystals suitable for X‐ray diffraction analysis of **1**‐*ms*‐DPS and **1**‐*o*‐TTO. The molecular structures are shown in Figure 3 and demonstrate a twist between donor and acceptor unit. **1**‐*ms*‐DPS possesses dihedral angles of 57.3° between the heterospirane donor and the 1,2‐phenylene spacer and of 53.8° between the latter and the diphenylsulfone acceptor. In **1**‐*o*‐TTO, the torsional angle between donor and acceptor amounts to 46.1°. Interesting are also the distances between donor and acceptor units for the intramolecular CT process. In **1**‐*o*‐TTO the distance between the S‐atom of the acceptor and the spiro‐center of the donor amounts to 8.3 Å, while this distance decreases to 6.8 Å for **1**‐*ms*‐DPS.

To obtain structural information on all spiro‐NN‐DA compounds, all 12 were optimized in the gas phase using the PBEh‐3c composite scheme.^[38]^ As for **1**‐*ms*‐DPS and **1**‐*o*‐TTO the other compounds also showed a twist between donor and acceptor (or to the *ortho*‐phenylene bridges). The torsional angles between donor and acceptor *Θ*_D‐A_ for the *m*‐ and *o*‐connection mode or between donor/acceptor and the spacer (*Θ*_D‐S_ and *Θ*_A‐S_) for the *ms*‐connection are listed in Table 1. The largest torsion angles with values of ca. 50°–60° were found for the DA compounds with a *meta*‐spacer in between donor and acceptor and for those with an *ortho*‐connection to the donor. The direct *meta*‐connection resulted in smaller values of 37°–39° due to decreased steric repulsion. Large torsion angles between donor and acceptor help in localizing the electron densities of the HOMO and LUMO states on donor and acceptor moieties, respectively, but can also diminish the oscillator strengths of the charge‐transfer absorptions.

**Table 1:** Torsional angles between donor and 1,2‐phenylene spacer *Θ*_D−S_, acceptor and 1,2‐phenylene spacer *Θ*_A−S_, or donor and acceptor *Θ*_DA_ in spiro‐NN‐DA compounds (from gas phase structures calculated at the PBEh‐3c level of theory).

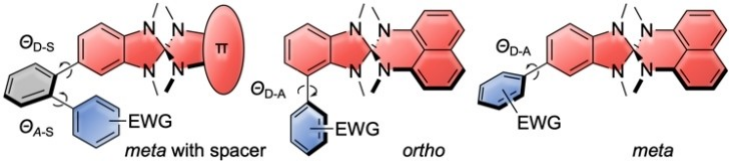				
Compound	*Θ*_D‐S_	*Θ*_A‐S_	Compound	*Θ*_D‐A_
**1**‐*ms*‐BN	54.3°	51.3°	**1**‐*o*‐DBTO	60.9°
**1**‐*ms*‐DPOD	54.1°	51.7°	**1**‐*o*‐DPS	61.7°
**1** *‐ms*‐DBTO	56.1°	49.6°	**1**‐*o*‐TTO	58.4°
**1**‐*ms*‐DPS	55.7°	56.0°	**1**‐*m*‐DBTO	38.9°
**1**‐*ms*‐TTO	54.2°	57.3°	**1**‐*m*‐DPS	37.8°
**2**‐*ms*‐BN	53.1°	51.3°	**1**‐*m*‐TTO	37.3°

### Optoelectronic and photophysical properties

**Figure 4:**
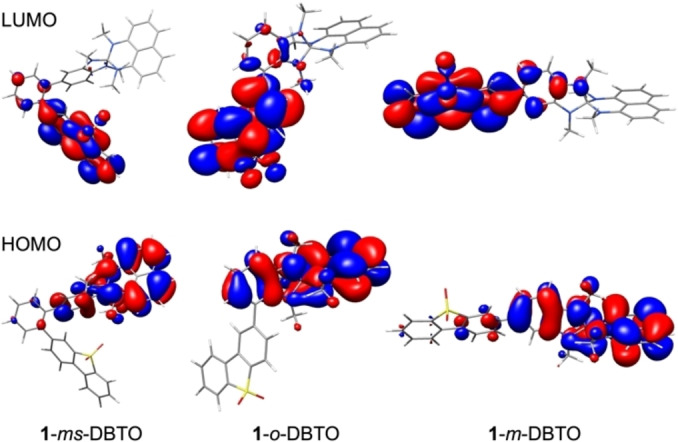
Calculated frontier molecular orbitals of **1**‐*ms*‐DBTO**, 1**‐*o*‐DBTO, and **1**‐*m*‐DBTO (B3LYP−D3/def2‐TZVP//PBEh‐3c).

DFT calculations provided information on the optoelectronic properties of the 12 spiro‐NN‐DA compounds. Selected calculated optoelectronic properties are listed in Table 2. The calculated HOMO energies are fairly similar for all compounds (between −5.13 eV and −4.25 eV), since the electron densities of the HOMOs are located on the spiro‐NN‐donor moieties (see Figure 4 for the HOMO and LUMO plots of three selected DA compounds, HOMO and LUMO plots of all other compounds can be found in the Supporting Information). The calculated LUMO energies depend on the type of acceptor chosen, and the LUMO electron densities are located on the acceptors in each case. The calculated HOMO‐LUMO‐energy differences (*E*_gap_) are related to the (emission) colors of the compounds and increase for the same donor‐acceptor pair from the *meta*‐spacer over the *ortho* to the *meta*‐connection. Of interest are further the splitting Δ*E*_ST_ between the lowest energy triplet (T_1_) and singlet state (S_1_) as well as the oscillator strength *f*. A large value *f* is beneficial to ensure efficient radiative decay from S_1_ to the ground state, while a small energy gap Δ*E*_ST_ <0.1 eV can enable thermal up‐conversion from the triplet to the singlet excited state by reverse intersystem‐crossing (RISC).^[39]^ For the spiro‐NN‐DA compounds with donor **1** investigated herein, these values are proportionate to each other. With the *meta*‐spacer in between donor and acceptor, the smallest Δ*E*_ST_ values between 8–118 meV are predicted with at the same time low oscillator strengths *f*=0.0005–0.0050. The *ortho*‐connection leads to intermediate values of Δ*E*_ST_=20–280 meV and *f*=0.0045–0.0105, while for the direct *meta‐*connection large singlet‐triplet splittings between 200 and 490 meV were calculated with high oscillator strengths of 0.0997–0.2918.

**Table 2:** Calculated optoelectronic properties of spiro‐NN‐DA compounds (B3LYP‐D3/def2‐TZVP//PBEh‐3c).

Compound	*E*_HOMO‐calc_ [eV]	*E*_LUMO‐calc_ [eV]	*E*_gap‐calc_ [eV]	Δ*E*_ST‐calc_ [meV]	*f* ^[a]^
**1**‐*ms*‐BN	−4.97	−2.25	2.72	108	0.0399
**1**‐*ms*‐DPOD	−5.01	−1.90	3.11	8	0.0760
**1**‐*ms*‐DBTO	−4.25	−2.71	1.54	20	0.0079
**1**‐*ms*‐DPS	−4.68	−1.77	2.91	10	0.0005
**1**‐*ms*‐TTO	−4.67	−2.45	2.22	10	0.0050
**2**‐*ms*‐BN	−5.13	−1.81	3.32	118	0.0670
**1**‐*o*‐DBTO	−4.98	−1.95	3.02	70	0.0105
**1**‐*o*‐DPS	−4.93	−1.69	3.24	280	0.0053
**1**‐*o*‐TTO	−5.02	−2.38	2.63	20	0.0045
**1**‐*m*‐DBTO	−4.99	−1.83	3.16	410	0.2681
**1**‐*m*‐DPS	−5.01	−1.60	3.41	490	0.2918
**1**‐*m*‐TTO	−5.08	−2.26	2.82	200	0.0997

[a] Oscillator strength for the first singlet excitation.

**Figure 5:**
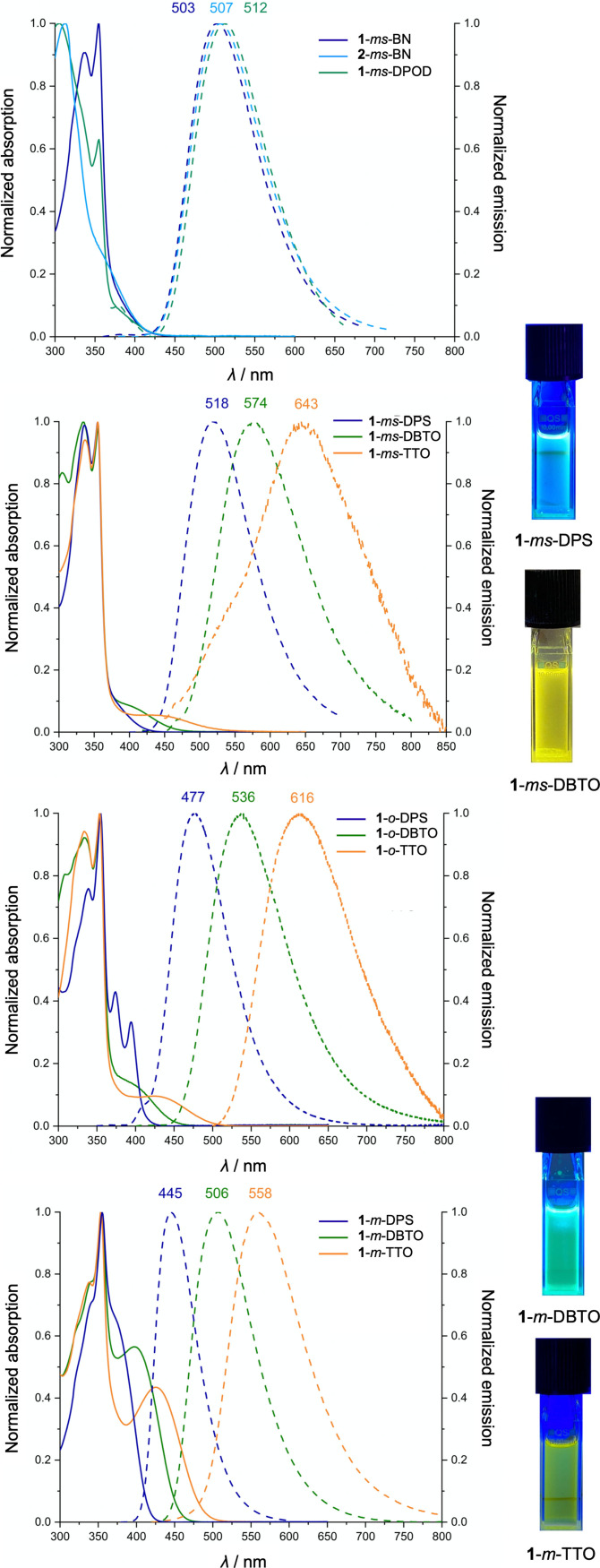
Normalized UV/Vis‐absorption (solid lines) and fluorescence spectra (dashed lines) of spiro‐NN‐DA compounds (10^−5^ m in toluene at rt) and photos of selected solutions irradiated with a hand‐held UV lamp.

As can be seen from the solution fluorescence spectra in Figure 5, the emission colors of the spiro‐NN‐DA compounds ranged from blue to red with emission maxima between 445 and 643 nm (see also Table 3). The highest energy blue emissions were found for **1**‐*o*‐DPS and **1**‐*m*‐DPS with the diphenylsulfone acceptor. The benzonitrile, diphenyloxadiazole, and dibenzothiophenedioxide acceptors led to green emissions with the exception of **1**‐*ms*‐DBTO (yellow). The thianthrenetetroxide acceptor caused a bathochromic shift to yellow (**1**‐*m*‐TTO), orange (**1**‐*o*‐TTO), or red emissions (**1**‐*ms*‐TTO). As DFT calculations had predicted, for the same acceptor, the band gap of the DA compounds decreased from the *meta*‐ over the *ortho‐*connection to the *meta*‐spacer.

**Table 3:** Experimental photophysical data of the spiro‐NN‐DA emitters.

Compound	*ƛ*_fluo, max_ [nm]^[a]^	PLQY_air_ [%]^[a]^	PLQY_Ar_ [%]^[a,b]^	PLQY_film_ [%]^[c]^	Δ*E*_ST_ [meV]^[d]^
**1**‐*ms*‐BN	503	49	97	54	475
**1**‐*ms*‐DPOD	512	37	74	52	407
**1**‐*ms*‐DPS	518	39	95	61	440
**1**‐*ms*‐DBTO	574	11	18	24	280
**1**‐*ms*‐TTO	643	1	2	3	283
**2**‐*ms*‐BN	507	45	96	52	465
**1**‐*o*‐DPS	477	57	90	54	685
**1**‐*o*‐DBTO	536	22	37	24	320
**1**‐*o*‐TTO	616	2	3	9	525
**1**‐*m*‐DPS	445	76	90	66	770
**1**‐*m*‐DBTO	506	78	99	6	360
**1**‐*m*‐TTO	558	73	92	7	610

[a] 10^−5^ m in toluene at rt. [b] in degassed toluene. [c] 10 wt% in *m*‐CBP. [d] S_1_ and T_1_ energies were determined from the onsets of fluorescence (at rt in toluene) and phosphorescence (at 77 K in toluene), respectively.

The photoluminescence quantum yields (PLQY) in toluene solution were high for most spiro‐NN‐DA compounds (PLQY_air_ in Table 3). In all cases, performing the measurement in degassed and oxygen‐free toluene led to a significant increase in PLQY (PLQY_Ar_) with values of 90 % or higher for seven of the 12 compounds. This indicates that the excited states participating in the photochemistry of the compounds are deactivated by oxygen. Usually, these are triplet excitons quenched by triplet O_2_, but it could also be singlet excitons quenched by singlet O_2_.^[40]^ The *meta*‐connected DA compounds **1**‐*m*‐DBTO, **1**‐*m*‐DPS, and **1**‐*m*‐TTO featured the highest PLQYs, likely related to the higher oscillator strengths of the first singlet excitations and probably also the emissions.

We next measured PLQY in thin films with 10 wt% spiro‐NN‐DA in *m*‐CBP (3,3′‐di(9H‐carbazol‐9‐yl)‐1,1′‐biphenyl) matrix. Interestingly, the quantum yields were lower than in solution for most compounds. The spiro‐NN‐DA compounds with the shortest wavelength absorptions featured the highest PLQYs in the thin‐films (54–66 %), namely **1**‐*ms*‐BN and **2**‐*ms*‐BN, **1**‐*ms*‐DPOD, and all three compounds with the diphenylsulfone acceptor **1**‐*ms*‐DPS, **1**‐*o*‐DPS, and **1**‐*m*‐DPS. This may be related to an energy alignment with the *m*‐CPB matrix for these molecules, which has to efficiently transfer its excited state energy to the emitter molecule. The fact that the PLQY of the spiro‐NN‐DA emitters decreased in the matrix may indicate that the CT state is reached through an intermolecular electron‐transfer process rather than an intramolecular one.

We next measured the transient photoluminescent decay for all 12 spiro‐NN‐DA emitters to investigate whether an indication for efficient RISC was found, which would result in a delayed component. In all cases, only first‐order decay was observed, corresponding to a prompt fluorescence component with lifetimes τ=2–14 ns. To rationalize this observation, we experimentally determined the S_1_−T_1_‐energy splittings Δ*E*_ST_ from the onsets of the solution fluorescence (at rt) and phosphorescence spectra (at 77 K, see Supporting Information) (Table 3). With values Δ*E*_ST_ between 280 and 770 meV, these energy differences are significantly higher than the calculated ones and too large to be efficiently overcome by thermally activated RISC. Such a deviation between calculated and experimental values has been reported in other instances.^[41]^ Hence, the fact that the PLQY increased upon deoxygenation of the solutions was probably due to singlet exciton quenching by singlet O_2_, which was thereby inhibited. Still, their high PLQYs in solution and tunable emission colors make the spiro‐NN‐DA compounds herein attractive as fluorescent emitters.

## Conclusions

In summary, we developed efficient synthetic routes to two monofunctionalized tetraaminospirenes as spiroconjugated donor units. Palladium‐catalyzed cross‐coupling to a variety of acceptor moieties in three different modes of connection between donor and acceptor afforded 12 spiro‐NN‐DA compounds. Their structures were investigated by X‐ray crystallography and/or DFT calculations, confirming spatial separation of the donor and acceptor part and as a consequence of the HOMO and LUMO. Photophysical measurements provided photoluminescence quantum yields of up to 99 %, depending on the connection mode between the donor and acceptor unit, and a range of emission colors. Time‐resolved emission spectroscopy showed that these compounds featured a prompt fluorescence with lifetimes up to 14 ns. The spiroconjugated tetraamine donors presented herein enable access to structurally and optoelectronically interesting DA‐type emitters.

## Experimental Section

Experimental Details can be found in the Supporting Information. Deposition Numbers 1949447 (for **1**‐*ms*‐DPS), 1949585 (for **1**‐*o*‐TTO), 1912248 (for **3** **a**), 1962741 (for **7** **a**), 2041007 (for **14** **a**), 2046064 (for **S1**, structure see Supporting Information), and 2043983 (for **S3**, structure see Supporting Information) contains the supplementary crystallographic data for this paper. These data are provided free of charge by the joint Cambridge Crystallographic Data Centre and Fachinformationszentrum Karlsruhe Access Structures service

## Conflict of interest

The authors declare no conflict of interest.

1

## Supporting information

As a service to our authors and readers, this journal provides supporting information supplied by the authors. Such materials are peer reviewed and may be re‐organized for online delivery, but are not copy‐edited or typeset. Technical support issues arising from supporting information (other than missing files) should be addressed to the authors.

Supporting Information

## Data Availability

The data that support the findings of this study are available from the corresponding author upon reasonable request.
