# Supplementary material for: Spiroconjugated Tetraaminospirenes as Donors in Color‐Tunable Charge‐Transfer Emitters with Donor‐Acceptor Structure
Source: Chemistry. 2021 Dec 16;28(6):e202104150. doi: 10.1002/chem.202104150 (PMC9299689; doi:10.1002/chem.202104150)
Supplement: Supplementary file 1 — Supporting Information [file CHEM-28-0-s001.pdf]

Chemistry–A European Journal

Supporting Information

Spiroconjugated Tetraaminospirenes as Donors in Color-Tunable Charge-Transfer Emitters with Donor-Acceptor Structure

David C. Grenz, Daniel Rose, Jan S. Wössner, Jennifer Wilbuer, Florin Adler, Mathias Hermann, Chin-Yiu Chan, Chihaya Adachi, and Birgit Esser*

Table of contents

1. Materials and Methods S1

2. Synthetic Manipulations..S4

2.1 Synthesis of Tetraaminospirenes S4

2.1.1 4-Bromo-*N*-methyl-2-nitroaniline (**S1**)... S4

2.1.2 4-Bromo-N^1-methylbenzene-1,2-diamine (**5a**)... S4

2.1.3 5-Bromo-1-methyl-1,3-dihydro-2*H*-benzimidazol-2-one (**6a**) S5

2.1.4 5-Bromo-2-chloro-1-methyl-1*H*-benzimidazole (**7a**) S5

2.1.5 5-Bromo-2-chloro-1,3-dimethyl-1*H*-benzo[*d*]imidazol-3-ium-tetrafluoroborate (**3a**) S6

2.1.6 2-Chloro-*N*-methyl-6-nitroaniline (**S2**)... S7

2.1.7 6-Chloro-N^1-methylbenzene-1,2-diamine (**5b**)... S7

2.1.8 7-Chloro-1-methyl-1,3-dihydro-2*H*-benzo[*d*]imidazol-2-one (**6b**)................. S8

2.1.9 7-Chloro-1-methyl-1*H*-benzo[*d*]imidazole (**7b**) S9

2.1.10 2,7-Dichloro-1,3-dimethyl-1*H*-benzo[*d*]imidazol-3-ium tetrafluoroborate (**3b**) S9

2.1.11 Synthesis of N^1,N^8-Dimethylnaphthalene-1,8-diamine (**10**)........................ S10

2.1.12 5-Bromo-1,1',3,3'-tetramethyl-1,3-dihydro-1'*H*,3'*H*-spiro[benzo[*d*]imidazole-2,2'-perimidine] (**8a**) S13

2.1.13 4-Chloro-1,1',3,3'-tetramethyl-1,3-dihydro-1'*H*,3'*H*-spiro[benzo[*d*]imidazole-2,2'-perimidine] (**8b**) S13

2.1.14 5-(2-Chlorophenyl)-1,1',3,3'-tetramethyl-1,3-dihydro-1'*H*,3'*H*-spiro[benzo[*d*]-imidazole-2,2'-perimidine] (**12**) S14

2.1.15 5-Bromo-1,1‘3,3‘-tetramethyl-1,1‘,3,3‘-tetrahydro-2,2‘-spirobi[benzo[*d*]imidazole] (**9**).. S15

2.2 Synthesis of Acceptor Molecules S16

2.2.1 Benzhydrazide (**S6**).. S16

2.2.2 *N*'-Benzoyl-4-bromobenzhydrazide (**15**).. S16

2.2.3 2-(4-Bromophenyl)-5-phenyl-1,3,4-oxadiazole (**S7**) S17

2.2.4 2-Phenyl-5-(4-(4,4,5,5-tetramethyl-1,3,2-dioxaborolan-2-yl)phenyl)-1,3,4-oxadiazole (**DPOD-Bpin**) S17

2.2.5 2-(2'-Bromo-[1,1'-biphenyl]-4-yl)-5-phenyl-1,3,4-oxadiazole (**S8**) S18

2.2.6 2-Phenyl-5-(2'-(4,4,5,5-tetramethyl-1,3,2-dioxaborolan-2-yl)-[1,1'-biphenyl]-4-yl)-1,3,4-oxadiazole (**14**)... S18

2.2.7 3-Bromodibenzo[*b*,*d*]thiophene 5,5-dioxide (**DBTO-Br**) and 3,7-Bromodibenzo[*b*,*d*]thiophene 5,5-dioxide (**Br-DBTO-Br**) S19

2.2.8 1-Bromo-4-(phenylsulfonyl)benzene (**DPS-Br**) S20

2.2.9 GP1 - General procedure for the borylation of acceptor molecules S21

2.2.10 3-(4,4,5,5-Tetramethyl-1,3,2-dioxaborolan-2-yl)dibenzo[*b*,*d*]thiophene 5,5-dioxide (**DBTO-Bpin**) S21

2.2.11 4,4,5,5-Tetramethyl-2-(4-(phenylsulfonyl)phenyl)-1,3,2-dioxaborolane (**DPS-Bpin**) S22

2.2.12 2-(4,4,5,5-Tetramethyl-1,3,2-dioxaborolan-2-yl)thianthrene 5,5,10,10-tetraoxide (**TTO-Bpin**) S22

2.2.13 2′-Bromo-[1,1′-biphenyl]-4-carbonitrile (**BN-Br**) S23

2.2.14 2'-(4,4,5,5-Tetramethyl-1,3,2-dioxaborolan-2-yl)-[1,1'-biphenyl]-4-carbonitrile (**20**) S24

2.3 Synthesis of Spiro-NN-Donor-Acceptor Compounds S25

2.3.1 2'-(1,1',3,3'-Tetramethyl-1,1',3,3'-tetrahydro-2,2'-spirobi[benzo[*d*]imidazol]-5-yl)-[1,1'-biphenyl]-4-carbonitrile (**2-*ms*-BN**) S25

2.3.2 2'-(1,1',3,3'-Tetramethyl-1,3-dihydro-1'*H*,3'*H*-spiro[benzo[*d*]imidazole-2,2'-perimidin]-5-yl)-[1,1'-biphenyl]-4-carbonitrile (**1-*ms*-BN**) S26

2.3.3 2-Phenyl-5-(2'-(1,1',3,3'-tetramethyl-1,3-dihydro-1'*H*,3'*H*-spiro[benzo[*d*]imidazole-2,2'-perimidin]-5-yl)-[1,1'-biphenyl]-4-yl)-1,3,4-oxadiazole (**1-*ms*-DPOD**) S27

2.3.4 GP2 - general procedure for the preparation of donor-acceptor scaffolds via Suzuki-Miyaura-coupling S28

2.3.5 3-(2-(1,1',3,3'-Tetramethyl-1,3-dihydro-1'*H*,3'*H*-spiro[benzo[*d*]imidazole-2,2'-perimidin]-5-yl)phenyl)dibenzo[*b*,*d*]thiophene 5,5-dioxide (**1-*ms*-DBTO**)... S28

2.3.6 1,1',3,3'-Tetramethyl-5-(4'-(phenylsulfonyl)-[1,1'-biphenyl]-2-yl)-1,3-dihydro-1'*H*,3'*H*-spiro[benzo[*d*]imidazole-2,2'-perimidine] (**1-*ms*-DPS**) S29

2.3.7 2-(2-(1,1',3,3'-Tetramethyl-1,3-dihydro-1'*H*,3'*H*-spiro[benzo[*d*]imidazole-2,2'-perimidin]-5-yl)phenyl)thianthrene 5,5,10,10-tetraoxide (**1-*ms*-TTO**) S30

2.3.8 3-(1,1',3,3'-Tetramethyl-1,3-dihydro-1'*H*,3'*H*-spiro[benzo[*d*]imidazole-2,2'-perimidin]-5-yl)dibenzo[*b*,*d*]thiophene 5,5-dioxide (**1-*m*-DBTO**)................ S31

2.3.9 1,1',3,3'-Tetramethyl-5-(4-(phenylsulfonyl)phenyl)-1,3-dihydro-1'*H*,3'*H*-spiro[benzo[*d*]- imidazole-2,2'-perimidine] (**1-*m*-DPS**) S32

2.3.10 2-(1,1',3,3'-Tetramethyl-1,3-dihydro-1'*H*,3'*H*-spiro[benzo[*d*]imidazole-2,2'-perimidin]-5-yl)thianthrene 5,5,10,10-tetraoxide (**1-*m*-TTO**) S32

2.3.11 3-(1,1',3,3'-Tetramethyl-1,3-dihydro-1'*H*,3'*H*-spiro[benzo[*d*]imidazole-2,2'-perimidin]-4-yl)dibenzo[*b*,*d*]thiophene 5,5-dioxide (**1-*o*-DBTO**)................. S33

2.3.12 1,1',3,3'-Tetramethyl-4-(4-(phenylsulfonyl)phenyl)-1,3-dihydro-1'*H*,3'*H*-spiro[benzo-[*d*]imidazole-2,2'-perimidine] (**1-*o*-DPS**) S34

2.3.13 2-(1,1',3,3'-Tetramethyl-1,3-dihydro-1'*H*,3'*H*-spiro[benzo[*d*]imidazole-2,2'-perimidin]-4-yl)thianthrene 5,5,10,10-tetraoxide (**1-*o*-TTO**) S35

3. Single Crystal X-ray Diffraction S36

4. Optical Properties S43

5. DFT Calculations S55

5.1 Methods S55

5.2 Calculated Transitions S56

5.3 Cartesian Coordinates of Calculated Structures S60

6. NMR Spectra S73

7. References S111

1. Materials and Methods

Chemicals were purchased from ABCR, ACROS-ORGANICS, ALFA-AESAR, CHEMPUR, FLUROCHEM, ROTH, SIGMA-ALDRICH or TCI and used directly without further purification unless otherwise noted. Moisture- or oxygen-sensitive reactions were carried out in dried glassware, heated under vacuum (10^{-3} mbar), using standard Schlenk techniques in a dry argon atmosphere (Argon 5.0 from SAUERSTOFFWERKE FRIEDRICHSHAFEN). Anhydrous solvents (CH_2Cl_2, THF) were obtained from an M. BRAUN solvent purification system (MB-SPS-800) and stored over molecular sieves (3 Å). Other anhydrous solvents were obtained by drying over activated molecular sieves (3 Å) for several days.[1] Cyclohexane for flash chromatography was purchased in technical grade and purified by distillation using a rotary evaporator. Other solvents were purchased and used in analytical or HPLC grade.

Analytical thin layer chromatography was carried out using silica gel-coated aluminum plates with a fluorescence indicator (MERCK 60 F_{254} or MACHERY-NAGEL ALUGRAM Xtra SIL G/UV_{254}). Detection was carried out by using short wavelength UV light (λ_{max} = 254 nm).

Flash column chromatography was carried out using silica gel 60, grain size 40–63 µm (230–400 mesh) from MACHERY-NAGEL. In some cases, an automated flash chromatography system (GRACE Reveleris X2) was employed using prepacked columns (silica gel, 25 g to 80 g, 30 µm grain size) from INTERCHIM (INTERCHIM Puriflash Silica HP 30 µm flash column).

NMR spectra were recorded at 300 K, unless otherwise noted, on the following spectrometers: BRUKER *Avance III HD 300* [300.1 MHz (^{1}H)], BRUKER *Avance Neo 400* with a *Prodigy* CryoProbe [400.1 MHz (^{1}H), 100.6 MHz (^{13}C)] and BRUKER *Avance III HD 500* [500.3 MHz (^{1}H), 125.8 MHz (^{13}C)]. Chemical shifts are reported in parts per million (ppm, δ = scale) relative to the signal of tetramethylsilane (δ = 0.00 ppm). ^{1}H NMR spectra are referenced to tetramethylsilane as an internal standard or the residual proton signal of the respective solvent: $CDCl_3$: δ = 7.26 ppm; C_6D_6: δ = 7.16 ppm, 1,1,2,2-tetrachloroethane-d_2: δ = 5.91 ppm, CD_2Cl_2: δ = 5.32 ppm, DMSO-d_6: δ = 2.50 ppm, acetone-d_6: δ = 2.05 ppm, toluene-d_8: δ = 2.03 ppm, CD_3CN: δ = 1.94 ppm. ^{13}C NMR spectra are referenced to the following signals: $CDCl_3$: δ = 77.16 ppm; C_6D_6: δ = 128.06 ppm, 1,1,2,2-tetrachloroethane-d_2: δ = 74.20 ppm, CD_2Cl_2: δ = 53.84 ppm, DMSO-d_6: δ = 39.52 ppm, acetone-d_6: δ = 29.84 ppm, toluene-d_8:

δ = 20.43 ppm, CD_3CN: δ = 1.32 ppm.[2] Analysis followed first order, and the following abbreviations for multiplets were used: singlet (s), doublet (d), multiplet (m) and combinations thereof *i.e.* doublet of doublets (dd). Coupling constants (*J*) are given in Hertz [Hz].

High resolution mass spectra were measured on a THERMO FISHER SCIENTIFIC Exactive via electrospray ionization (ESI) or atmospheric pressure chemical ionization (APCI) with an orbitrap analyzer.

UV/Vis absorption spectra were measured on a SHIMADZU UV-1800 using *Quartz (Suprasil)* cuvettes (10 mm path length) from HELLMA ANALYTICS.

Cyclic voltammograms (CVs) and differential pulse voltammograms (DPVs) were measured inside an argon-filled glovebox using a PGSTAT128N potentiostat by METROHM AUTOLAB. As working electrode, a glassy carbon disc electrode (2 mm diameter) was used, as counter electrode a platinum rod was used, as reference electrode a $Ag/AgNO_3$ electrode containing a silver wire immersed in an inner chamber filled with 1 M $AgNO_3$ and 0.1 M n-Bu_4NPF_6 in anh. CH_3CN or a silver wire was used. The analyte solution contained 10 mL of solvent (anh. CH_2Cl_2 or THF) with 0.1 M n-Bu_4NPF_6 and the specified analyte concentration. The ferrocene/ferrocenium redox couple was used as internal reference. HOMO and LUMO levels were calculated using the following equations: E_{LUMO} (eV) = $-(E_{i,Fc}+x_{Red})$ (with $E_{i,Fc}$ = 4.8 eV (ionization energy of ferrocene)[3]; x_{Red} = onset of the first reduction peak, calibrated *vs*. Fc/Fc^+ in eV), E_{HOMO} (eV) = $-(E_{i,Fc}+x_{Ox})$ (with x_{Ox} = onset of the first oxidation peak, calibrated *vs*. Fc/Fc^+ in eV).

UV/Vis Absorption Spectroscopy

UV/Vis absorption spectra were measured on a Shimadzu UV-1800 or a PERKINELMER LAMBDA 950 UV/Vis spectrophotometer using a quartz cuvette with a path length of 10 mm.

Fluorescence Spectroscopy

Room temperature fluorescence spectra were measured on a JASCO Spectrofluorometer FP-8600 or a Perkin Elmer *LS 55* fluorescence spectrometer using a quartz cuvette with a path length of 10 mm. Photoluminescence spectra at 77 K were measured on a JASCO Spectrofluorometer FP-8600 using a 5 mm quartz NMR tube as cuvette.

Absolute photoluminescence quantum yields were determined using a HAMAMATSU UV-NIR absolute PL Quantum Yield Spectrometer (13534). Measurements in solution were either degassed or performed under ambient conditions as mentioned. Measurements in thin films were carried out under an argon atmosphere.

2. Synthetic Manipulations

2.1 Synthesis of Tetraaminospirenes

2.1.1 4-Bromo-*N*-methyl-2-nitroaniline (**S1**)

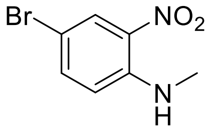

To 4-bromo-1-fluoro-2-nitrobenzene (**4a**, 5.04 g, 22.9 mmol) a solution of methylamine in EtOH (33% w/w, 8.5 mL, 6.43 g, 68.3 mmol, 3.1 eq.) was slowly added at −20 °C, and the resulting mixture was stirred at rt for 18 h. After addition of sat. aq. $CaCO_3$ (10 mL) the mixture was extracted with CH_2Cl_2 (5 × 100 mL). The combined organic layers were washed with H_2O (100 mL), dried (Na_2SO_4), and the solvent was removed under reduced pressure to afford **S1** (5.29 g, 22.9 mmol, quant.) as an orange solid.

***R*f** 0.70 (cyclohexane/EtOAc: 1/1); **m.p.** = 115 °C; **^{1}H NMR** (300 MHz, $CDCl_3$): δ = 8.32 (d, J = 2.4 Hz, 1H), 8.00 (br, 1H), 7.51 (ddd, J = 9.1, 2.4, 0.7 Hz, 1H), 6.76 (d, J = 9.1 Hz, 1H), 3.02 (m, 3H) ppm; **^{13}C NMR** (101 MHz, $CDCl_3$): δ = 145.4, 139.1, 132.5, 129.0, 115.3, 106.5, 30.0 ppm; **HRMS** (pos. APCI): m/z calcd. for $C_7H_8O_2N_2Br$ 230.9764 $[M+H]^+$, found 230.9765.

2.1.2 4-Bromo-N^1-methylbenzene-1,2-diamine (**5a**)

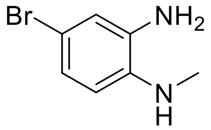

4-Bromo-*N*-methyl-2-nitroaniline (**S1**, 5.04 g, 21.8 mmol) was dissolved in EtOH (200 mL) and aq. HCl (37% w/w, 54.0 mL) was added. Afterwards Sn powder (10.7 g, 89.5 mmol, 3.9 eq.) was carefully added, and the mixture was refluxed for 2.5 h. The mixture was cooled to 0 °C and aq. NaOH (40 % w/w) was added until the mixture had basic pH. H_2O (200 mL) and CH_2Cl_2 (150 mL) were added, and the mixture was extracted with CH_2Cl_2 (2 × 70 mL) and Et_2O (2 × 70 mL). The combined organic layers were dried ($MgSO_4$). After evaporation of the solvent the residue was dried *in vacuo* to afford **5a** (3.94 g, 19.6 mmol, 90%) as an off-white solid.

***R*f** 0.72 (CH_2Cl_2); **^{1}H NMR** (300 MHz, $CDCl_3$): δ = 6.93 (dd, J = 8.4, 2.2 Hz, 1H), 6.81 (d, J = 2.2 Hz 1H), 6.49 (d, J = 8.4 Hz, 1H), 3.33 (br, 3H), 2.83 (s, 3H) ppm; **^{13}C NMR** (76 MHz, $CDCl_3$): δ = 136.4, 136.1, 123.2, 119.1, 113.9, 112.1, 31.6 ppm; **HRMS** (pos. APCI): *m/z* calcd. for $C_7H_{10}BrN_2^+$ 201.0022 [M+H]$^+$, found 201.0025.

2.1.3 5-Bromo-1-methyl-1,3-dihydro-2*H*-benzimidazol-2-one (**6a**)

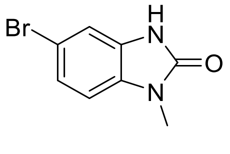

A suspension of 4-bromo-*N*^{1}-methylbenzene-1,2-diamine (**5a,** 2.00 g, 9.93 mmol) and carbonyldiimidazole (1.94 g, 12.0 mmol, 1.2 eq.) in 1,4-dioxane (20 mL) was stirred at 40 °C for 3 h. The mixture was cooled to 15 °C, and the resulting precipitate was filtered off and washed with cold cyclohexane (50 mL) and cold CH_2Cl_2 (20 mL) to afford **6a** (2.03 g, 8.93 mmol, 90%) as an off-white solid.

***R*f** 0.66 (EtOAc); **^{1}H NMR** (300 MHz, DMSO-d_6): δ = 11.01 (br, 1H), 7.18 (dd, J = 8.3, 1.9 Hz, 1H), 7.11 (dd, J = 1.9, 0.4 Hz, 1H), 7.05 (d, J = 8.3 Hz, 1H), 3.26 (s, 3H) ppm; **^{13}C NMR** (101 MHz, DMSO-d_6): δ = 154.2, 130.3, 129.7, 122.9, 112.4, 111.1, 109.2, 26.5 ppm; **HRMS** (pos. APCI): *m/z* calcd. for $C_8H_8BrN_2O^+$ 226.9815 [M+H], found 226.9816.

2.1.4 5-Bromo-2-chloro-1-methyl-1*H*-benzimidazole (**7a**)

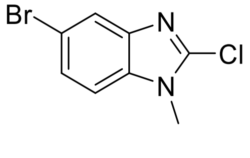

5-Bromo-1-methyl-1,3-dihydro-2*H*-benzimidazol-2-one (**6a**, 444 mg, 1.96 mmol) was suspended in $POCl_3$ (20 mL), and the resulting mixture was refluxed under argon for 22 h. After cooling to 0 °C H_2O (5 mL) was slowly added. Sat. aq. NaOH was added until pH 12 was reached. The mixture was extracted with CH_2Cl_2 (3 × 100 mL). The combined organic extracts were washed with brine (3 × 180 mL), dried ($MgSO_4$) and evaporated to dryness. The residue was purified by flash chromatography (SiO_2, cyclohexane/EtOAc: 1/1) to give product **7a** (471 mg, 1.93 mmol, 98%) as a white solid.

***R*$_f$** 0.63 (cyclohexane/EtOAc: 1/2); **^{1}H NMR** (400 MHz, DMSO-*d*$_6$): δ = 7.83 (dd, *J* = 1.8, 0.5 Hz, 1H), 7.41 (dd, *J* = 8.7, 1.8 Hz, 1H), 7.17 (d, *J* = 8.7 Hz, 1H), 3.78 (s, 3H) ppm; **^{13}C NMR** (101 MHz, DMSO-*d*$_6$): δ = 143.0, 142.2, 134.8, 126.4, 122.5, 115.9, 110.6, 30.8 ppm; **HRMS** (pos. ESI): *m*/*z* calcd. for $C_8H_7N_2BrCl^+$ 246.9455 [M+H]$^+$, found 246.9454.

2.1.5 5-Bromo-2-chloro-1,3-dimethyl-1*H*-benzo[*d*]imidazol-3-ium-tetrafluoroborate (**3a**)

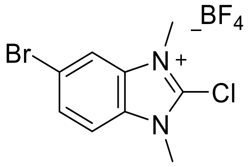

5-Bromo-2-chloro-1-methyl-1*H*-benzimidazole (**7a**, 500 mg, 2.04 mmol) and trimethyloxonium tetrafluoroborate (347 mg, 2.35 mmol, 1.2 eq.) were dissolved in 1,2-dichloroethane (50 mL) and refluxed under argon for 3 h. The mixture was stirred at rt for 14 h. After filtration, the residue was washed with cold cyclohexane (3 × 10 mL) and then suspended in cyclohexane/EtOAc (10/1) and stirred for another 16 h. After filtration, the residue was dried *in vacuo* to afford **3a** (673 mg, 1.94 mmol, 95%) as a white powder.

^{1}H NMR (500 MHz, CD_3CN): δ = 8.11 (dd, *J* = 1.7, 0.5 Hz, 1H), 7.85 (dd, *J* = 8.9, 1.7 Hz, 1H), 7.76 (d, *J* = 8.9 Hz, 1H), 3.99 (s, 3H), 3.97 (s, 3H) ppm; **^{13}C NMR** (101 MHz, CD_3CN): δ = 133.3, 131.5, 131.4, 121.0, 117.1, 115.5, 33.9 ppm; **^{11}B NMR** (96 MHz, DMSO-*d*$_6$): δ = −1.30 ppm; **^{19}F NMR** (282 MHz, DMSO-*d*$_6$): δ = −148.24, −148.30 ppm; **HRMS** (pos. ESI): *m*/*z* calcd. For $C_9H_9N_2BrCl^+$ 258.9632 [M−BF$_4$]$^+$, found 258.9631.

2.1.6 2-Chloro-*N*-methyl-6-nitroaniline (**S2**)

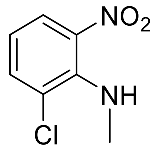

1-Chloro-2-fluoro-3-nitrobenzene (**4b**, 26.2 g, 149 mmol) was dissolved in EtOH (100 mL), and a solution of methylamine in EtOH (33% w/w, 40.3 mL, 428 mmol, 3.0 eq.) was added in portions. The reaction mixture was stirred at rt for 19 h, then diluted with CH_2Cl_2 (300 mL) and washed with sat. aq. Na_2CO_3 (300 mL). The aqueous layer was extracted with CH_2Cl_2 (2 × 200 mL) and the combined organic layers were washed with H_2O (300 mL), brine (300 mL) and dried over $MgSO_4$. Removing the organic solvents under reduced pressure yielded the title compound **S2** as an orange solid (27.6 g, 148 mmol, 99%).

***R*f** (cyclohexane/EtOAc: 8/1): 0.61; **^{1}H NMR** (400 MHz, $CDCl_3$): δ = 7.90 (dd, *J* = 8.5, 1.6 Hz, 1H), 7.48 (dd, *J* = 7.7, 1.6 Hz, 1H), 6.70 (dd, *J* = 8.5, 7.7 Hz, 1H), 3.10 (s, 3H) ppm; **^{13}C NMR** (101 MHz, $CDCl_3$): δ = 143.6, 137.8, 136.3, 125.6, 123.8, 117.2, 34.2 ppm; **HRMS** (pos. ESI): *m*/*z* calcd. for $C_7H_8ClN_2O_2$ $[M+H]^+$ 187.0269, found 187.0269.

2.1.7 6-Chloro-N^1-methylbenzene-1,2-diamine (**5b**)

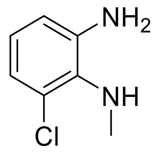

2-Chloro-*N*-methyl-6-nitroaniline (**S2**, 24.1 g, 129 mmol) was dissolved in EtOH (250 mL), and $SnCl_2 \cdot 2\,H_2O$ (132 g, 581 mmol, 4.5 eq.) was added in portions. The reaction mixture was refluxed for 3 d. The reaction mixture was then allowed to cool to rt, quenched by addition of aq. NaOH until pH > 10 was reached and diluted with CH_2Cl_2 (500 mL). The organic layer was decanted and washed with H_2O (3 × 500 mL) and brine (1 × 500 mL). The solution was dried over $MgSO_4$ and the solvents removed under reduced pressure to afford the title compound **5b** (20.1 g, 128 mmol, 99%) as a reddish oil. The product was used in the following step without further purification.

***R*f** (CH_2Cl_2): 0.49; **^{1}H NMR** (400 MHz, $CDCl_3$): δ = 6.80 (dd, *J* = 7.6, 7.6 Hz, 1H), 6.76 (dd, *J* = 8.0, 1.8 Hz, 1H), 6.60 (dd, *J* = 7.6, 1.8 Hz, 1H), 3.71 (br, 3H), 2.70 (s, 3H) ppm; **^{13}C NMR** (101 MHz, $CDCl_3$): δ = 142.9, 133.4, 129.0, 124.5, 118.8, 114.0, 34.0 ppm; **HRMS** (pos. ESI): *m*/*z* calcd. for $C_7H_{10}ClN_2$ $[M+H]^+$ 157.0528, found 157.0525.

2.1.8 7-Chloro-1-methyl-1,3-dihydro-2*H*-benzo[*d*]imidazol-2-one (**6b**)

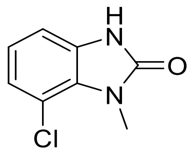

6-Chloro-*N*^{1}-methylbenzene-1,2-diamine (**5b**, 20.0 g, 128 mmol) was dissolved in 1,4-dioxane (250 mL), and carbonyldiimidazole (34.8 g, 153 mmol, 1.2 eq.) was added. The reaction mixture was stirred at rt for 17 h. The resulting precipitate was filtered off, washed with cyclohexane and cold $CH_2Cl_2$to yield the title compound **6b** as an offwhite solid (7.17 g, 39.4 mmol, 31%).

***R*f** (CH_2Cl_2): 0.87; **^{1}H NMR** (400 MHz, DMSO-*d*$_6$): δ = 11.15 (br, 1H), 7.03–6.92 (m, 3H), 3.55 (s, 3H) ppm; **^{13}C NMR** (101 MHz, DMSO-*d*$_6$): δ = 154.2, 130.3, 126.6, 121.83, 121.81, 113.2, 107.8, 28.7 ppm; **HRMS** (pos. ESI): *m*/*z* calcd. for $C_8H_8ClN_2O$ $[M+H]^+$ 183.0320, found 183.0320.

2.1.9 7-Chloro-1-methyl-1*H*-benzo[*d*]imidazole (**7b**)

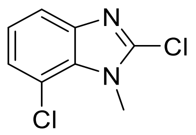

7-Chloro-1-methyl-1,3-dihydro-2*H*-benzo[*d*]imidazol-2-one (**6b**, 7.13 g, 39.2 mmol) was suspended in $POCl_3$, and the resulting mixture was refluxed for 16 h. The reaction mixture was then allowed to cool to rt and carefully poured onto a mixture of ice and water. Aq. NaOH was added until pH > 12 was reached. The mixture was extracted with CH_2Cl_2 (500 mL), dried over $MgSO_4$ and filtered over a pad of SiO_2. Evaporation of the organic solvents yielded the title compound **7b** as an off-white solid (5.86 g, 29.1 mmol, 75%).

***R*f** (CH_2Cl_2): 0.28; **^{1}H NMR** (500 MHz, $CDCl_3$): δ = 7.57 (dd, *J* = 7.9, 1.1 Hz, 1H), 7.22 (dd, *J* = 7.9, 1.1 Hz, 1H), 7.15 (dd, *J* = 7.9, 7.9 Hz, 1H), 4.11 (s, 3H) ppm; **^{13}C NMR** (126 MHz, $CDCl_3$): δ = 143.6, 142.7, 131.7, 124.7, 123.3, 118.4, 116.1, 33.3 ppm; **HRMS** (pos. ESI): *m*/*z* calcd. for $C_8H_7Cl_2N_2$ $[M+H]^+$ 200.9981, found 200.9981.

2.1.10 2,7-Dichloro-1,3-dimethyl-1*H*-benzo[*d*]imidazol-3-ium tetrafluoroborate (**3b**)

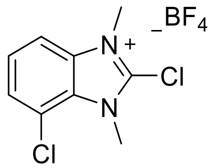

2,4-Dichloro-1-methyl-1H-benzo[*d*]imidazole (**7b**, 4.80 g, 23.9 mmol) was added to a stirred solution of trimethyloxonium tetrafluoroborate (3.88 g, 26.3 mmol, 1.1 eq.) in degassed, anh. 1,2-dichloroethane (115 mL), and the mixture was refluxed for 15 h. The resulting precipitate was filtered off, washed with cyclohexane and dried *in vacuo* to afford the title compound **3b** as an off-white powder (5.98 g, 19.7 mmol, 83%).

^{1}H NMR (500 MHz, CD_3CN): δ = 7.79 (dd, *J* = 8.3, 1.1Hz, 1H), 7.71 (dd, *J* = 8.0, 1.1 Hz, 1H), 7.65 (dd, *J* = 8.3, 8.0 Hz, 1H), 4.29 (s, 3H), 4.01 (s, 3H) ppm; **^{13}C NMR** (126 MHz, CD_3CN): δ = 134.4, 129.8, 129.0, 128.8, 120.1, 113.2, 36.6, 34.3 ppm; **HRMS** (pos. ESI): *m*/*z* calcd. for $C_9H_9Cl_2N_2$ $[M-BF_4]^+$ 215.0137, found 215.0136.

2.1.11 Synthesis of N^1,N^8-Dimethylnaphthalene-1,8-diamine (**10**)

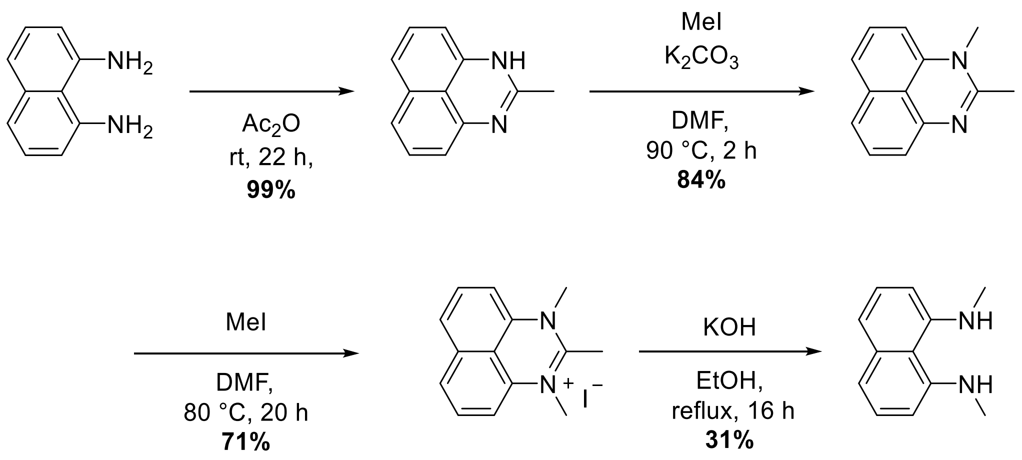

2-Methyl-1*H*-perimidine (S3)

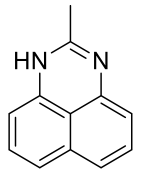

Naphthalene-1,8-diamine (103 g, 652 mmol) was added in small portions to Ac_2O (500 mL) and stirred at rt for 22 h. The reaction mixture was poured onto H_2O, the resulting precipitate was filtered off and washed with H_2O to afford the title compound **S3** as a yellow solid (118 g, 647 mmol, 99%).

***R*f** (EtOAc): 0.16; **^{1}H NMR** (500 MHz, $CDCl_3$): δ = 9.99 (br, 1H), 7.09–6.90 (m, 4H), 6.47 (dd, J = 6.7, 1.6 Hz, 2H), 2.25 (s, 3H) ppm; **^{13}C NMR** (126 MHz, $CDCl_3$): δ = 155.3, 137.6, 135.0, 128.2, 121.1, 120.7, 108.2, 22.6 ppm; **HRMS** (pos. ESI): m/z calcd. for $C_{12}H_{11}N_2$ $[M+H]^+$ 183.0917, found 183.0919.

1,2-Dimethyl-1*H*-perimidine (S4)

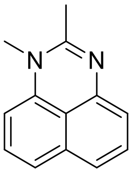

2-Methyl-1*H*-perimidine (**S3**, 60.0 g, 330 mmol), K_2CO_3 (93.7 g, 660 mmol, 2.0 eq.), and MeI (41.0 mL, 660 mmol, 2.0 eq.) were suspended in DMF (500 mL) and stirred at 90 °C for 2 h, before another portion of MeI (20.5 mL, 330 mmol, 1.0 eq.) was added. After 15 min, H_2O (500 mL) was added to form a yellow precipitate and the reaction mixture was allowed to cool to rt. Excessive MeI was quenched by addition of aq. NH_3 (30% w/w, 200 mL). The reaction mixture was diluted with H_2O (1500 mL) and the resulting precipitate was filtered off and washed with H_2O and acetone to afford the title compound **S4** as a yellow solid (54.3 g, 277 mmol, 84%).

***R*$_f$** (EtOAc): 0.20; **^{1}H NMR** (500 MHz, DMSO-d_6): δ = 7.23−7.18 (m, 2H), 7.13–7.09 (m, 2H), 6.64 (dd, J = 7.5, 1.0 Hz, 1H), 6.36 (dd, J = 7.7, 1.0 Hz, 1H), 3.19 (s, 3H), 2.29 (s, 3H) ppm; **^{13}C NMR** (126 MHz, DMSO-d_6): δ = 155.0, 143.4, 139.7, 134.6, 128.6, 127.9, 121.5, 188.7, 118.1, 113.3, 101.0, 33.8, 23.0 ppm; **HRMS** (pos. ESI): m/z calcd. for $C_{13}H_{13}N_2$ $[M+H]^+$ 197.1074, found 197.1074.

1,2,3-Trimethyl-1*H*-perimidin-3-ium iodide (S5)

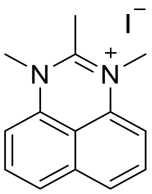

1,2-Dimethyl-1*H*-perimidine (**S4**, 54.3 g, 277 mmol) was suspended in DMF (500 mL). MeI (104 mL, 1.66 mol, 6.0 eq.) was added, and the reaction mixture was stirred at 80 °C for 20 h. The precipitate was decanted, filtered and washed with CH_2Cl_2. Excessive MeI in the filtrate was quenched by addition of aq. NH_3 (30% w/w, 300 mL). The title compound **S5** was afforded as a yellow salt (66.3 g, 196 mmol, 71%).

^{1}**H NMR** (500 MHz, DMSO-d_6): δ = 7.66 (dd, J = 8.4, 0.7 Hz, 2H), 7.56 (dd, J = 8.4, 7.8 Hz, 2H), 7.20 (dd, J = 7.8, 0.7 Hz, 2H), 3.62 (s, 6H), 2.78 (s, 3H) ppm; ^{13}**C NMR** (126 MHz, DMSO-d_6): δ = 162.6, 133.8, 113.5, 128.3, 123.1, 119.7, 108.3, 37.6, 19.9 ppm; **HRMS** (pos. ESI): m/z calcd. For $C_{14}H_{15}N_2$ $[M{-}I]^+$ 211.1230, found 211.1224.

***N*¹,*N*⁸-Dimethylnaphthalene-1,8-diamine (10)**

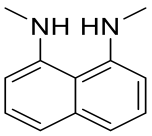

1,2,3-Trimethyl-1*H*-perimidin-3-ium iodide (**S5**, 66.3 g, 196 mmol) was added in portions to a solution of KOH (220 g, 3.92 mol, 20 eq.) in EtOH (500 mL) and the resulting mixture was refluxed for 16 h. The reaction mixture was then allowed to cool to rt, the solvents were evaporated and the crude material was dissolved in CH_2Cl_2 (500 mL). H_2O (500 mL) was added and the aqueous layer was extracted with CH_2Cl_2 (2 × 500 mL). The combined organic extracts were washed with H_2O (500 mL), dried over $MgSO_4$ and the solvents were removed under reduced pressure to yield the crude product. This was then filtered over a short column of silica gel (dichloromethane) to afford the title compound **10** as a yellow solid (11.4 g, 61 mmol, 31%).

$\boldsymbol{R_f}$ (cyclohexane/CH_2Cl_2: 1/1): 0.33; ^{1}**H NMR** (500 MHz, DMSO-d_6): δ = 7.20 (dd, J = 7.8, 7.8 Hz, 2H), 7.06 (dd, J = 7.8, 1.1 Hz, 2H), 6.49 (dd, J = 7.8, 1.1 Hz, 2H), 6.29 (q, J = 5.1 Hz, 2H), 2.75 (d, J = 5.1 Hz, 6H) ppm; ^{13}**C NMR** (126 MHz, DMSO-d_6): δ = 147.7, 136.3, 126.2, 117.6, 116.1, 105.8, 31.9 ppm; **HRMS** (pos. ESI): m/z calcd. for $C_{12}H_{15}N_2$ $[M{+}H]^+$ 187.1230, found 187.1231.

2.1.12 5-Bromo-1,1',3,3'-tetramethyl-1,3-dihydro-1'*H*,3'*H*-spiro[benzo[*d*]imidazole-2,2'-perimidine] (**8a**)

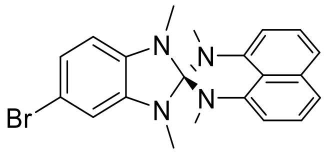

The title compound was prepared according to a modified procedure by QUAST and co-workers.[4] 5-Bromo-2-chloro-1,3-dimethyl-1*H*-benzo[*d*]imidazol-3-ium tetrafluoroborate (**3a**, 500 mg, 1.44 mmol) and N^1,N^8-dimethylnaphthalene-1,8-diamine (**10**, 542 mg, 2.88 mmol, 2.0 eq.) were dissolved in a degassed mixture of anh. CH_3CN (70 mL) and anh. NEt_3 (10 mL) and stirred at rt for 5 d. The reaction mixture was diluted with CH_2Cl_2 (200 mL), washed with H_2O (200 mL), and the aqueous layer was extracted with CH_2Cl_2 (3 × 200 mL). The solvents were removed under reduced pressure, and the crude material was purified by flash column chromatography (SiO_2, cyclohexane/CH_2Cl_2: 3/1) to afford the title compound **8a** as an off-white solid (447 mg, 1.09 mmol, 76%).

$\boldsymbol{R_f}$ (cyclohexane/CH_2Cl_2: 3/1): 0.33; **^{1}H NMR** (500 MHz, $CDCl_3$): δ = 7.33 (dd, J = 8.1, 7.9 Hz, 2H), 7.15 (dd, J = 8.1, 0.8 Hz, 2H), 6.77 (dd, J = 7.9, 1.9 Hz, 1H), 6.44 (d, J = 1.9 Hz, 1H), 6.41 (dd, J = 7.9, 0.8 Hz, 2H), 6.20 (d, J = 7.9 Hz, 1H), 2.714 (s, 3H), 2.707 (s, 3H), 2.66 (s, 6H) ppm; **^{13}C NMR** (126 MHz, $CDCl_3$): δ = 140.7, 137.6, 135.5, 134.2, 127.8, 119.7, 116.4, 110.8, 109.3, 106.2, 104.6, 102.3, 100.8, 31.0, 27.22, 27.19 ppm; **HRMS** (pos. ESI): m/z calcd. for $C_{21}H_{22}BrN_4$ $[M+H]^+$ 409.1023, found 409.1026.

2.1.13 4-Chloro-1,1',3,3'-tetramethyl-1,3-dihydro-1'*H*,3'*H*-spiro[benzo[*d*]imidazole-2,2'-perimidine] (**8b**)

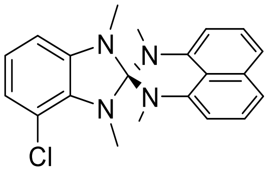

The title compound was prepared according to a modified procedure of QUAST and co-workers.[4] 7-Chloro-1,3-dimethyl-1*H*-benzo[*d*]imidazol-3-ium tetrafluoroborate (**3b**, 1.18 g, 3.89 mmol) and N^1,N^8-dimethylnaphthalene-1,8-diamine (**10**, 1.45 g, 7.78 mmol, 2.0 eq.)

were dissolved in a mixture of degassed, anh. CH_3CN (27 mL) and anh. NEt_3 (27 mL). The reaction mixture was stirred at 0 °C for 5 h and at rt for 16 h. The solvents were removed under reduced pressure, and the crude product was purified by flash column chromatography (SiO_2, cyclohexane/CH_2Cl_2: 8/1) to afford the title compound **8b** as an off-white solid (960 mg, 2.63 mmol, 68%).

***R*f** (cyclohexane/CH_2Cl_2: 8/1): 0.30; **^{1}H NMR** (500 MHz, acetone-d_6): δ = 7.31 (dd, J = 8.3, 7.8 Hz, 2H), 7.11 (dd, J = 8.3, 0.8 Hz, 2H), 6.58 (dd, J = 8.4, 7.4 Hz, 1H), 6.51 (dd, J = 8.4, 1.2 Hz, 1H), 6.49 (dd, J = 7.8, 0.8Hz, 2H), 6.37 (dd, J = 7.4, 1.2 Hz, 1H), 3.06 (s, 3H), 2.75 (s, 3H), 2.71 (s, 6H) ppm; **^{13}C NMR** (126 MHz, acetone-d_6): δ = 141.9, 139.4, 135.2, 132.1, 128.8, 120.1, 119.6, 117.0, 111.6, 109.8, 107.3, 101.7, 101.3, 31.3, 27.5 ppm (one carbon signal is not visible); **HRMS** (pos. ESI): m/z calcd. for $C_{21}H_{22}ClN_4$ $[M+H]^+$ 365.1528, found 365.1530.

2.1.14 5-(2-Chlorophenyl)-1,1',3,3'-tetramethyl-1,3-dihydro-1'*H*,3'*H*-spiro[benzo[*d*]-imidazole-2,2'-perimidine] (**12**)

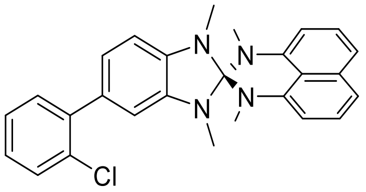

2-Chlorophenylboronic acid (300 mg, 1.92 mmol, 1.5 eq.), $PdCl_2dppf$ (70 mg, 96 µmol, 5 mol%), **8a** (524 mg, 1.28 mmol) and Na_2CO_3 (1.22 g, 11.5 mmol, 6.0 eq.) were combined in a glove box, dissolved in degassed 1,4-dioxane/H_2O (2/1, 60 mL) and stirred under argon at 100 °C. After 17 h the reaction mixture was allowed to cool to rt, filtered through a pad of silica gel, diluted with CH_2Cl_2 (50 mL), and the aqueous layer was extracted with CH_2Cl_2 (2 × 50 mL). The combined organic extracts were dried over $MgSO_4$ and the solvents were removed under reduced pressure. The crude material was purified by flash column chromatography (SiO_2, cyclohexane/CH_2Cl_2: 5/3) to yield the title compound **12** as an off-white powder (489 mg, 1.11 mmol, 87%).

***R*f** (cyclohexane/CH_2Cl_2: 5/3): 0.37; **^{1}H NMR** (400 MHz, acetone-d_6): δ = 7.50–7.46 (m, 1H), 7.44–7.40 (m, 1H), 7.37–7.25 (m, 4H), 7.10 (dd, J = 8.3, 0.9 Hz, 2H), 6.73 (dd, J = 7.7, 1.7 Hz, 1H), 6.52 (d, J = 1.7 Hz, 1H), 6.50–6.45 (m, 3H), 2.76 (s, 6H), 2.70 (s, 6H) ppm; **^{13}C NMR** (101 MHz, acetone-d_6): δ = 142.9, 142.1, 137.2, 137.1, 135.3, 133.2, 132.7, 130.9, 129.6,

128.8, 128.7, 128.0, 120.0, 116.9, 111.9, 107.2, 103.7, 101.8, 101.7, 31.5, 27.6, 27.5 ppm; **HRMS** (pos. ESI): m/z calcd. for $C_{27}H_{25}ClN_4$ $[M+H]^+$ 441.1841, found 441.1839.

2.1.15 5-Bromo-1,1‘3,3‘-tetramethyl-1,1‘,3,3‘-tetrahydro-2,2‘-spirobi[benzo[*d*]imidazole] (**9**)

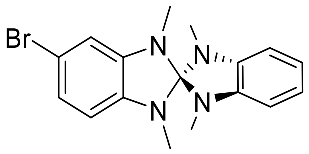

5-Bromo-2-chloro-1,3-dimethyl-1*H*-benzo[*d*]imidazol-3-ium-tetrafluoroborate (**3a,** 99.8 mg, 287 µmol) and N^1,N^2-dimethylbenzene-1,2-diamine (**11**, 62.1 mg, 456 µmol, 1. 6 eq.) were dissolved in a degassed and anh. mixture of CH_3CN (15.0 mL) and NEt_3 (2.50 mL) under argon. The mixture was stirred at rt for 7 d. H_2O (10 mL) was added and after extraction with EtOAc (3 × 30 mL) the combined organic layers were washed (2 × 50 mL), dried ($MgSO_4$), filtered and evaporated to dryness. The residue was purified by flash chromatography (SiO_2, cyclohexane/EtOAc/NEt_3: 20/10/1) to afford **9** (55.3 mg, 154 µmol, 54%) as an off-white solid.

***R*f** 0.57 (cyclohexane/EtOAc: 4/1); **¹H NMR** (500 MHz, $CDCl_3$): δ = 6.75 (dd, J = 7.8, 1.9 Hz, 1H), 6.70–6.66 (m, 2H), 6.40 (d, J = 1.9 Hz, 1H), 6.36–6.33 (m, 2H), 6.16 (d, J = 7.8 Hz, 1H), 2.66 (s, 6H), 2.64 (s, 6H) ppm; **¹³C NMR** (126 MHz, $CDCl_3$): δ = 136.9, 135.5, 134.8, 119.8, 117.9, 116.6, 109.3, 104.7, 102.4, 101.8, 27.4, 27.3 ppm; **HRMS** (pos. ESI): m/z calcd. for $C_{17}H_{20}N_4Br$ 359.0866 $[M+H]^+$, found 359.0869.

2.2 Synthesis of Acceptor Molecules

2.2.1 Benzhydrazide (**S6**)

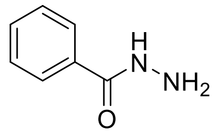

Methyl benzoate (501 mg, 3.65 mmol) was dissolved in EtOH (20 mL), and 4-bromobenzoyl chloride (1.08 g, 18.5 mmol, 5.1 eq.) was slowly added. The resulting mixture was refluxed for 6 h. The reaction mixture was allowed to cool to rt and the solvent was removed under reduced pressure. H_2O (6 mL) was added, and the residue was extracted with EtOAc (3 × 50 mL). The combined organic layers were dried ($MgSO_4$), filtered and evaporated to dryness to afford **S6** (318 mg, 2.34 mmol, 64%) as a white powder.

^{1}H NMR (400 MHz, DMSO-d_6): δ = 9.74 (br, 1H), 7.83–7.80 (m, 2H), 7.53–7.44 (m, 3H), 4.47 (br, 2H) ppm; **^{13}C NMR** (101 MHz, DMSO-d_6): δ = 156.1, 123.6, 121.3, 118.5, 117.2 ppm; **HRMS** (pos. APCI): *m/z* calcd. for $C_7H_8N_2ONa$ 159.0529 $[M+Na]^+$, found 159.0529.

2.2.2 *N*'-Benzoyl-4-bromobenzhydrazide (**15**)

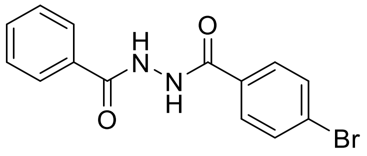

Benzhydrazide (**S6**, 880 mg, 6.47 mmol, 1.0 eq.) and Na_2CO_3 (1.29 g, 12.2 mmol, 1.9 eq.) were dissolved in NMP (5 mL). 4-Bromobenzoyl chloride (1.38 g, 6.30 mmol) was dissolved in NMP (5 mL) and slowly added to the other solution within 90 min. The resulting mixture was stirred at rt for 41 h. The mixture was poured into H_2O, and the resulting precipitate was filtered off and washed with H_2O (50 mL) to afford **15** (1.93 g, 6.05 mmol, 96%) as a white solid.

^{1}H NMR (300 MHz, DMSO-d_6): δ = 10.58 (br, 2H), 7.90–7.84 (m, 4H), 7.72–7.69 (m, 2H), 7.59–7.48 (m, 3H) ppm; **^{13}C NMR** (101 MHz, DMSO-d_6): δ = 164.9, 164.4, 133.1, 132.6, 131.5, 131.4, 129.4, 128.4, 127.2, 125.0 ppm; **HRMS** (pos. APCI): *m/z* calcd. for $C_{14}H_{11}{}^{81}BrN_2O_2Na$ 340.9896 $[M+Na]^+$, found 340.9698.

2.2.3 2-(4-Bromophenyl)-5-phenyl-1,3,4-oxadiazole (**S7**)

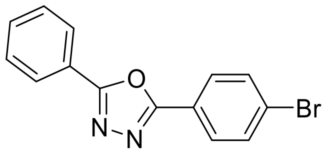

N'-Benzoyl-4-bromobenzhydrazide (**15**, 1.50 mg, 4.70 mmol) was suspended in $POCl_3$ (50 mL), refluxed for 5 h, cooled to rt and stirred at rt for 18 h. Ice (100 mL) was added, and aq. NaOH (1M) was added to the suspension until a basic pH was reached. The mixture was extracted with CH_2Cl_2 (3 × 1000 mL), and the combined organic extracts were dried ($MgSO_4$). The solvent was removed under reduced pressure to afford **S7** (1.27 g, 4.23 mmol, 96%) as a white solid.

***R*f** 0.54 (cyclohexane/EtOAc: 4/1); **^{1}H NMR** (300 MHz, $CDCl_3$): δ = 8.18–8.10 (m, 2H), 8.05–7.99 (m, 2H), 7.71–7.66 (m, 2H), 7.58–7.51 (m, 3H) ppm; **^{13}C NMR** (101 MHz, $CDCl_3$): δ = 164.9, 164.1, 132.6, 132.0, 129.3, 128.5, 127.2, 126.6, 124.0, 123.1 ppm; **HRMS** (pos. ESI): *m/z* calcd. for $C_{14}H_{10}BrN_2O$ 300.9971 $[M+H]^+$, found 300.9971.

2.2.4 2-Phenyl-5-(4-(4,4,5,5-tetramethyl-1,3,2-dioxaborolan-2-yl)phenyl)-1,3,4-oxadiazole (**DPOD-Bpin**)

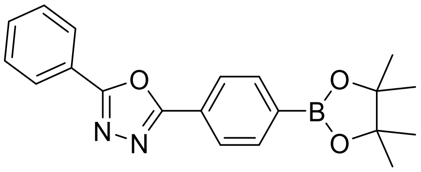

Compound **S7** (400 mg, 1.33 mmol), Pd(dppf)Cl_2 (60.0 mg, 82.0 µmol, 8 mol%), bis(pinacolato)diboron (742 mg, 2.92 mmol, 2.2 eq.) and KOAc (766 mg, 7.97 mmol, 6. 0 eq.) were combined in a glovebox, dissolved in anh. 1,4-dioxane (10 mL) and stirred under argon at 80 °C for 4 d. H_2O (30 mL) and CH_2Cl_2 (50 mL) were added, the phases separated, and the aqueous phase was further extracted with CH_2Cl_2 (3 × 40 mL). The combined organic extracts were dried ($MgSO_4$), filtered and evaporated to dryness. The residue was washed with MeOH to afford **DPOD-Bpin** (1.22 g, 3.50 mmol, 71%) as a light brown solid.

***R*f** 0.50 (cyclohexane/EtOAc: 1/2); **^{1}H NMR** (300 MHz, $CDCl_3$): δ = 8.18–8.12 (m, 4H), 7.98–7.95 (m, 2H), 7.58–7.51 (m, 3H), 1.38 (s, 12H) ppm; **^{13}C NMR** (101 MHz, $CDCl_3$): δ = 164.8, 164.7, 135.4, 131.8, 129.2, 127.1, 126.2, 126.1, 124.1, 84.3, 25.1 ppm (one signal missing of C_{quart}-B); **HRMS** (pos. ESI): *m/z* calcd. for $C_{20}H_{22}BN_2O_3$ 349.1718 $[M+H]^+$, found 349.1713.

2.2.5 2-(2'-Bromo-[1,1'-biphenyl]-4-yl)-5-phenyl-1,3,4-oxadiazole (**S8**)

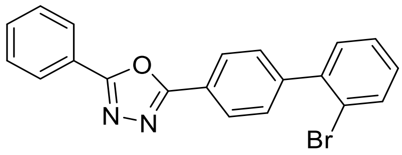

Compound **DPOD-Bpin** (100 mg, 287 µmol), $Pd(PPh_3)_4$ (15 mg, 13 µmol, 4 mol%) and Na_2CO_3 (174 mg, 1.65 mmol, 5.7 eq.) were combined in a glovebox and added to a solution of 1,2-dibromobenzene (343 mg, 1.45 mmol, 5.1 eq.) in a degassed mixture of THF (20 mL) and H_2O (20 mL). The resulting mixture was refluxed for 17 h. CH_2Cl_2 (35 mL) was added, the phases separated, and the aqueous phase was further extracted with CH_2Cl_2 (3 × 30 mL). The combined organic extracts were dried ($MgSO_4$), the solvent removed under reduced pressure and the residue purified by flash chromatography (SiO_2, cyclohexane/EtOAc: 8/1) to afford **S8** (94.2 mg, 250 µmol, 87%) as a white solid.

$\boldsymbol{R}_{\mathbf{f}}$ 0.33 (cyclohexane/EtOAc: 8/1); **^{1}H NMR** (400 MHz, $CDCl_3$) δ = 8.24–8.19 (m, 2H), 8.18–8.15 (m, 2H), 7.71 (ddd, J = 8.1, 1.2, 0.5 Hz, 1H), 7.62–7.58 (m, 2H), 7.58–7.52 (m, 3H), 7.41–7.34 (m, 2H), 7.28–7.23 (m, 1H) ppm; **^{13}C NMR** (101 MHz, $CDCl_3$) δ = 164.8, 164.5, 144.5, 141.5, 133.4, 131.8, 131.1, 130.3, 129.5, 129.2, 127.7, 127.1, 126.7, 124.1, 123.3, 122.4 ppm; **HRMS** (pos. ESI): m/z calcd. for $C_{20}H_{14}BrN_2O$ 387.0284 $[M+H]^+$, found 387.0290.

2.2.6 2-Phenyl-5-(2'-(4,4,5,5-tetramethyl-1,3,2-dioxaborolan-2-yl)-[1,1'-biphenyl]-4-yl)-1,3,4-oxadiazole (**14**)

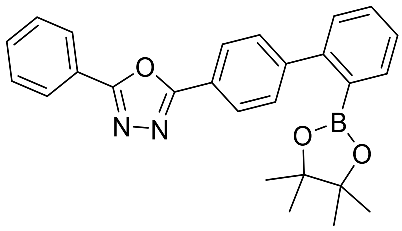

Compound **S8** (300 mg, 795 µmol), $Pd(dppf)Cl_2$ (30 mg, 40.6 µmol, 5 mol%), bis(pinacolato)diboron (242 mg, 953 µmol, 1.0 eq.) and KOAc (465 mg, 4.84 mmol, 6.1 eq.) were combined in a glovebox, dissolved in anh. 1,4-dioxane (10 mL) and stirred under argon at 85 °C for 20 h. H_2O (20 mL) and CH_2Cl_2 (20 mL) were added, the phases separated, and the aqueous phase was further extracted with CH_2Cl_2 (3 × 10 mL). The combined organic extracts were washed with H_2O (50 mL), dried ($MgSO_4$), filtered and the solvent was removed under

reduced pressure. The residue was purified by flash chromatography (SiO_2, cyclohexane/EtOAc: 5/1) to afford **14** as a white solid.

***R*f** 0.43 (cyclohexane/EtOAc: 5/1); ^{1}H NMR (300 MHz, $CDCl_3$) δ = 8.18–8.20 (m, 3H), 7.82–7.77 (m, 1H), 7.57–7.54 (m, 5H), 7.52–7.48 (m, 1H), 7.41–7.41 (m, 3H), 1.22 (s, 12H) ppm; ^{13}C NMR (101 MHz, $CDCl_3$) δ = 164.8, 147.0, 146.6, 142.3, 135.2, 133.0, 131.8, 130.5, 130.1, 129.2, 128.1, 127.1, 126.5, 124.3, 122.5, 83.6, 25.2, 24.8 ppm; HRMS (pos. ESI): *m*/*z* calcd. for $C_{26}H_{26}BN_2O_3$ 425.2031 $[M+H]^+$, found 425.2033.

2.2.7 3-Bromodibenzo[*b*,*d*]thiophene 5,5-dioxide (**DBTO-Br**) and 3,7-Bromodibenzo[*b*,*d*]thiophene 5,5-dioxide (**Br-DBTO-Br**)

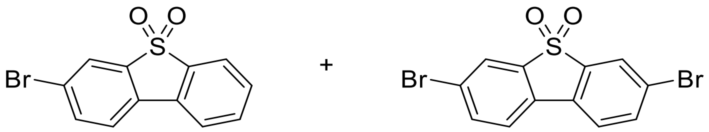

The title compounds were prepared according to a modified procedure by BRUST and co-workers.[5] Dibenzo[*b*,*d*]thiophene-5,5-dioxide (2.00 g, 9.25 mmol) was dissolved in conc. H_2SO_4 (50 mL), then NBS (1.66 g, 9.25 mmol, 1.0 eq.) was added portionwise, and the reaction mixture was stirred at rt for 24 h. The mixture was poured into H_2O at 0 °C, and the resulting white precipitate was filtered off, washed with water and dried under reduced pressure. The crude material was purified by flash column chromatography (SiO_2, cyclohexane/CH_2Cl_2: 2/1 to 1/2) to afford DBTO-Br (614 mg, 1.64 mmol, 18%) and 3,7-Bromodibenzo[*b*,*d*]thiophene 5,5-dioxide (1140 mg, 3.86 mmol, 42%) as off-white solids.

DBTO-Br: ***R*f** (cyclohexane/CH_2Cl_2: 3/1): 0.23; **^{1}H NMR** (400 MHz, DMSO-*d*6): δ = 8.31 (dd, *J* = 1.9, 0.5 Hz, 1H), 8.22–8.12 (m, 2H), 8.02–7.98 (m, 2H), 7.81 (ddd, *J* = 7.6, 7.6, 1.1 Hz, 1H), 7.68 (ddd, *J* = 7.6, 7.6, 1.1 Hz, 1H) ppm; **^{13}C NMR** (101 MHz, DMSO-*d*6): δ = 138.6, 137.3, 136.7, 134.7, 131.2, 130.0, 129.9, 124.9, 124.6, 123.7, 122.8, 122.0 ppm; **HRMS** (pos. ESI): *m*/*z* calcd. for $C_{12}H_8BrO_2S$ $[M+H]^+$ 294.9423, found 294.9424.

Br-DBTO-Br: ***R*f** (cyclohexane/CH_2Cl_2: 3/1): 0.30; **^{1}H NMR** (400 MHz, DMSO-*d*6): δ = 8.35 (d, *J* = 1.9 Hz, 2H), 8.16 (d, *J* = 8.3 Hz, 2H), 8.02 (dd, *J* = 8.3, 1.9 Hz, 2H) ppm; **^{13}C NMR** (101 MHz,

DMSO-d_6): δ = 138.3, 137.5, 129.2, 125.1, 124.8, 124.0 ppm; **HRMS** (pos. APCI): *m*/*z* calcd. for $C_{12}H_{10}NBr_2O_2S$ $[M+NH_4]^+$ 389.8794, found 389.8790.

2.2.8 1-Bromo-4-(phenylsulfonyl)benzene (**DPS-Br**)

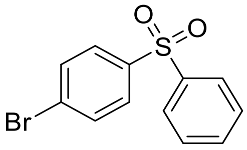

CuI (238 mg, 1.25 mmol, 5 mol%), K_2CO_3 (6.91 g, 50.0 mmol, 2.0 eq.), and 4-bromoiodobenzene (7.07 g, 25.0 mmol) were added to a flame-dried flask under argon. ^{i}PrOH (25 mL), ethylene glycol (2.80 mL, 50.0 mmol, 2.0 eq.) and thiophenol (2.55 mL, 25.0 mmol, 1.0 eq.) were added at rt, and the reaction mixture was heated to 80 °C and stirred for 20 h. The reaction mixture was filtered through a pad of silica gel and rinsed with cyclohexane. The filtrate was evaporated to dryness to yield the crude product as a colorless oil, which was suspended in HOAc (25 mL) and oxidized by addition of aq. H_2O_2 (30% w/w, 7.7 mL, 75 mmol, 3.0 eq.). The reaction mixture was stirred at rt for 24 h. The resulting white precipitate was filtered off, washed with H_2O and dried under reduced pressure. The crude material was purified by flash column chromatography (SiO_2, cyclohexane/EtOAc: 9/1) to yield the title compound **DPS-Br** as a white solid (3.75 g, 12.6 mmol, 51%).

$\boldsymbol{R_f}$ (cyclohexane/EtOAc: 9/1): 0.27; **^{1}H NMR** (400 MHz, DMSO-d_6): δ = 7.98–7.95 (m, 2H), 7.91–7.86 (m, 2H), 7.84–7.81 (m, 2H), 7.73–7.68 (m, 1H), 7.66–7.60 (m, 2H) ppm; **^{13}C NMR** (101 MHz, DMSO-d_6): δ = 140.6, 140.3, 133.9, 132.8, 129.8, 129.3, 127.8, 127.3 ppm; **HRMS** (APCI): *m*/*z* calcd. for $C_{12}H_9BrO_2S$ $[M]^+$ 295.9507, found 295.9509.

2.2.9 GP1 - General procedure for the borylation of acceptor molecules

Aryl bromide, the respective palladium catalyst, bis(pinacolato)diboron and the respective base were combined in a glovebox, dissolved in degassed, anh. 1,4-dioxane and stirred under argon at 80 °C. After consumption of the starting material the reaction mixture was allowed to cool to rt and filtered through a pad of silica gel. The organic layers were separated and evaporated to dryness. The crude products were dissolved in a minimum amount of CH_2Cl_2 and triturated with *n*-hexane. The resulting solids were filtered off and washed with cold MeOH to afford the respective aryl boronic ester.

2.2.10 3-(4,4,5,5-Tetramethyl-1,3,2-dioxaborolan-2-yl)dibenzo[*b*,*d*]thiophene 5,5-dioxide (**DBTO-Bpin**)

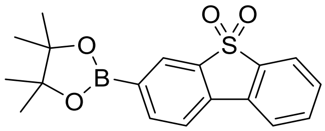

DBTO-Bpin was synthesized following **GP1**, using **DBTO-Br** (565 mg, 1.91 mmol), $PdCl_2$dppf (70.0 mg, 960 µmol, 5 mol%), bis(pinacolato)diboron (778 mg, 3.06 mmol, 1.6 eq.) and KOAc (1.17 g, 11.9 mmol, 6.3 eq.) in degassed, anh. 1,4-dioxane (20 mL). The reaction was worked up after 26 h to afford **DBTO-Bpin** as an off-white powder (457 mg, 1.34 mmol, 70%).

***R*f** (EtOAc): 0.29; **^{1}H NMR** (500 MHz, $CDCl_3$): δ = 8.30–8.27 (m, 1H), 8.05 (dd, *J* = 7.8, 0.9 Hz, 1H), 7.84–7.82 (m, 1H), 7.82–7.81 (m, 1H), 7.78 (dd, *J* = 7.6, 0.8 Hz, 1H), 7.64 (ddd, *J* = 7.6, 7.6, 1.1 Hz, 1H), 7.54 (ddd, *J* = 7.6, 7.6, 1.1 Hz, 1H), 1.36 (s, 12H) ppm; **^{13}C NMR** (126 MHz, $CDCl_3$): δ = 140.2, 138.3, 137.3, 133.9, 131.7, 130.9, 128.5, 122.3, 122.0, 120.9, 84.7, 25.0 ppm (two carbon signals are overlapping/not visible); **HRMS** (pos. ESI): *m*/*z* calcd. for $C_{18}H_{20}BO_4S$ $[M+H]^+$ 343.1170, found 343.1172.

2.2.11 4,4,5,5-Tetramethyl-2-(4-(phenylsulfonyl)phenyl)-1,3,2-dioxaborolane (**DPS-Bpin**)

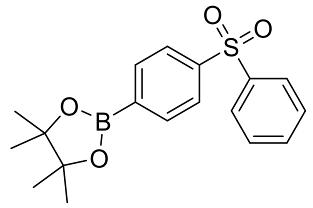

DPS-Bpin was synthesized following **GP1**, using **DPS-Br** (1.50 g, 5.05 mmol), $PdCl_2$dppf (185 mg, 0.253 mmol, 5 mol%), bis(pinacolato)diboron (2.05 g, 8.08 mmol, 1.6 eq.) and KOAc (3.10 g, 31.6 mmol, 6.3 eq.) in degassed anh. 1,4-dioxane (50 mL). The reaction was worked up after 27 h to afford **DPS-Bpin** as an off-white powder (600 mg, 1.74 mmol, 35%).

***R*f** (EtOAc): 0.11; **^{1}H NMR** (500 MHz, $CDCl_3$): δ = 7.94–7.93 (m, 1H), 7.92 (s, 5H), 7.57–7.52 (m, 1H), 7.51–7.46 (m, 1H), 1.32 (s, 12H) ppm; **^{13}C NMR** (126 MHz, $CDCl_3$): δ = 143.8, 141.7, 135.6, 133.3, 129.4, 127.8, 126.8, 84.6, 25.0 ppm (one carbon signal ist not visible); **HRMS** (pos. ESI): *m/z* calcd. for $C_{18}H_{22}BO_4S$ $[M+H]^+$ 345.1327, found 345.1329.

2.2.12 2-(4,4,5,5-Tetramethyl-1,3,2-dioxaborolan-2-yl)thianthrene 5,5,10,10-tetraoxide (**TTO-Bpin**)

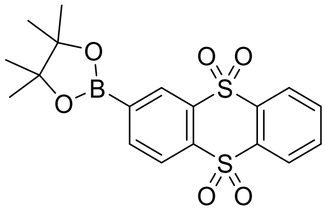

TTO-Bpin was synthesized following **GP1**, using 2-bromothianthrene-5,5,10,10-tetroxide (**TTO-Br**, 684 mg, 1.90 mmol), $PdCl_2$dppf (139 mg, 0.190 mmol, 10 mol%), bis(pinacolato)diboron (677 mg, 2.67 mmol, 1.4 eq.) and KOAc (1.12 g, 11.4 mmol, 6.0 eq.) in degassed, anh. 1,4-dioxane (15 mL). The reaction was worked up after 18 h to afford **TTO-Bpin** as a white solid (672 mg, 1.65 mmol, 87%).

***R*f** (cyclohexane/EtOAc: 4/1): 0.12; **^{1}H NMR** (500 MHz, $CDCl_3$): δ = 8.65 (d, *J* = 1.3 Hz, 1H), 8.29–8.24 (m, 2H), 8.23–8.18 (m, 2H), 7.85–7.79 (m, 2H), 1.36 (s, 12H) ppm; **^{13}C NMR** (126 MHz, $CDCl_3$): δ = 141.3, 139.9, 139.6, 138.7, 133.9, 133.8, 131.9, 126.2, 126.1, 125.1, 85.3, 25.0 ppm

(two carbon signals are overlapping/not visible); **HRMS** (pos. ESI): *m/z* calcd. for $C_{18}H_{19}O_6BNaS_2$ $[M+Na]^+$ 429.0608, found 429.0613.

2.2.13 2′-Bromo-[1,1′-biphenyl]-4-carbonitrile (**BN-Br**)

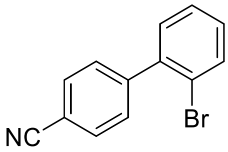

(4-Cyanophenyl)boronic acid (300 mg, 2.04 mmol), 1,2-dibromobenzene (3.98 g, 16.5 mmol, 8.1 eq.), Na_2CO_3 (1.33 g, 12.5 mmol, 6.1 eq.) and $Pd(PPh_3)_4$ (50 mg, 41.3 µmol, 2 mol%) were combined in a glovebox, dissolved in a degassed mixture of THF (12 mL) and H_2O (6 mL) and stirred under argon at 100 °C for 20 h. H_2O (30 mL) and CH_2Cl_2 (30 mL) were added, and the mixture was extracted with CH_2Cl_2 (3 × 30 mL). The combined organic layers were dried ($MgSO_4$), filtered and dried *in vacuo*. The residue was purified by two consecutive steps of flash column chromatography (SiO_2, cyclohexane/EtOAc: 4/1, SiO_2, cyclohexane/CH_2Cl_2: 4/1) to afford **BN-Br** (360 mg, 1.39 mmol, 68%) as a white solid.

***R*f** 0.30 (cyclohexane/CH_2Cl_2: 4/1); **^{1}H NMR** (500 MHz, $CDCl_3$): δ = 7.74–7.71 (m, 2H), 7.70 (ddd, *J* = 8.0, 1.3, 0.4 Hz, 1H), 7.54–7.51 (m, 2H), 7.40 (ddd, *J* = 7.7, 7.3, 1.3 Hz, 1H), 7.31–7.24 (m, 2H) ppm; **^{13}C NMR** (101 MHz, $CDCl_3$): δ = 145.8, 140.9, 133.6, 132.0, 131.0, 130.4, 129.9, 127.8, 122.2, 118.9, 111.8 ppm; **HRMS** (pos. ESI): *m/z* calcd. for $C_{13}H_9BrN^+$ 257.9913 $[M+H]^+$, found 257.9913.

2.2.14 2'-(4,4,5,5-Tetramethyl-1,3,2-dioxaborolan-2-yl)-[1,1'-biphenyl]-4-carbonitrile (**20**)

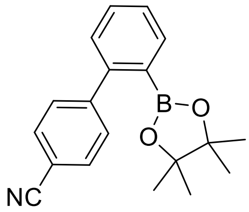

Compound **BN-Br** (199 mg, 771 µmol), Pd(dppf)Cl_2 (46 mg, 63 µmol, 8 mol%), bis(pinacolato)diboron (419 mg, 1.63 mmol, 2.1 eq.) and KOAc (474 mg, 4.88 mmol, 6.3 eq.) were combined in a glovebox, dissolved in anh. 1,4-dioxane (5 mL) and stirred under argon at 80 °C for 16 h. H_2O (5 mL) and CH_2Cl_2 (10 mL) were added, the mixture was extracted with CH_2Cl_2 (3 × 10 mL) and washed with H_2O (2 × 20 mL). The combined organic layers were dried ($MgSO_4$), filtered and evaporated *in vacuo*. The residue was purified by two consecutive steps of flash column chromatography (SiO_2, cyclohexane/CH_2Cl_2: 1/1, SiO_2, cyclohexane/EtOAc/MeOH: 16/2/1) to afford **20** (90 mg, 294 µmol, 37%) as a white solid.

***R*$_f$** 0.35 (cyclohexane/CH_2Cl_2: 1/1); **^{1}H NMR** (500 MHz $CDCl_3$): *δ* = 7.80 (dd, *J* = 7.0, 1.4 Hz, 1H), 7.66 (m, 2H), 7.49–7.46 (m, 2H), 7.41–7.37 (m, 2H), 7.32 (ddd, *J* = 7.7, 1.4, 0.5 Hz, 1H), 1.20 (s, 12H) ppm; **^{13}C NMR** (126 MHz, $CDCl_3$): *δ* = 126.8, 117.1, 114.1, 112.1, 110.2, 109.3, 108.8, 107.6, 106.9, 106.2, 63.2, 62.7 ppm; **HRMS** (pos. ESI): *m/z* calcd. for $C_{19}H_{20}BNNa^+$ 328.1479 $[M+Na]^+$, found 328.1478.

2.3 Synthesis of Spiro-NN-Donor-Acceptor Compounds

2.3.1 2'-(1,1',3,3'-Tetramethyl-1,1',3,3'-tetrahydro-2,2'-spirobi[benzo[*d*]imidazol]-5-yl)-[1,1'-biphenyl]-4-carbonitrile (**2-*ms*-BN**)

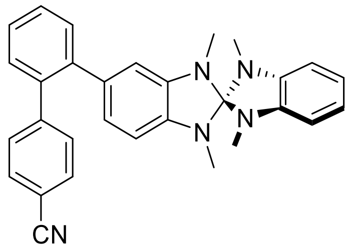

Spiro-bromide **9** (500 mg, 1.19 mmol), boronic ester **20** (552 mg, 1.81 mmol, 1.3 eq.), $Pd(PPh_3)_4$ (80 mg, 69 µmol, 5 mol%) and Na_2CO_3 (757 mg, 7.14 mmol, 6.0 eq.) were combined in a glovebox, dissolved in a degassed mixture of THF (40 mL) and H_2O (20 mL) and refluxed under argon for 48 h. CH_2Cl_2 (40 mL) was added, the aqueous phase was separated, further extracted with CH_2Cl_2 (3 × 30 mL), and the combined organic extracts were dried ($MgSO_4$). The solvent was removed under reduced pressure and the residue was purified by flash column chromatography (SiO_2, cyclohexane/EtOAc: 10/1). Sublimation at 230 °C ($p < 10^{-3}$ mbar) afforded **2-*ms*-BN** (201 mg, 440 µmol, 37%) as a yellow solid.

Rf 0.34 (cyclohexane/EtOAc: 10/1); **^{1}H NMR** (500 MHz, C_6D_6) δ 7.62–7.53 (m, 1H), 7.25–7.22 (m, 1H), 7.08–6.92 (m, 6H), 6.85–6.84 (m, 2H) 6.54–6.42 (m, 1H), 6.27–6.26 (m, 2H), 6.04–5.99 (m, 1H), 5.93–5.86 (m, 1H), 2.29–2.28 (m, 6H), 2.24–2.23 (m, 3H), 2.13 (s, 3H); **HRMS** (pos. ESI): *m*/*z* calcd. for $C_{30}H_{28}N$ 457.2266 $[M+H]^+$, found 457.2261.

2.3.2 2'-(1,1',3,3'-Tetramethyl-1,3-dihydro-1'*H*,3'*H*-spiro[benzo[*d*]imidazole-2,2'-perimidin]-5-yl)-[1,1'-biphenyl]-4-carbonitrile (**1-*ms*-BN**)

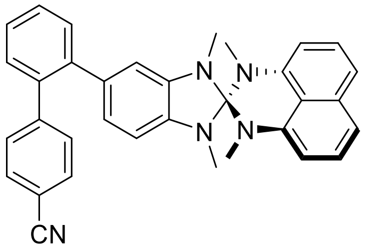

Spiro-bromide **8a** (550 mg, 1.34 mmol), boronic ester **20** (548 mg, 1.80 mmol, 1.3 eq.), $Pd(PPh_3)_4$ (80 mg, 69 µmol, 5 mol%) and Na_2CO_3 (757 mg, 7.14 mmol, 6.0 eq.) were combined in a glovebox, dissolved in a degassed mixture of THF (40 mL) and H_2O (20 mL) and refluxed under argon for 4 d. CH_2Cl_2 (20 mL) was added, the aqueous phase was separated, further extracted with CH_2Cl_2 (3 × 20 mL), and the combined organic extracts were dried ($MgSO_4$). The solvent was removed under reduced pressure and the residue was purified by flash column chromatography (SiO_2, cyclohexane/Et_2O: 20/1 to 15/1). Sublimation at 250 °C ($p < 10^{-3}$ mbar) afforded **1-*ms*-BN** (401 mg, 791 µmol, 59%) as a yellow solid.

***R*f** 0.47 (cyclohexane/EtOAc: 10/1); **^{1}H NMR** (500 MHz, CD_2Cl_2) δ = 7.54–7.48 (m, 3H), 7.47–7.44 (m, 1H), 7.41–7.40 (m, 2H), 7.38–7.30 (m, 4H), 7.09 (dd, *J* = 8.3, 0.9 Hz, 2H), 6.44 (dd, *J* = 7.6, 1.7 Hz, 1H), 6.41 (dd, *J* = 7.8, 0.9 Hz, 2H), 6.28 (d, *J* = 7.6 Hz, 1H), 5.94 (d, *J* = 1.7 Hz, 1H), 2.69 (s, 3H), 2.61 (s, 6H), 2.49 (s, 3H) ppm; **^{13}C NMR** (126 MHz, CD_2Cl_2) δ = 147.9, 142.5, 141.3, 139.0, 136.5, 136.0, 134.4, 131.8, 130.9, 130.7, 130.4, 130.1, 128.8, 128.0, 127.1, 119.6, 119.4, 116.1, 111.0, 110.1, 106.4, 103.9, 101.1, 100.8, 31.0, 27.3, 27.0 ppm; **HRMS** (pos. ESI): *m/z* calcd. for $C_{38}H_{35}N_5$ 508.2501 $[M+H]^+$, found 508.2891.

2.3.3 2-Phenyl-5-(2'-(1,1',3,3'-tetramethyl-1,3-dihydro-1'*H*,3'*H*-spiro[benzo[*d*]imidazole-2,2'-perimidin]-5-yl)-[1,1'-biphenyl]-4-yl)-1,3,4-oxadiazole (**1-*ms*-DPOD**)

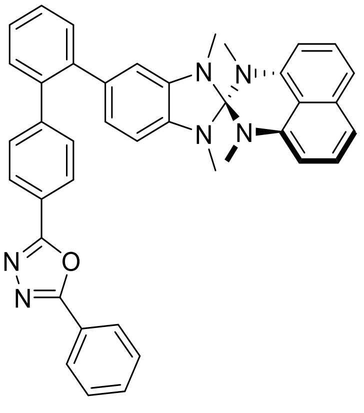

Spiro-bromide **8a** (500 mg, 1.22 mmol), boronic ester **14** (531 mg, 1.25 mmol, 1.0 eq.), $Pd(PPh_3)_4$ (43 mg, 37 µmol, 4 mol%) and Na_2CO_3 (8.63 g, 8.15 mmol, 6.7 eq.) were combined in a glovebox, dissolved in a degassed mixture of THF (40 mL) and H_2O (20 mL) and refluxed under argon for 48 h. H_2O (5 mL) and CH_2Cl_2 (10 mL) were added, the aqueous phase was separated, further extracted with CH_2Cl_2 (3 × 10 mL), and the combined organic extracts were dried ($MgSO_4$). The solvent was removed under reduced pressure and the residue was purified by flash column chromatography (SiO_2, cyclohexane/EtOAc: 10/1). Sublimation at 260 °C ($p < 10^{-3}$ mbar) afforded **1-*ms*-DPOD** (535 mg, 854 µmol, 70%) as a yellow solid.

***R*f** 0.27 (cyclohexane/EtOAc: 10/1); **^{1}H NMR** (500 MHz, CD_2Cl_2) δ = 8.19–8.10 (m, 3H), 8.00–7.97 (m, 1H), 7.61–7.55 (m, 4H), 7.53 (dd, *J* = 7.6, 1.3 Hz, 1H), 7.49–7.43 (m, 2H), 7.43–7.41 (m, 1H), (dd, *J* = 8.0 Hz, 1H), 7.31–7.27 (m, 2H), 7.08 (dd, *J* = 8.3, 0.7 Hz, 1H), 7.05 (dd, *J* = 8.3, 0.7 Hz, 1H), 6.55 (d, *J* = 1.7 Hz, 1H), 6.43 (d, *J* = 7.5 Hz, 1H), 6.39 (d, *J* = 7.4 Hz, 1H), 6.30 (d, *J* = 7.6 Hz, 1H), 6.05 (d, *J* = 1.6 Hz, 1H), 2.68 (s, 3H), 2.61 (s, 6H), 2.45 (s, 3H) ppm; **^{13}C NMR** (126 MHz, $CDCl_3$) δ = 146.7, 144.2, 143.3, 144.5, 141.8, 141.3, 142.6, 140.5, 139.9, 138.4, 136.1, 135.0, 134.7, 134.7, 134.0, 133.5, 132.8, 132.4, 132.0, 131.1, 126.5, 128.5, 126.0, 119.6, 120.0, 106.4, 104.1, 101.1, 100.8, 31.3, 31.1, 31.0 ppm; **HRMS** (pos. ESI): *m/z* calcd. for $C_{41}H_{35}N_6O$ 627.2867 $[M+H]^+$, found 627.2861.

2.3.4 GP2 - general procedure for the preparation of donor-acceptor scaffolds via SUZUKI-MIYAURA-coupling

The respective aryl halide, aryl boronic ester, $Pd(OAc)_2$, SPhos and K_3PO_4 were combined in a glovebox, dissolved in a degassed mixture of 1,4-dioxane and H_2O (2/1) and stirred under argon at 100 °C. After consumption of the starting material, the reaction mixture was allowed to cool to rt, diluted with CH_2Cl_2, and the aqueous layer was separated. The organic phase was filtered over a pad of silica, and the solvents were removed under reduced pressure. Further purification is stated in the respective procedure.

2.3.5 3-(2-(1,1',3,3'-Tetramethyl-1,3-dihydro-1'*H*,3'*H*-spiro[benzo[*d*]imidazole-2,2'-perimidin]-5-yl)phenyl)dibenzo[*b*,*d*]thiophene 5,5-dioxide (**1-*ms*-DBTO**)

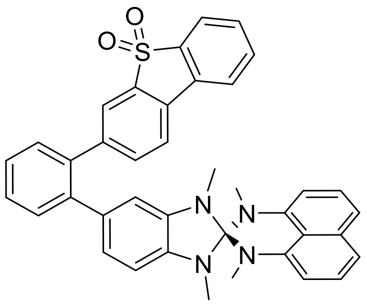

1-*ms*-DBTO was synthesized following **GP2**, using spiro-chloride **12** (100 mg, 227 µmol), **DBTO-Bpin** (93.0 mg, 272 µmol, 1.2 eq.), $Pd(OAc)_2$ (2.5 mg, 11.4 µmol, 5 mol%), SPhos (9.0 mg, 22.7 mmol, 10 mol%) and K_3PO_4 (289 mg, 1.36 mmol, 6.0 eq.) in degassed 1,4-dioxane/H_2O (7.5 mL). After 26 h, the reaction was worked up and the crude material was purified by recrystallization from MeOH/$CHCl_3$ (125 mL, 1/1) and subsequent flash column chromatography SiO_2, cyclohexane/CH_2Cl_2: 1/1) to yield the title compound **1-*ms*-DBTO** as a yellow solid (38 mg, 61 µmol, 27%).

***R*$_f$** (cyclohexane/CH_2Cl_2: 1/1): 0.38; **^{1}H NMR** (400 MHz, $CDCl_3$): δ = 7.81 (ddd, *J* = 7.7, 1.2, 0.7 Hz, 1H), 7.76 (ddd, *J* = 7.7, 0.9, 0.9 Hz, 1H), 7.70–7.66 (m, 1H), 7.64 (ddd, *J* = 7.6, 7.6, 1.2 Hz, 1H), 7.58–7.49 (m, 4H), 7.48–7.37 (m, 3H), 7.29 (dd, *J* = 8.2, 7.7 Hz, 2H), 7.09 (dd, *J* = 8.3, 0.9 Hz, 2H), (dd, *J* = 7.7, 1.7 Hz, 1H), 6.36 (dd, *J* = 7.7, 0.9 Hz, 2H), 6.24–6.08 (m, 2H), 2.69 (s, 3H), 2.63 (s, 6H), 2.56 (s, 3H) ppm; **^{13}C NMR** (101 MHz, $CDCl_3$): δ = 145.6, 142.3, 141.0, 138.3, 138.1, 137.6, 135.1, 134.2, 133.9, 130.9, 130.2, 130.1, 129.8, 129.3, 128.6, 127.8, 127.1, 123.8,

122.3, 121.5, 121.0, 119.7, 116.0, 101.2, 100.6, 31.1, 27.2, 27.1 ppm (two signals are overlapping/not visible); **HRMS** (pos. ESI): *m*/*z* calcd. for $C_{39}H_{33}N_4O_2S$ $[M+H]^+$ 621.2319, found 621.2306.

2.3.6 1,1',3,3'-Tetramethyl-5-(4'-(phenylsulfonyl)-[1,1'-biphenyl]-2-yl)-1,3-dihydro-1'*H*,3'*H*-spiro[benzo[*d*]imidazole-2,2'-perimidine] (**1-*ms*-DPS**)

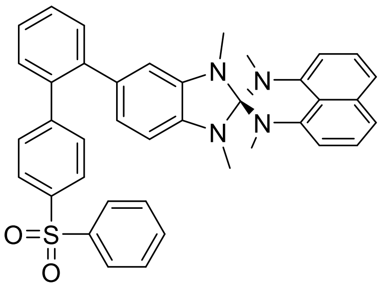

1-*ms*-DPS was synthesized following **GP2**, using spiro-chloride **12** (150 mg, 340 µmol), **DPS-Bpin** (140 mg, 408 µmol, 1.2 eq.), $Pd(OAc)_2$ (3.8 mg, 16.9 µmol, 5 mol%), SPhos (13.9 mg, 33.9 µmol, 10 mol%) and K_3PO_4 (433 mg, 2.04 mmol, 6.0 eq.) in degassed 1,4-dioxane/H_2O (11.4 mL). After 23 h, the reaction was worked up and the crude material was purified by flash column chromatography (silica gel, cyclohexane/CH_2Cl_2: 1/3) to afford the title compound **1-*ms*-DPS** as a yellow solid (206 mg, 330 µmol, 97%).

***R*$_f$** (cyclohexane/CH_2Cl_2: 1/3): 0.33; **^{1}H NMR** (500 MHz, CD_2Cl_2): δ = 7.96–7.90 (m, 3H), 7.81–7.76 (m, 2H), 7.61–7.31 (m, 10H), 7.11 (dd, *J* = 8.3, 0.9 Hz, 2H), 6.52 (dd, *J* = 7.6, 1.7 Hz, 1H), 6.41 (dd, *J* = 7.9, 0.9 Hz, 2H), 6.26 (d, *J* = 7.6 Hz, 1H), 5.79 (d, *J* = 1.7 Hz, 1H), 2.68 (s, 3H), 2.55 (s, 6H), 2.24 (s, 3H) ppm; **^{13}C NMR** (126 MHz, CD_2Cl_2): δ = 148.4, 142.4, 142.1, 141.3, 139.6, 138.7, 136.4, 136.0, 134.4, 133.61, 133.58, 131.2, 130.7, 130.6, 123.0, 129.7, 128.8, 128.1, 128.0, 127.8, 127.4, 127.1, 119.6, 116.2, 111.0, 106.4, 104.1, 101.2, 100.9, 31.0, 27.3, 26.9 ppm (two signals are overlapping/not visible); **HRMS** (pos. ESI): *m*/*z* calcd. for $C_{39}H_{35}N_4O_2S$ $[M+H]^+$ 623.2476, found 623.2475.

2.3.7 2-(2-(1,1',3,3'-Tetramethyl-1,3-dihydro-1'*H*,3'*H*-spiro[benzo[*d*]imidazole-2,2'-perimidin]-5-yl)phenyl)thianthrene 5,5,10,10-tetraoxide (**1-*ms*-TTO**)

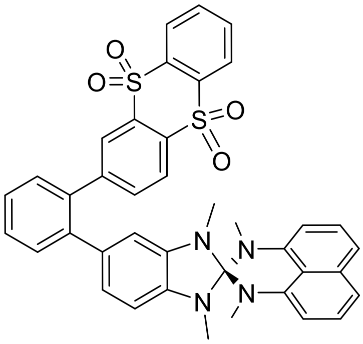

1-*ms*-TTO was synthesized following **GP2**, using spiro-chloride **12** (150 mg, 340 µmol), aryl boronic ester **TTO-Bpin** (166 mg, 408 µmol, 1.2 eq.), $Pd(OAc)_2$ (3.8 mg, 16.9 µmol, 5 mol%), SPhos (13.9 mg, 33.9 µmol, 10 mol%) and K_3PO_4 (433 mg, 2.04 mmol, 6.0 eq.) in degassed 1,4-dioxane/H_2O (11.4 mL). After 21 h, the reaction was worked up and the crude material was purified by flash column chromatography (SiO_2, cyclohexane/CH_2Cl_2: 1/2) to afford the title compound **1-*ms*-TTO** as an orange solid (169 mg, 247 µmol, 73%).

$\boldsymbol{R_f}$ (cyclohexane/CH_2Cl_2: 1/1): 0.57; **^{1}H NMR** (500 MHz, CD_2Cl_2): δ = 8.27–8.21 (m, 1H), 8.20–8.16 (m, 1H), 8.09 (d, J = 8.1 Hz, 1H), 8.04 (d, J = 1.7 Hz, 1H), 7.85–7.80 (m, 2H), 7.69 (dd, J = 8.1, 1.7 Hz, 1H), 7.58–7.54 (m, 1H), 7.54–7.48 (m, 1H), 7.47–7.41 (m, 2H), 7.31 (dd, J = 8.2, 7.7 Hz, 2H), 7.09 (dd, J = 8.2, 0.9 Hz, 2H), 6.39 (dd, J = 7.7, 0.9 Hz, 2H), 6.36 (dd, J = 7.6, 1.7 Hz, 1H), 6.23 (d, J = 7.6 Hz, 1H), 6.05 (d, J = 1.7 Hz, 1H), 2.68 (s, 3H), 2.61 (s, 6H), 2.50 (s, 3H) ppm; **^{13}C NMR** (126 MHz, CD_2Cl_2): δ = 149.5, 143.0, 141.3, 140.04, 140.02, 139.0, 137.1, 137.0, 136.7, 136.3, 134.7, 134.7, 134.4, 134.2, 134.1, 131.1, 130.3, 129.7, 129.2, 128.1, 127.7, 127.5, 126.1, 126.0, 120.1, 116.1, 111.0, 106.4, 103.5, 101.3, 100.9, 31.2, 27.3, 27.1 ppm; **HRMS** (pos. ESI): m/z calcd. for $C_{39}H_{33}N_4O_4S_2$ $[M+H]^+$ 685.1938, found 685.1920.

2.3.8 3-(1,1',3,3'-Tetramethyl-1,3-dihydro-1'*H*,3'*H*-spiro[benzo[*d*]imidazole-2,2'-perimidin]-5-yl)dibenzo[*b*,*d*]thiophene 5,5-dioxide (**1-*m*-DBTO**)

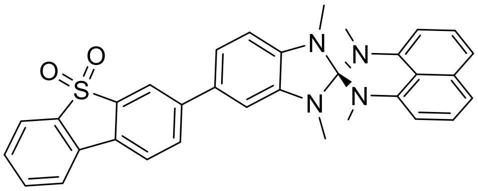

1-*m*-DBTO was synthesized following **GP2**, using spiro-bromide **8a** (100 mg, 244 µmol), **DBTO-Bpin** (100 mg, 293 µmol, 1.2 eq.), $Pd(OAc)_2$ (2.7 mg, 12.0 µmol, 5 mol%), SPhos (9.9 mg, 24.1 µmol, 10 mol%) and K_3PO_4 (311 mg, 1.46 mmol, 6.0 eq.) in degassed 1,4-dioxane/H_2O (8.0 mL). After 20 h, the reaction mixture was worked up and the crude material was purified by recrystallization from MeOH/$CHCl_3$ (125 mL) to yield the title compound **1-*m*-DBTO** as a yellow solid (135 mg, 240 µmol, 99%).

$\boldsymbol{R_f}$ (cyclohexane/CH_2Cl_2: 1/1): 0.36; **^{1}H NMR** (400 MHz, C_6D_6): δ = 8.15 (dd, *J* = 1.8, 0.5 Hz, 1H), 7.54 (dd, *J* = 8.1, 1.8 Hz, 1H), 7.49 (ddd, *J* = 7.6, 1.2, 1.2 Hz, 1H), 7.34 (dd, *J* = 8.2, 7.6 Hz, 2H), 7.27 (dd, *J* = 8.3, 1.0 Hz, 2H), 7.15–7.12 (m, 1H), 7.04 (ddd, *J* = 7.7, 1.0, 1.0 Hz, 1H), 6.91 (ddd, *J* = 7.6, 7.6, 1.2 Hz, 1H), 6.85 (dd, *J* = 7.7, 1.8 Hz, 1H), 6.75 (ddd, *J* = 7.6, 7.6, 1.0 Hz, 1H), 6.35 (d, *J* = 1.8 Hz, 1H), 6.25 (dd, *J* = 7.6, 1.0 Hz, 2H), 6.16 (d, *J* = 7.7 Hz, 1H), 2.34 (s, 6H), 2.29 (s, 3H), 2.27 (s, 3H) ppm; **^{13}C NMR** (101 MHz, C_6D_6): δ = 145.6, 141.1, 140.0, 139.2, 137.3, 137.2, 134.9, 133.1, 132.0, 131.2, 129.6, 129.0, 128.9, 122.3, 121.9, 121.2, 120.0, 117.6, 117.0, 111.4, 106.4, 101.7, 101.2, 100.2, 30.7, 26.8, 26.7 ppm (one signal is overlapping/not visible); **HRMS** (pos. ESI): *m/z* calcd. for $C_{33}H_{28}N_4O_2S$ $[M]^+$ 544.1927, found 544.1927.

2.3.9 1,1',3,3'-Tetramethyl-5-(4-(phenylsulfonyl)phenyl)-1,3-dihydro-1'*H*,3'*H*-spiro[benzo[*d*]- imidazole-2,2'-perimidine] (**1-*m*-DPS**)

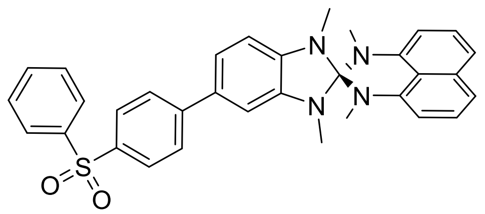

1-*m*-DPS was synthesized following **GP2**, using spiro-bromide **8a** (150 mg, 366 µmol), **DPS-Bpin** (154 mg, 447 µmol, 1.2 eq.), $Pd(OAc)_2$ (4.0 mg, 17.8 µmol, 5 mol%), SPhos (15.2 mg, 37.0 µmol, 10 mol%) and K_3PO_4 (458 mg, 2.20 mmol, 6.0 eq.) in degassed 1,4-dioxane/H_2O (12.2 mL). After 23 h, the reaction mixture was worked up and the crude material was purified by flash column chromatography (SiO_2, cyclohexane/CH_2Cl_2: 1/3) to yield the title compound **1-*m*-DPS** as a yellow solid (179 mg, 327 µmol, 90%).

***R*f** (cyclohexane/CH_2Cl_2: 1/3): 0.23; **^{1}H NMR** (500 MHz, CD_2Cl_2): *δ* = 7.99–7.96 (m, 2H), 7.95–7.91 (m, 2H), 7.75–7.69 (m, 2H), 7.61–7.56 (m, 1H), 7.56–7.50 (m, 2H), 7.33 (dd, *J* = 7.9, 7.9 Hz, 2H), 7.12 (dd, *J* = 8.3, 0.8 Hz, 2H), 6.97 (dd, *J* = 7.9, 1.8 Hz, 1H), 6.60 (d, *J* = 1.8 Hz, 1H), 6.45–6.40 (m, 3H), 2.77 (s, 3H), 2.75 (s, 3H), 2.67 (s, 6H) ppm; **^{13}C NMR** (126 MHz, CD_2Cl_2): *δ* = 147.7, 142.0, 141.1, 138.6, 137.6, 137.5, 134.4, 133.4, 129.7, 128.5, 128.4, 128.1, 127.8, 127.1, 117.7, 116.4, 111.0, 106.5, 101.8, 101.0, 100.2, 31.1, 27.3, 27.3 ppm; **HRMS** (pos. ESI): *m*/*z* calcd. for $C_{33}H_{31}N_4O_2S$ $[M+H]^+$ 547.2162, found 547.2164.

2.3.10 2-(1,1',3,3'-Tetramethyl-1,3-dihydro-1'*H*,3'*H*-spiro[benzo[*d*]imidazole-2,2'-perimidin]-5-yl)thianthrene 5,5,10,10-tetraoxide (**1-*m*-TTO**)

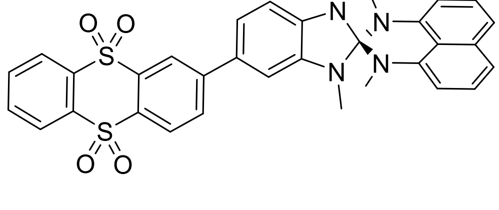

1-*m*-TTO was synthesized following **GP2**, using spiro-bromide **8a** (150 mg, 366 µmol), **TTO-Bpin** (182 mg, 447 µmol, 1.2 eq.), $Pd(OAc)_2$ (4.0 mg, 17.8 µmol, 5 mol%), SPhos (15.2 mg, 37.0 µmol, 10 mol%) and K_3PO_4 (458 mg, 2.20 mmol, 6.0 eq.) in degassed 1,4-dioxane/H_2O (12.2 mL). After 21 h, the reaction mixture was worked up and the crude material was purified by flash column chromatography (SiO_2, cyclohexane/CH_2Cl_2: 1/2) to yield the title compound **1-*m*-TTO** as a red solid (221 mg, 362 µmol, 99%).

***R*f** (cycohexane/CH_2Cl_2: 1/2): 0.38; **^{1}H NMR** (400 MHz, CD_2Cl_2): δ = 8.42 (d, J = 1.8 Hz, 1H), 8.30–8.22 (m, 2H), 8.19 (d, J = 8.2 Hz, 1H), 8.01–7.93 (m, 1H), 7.86–7.81 (m, 2H), 7.33 (dd, J = 7.9, 7.9 Hz, 2H), 7.13 (dd, J = 8.2, 0.8 Hz, 2H), 7.08 (dd, J = 7.7, 1.8 Hz, 1H), 6.67 (d, J = 1.8 Hz, 1H), 6.46 (dd, J = 7.7, 6.3 Hz, 3H), 2.81 (s, 3H), 2.77 (s, 3H), 2.68 (s, 6H) ppm; **^{13}C NMR** (101 MHz, CH_2Cl_2): δ = 148.7, 141.1, 140.4, 140.1, 140.0, 139.9, 138.6, 137.8, 135.5, 134.5, 134.3, 134.2, 134.0, 130.3, 128.1, 126.9, 126.6, 126.3, 126.2, 126.0, 118.5, 116.5, 111.1, 106.6, 101.9, 101.2, 99.9, 31.1, 27.4 ppm (one signal is overlapping/not visible); **HRMS** (pos. ESI): m/z calcd. for $C_{33}H_{29}N_4O_4S_2$ $[M+H]^+$ 609.1625, found 609.1615.

2.3.11 3-(1,1',3,3'-Tetramethyl-1,3-dihydro-1'*H*,3'*H*-spiro[benzo[*d*]imidazole-2,2'-perimidin]-4-yl)dibenzo[*b*,*d*]thiophene 5,5-dioxide (**1-*o*-DBTO**)

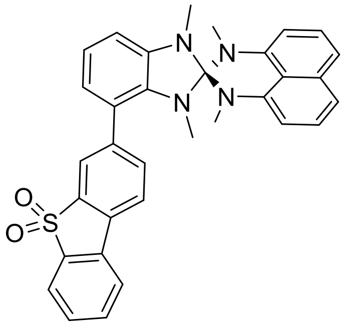

1-*o*-DBTO was synthesized following **GP2**, using spiro-bromide **8b** (150 mg, 411 µmol), **DBTO-Bpin** (169 mg, 493 µmol, 1.2 eq.), $Pd(OAc)_2$ (4.6 mg, 20.5 µmol, 5 mol%), SPhos (17.3 mg, 42.1 mmol, 10 mol%) and K_3PO_4 (523 mg, 2.47 mmol, 6.0 eq.) in degassed 1,4-dioxane/H_2O (13.7 mL). After 19 h, the reaction mixture was worked up and the crude material was purified

by flash column chromatography (SiO_2, cyclohexane/CH_2Cl_2: 1/1) to yield the title compound **1-*o*-DBTO** as a yellow solid (198 mg, 364 µmol, 89%).

***R*f** (cyclohexane/ CH_2Cl_2: 1/1): 0.27; **^{1}H NMR** (400 MHz, CD_2Cl_2): δ = 7.86–7.79 (m, 4H), 7.69–7.64 (m, 2H), 7.54 (ddd, *J* = 7.7, 7.7, 1.0 Hz, 1H), 7.31 (dd, *J* = 8.3, 7.7 Hz, 2H), 7.09 (dd, *J* = 8.3, 0.8 Hz, 2H), 6.75 (dd, *J* = 8.0, 7.5 Hz, 1H), 6.56 (dd, *J* = 8.0, 1.2 Hz, 1H), 6.43 (dd, *J* = 7.8, 1.2 Hz, 1H), 6.42 (dd, *J* = 7.8, 0.9 Hz, 2H), 2.78 (s, 3H), 2.73 (s, 6H), 2.38 (s, 3H) ppm; **^{13}C NMR** (101 MHz, CD_2Cl_2): δ = 143.5, 141.2, 138.4, 138.0, 137.8, 135.8, 134.5, 134.4, 133.4, 132.0, 130.6, 130.2, 128.1, 123.1, 122.3, 122.1, 121.3, 120.4, 118.2, 116.9, 116.2, 110.9, 106.9, 101.6, 100.8, 31.2, 31.1, 27.4 ppm; **HRMS** (pos. ESI): *m/z* calcd. for $C_{33}H_{29}N_4O_2S$ $[M+H]^+$ 545.2006, found 545.2007.

2.3.12 1,1',3,3'-Tetramethyl-4-(4-(phenylsulfonyl)phenyl)-1,3-dihydro-1'*H*,3'*H*-spiro[benzo-[*d*]imidazole-2,2'-perimidine] (**1-*o*-DPS**)

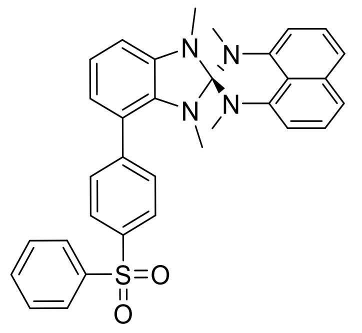

1-*o*-DPS was synthesized following **GP2**, using spiro-bromide **8b** (150 mg, 411 µmol), **DPS-Bpin** (170 mg, 493 µmol, 1.2 eq.), $Pd(OAc)_2$ (4.6 mg, 20.5 µmol, 5 mol%), SPhos (17.3 mg, 42.1 µmol, 10 mol%) and K_3PO_4 (523 mg, 2.47 mmol, 6.0 eq.) in degassed 1,4-dioxane/H_2O (13.7 mL). After 19 h, the reaction mixture was worked up and the crude material was purified by flash column chromatography (SiO_2, cyclohexane/CH_2Cl_2: 1/1) to yield the title compound **1-*o*-DPS** as a yellow solid (176 mg, 321 µmol, 78%).

***R*f** (cyclohexane/CH_2Cl_2: 1/1): 0.36; **^{1}H NMR** (500 MHz, CD_2Cl_2): δ = 7.96–7.89 (m, 4H), 7.60–7.49 (m, 5H), 7.30 (dd, *J* = 7.9, 7.9 Hz, 2H), 7.08 (dd, *J* = 8.3, 0.8 Hz, 2H), 6.72–6.67 (m, 1H), 6.45 (dd, *J* = 7.9, 1.2 Hz, 1H), 6.41–6.38 (m, 3H), 2.76 (s, 3H), 2.69 (s, 6H), 2.25 (s, 3H) ppm; **^{13}C NMR**

(101 MHz, CD_2Cl_2): δ = 146.1, 142.2, 141.2, 140.1, 137.6, 134.4, 133.6, 133.3, 131.0, 129.7, 128.1, 127.9, 127.4, 120.3, 118.0, 117.2, 116.2, 110.9, 106.8, 101.5, 100.8, 31.1, 31.0, 27.3 ppm; **HRMS** (pos. ESI): *m/z* calcd. for $C_{33}H_{31}N_4O_2S$ $[M+H]^+$ 547.2162, found 547.2158.

2.3.13 2-(1,1',3,3'-Tetramethyl-1,3-dihydro-1'*H*,3'*H*-spiro[benzo[*d*]imidazole-2,2'-perimidin]-4-yl)thianthrene 5,5,10,10-tetraoxide (**1-*o*-TTO**)

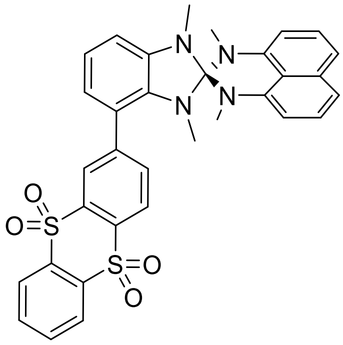

1-*o*-TTO was synthesized following **GP2**, using spiro-bromide **8b** (100 mg, 274 µmol), **TTO-Bpin** (134 mg, 329 µmol, 1.2 eq.), $Pd(OAc)_2$ (3.1 mg, 13.7 µmol, 5 mol%), SPhos (11.3 mg, 27.4 µmol, 10 mol%) and K_3PO_4 (349 mg, 1.64 mmol, 6.0 eq.) in degassed 1,4-dioxane/H_2O (9.1 mL,). After 19 h, the reaction mixture was worked up and the crude material was purified by flash column chromatography (SiO_2, cyclohexane/EtOAc: 3/1) to yield the title compound **1-*o*-TTO** as a red solid (142 mg, 232 µmol, 85%).

***R*$_f$** (cyclohexane/EtOAc: 3/1): 0.24; **^{1}H NMR** (400 MHz, CD_2Cl_2): δ = 8.27–8.20 (m, 4H), 7.87–7.78 (m, 3H), 7.31 (dd, *J* = 8.3, 7.7 Hz, 2H), 7.10 (dd, *J* = 8.3, 0.8 Hz, 2H), 6.77 (dd, *J* = 8.0, 7.4 Hz, 1H), 6.52 (dd, *J* = 8.0, 1.1 Hz, 1H), 6.45 (dd, *J* = 7.5, 1.1 Hz, 1H), 6.42 (dd, *J* = 7.7, 0.8 Hz, 2H), 2.79 (s, 3H), 2.71 (s, 6H), 2.32 (s, 3H) ppm; **^{13}C NMR** (101 MHz, CD_2Cl_2): δ = 147.2, 141.0, 140.1, 139.9, 139.3, 137.9, 137.3, 134.7, 134.5, 134.3, 134.2, 133.7, 128.1, 127.0, 126.3, 126.2, 125.9, 120.1, 118.6, 116.4, 115.3, 110.9, 107.0, 102.1, 101.0, 31.6, 31.1, 27.4 ppm; **HRMS** (pos. ESI): *m/z* calcd. for $C_{33}H_{29}N_4O_4S_2$ $[M+H]^+$ 609.1625, found 609.1628.

3. Single Crystal X-ray Diffraction

X-ray data were collected from a shock-cooled single crystal at 100(2) K on a BRUKER APEX2 QUAZAR three-circle diffractometer with a microfocus sealed X-ray tube using mirror optics as monochromator and a BRUKER APEXII detector. The diffractometer was equipped with an OXFORD CRYOSTREAM 800 low temperature device and used MoK_α radiation (λ = 0.71073 Å). The data of **S3** was collected from a shock-cooled single crystal at 100(2) K on a BRUKER D8 VENTURE dual wavelength Mo/Cu three-circle diffractometer with a microfocus sealed X-ray tube using mirror optics as monochromator and a BRUKER PHOTON III detector. The diffractometer was equipped with an OXFORD CRYOSTREAM 800 low temperature device and used CuKα radiation (λ = 1.54184 Å). All data were integrated with SAINT[6] and a multi-scan absorption correction using SADABS[7] was applied. The structure were solved by direct methods using SHELXT[8] and refined by full-matrix least-squares methods against F^2 by SHELXL-2018/3[9] using ShelXle.[10] Disorder was modelled using DSR.[11,12] All non-hydrogen atoms were refined with anisotropic displacement parameters. The hydrogen atoms were refined isotropically on calculated positions using a riding model with their U_{iso} values constrained to 1.5 times the U_{eq} of their pivot atoms for terminal sp^3 carbon atoms and 1.2 times for all other carbon atoms. Crystallographic data for the structures reported in this paper have been deposited with the Cambridge Crystallographic Data Centre[13] and can be obtained free of charge from The Cambridge Crystallographic Data Centre via www.ccdc.cam.ac.uk/structures. This report and the CIF file were generated using FinalCif.[14] The BF4 anion in **3a** was positionally disordered. **1-o-TTO** contained disordered solvent molecules: *n*-pentane (75% occupancy) and CH_2Cl_2 (25% occupancy). H-atoms of the water molecules in the structure of **S3** were restrained using the DFIX command. One molecule was disordered and was refined using bond lengths restraints and displacement parameter restraints.

Compound	S1	1-ms-DPS	1-o-TTO	3a	7a•	14a	S3
CCDC number	2046064	1949447	1949585	1912248	1962741	2041007	2043983
Empirical formula	$C_7H_7BrN_2O_2$	$C_{39}H_{34}N_4O_2S$	$C_{36.99}H_{37.48}Cl_{0.50}N_4O_4S_2$	$C_9H_9BBrClF_4N_2$	$C_8H_6BrClN_2$	$C_{27}H_{27}BrN_4$	$C_{39}H_{40}N_6O_2$
Formula weight	231.06	622.76	684.08	347.35	245.51	487.43	624.77
Temperature [K]	100(2)	100(2)	100(2)	100.01	100(2)	100(2)	100(2)
Crystal system	orthorhombic	monoclinic	monoclinic	monoclinic	monoclinic	monoclinic	triclinic
Space group (number)	$Pna2_1$ (33)	$P2_1/c$ (14)	$P2_1/c$ (14)	$P2_1/c$ (14)	$P2_1/c$ (14)	$P2_1/c$ (14)	$P\overline{1}$ (2)
a [Å]	7.5046(19)	17.886(10)	16.409(8)	8.031(7)	10.8538(9)	8.953(3)	7.3294(9)
b [Å]	15.780(4)	12.394(6)	8.092(4)	14.555(13)	6.9764(5)	12.097(4)	15.4129(14)
c [Å]	6.9161(17)	15.576(8)	25.544(13)	10.890(10)	11.9935(10)	20.596(7)	16.151(2)
α [Å]	90	90	90	90	90	90	115.740(6)
β [Å]	90	115.039(8)	97.67(2)	103.934(15)	110.192(5)	93.975(6)	94.786(12)
γ [Å]	90	90	90	90	90	90	98.461(9)
Volume [Å3]	819.0(4)	3128(3)	3361(3)	1235(2)	852.34(12)	2225.3(12)	1603.2(3)
Z	4	4	4	4	4	4	2
ρcalc [g/cm3]	1.874	1.322	1.352	1.868	1.913	1.455	1.294
μ [mm-1]	4.976	0.146	0.245	3.574	5.074	1.870	0.646
F(000)	456	1312	1440	680	480	1008	664
Crystal size [mm3]	0.180×0.100×0.050	0.150×0.100×0.080	0.250×0.100×0.060	0.26×0.18×0.08	0.17×0.13×0.05	0.30×0.10×0.03	0.140×0.060×0.030
Crystal colour	red	yellow	orange	colourless	colourless	colourless	yellow
Crystal shape	plate	block	needle	block	block	block	block
Radiation	MoK_α (λ=0.71073 Å)	MoK_α (λ=0.71073 Å)	MoK_α (λ=0.71073 Å)	MoK_α (λ=0.71073 Å)	MoK_α (λ=0.71073 Å)	MoK_α (λ=0.71073 Å)	CuK_α (λ=1.54184 Å)
2Θ range [°]	5.16 to 60.08 (0.71 Å)	2.51 to 51.85 (0.81 Å)	2.79 to 58.44 (0.73 Å)	4.76 to 57.52 (0.74 Å)	4.00 to 50.24 (0.84 Å)	3.91 to 60.29 (0.71 Å)	6.16 to 133.78 (0.84 Å)
Index ranges	−10 ≤ h ≤ 10	−21 ≤ h ≤ 22	−22 ≤ h ≤ 22	−10 ≤ h ≤ 10	−12 ≤ h ≤ 12	−12 ≤ h ≤ 12	−8 ≤ h ≤ 8
	−22 ≤ k ≤ 22	−15 ≤ k ≤ 15	−11 ≤ k ≤ 11	−19 ≤ k ≤ 19	−8 ≤ k ≤ 8	−16 ≤ k ≤ 16	−18 ≤ k ≤ 18
	−9 ≤ l ≤ 9	−19 ≤ l ≤ 18	−35 ≤ l ≤ 35	−14 ≤ l ≤ 14	−14 ≤ l ≤ 14	−28 ≤ l ≤ 28	−19 ≤ l ≤ 19
Reflections collected	25223	58592	117618	32075	16213	57570	17351
Independent	2393	6031	9108	3193	1519	6279	5550
reflections	R_{int} = 0.0257	R_{int} = 0.0597	R_{int} = 0.0697	R_{int} = 0.0367	R_{int} = 0.0372	R_{int} = 0.0777	R_{int} = 0.0227
	R_{sigma} = 0.0202	R_{sigma} = 0.0324	R_{sigma} = 0.0326	R_{sigma} = 0.0195	R_{sigma} = 0.0180	R_{sigma} = 0.0511	R_{sigma} = 0.0225
Completeness to θ = 25.242°	100.0 %	100.0 %	100.0 %	100.0 %	99.7 %[1]	100.0 %	97.3 %[2]
Data / Restraints / Parameters	2393/2/113	6031/0/419	9108/111/519	3193/262/211	1519/0/110	6279/0/293	5550/277/575
Goodness-of-fit on F2	1.088	1.031	1.031	1.030	0.878	0.846	1.024
Final R indexes	R_1 = 0.0153	R_1 = 0.0502	R_1 = 0.0464	R_1 = 0.0401	R_1 = 0.0222	R_1 = 0.0364	R_1 = 0.0320
[I≥2σ(I)]	wR_2 = 0.0354	wR_2 = 0.1156	wR_2 = 0.1143	wR_2 = 0.0932	wR_2 = 0.0917	wR_2 = 0.1039	wR_2 = 0.0836
Final R indexes	R_1 = 0.0164	R_1 = 0.0804	R_1 = 0.0625	R_1 = 0.0482	R_1 = 0.0276	R_1 = 0.0633	R_1 = 0.0375
[all data]	wR_2 = 0.0356	wR_2 = 0.1317	wR_2 = 0.1247	wR_2 = 0.0969	wR_2 = 0.1005	wR_2 = 0.1229	wR_2 = 0.0880
Largest peak/hole [eÅ3]	0.27/−0.29	0.16/−0.29	0.82/−0.54	1.72/−1.43	0.39/−0.42	0.62/−0.64	0.19/−0.16
Extinction coefficient							0.0017(3)
Flack X parameter	0.020(3)						

[1]Completeness to θ = 25.119°. [2]Completeness to θ = 66.888°

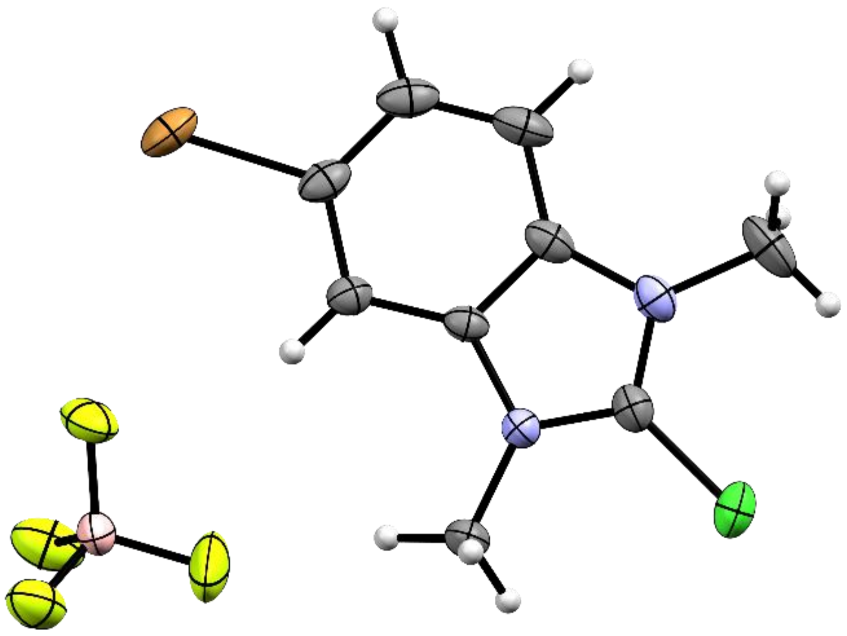

Figure S1: Crystal structure of **3a**, thermal ellipsoids at 50% probability, disorder is not shown.

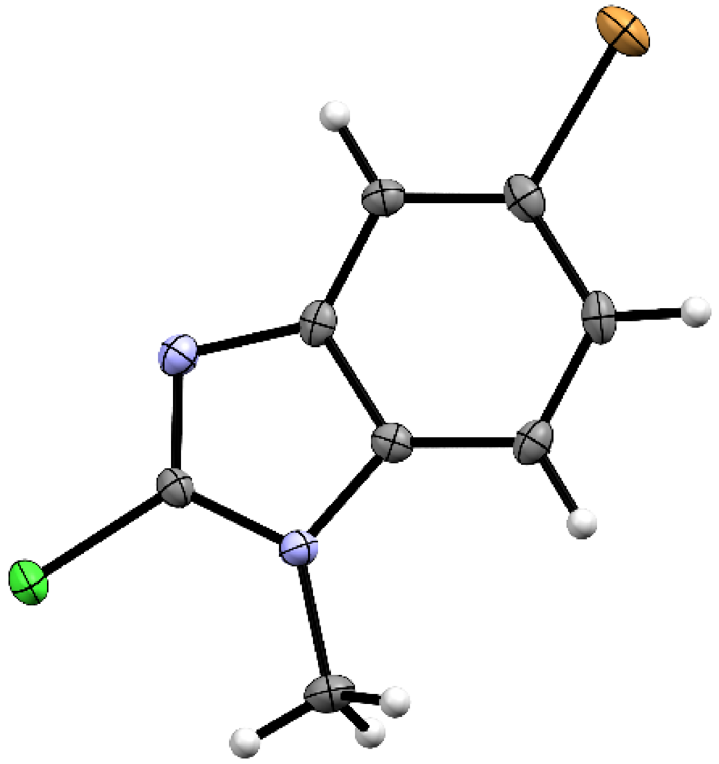

Figure S2: Crystal structure of **7a**, thermal ellipsoids at 50% probability, disorder is not shown.

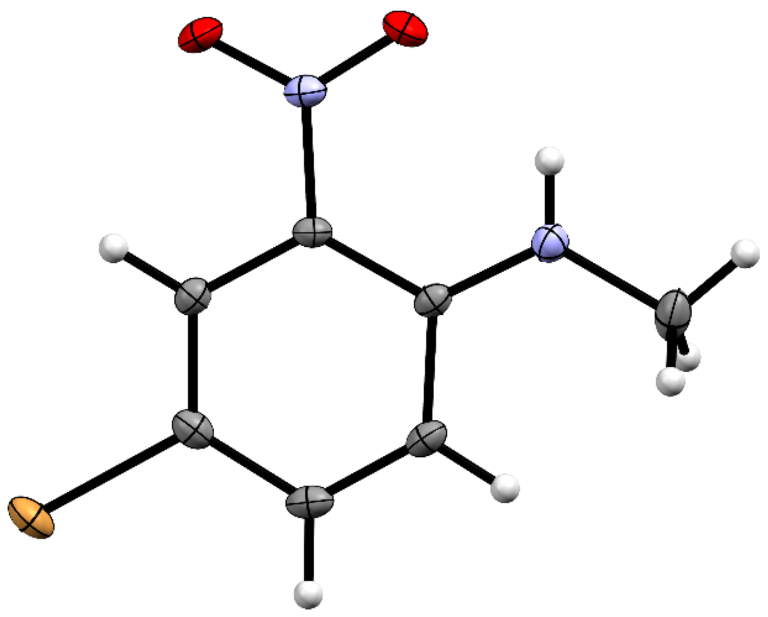

Figure S3: Crystal structure of **4a**, thermal ellipsoids at 50% probability, disorder is not shown.

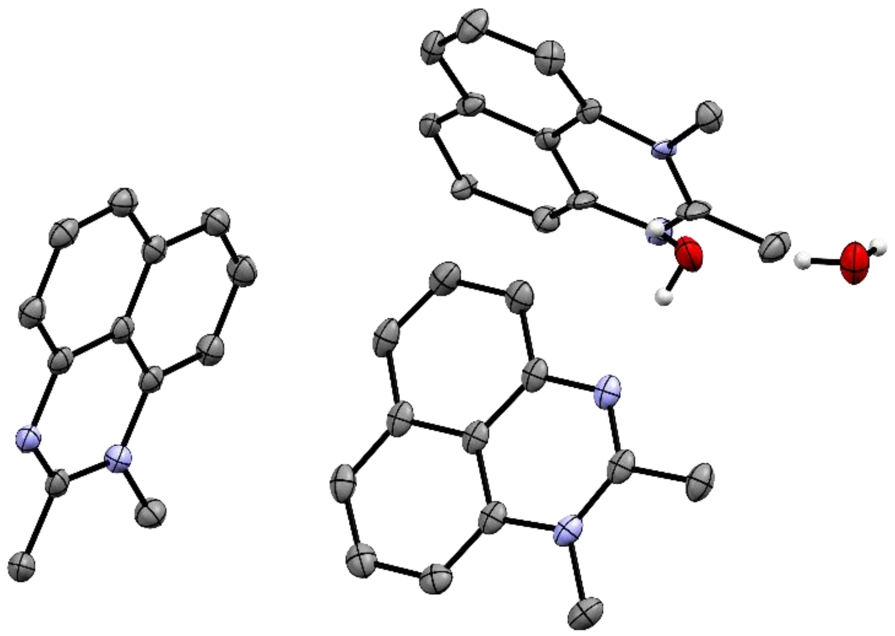

Figure S4: Crystal structure of **S3**, thermal ellipsoids at 50% probability, disorder is not shown.

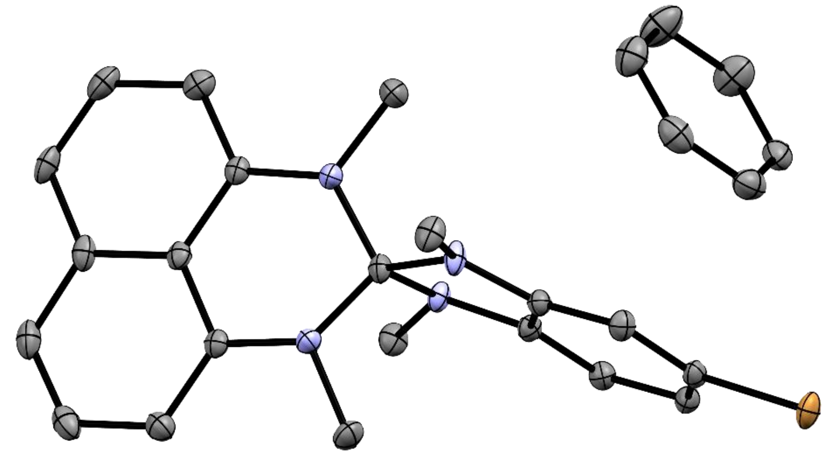

Figure S5: Crystal structure of **8a**, thermal ellipsoids at 50% probability, disorder is not shown.

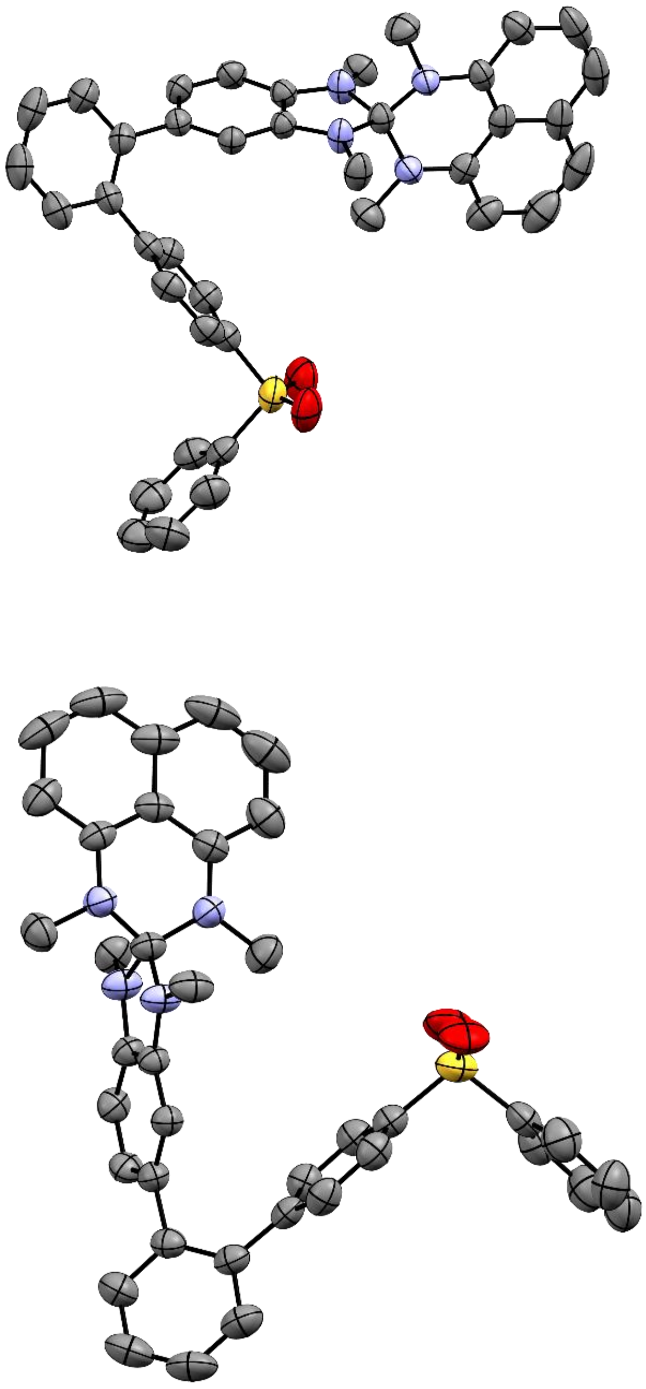

Figure S6: Crystal structure of **1-*ms*-DPS**, thermal ellipsoids at 50% probability, disorder is not shown.

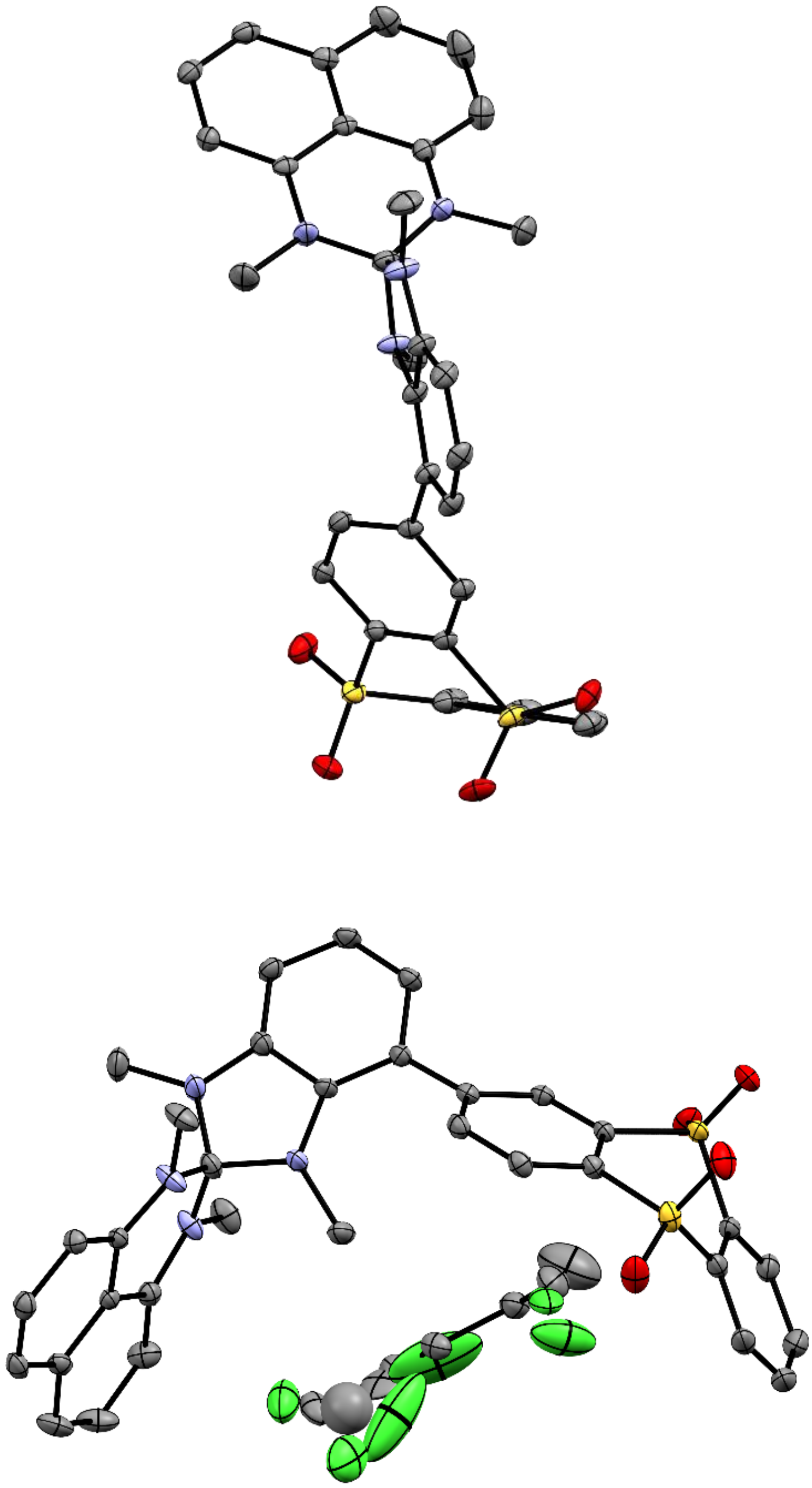

Figure S7: Crystal structure of **1-*m*-TTO**, thermal ellipsoids at 50% probability, disorder is not shown.

4. Optical Properties

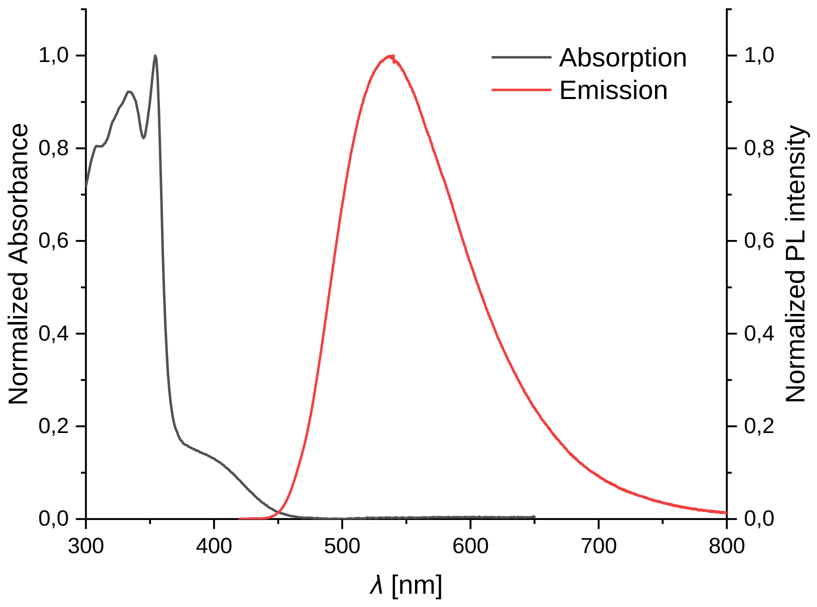

Figure S8: Normalized absorption and emission at 300 K of **1-o-DBTO** in toluene.

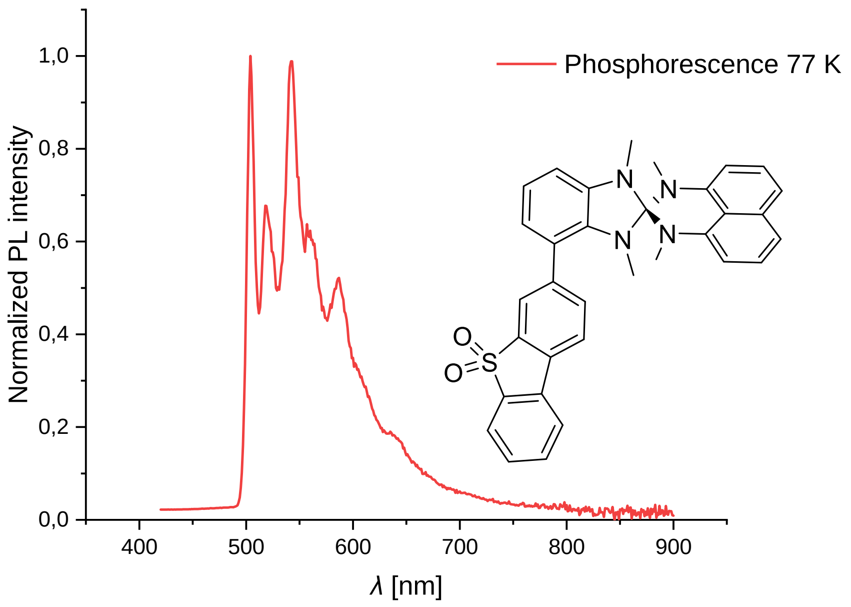

Figure S9: Normalized phosphorescence of **1-o-DBTO** at 77 K in toluene.

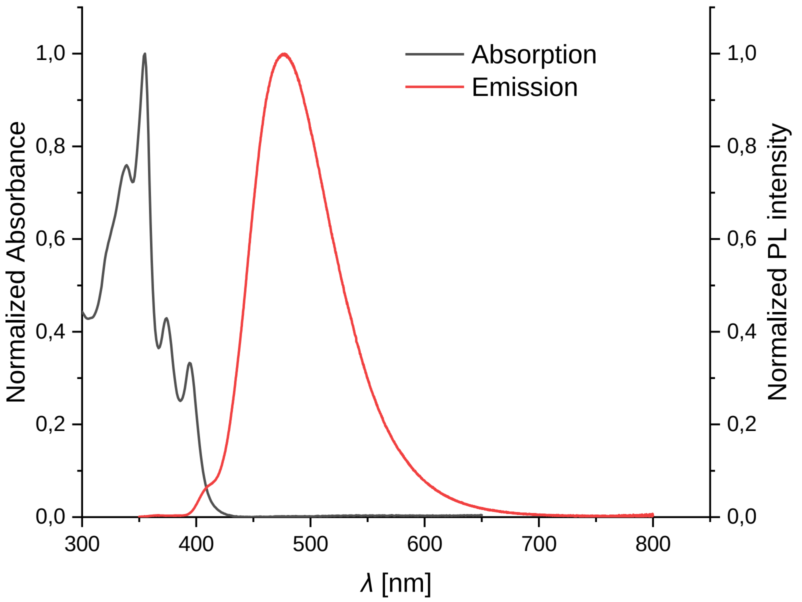

Figure S10: Normalized absorption and emission at 300 K of **1-o-DPS** in toluene.

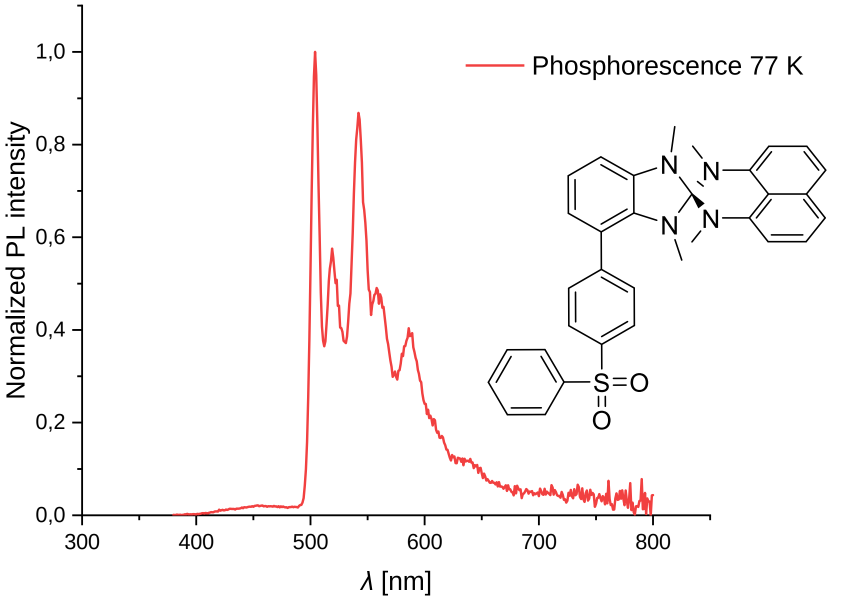

Figure S11: Normalized phosphorescence of **1-o-DPS** at 77 K in toluene.

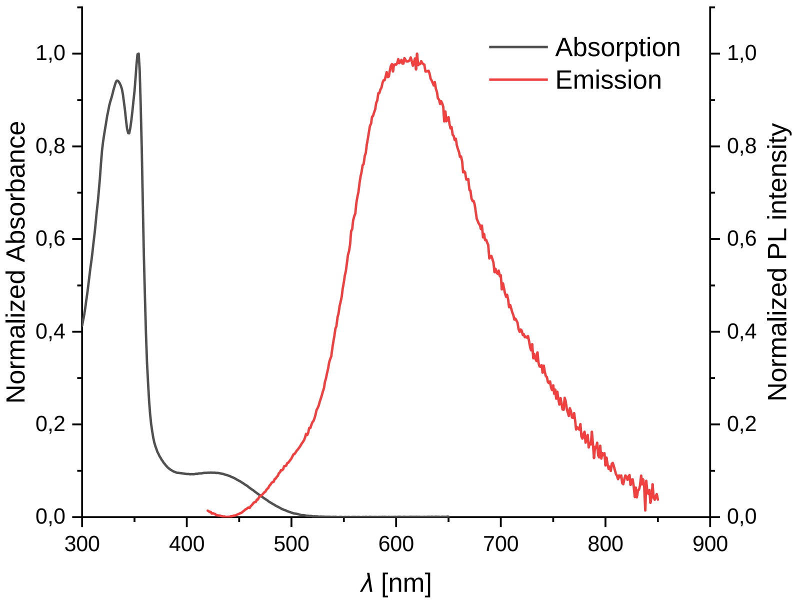

Figure S12: Normalized absorption and emission at 300 K of **1-o-TTO** in toluene.

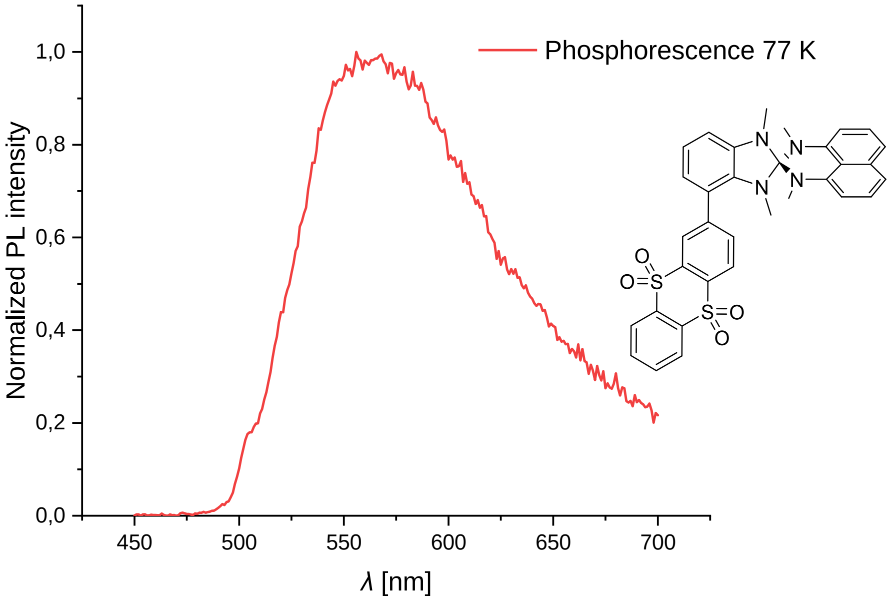

Figure S13: Normalized phosphorescence of **1-o-TTO** at 77 K in toluene.

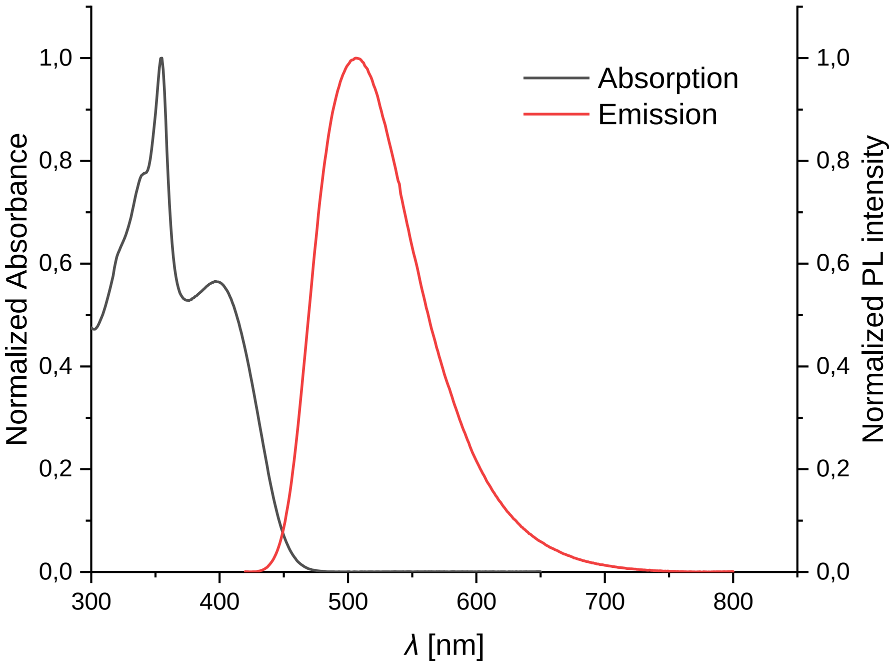

Figure S14: Normalized absorption and emission at 300 K of **1-m-DBTO** in toluene.

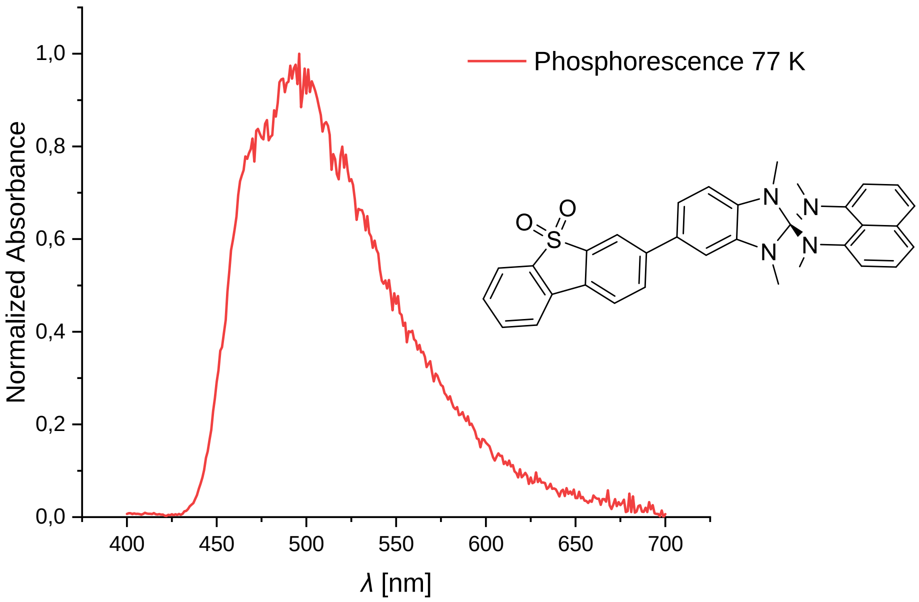

Figure S15: Normalized phosphorescence of **1-m-DBTO** at 77 K in toluene.

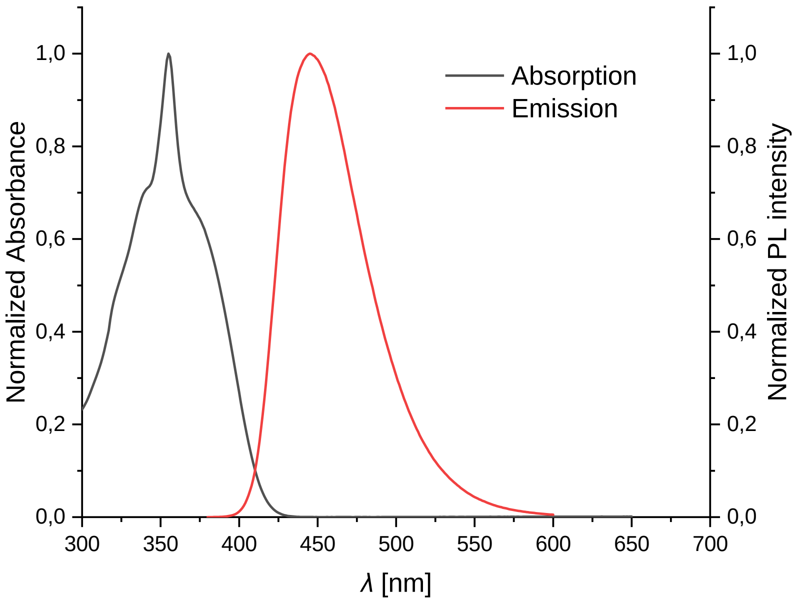

Figure S16: Normalized absorption and emission at 300 K of **1-m-DPS** in toluene.

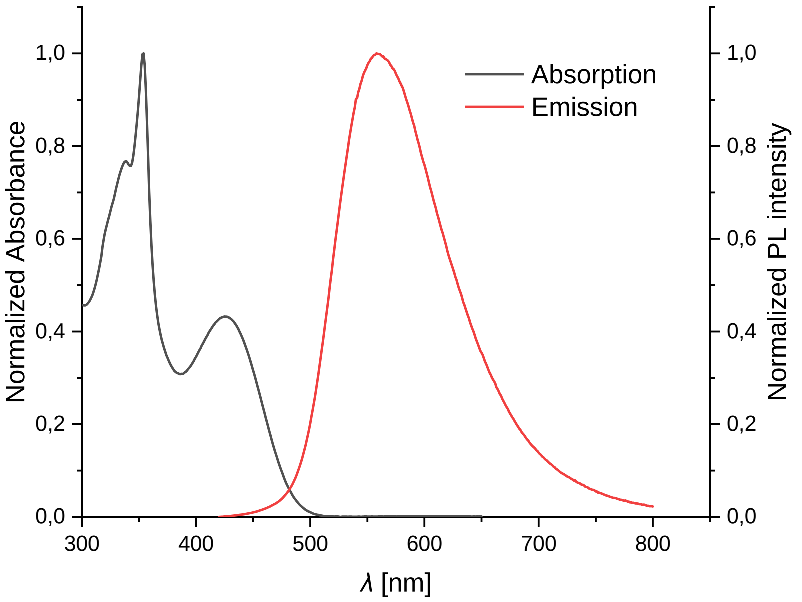

Figure S17: Normalized absorption and emission at 300 K of **1-m-TTO** in toluene.

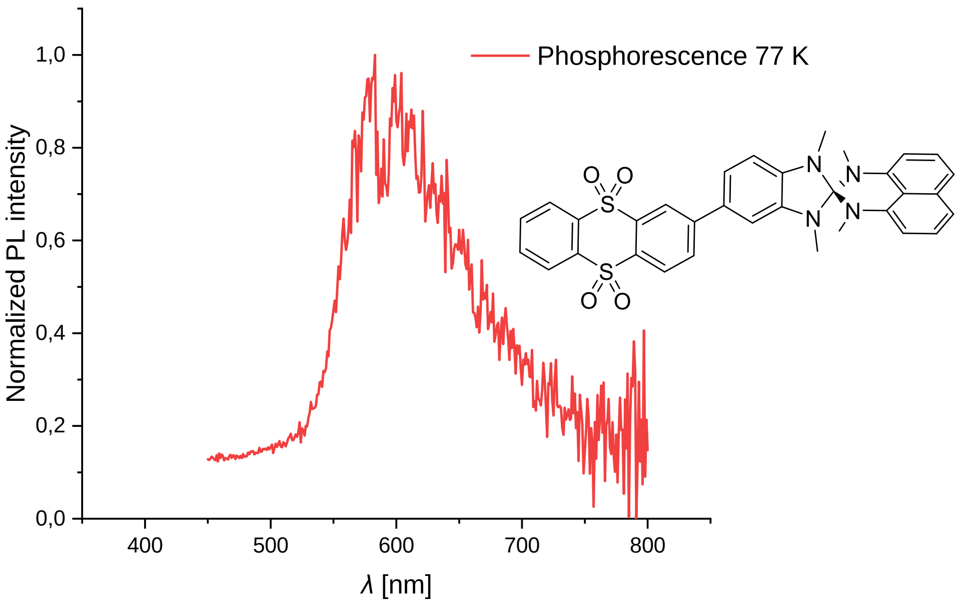

Figure S18: Normalized phosphorescence of **1-m-TTO** at 77 K in toluene.

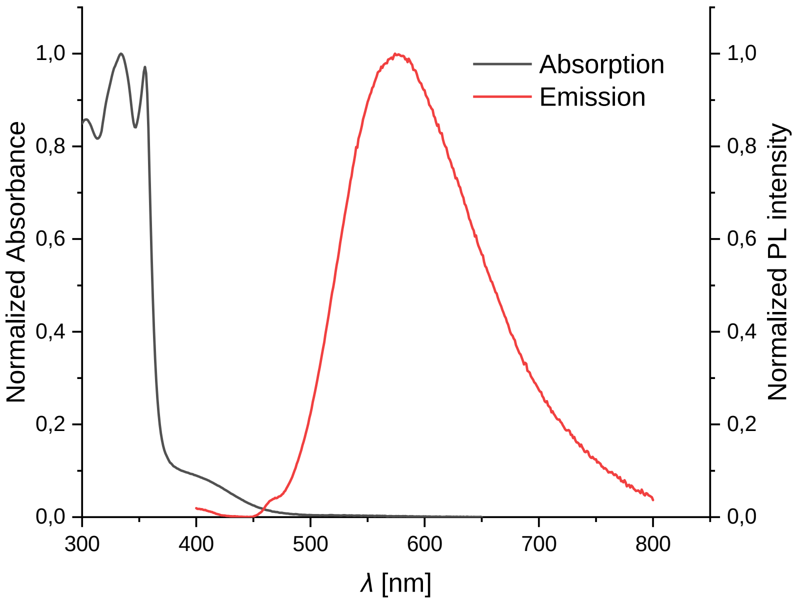

Figure S19: Normalized absorption and emission at 300 K of **1-ms-DBTO** in toluene.

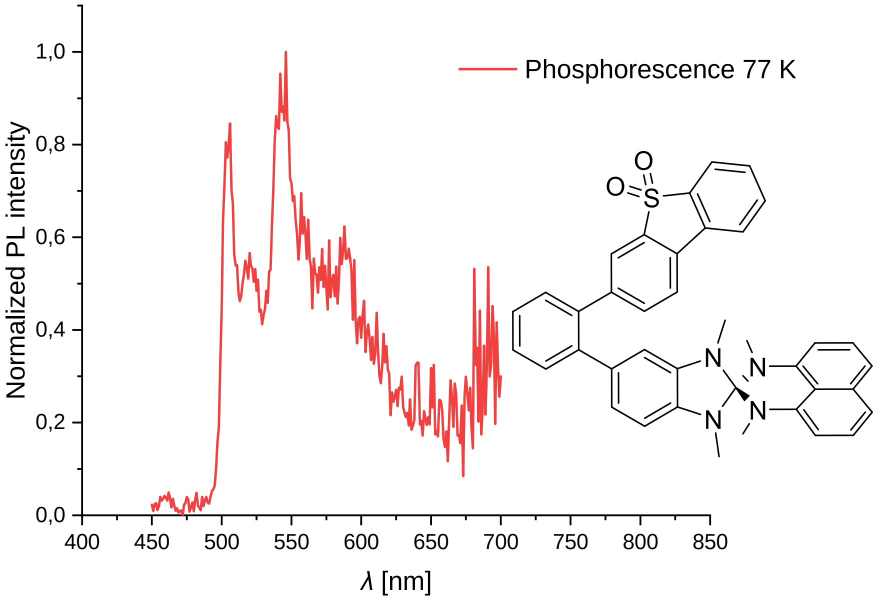

Figure S20: Normalized phosphorescence of **1-ms-DBTO** at 77 K in toluene.

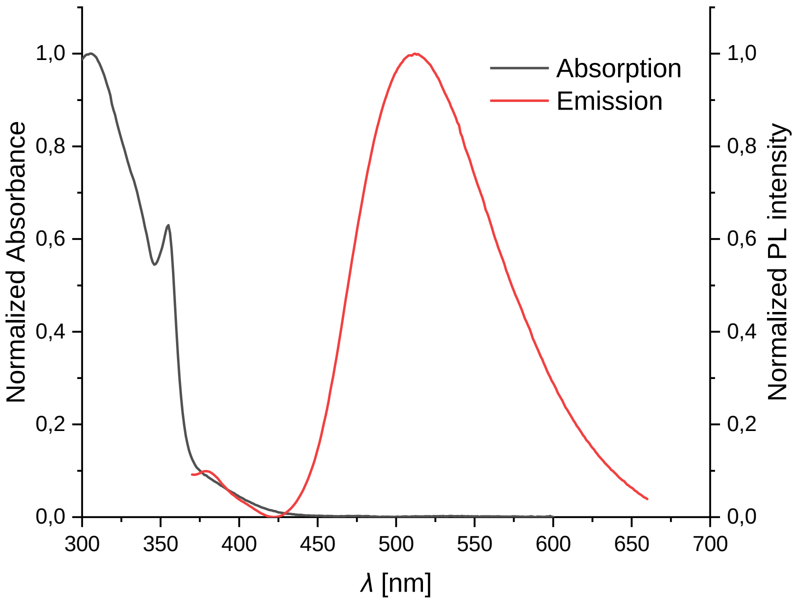

Figure S21: Normalized absorption and emission at 300 K of **1-ms-DPOD** in toluene.

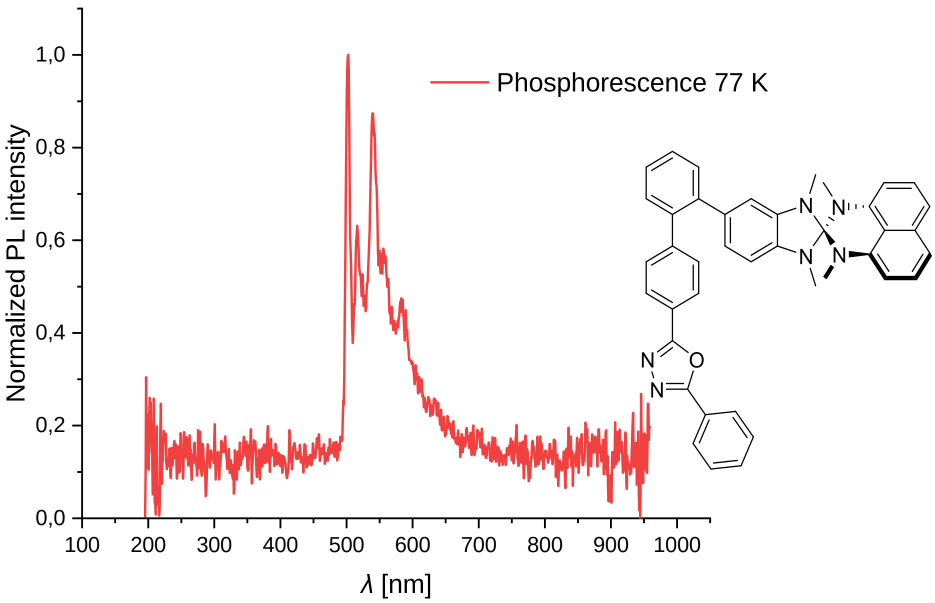

Figure S22: Normalized phosphorescence of **1-ms-DPOD** at 77 K in toluene.

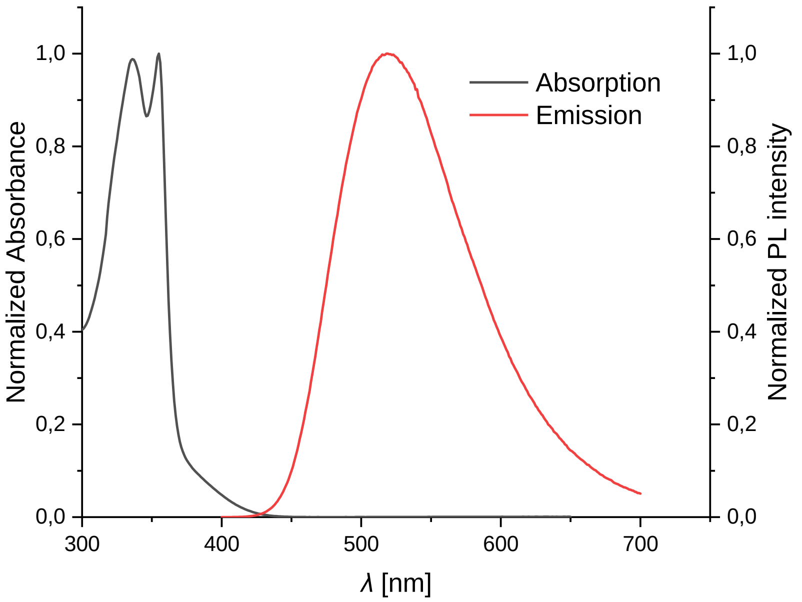

Figure S23: Normalized absorption and emission at 300 K of **1-ms-DPS** in toluene.

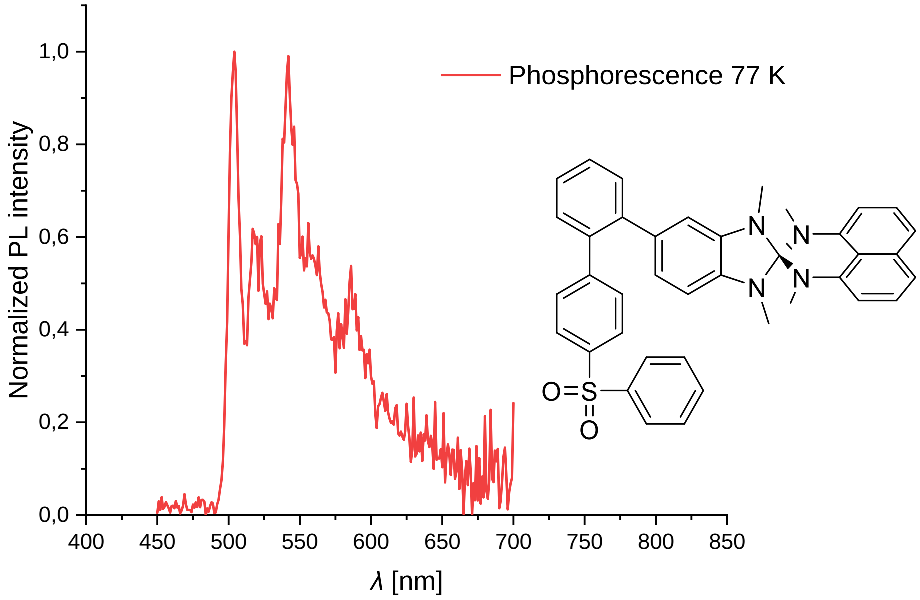

Figure S24: Normalized phosphorescence of **1-ms-DPS** at 77 K in toluene.

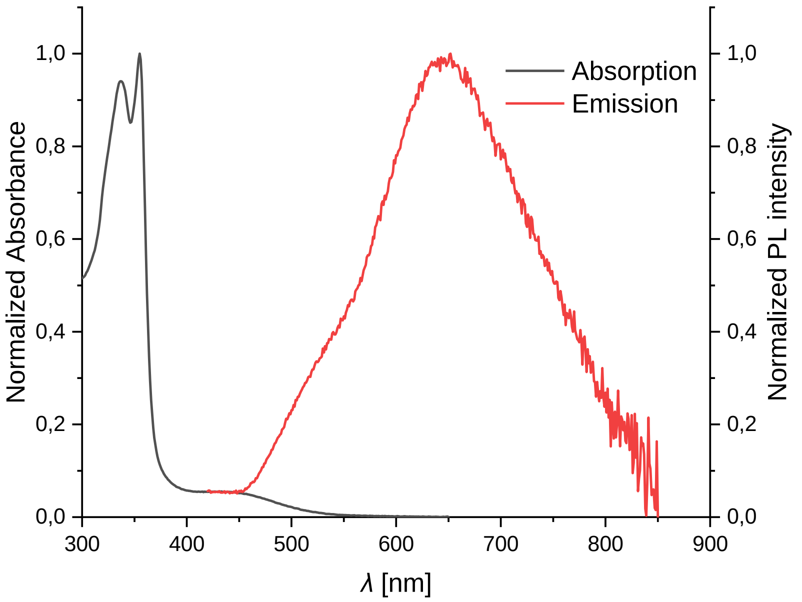

Figure S25: Normalized absorption and emission at 300 K of **1-ms-TTO** in toluene.

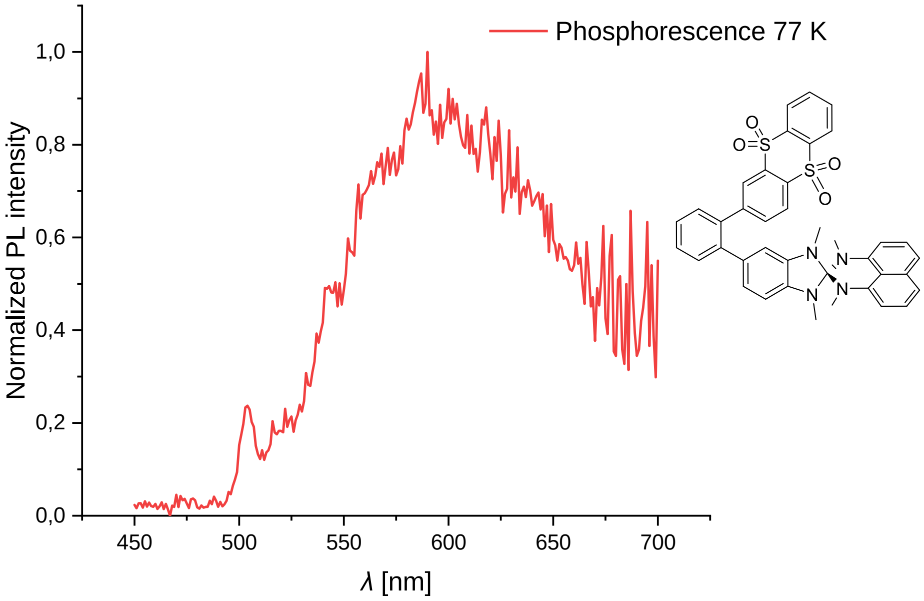

Figure S26: Normalized phosphorescence of **1-ms-TTO** at 77 K in toluene.

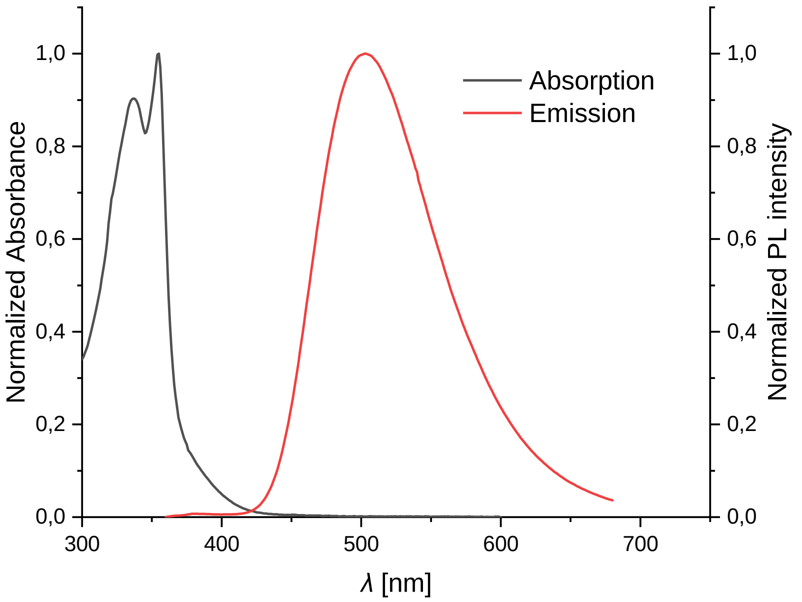

Figure S27: Normalized absorption and emission at 300 K of **1-ms-BN** in toluene.

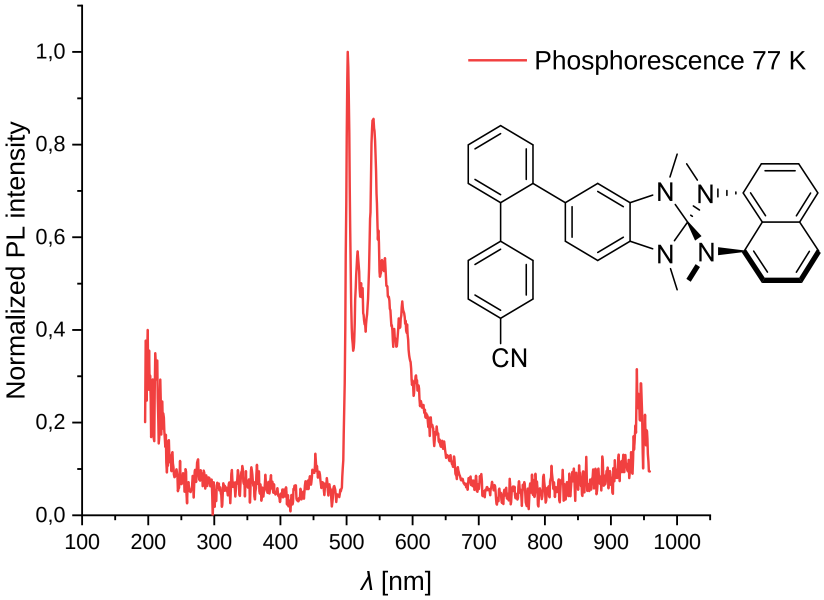

Figure S28: Normalized phosphorescence of **1-ms-BN** at 77 K in toluene.

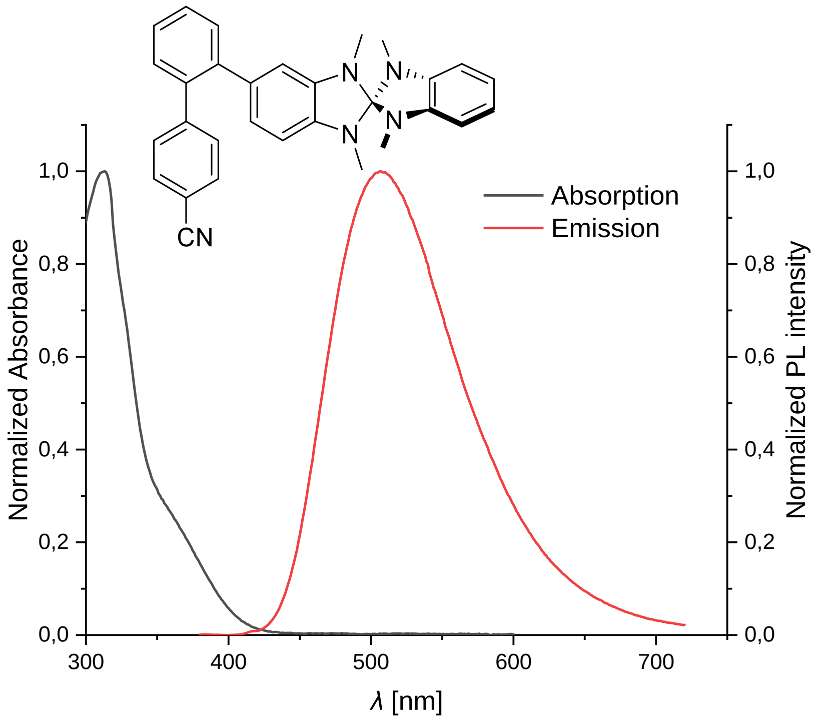

Figure S29: Normalized absorption and emission at 300 K of **2-ms-BN** in toluene.

5. DFT Calculations

5.1 Methods

DFT and TD-DFT calculations were performed with the TURBOMOLE v7.3 program package.[15] The resolution-of-identity (RIJDX for SP)[16] approximation for the Coulomb integrals was used in all DFT calculations employing matching auxiliary basis sets def2-XVP/J.[17,18] Further, the D3 dispersion correction scheme[19,20] with BECKE-JOHNSON damping functional was applied.[21–23] The geometries were optimized without symmetry restrictions at the PBEh-3c-D3/def2-mSVP[24] or B3LYP[25,26]-D3/def2-TZVP[27] level followed by harmonic vibrational frequency analyses to confirm minima as stationary points. Vertical excitation energies were calculated with TD-DFT employing the B3LYP[25,26] functional and the def2-TZVP basis set.[27]

5.2 Calculated Transitions

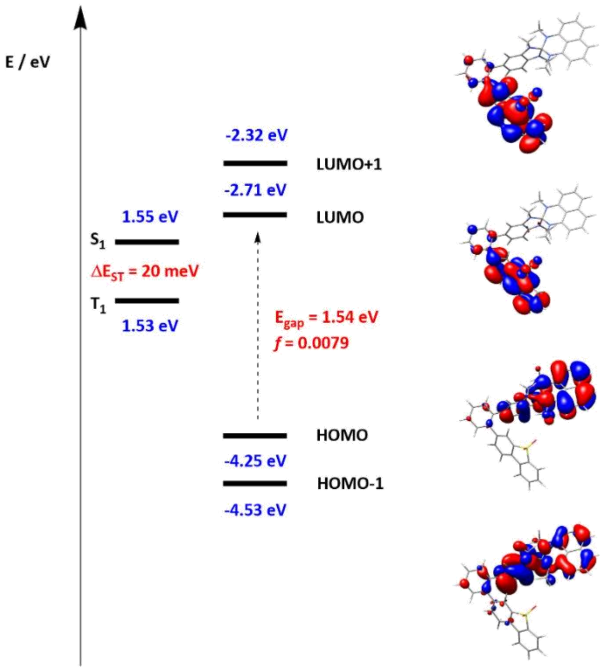

Figure S30: HOMO−1, HOMO, LUMO and LUMO+1 electronic distribution after geometry optimization and TD-DFT calculation (PBEh-3c-D3/def2-mSVP//B3LYP-D3/def2-TZVP) of **1-ms-DBTO**.

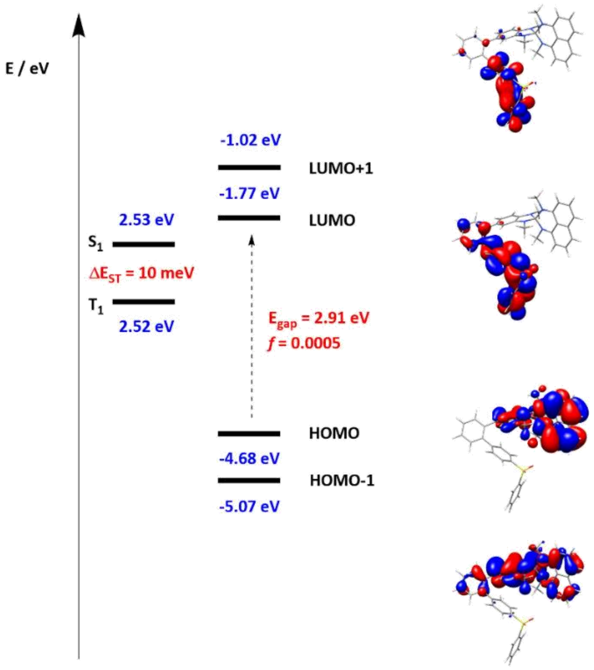

Figure S31: HOMO−1, HOMO, LUMO and LUMO+1 electronic distribution after geometry optimization and TD-DFT calculation (PBEh-3c-D3/def2-mSVP//B3LYP-D3/def2-TZVP) of **1-ms-DPS**.

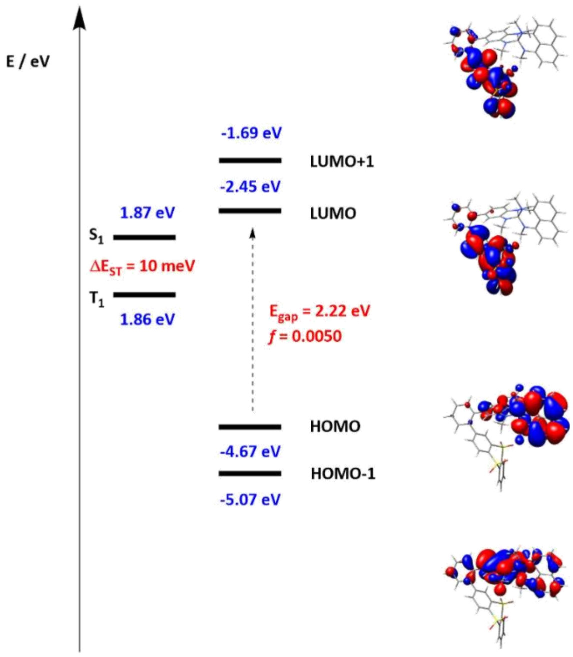

Figure S32: HOMO−1, HOMO, LUMO and LUMO+1 electronic distribution after geometry optimization and TD-DFT calculation (PBEh-3c-D3/def2-mSVP//B3LYP-D3/def2-TZVP) of **1-ms-TTO**.

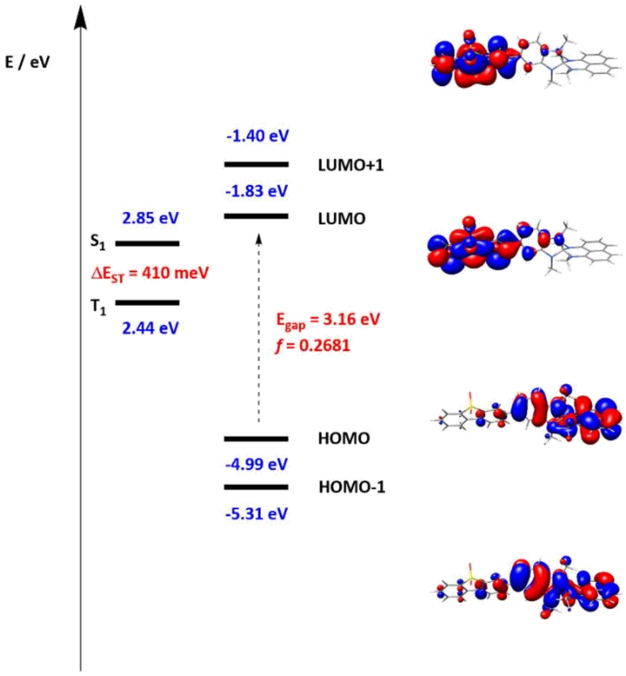

Figure S33: HOMO−1, HOMO, LUMO and LUMO+1 electronic distribution after geometry optimization and TD-DFT calculation (PBEh-3c-D3/def2-mSVP//B3LYP-D3/def2-TZVP) of **1-m-DBTO**.

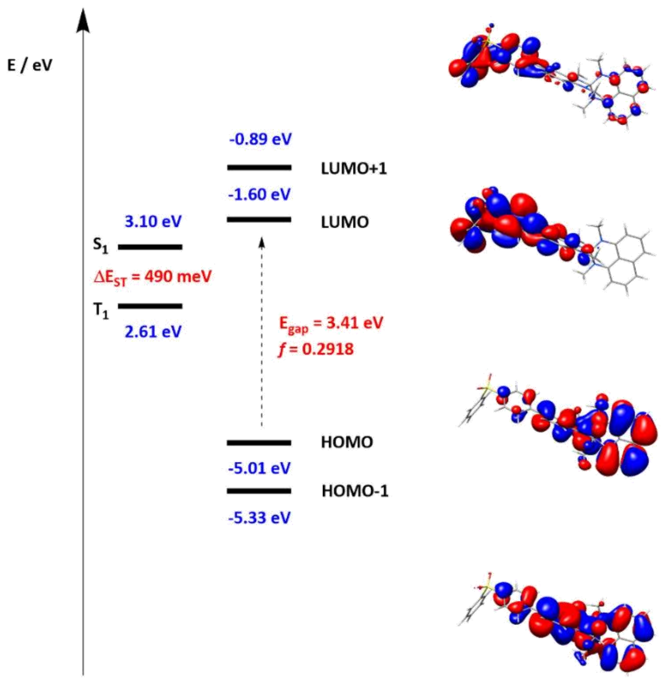

Figure S34: HOMO−1, HOMO, LUMO and LUMO+1 electronic distribution after geometry optimization and TD-DFT calculation (PBEh-3c-D3/def2-mSVP//B3LYP-D3/def2-TZVP) of **1-m-DPS**.

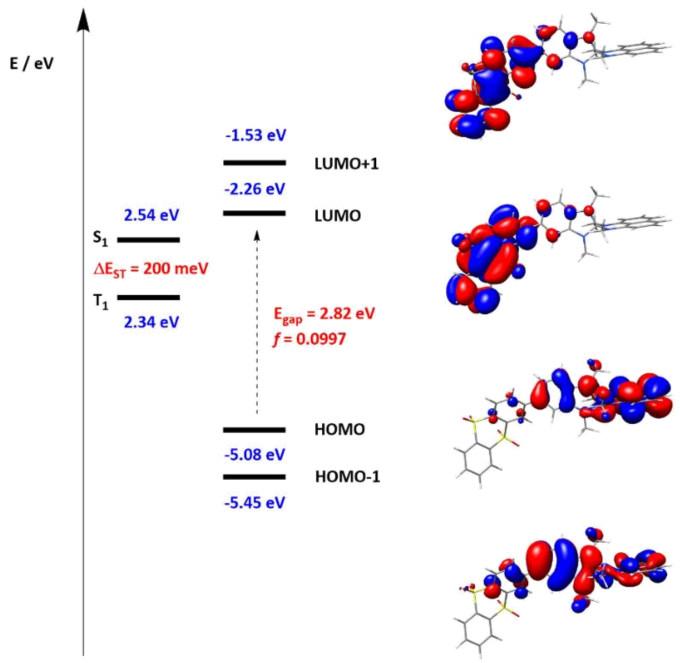

Figure S35: HOMO−1, HOMO, LUMO and LUMO+1 electronic distribution after geometry optimization and TD-DFT calculation (PBEh-3c-D3/def2-mSVP//B3LYP-D3/def2-TZVP) of **1-m-TTO**.

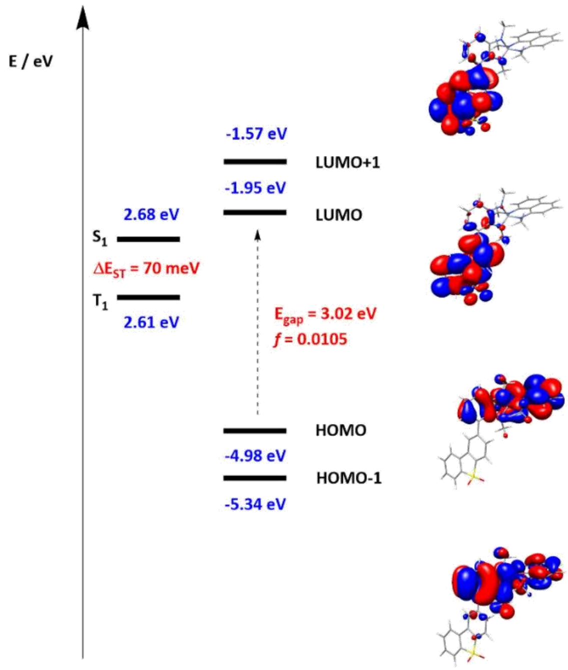

Figure S36: HOMO−1, HOMO, LUMO and LUMO+1 electronic distribution after geometry optimization and TD-DFT calculation (PBEh-3c-D3/def2-mSVP//B3LYP-D3/def2-TZVP) of **1-o-DBTO**.

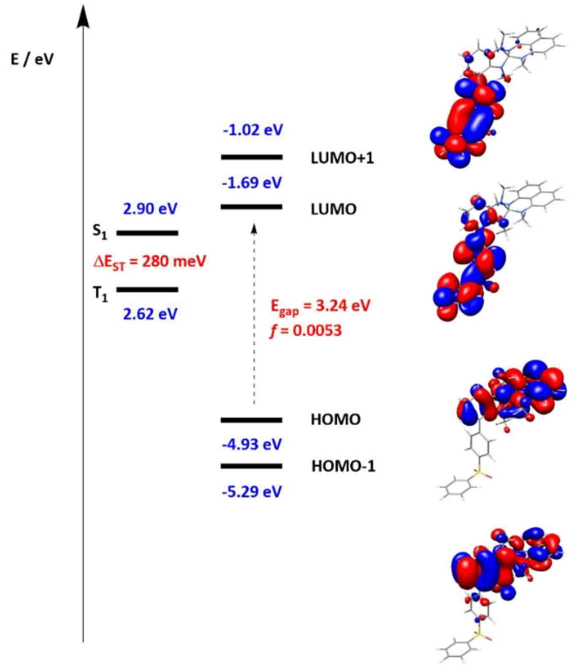

Figure S37: HOMO−1, HOMO, LUMO and LUMO+1 electronic distribution after geometry optimization and TD-DFT calculation (PBEh-3c-D3/def2-mSVP//B3LYP-D3/def2-TZVP) of **1-o-DPS**.

5.3 Cartesian Coordinates of Calculated Structures

Table S1: Coordinates of the calculated structure of **1-ms-BN** (PBEh-3c-D3/def2-mSVP).

	x	y	z		x	y	z
C	−1.16379	0.90965	0.46533	C	7.26370	2.83149	1.98622
C	−0.88907	0.47850	−0.82285	C	7.58286	4.19689	1.82593
C	0.44521	0.39896	−1.26393	C	6.71446	5.02316	1.17156
C	1.44713	0.75764	−0.39356	C	5.50217	4.55101	0.64731
C	1.15855	1.19170	0.91033	C	6.55554	0.13694	2.28465
C	−0.14386	1.27596	1.34820	C	7.76505	0.62757	2.80024
N	2.80929	0.80311	−0.55698	C	8.12249	1.93780	2.66272
C	3.46057	1.40614	0.58693	H	−2.19133	0.96879	0.79903
N	2.34804	1.47659	1.52850	H	0.66454	0.09498	−2.28021
N	4.50870	0.53256	1.06215	H	−0.38340	1.62656	2.34316
N	3.99359	2.70579	0.24784	H	3.49072	2.16123	3.11943
C	2.46650	2.21216	2.75325	H	1.82879	1.76718	3.51787
C	3.04358	3.64786	−0.30773	H	2.19123	3.26617	2.64586
C	3.48147	0.67996	−1.81503	H	2.14806	3.14803	−0.65887
C	4.09905	−0.81931	1.36628	H	3.47872	4.16548	−1.16364
C	−2.45471	9.08330	0.32682	H	2.72806	4.39781	0.42065
C	−1.49247	8.26760	−0.25472	H	3.25781	1.50615	−2.49797
C	−1.83645	6.96224	−0.52837	H	3.20025	−0.25437	−2.30363
C	−3.09305	6.43716	−0.24922	H	4.55622	0.65510	−1.64833
C	−4.04459	7.26111	0.33247	H	3.14117	−1.03640	0.90667
C	−3.71692	8.57969	0.61688	H	4.00653	−0.98831	2.44299
S	−0.77392	5.73503	−1.25507	H	4.82144	−1.53320	0.96727
C	−2.06128	4.51020	−1.20421	H	−2.21991	10.11403	0.55424
C	−1.95490	3.21967	−1.66020	H	−0.50604	8.64672	−0.48638
C	−3.06638	2.37905	−1.55076	H	−5.03357	6.88743	0.56379
C	−4.23481	2.88774	−0.98376	H	−4.45694	9.22539	1.07029
C	−4.32385	4.19718	−0.53099	H	−1.03548	2.85970	−2.10241
C	−3.22316	5.02855	−0.64090	H	−5.09262	2.23539	−0.88015
O	−0.44415	6.08019	−2.62364	H	−5.24869	4.55282	−0.09547
O	0.29165	5.39613	−0.32869	H	−1.18340	−1.84460	−2.07260
C	−1.98001	0.11009	−1.74995	H	−2.97299	−2.55970	−3.60691
C	−1.98221	−1.15913	−2.32628	H	−4.83062	−1.00237	−4.14004
C	−2.99371	−1.56588	−3.17946	H	−4.83599	1.25936	−3.17691

C	−4.03504	−0.69911	−3.47267	H	8.51406	4.57419	2.22789
C	−4.04376	0.56821	−2.91684	H	6.95399	6.07199	1.05170
C	−3.02555	0.99255	−2.06352	H	4.85024	5.25046	0.14727
C	5.16212	3.21600	0.77393	H	6.32065	−0.90632	2.42957
C	5.69482	0.98058	1.60583	H	8.41968	−0.05898	3.32167
C	6.04587	2.34443	1.45560	H	9.05631	2.30509	3.06790

Table S2: Coordinates of the calculated structure of **1-ms-DPS** (PBEh-3c-D3/def2-mSVP).

	x	y	z		x	y	z
C	−0.88599	−4.06621	2.35678	C	5.72835	1.28930	0.24977
C	0.00010	−4.00138	3.39248	C	4.5458	0.71703	0.65732
C	1.14799	−3.19449	3.32935	C	3.45858	1.51388	1.05305
C	−0.11877	−1.78806	−1.10825	C	3.53218	2.88296	1.00615
C	−1.28246	−2.56677	−1.01007	C	4.72610	3.48122	0.56333
C	−1.54464	−3.32927	0.09225	C	5.80755	2.68861	0.21513
C	−0.64688	−3.32073	1.18149	H	−1.77637	−4.67761	2.42451
C	0.52345	−2.53127	1.09608	H	−0.18003	−4.5708	4.29512
C	1.41782	−2.4559	2.19200	H	1.80396	−3.16449	4.1860
C	0.80226	−1.79159	−0.07808	H	0.02312	−1.18571	−1.9933
C	3.84136	5.63484	−0.37415	H	−1.98266	−2.55010	−1.83513
C	3.89914	7.02316	−0.46193	H	−2.44188	−3.93168	0.15060
C	4.89006	7.73925	0.18925	H	3.17416	7.54354	−1.07578
C	5.83840	7.06489	0.94240	H	4.92497	8.81697	0.10161
C	5.77925	5.68495	1.05075	H	6.61703	7.61320	1.45619
C	4.78675	4.95024	0.40700	H	6.49824	5.15948	1.66644
O	0.16231	1.24637	−3.67289	H	2.46564	2.40398	−3.4003
O	−1.63592	2.64748	−2.55417	H	4.19284	3.66090	−2.18684
C	2.18015	3.19196	−2.71662	H	1.16504	5.96401	−0.2358
C	3.14933	3.9004	−2.03370	H	−0.57713	4.69471	−1.42854
C	2.79937	4.90508	−1.13125	H	−1.16397	5.37213	−8.12365
C	1.45084	5.19753	−0.94515	H	−2.51069	5.93751	−6.13216
C	0.46768	4.48533	−1.61331	H	−2.19489	4.68150	−4.0289
C	0.84062	3.48352	−2.49537	H	0.81184	2.28168	−5.92121
S	−0.41185	2.54161	−3.33077	H	0.49099	3.54198	−8.02106
C	−1.01982	4.82546	−7.20124	H	2.61725	−0.99051	4.11179
C	−1.77479	5.14645	−6.08218	H	4.03745	−0.63082	3.13010
C	−1.59667	4.4479	−4.89893	H	3.75350	−2.27575	3.67054
C	−0.65223	3.43323	−4.85137	H	0.47026	0.30962	1.98698

C	0.09953	3.09397	−5.96638	H	0.61499	1.42717	0.62189
C	−0.08742	3.79966	−7.14419	H	1.02989	1.94852	2.26280
C	3.26352	−1.36765	3.31438	H	2.14194	−0.95109	−2.2299
C	1.07363	1.11167	1.56524	H	3.23962	0.09189	−1.34987
C	2.23676	−0.32314	−1.34465	H	1.52866	0.50327	−1.44594
C	4.88477	−1.6997	0.20446	H	5.89687	−1.71642	0.61186
N	2.01083	−1.11985	−0.15807	H	4.95435	−1.66942	−0.88823
N	2.5211	−1.63047	2.10242	H	4.39620	−2.62902	0.48906
N	4.15289	−0.59545	0.74734	H	6.56975	0.68571	−0.06461
C	2.73426	−0.69798	1.02324	H	2.68527	3.49726	1.28232
N	2.43329	0.67667	1.41472	H	6.71705	3.15577	−0.14083

Table S3: Coordinates of the calculated structure of **1-ms-TTO** (PBEh-3c-D3/def2-mSVP).

	x	y	z		x	y	z
C	−1.46403	−0.07622	0.77413	C	5.12000	3.21921	1.75223
C	−1.37868	0.19346	−0.58249	C	6.25358	3.82208	2.34642
C	−0.21456	0.79108	−1.10363	C	6.18040	5.17414	2.74331
C	0.81248	1.09044	−0.24486	C	5.02282	5.87426	2.55268
C	0.71493	0.82191	1.13185	C	3.88920	5.28186	1.97803
C	−0.42184	0.24516	1.65247	C	6.32323	1.13937	1.53710
N	2.03779	1.66076	−0.47635	C	7.44252	1.74512	2.12969
C	2.74367	1.92324	0.76149	C	7.42365	3.05135	2.52509
N	1.88146	1.23116	1.71745	H	−2.37048	−0.51358	1.17311
N	4.05926	1.32803	0.70605	H	−0.13233	0.99775	−2.16286
N	2.83279	3.34033	1.00763	H	−0.52417	0.05506	2.71255
C	2.07645	1.37902	3.12947	H	3.13203	1.53810	3.34407
C	1.57711	4.06391	0.99960	H	1.77612	0.46665	3.64617
C	2.40153	2.32517	−1.69249	H	1.51171	2.21644	3.55124
C	4.08706	−0.08266	0.39592	H	0.77470	3.45477	0.59974
C	−4.53971	8.60539	0.30554	H	1.63931	4.95461	0.37430
C	−4.11576	7.33917	0.68566	H	1.28054	4.37181	2.00479
C	−3.10178	6.72435	−0.02579	H	1.79193	3.21429	−1.88527
C	−2.53385	7.35544	−1.12724	H	2.29866	1.64512	−2.53985
C	−2.97620	8.60431	−1.52313	H	3.44438	2.62955	−1.63852
C	−3.97298	9.23424	−0.79032	H	3.16750	−0.37809	−0.09742
S	−2.52359	5.13134	0.53734	H	4.21094	−0.70218	1.28928
C	−2.33148	4.26533	−1.01178	H	4.90762	−0.29958	−0.28930
C	−1.82534	4.90646	−2.14077	H	−5.32724	9.09325	0.86331

S	−1.22710	6.57909	−2.06221	H	−4.57663	6.83208	1.52225
C	−2.73422	2.94854	−1.07496	H	−2.55477	9.07675	−2.39983
C	−2.62898	2.24264	−2.27620	H	−4.31662	10.21455	−1.09067
C	−2.15908	2.90400	−3.40353	H	−3.14593	2.46191	−0.20076
C	−1.75812	4.23188	−3.34394	H	−2.08968	2.36958	−4.34237
O	−3.60893	4.50019	1.26466	H	−1.39914	4.73660	−4.23053
O	−1.25878	7.13137	−3.40451	H	−2.55711	−2.18591	−0.89920
O	−1.23248	5.31474	1.16179	H	−4.38058	−2.79889	−2.43858
O	−0.00804	6.60563	−1.28498	H	−5.33248	−1.09406	−3.96644
C	−2.49745	−0.13457	−1.48998	H	−4.49704	1.22295	−3.87831
C	−2.99768	−1.43404	−1.54134	H	7.04545	5.64159	3.19533
C	−4.01317	−1.78137	−2.41697	H	4.96418	6.91224	2.85420
C	−4.54315	−0.83042	−3.27546	H	2.99896	5.87950	1.85592
C	−4.06292	0.46775	−3.23447	H	6.39352	0.10239	1.24665
C	−3.05729	0.82923	−2.34204	H	8.33576	1.15050	2.27240
C	3.92579	3.95884	1.57472	H	8.29407	3.50774	2.97818
C	5.16619	1.86795	1.33073				

Table S4: Coordinates of the calculated structure of **1-m-DBTO** (PBEh-3c-D3/def2-mSVP).

	x	y	z		x	y	z
C	−1.28940	−0.33599	1.35623	C	6.25498	5.21598	2.28965
C	−1.61554	0.12619	0.08861	C	5.05346	5.79567	2.57845
C	−0.69243	0.92384	−0.61692	C	3.84161	5.21739	2.17008
C	0.51448	1.20982	−0.02883	C	6.29386	1.59160	0.14336
C	0.83524	0.73346	1.25328	C	7.49571	2.18639	0.55709
C	−0.06519	−0.03282	1.95868	C	7.51009	3.36160	1.25142
N	1.58682	1.93819	−0.48177	H	−2.01317	−0.91314	1.91684
C	2.60176	2.08155	0.53580	H	−0.92171	1.27573	−1.61495
N	2.09647	1.17076	1.55593	H	0.15234	−0.38660	2.95759
N	3.88006	1.65665	0.01446	H	3.77939	1.26041	2.76859
N	2.67461	3.45527	0.99087	H	2.57557	0.08203	3.25663
C	2.70782	1.08463	2.84902	H	2.29750	1.80366	3.56539
C	1.42654	4.03041	1.43888	H	0.57884	3.49305	1.02651
C	1.59030	2.74102	−1.66661	H	1.34659	5.06440	1.10083
C	3.90520	0.32728	−0.55453	H	1.33009	4.02017	2.52947
C	−2.91868	−0.19843	−0.51186	H	1.30766	2.14091	−2.53338
C	−3.48611	−1.46552	−0.34531	H	2.59435	3.12153	−1.84049
C	−4.71173	−1.72335	−0.91094	H	0.90622	3.59370	−1.60159
C	−5.41815	−0.78883	−1.65774	H	2.90007	0.00143	−0.79901
C	−4.85632	0.46610	−1.83198	H	4.34458	−0.40564	0.12836
C	−3.62582	0.74795	−1.26063	H	4.48216	0.32395	−1.48022
S	−5.59100	−3.26739	−0.81709	H	−2.95518	−2.23604	0.19890
C	−6.93080	−2.62364	−1.79587	H	−5.37175	1.23099	−2.39857
C	−8.06615	−3.31176	−2.16215	H	−3.21133	1.74109	−1.37627
C	−9.00918	−2.64414	−2.93349	H	−8.21943	−4.33903	−1.85938
C	−8.79573	−1.32471	−3.31299	H	−9.91235	−3.15409	−3.23933
C	−7.64428	−0.64809	−2.93432	H	−9.53814	−0.81599	−3.91321
C	−6.69538	−1.30380	−2.16458	H	−7.49546	0.37947	−3.23979
O	−4.88325	−4.30114	−1.54624	H	7.18293	5.66991	2.61188
O	−6.03141	−3.51804	0.54113	H	5.02079	6.72095	3.13907
C	3.84138	4.04033	1.44509	H	2.92148	5.71544	2.43443
C	5.08349	2.19911	0.42216	H	6.34212	0.65713	−0.39417
C	5.07706	3.41829	1.14316	H	8.42658	1.68814	0.31891
C	6.29342	4.00512	1.56484	H	8.44299	3.80834	1.56935

Table S5: Coordinates of the calculated structure of **1-m-DPS** (PBEh-3c-D3/def2-mSVP).

	x	y	z		x	y	z
C	−1.63095	0.04110	1.35307	C	5.11698	5.80567	2.16834
C	−1.82628	0.33556	0.01059	C	3.88961	5.25371	1.77024
C	−0.78691	0.94307	−0.72212	C	6.16991	1.20651	0.47131
C	0.39783	1.21474	−0.08447	C	7.38847	1.77680	0.87028
C	0.58470	0.90951	1.27381	C	7.45310	3.04086	1.38145
C	−0.42848	0.33049	2.00447	H	−2.44200	−0.38428	1.92935
N	1.56014	1.78176	−0.54706	H	−0.91511	1.16448	−1.77418
C	2.50755	2.00019	0.52180	H	−0.31369	0.11379	3.05817
N	1.85579	1.28733	1.61418	H	3.44646	1.45119	2.93737
N	3.77942	1.41091	0.17463	H	2.12989	0.42935	3.48450
N	2.66141	3.41615	0.78507	H	1.95220	2.19103	3.52543
C	2.36210	1.35267	2.95290	H	0.57694	3.60515	0.65744
C	1.43735	4.13851	1.04813	H	1.46203	5.11179	0.55629
C	1.71134	2.41141	−1.82363	H	1.27134	4.30153	2.11792
C	3.73494	0.01304	−0.19206	H	1.43078	1.72233	−2.62209
C	−3.65563	0.91547	−1.58529	H	2.75487	2.68105	−1.97076
C	−4.86264	0.64523	−2.20438	H	1.10468	3.31758	−1.92348
C	−5.54554	−0.51789	−1.88392	H	2.72286	−0.27784	−0.45133
C	−5.03612	−1.39902	−0.94098	H	4.07968	−0.63619	0.61799
C	−3.82464	−1.12136	−0.33675	H	4.35997	−0.16841	−1.06755
C	−3.11057	0.03969	−0.64415	H	−3.14520	1.84265	−1.81160
S	−7.09404	−0.87571	−2.67348	H	−5.28070	1.34753	−2.91261
C	−6.62288	−1.96171	−4.00503	H	−5.57838	−2.29763	−0.67951
C	−6.72338	−3.33358	−3.83282	H	−3.41497	−1.82732	0.37374
C	−6.35169	−4.17235	−4.87231	H	−7.09988	−3.74160	−2.90490
C	−5.89060	−3.63933	−6.06681	H	−6.43023	−5.24425	−4.75025
C	−5.80601	−2.26360	−6.23112	H	−5.60280	−4.29762	−6.87579
C	−6.17708	−1.41618	−5.20025	H	−5.45866	−1.84911	−7.16791
O	−7.90066	−1.65113	−1.74486	H	−6.13081	−0.34342	−5.32974
O	−7.58289	0.35907	−3.26588	H	7.22603	5.54676	2.35926
C	3.84120	3.98313	1.22758	H	5.12299	6.80551	2.58283
C	4.99534	1.92866	0.57574	H	2.99640	5.84607	1.89618
C	5.04109	3.24145	1.10532	H	6.17698	0.19964	0.08312
C	6.27300	3.80388	1.51485	H	8.29094	1.18766	0.77039
C	6.28577	5.11098	2.04787	H	8.39802	3.46815	1.69046

Table S6: Coordinates of the calculated structure of **1-m-TTO** (PBEh-3c-D3/def2-mSVP).

	x	y	z		x	y	z
C	−0.96469	−0.74767	1.37308	C	6.42632	3.93878	1.37944
C	−1.58026	0.08434	0.44789	C	6.55208	4.83855	2.45985
C	−0.85301	1.14954	−0.12158	C	5.45108	5.17687	3.19234
C	0.45643	1.32277	0.24917	C	4.18301	4.65243	2.89783
C	1.07077	0.47290	1.18592	C	6.10714	2.12956	−0.73506
C	0.36449	−0.56029	1.76108	C	7.36682	2.66692	−0.42811
N	1.38731	2.24991	−0.14430	C	7.53667	3.54875	0.59984
C	2.61234	2.13160	0.61339	H	−1.53487	−1.53616	1.84660
N	2.36125	0.89219	1.34311	H	−1.31090	1.79912	−0.85699
N	3.73404	2.01084	−0.28674	H	0.81116	−1.20566	2.50550
N	2.78282	3.27293	1.48613	H	4.27666	0.74121	2.12837
C	3.26033	0.42438	2.35628	H	3.26009	−0.66586	2.38216
C	1.67291	3.55698	2.36789	H	3.00505	0.79232	3.35504
C	1.10670	3.40272	−0.94639	H	0.75495	3.11321	1.99674
C	3.63214	0.95313	−1.26729	H	1.50626	4.63314	2.42764
C	−2.98736	−0.12318	0.07970	H	1.83995	3.18089	3.38238
C	−3.82842	0.96651	−0.16590	H	0.63875	3.10713	−1.88699
C	−5.15248	0.76808	−0.49709	H	2.03861	3.91025	−1.18495
C	−5.67107	−0.51668	−0.61552	H	0.44537	4.11541	−0.44335
C	−4.84505	−1.60646	−0.40587	H	2.60053	0.63694	−1.37740
C	−3.52088	−1.40508	−0.05442	H	4.22982	0.07864	−0.99401
S	−6.18064	2.20621	−0.73736	H	3.96860	1.30827	−2.24222
C	−7.12888	1.71207	−2.16780	H	−3.44775	1.97547	−0.07854
C	−7.64089	0.42496	−2.28774	H	−5.22697	−2.60957	−0.53801
S	−7.38025	-0.80057	−1.01479	H	−2.87822	−2.26498	0.07941
C	−7.36119	2.65171	−3.15581	H	−6.93506	3.64218	−3.07362
C	−8.13539	2.30426	−4.25430	H	−8.32701	3.03817	−5.02507
C	−8.64427	1.02191	−4.37440	H	−9.23417	0.75146	−5.23944
C	−8.38677	0.07041	−3.39705	H	−8.75581	−0.94068	−3.50153
O	−5.31801	3.30199	−1.14514	H	7.52508	5.24817	2.69743
O	−7.47587	−2.10812	−1.64013	H	5.54559	5.86171	4.02508
O	−7.07090	2.35065	0.39017	H	3.34977	4.94874	3.51629
O	−8.20258	−0.46727	0.12464	H	6.03336	1.42916	−1.55267
C	4.01916	3.78253	1.83624	H	8.21690	2.36250	−1.02489
C	4.99494	2.49905	−0.00179	H	8.51367	3.95307	0.82986
C	5.15067	3.41044	1.07093				

Table S7: Coordinates of the calculated structure of **1-o-DBTO** (PBEh-3c-D3/def2-mSVP).

	x	y	z		x	y	z
C	1.35935	−0.11996	4.55923	C	2.95934	−6.34551	−2.25345
C	1.93723	−0.64031	3.42254	C	2.11390	−6.15762	−1.16700
C	1.72728	−0.06512	2.15399	C	2.04269	−4.93156	−0.51970
C	0.88696	1.03376	2.08359	C	2.82958	−3.88281	−0.96960
C	0.32416	1.57704	3.25453	O	4.14652	−2.09381	−3.74331
C	0.53862	1.01070	4.48978	O	5.99366	−2.81251	−2.20706
N	0.41216	1.77220	1.02054	H	1.54944	−0.58180	5.51835
C	−0.52029	2.79165	1.45402	H	2.59867	−1.49385	3.49616
N	−0.41378	2.67462	2.89673	H	0.08476	1.41992	5.38256
N	−0.08724	4.08558	0.97671	H	−2.22213	2.79609	3.99107
N	−1.85295	2.49706	0.96552	H	−1.61313	4.31472	3.32039
C	−1.31522	3.36805	3.76816	H	−0.81770	3.60066	4.71021
C	−2.35742	1.18052	1.28292	H	−1.54679	0.50269	1.52910
C	0.53128	1.46451	−0.37809	H	−2.87821	0.75847	0.42220
C	1.26857	4.44839	1.32051	H	−3.05250	1.19427	2.12862
C	−2.77737	3.47220	0.64429	H	1.50803	1.73211	−0.78448
C	−0.95833	5.10629	0.65111	H	−0.22369	2.02471	−0.92560
C	−0.53259	6.41207	0.49058	H	0.36657	0.40297	−0.56892
C	−1.44660	7.41515	0.13336	H	1.84107	3.56469	1.58161
C	−2.77137	7.14273	−0.05175	H	1.31055	5.14091	2.16633
C	−3.24541	5.82364	0.11543	H	1.76148	4.91654	0.46738
C	−2.32991	4.80387	0.46690	H	0.50134	6.68674	0.63239
C	−4.60711	5.49279	−0.05580	H	−1.08061	8.42624	0.00972
C	−5.02001	4.20441	0.12540	H	−3.46737	7.92542	−0.32360
C	−4.12051	3.18767	0.48141	H	−5.31047	6.26892	−0.32769
C	2.43485	−0.63759	0.98993	H	−6.06410	3.94884	−0.00134
C	3.36303	0.12597	0.27899	H	−4.50448	2.18856	0.61925
C	4.06052	−0.40818	−0.79461	H	3.55144	1.14647	0.58659
C	3.81847	−1.72103	−1.13368	H	4.78041	0.18764	−1.33991
C	2.91686	−2.51572	−0.43684	H	1.50690	−2.55772	1.18724
C	2.22470	−1.96838	0.63007	H	4.41535	−5.43518	−3.55780
S	4.58043	−2.61999	−2.46418	H	3.00119	−7.30845	−2.74369
C	3.66728	−4.09309	−2.05839	H	1.50214	−6.97909	−0.81911
C	3.75245	−5.30181	−2.71332	H	1.37961	−4.80366	0.32604

Table S8: Coordinates of the calculated structure of **1-o-DPS** (PBEh-3c-D3/def2-mSVP).

	x	y	z		x	y	z
C	1.55742	−0.48752	3.63759	C	8.05302	−3.70462	−2.67061
C	2.05156	−0.78051	2.38573	C	6.88670	−4.38653	−2.98517
C	1.75070	0.01688	1.26457	C	5.75981	−3.68840	−3.39014
C	0.90708	1.09787	1.45986	O	3.49560	−2.32838	−4.67771
C	0.42818	1.40600	2.74744	O	4.82141	−0.16203	−4.59797
C	0.73264	0.62448	3.83790	H	1.81533	−1.11723	4.47805
N	0.35612	2.01308	0.58766	H	2.71376	−1.62591	2.25132
C	−0.55500	2.91103	1.26553	H	0.34305	0.85390	4.82083
N	−0.33778	2.53866	2.65167	H	−2.06654	2.41340	3.86793
N	−0.17723	4.28137	1.00257	H	−1.52105	4.04365	3.45325
N	−1.91706	2.67385	0.82893	H	−0.62327	3.10237	4.62885
C	−1.18323	3.03733	3.69540	H	−1.54232	0.61827	0.98722
C	−2.37801	1.30802	0.93339	H	−2.95989	1.03810	0.05100
C	0.36764	1.96187	−0.84884	H	−3.00391	1.14662	1.81699
C	1.19594	4.61270	1.30636	H	1.31512	2.29944	−1.27118
C	−2.87620	3.66524	0.75873	H	−0.41842	2.61088	−1.22879
C	−1.08384	5.31982	0.92856	H	0.17691	0.95180	−1.21524
C	−0.68891	6.64433	0.97500	H	1.79712	3.71153	1.36355
C	−1.64062	7.67023	0.87064	H	1.29286	5.14561	2.25679
C	−2.97202	7.40003	0.73696	H	1.61698	5.23688	0.51692
C	−3.41446	6.06008	0.69792	H	0.34902	6.91668	1.08934
C	−2.46141	5.01875	0.79263	H	−1.29837	8.69659	0.90276
C	−4.78077	5.72888	0.56964	H	−3.69715	8.19981	0.66281
C	−5.16151	4.41822	0.54574	H	−5.51308	6.52196	0.49382
C	−4.22415	3.37864	0.64580	H	−6.20882	4.16154	0.45189
C	2.37189	−0.31771	−0.03331	H	−4.58310	2.36108	0.62805
C	3.25156	0.56927	−0.65128	H	3.46763	1.51872	−0.17796
C	3.86086	0.24857	−1.85201	H	4.53065	0.95092	−2.32917
C	3.59688	−0.97978	−2.43809	H	2.50663	−2.82749	−2.31709
C	2.72784	−1.88153	−1.84164	H	1.43928	−2.24181	−0.17344
C	2.12740	−1.54881	−0.64021	H	7.01871	−0.53621	−3.26659
S	4.37157	−1.39981	−3.98198	H	9.01507	−1.79180	−2.53397
C	5.81670	−2.30524	−3.46977	H	8.93065	−4.25377	−2.35641
C	6.98125	−1.61284	−3.17175	H	6.85517	−5.46594	−2.92351
C	8.10088	−2.32096	−2.76664	H	4.85305	−4.21504	−3.65437

Table S9: Coordinates of the calculated structure of **1-o-TTO** (PBEh-3c-D3/def2-mSVP).

	x	y	z		x	y	z
C	0.69445	−0.37252	4.63959	C	4.14767	−6.27894	−0.65194
C	1.34268	−0.89346	3.54274	C	3.89608	−4.98191	−1.06098
C	1.31604	−0.24551	2.29179	O	3.76762	−2.60728	−3.41058
C	0.57769	0.92258	2.19111	O	5.86800	−1.86599	−2.23777
C	−0.05590	1.46378	3.32672	S	2.25713	−4.31977	−0.80897
C	−0.01686	0.82883	4.54620	O	1.57580	−4.25138	−2.08029
N	0.27268	1.73729	1.12290	O	1.66769	−5.02987	0.31204
C	−0.61922	2.81248	1.51198	H	0.74210	−0.89063	5.58741
N	−0.66144	2.63402	2.95267	H	1.91740	−1.80546	3.63960
N	−0.04342	4.08269	1.13453	H	−0.52635	1.23695	5.40889
N	−1.91540	2.64219	0.88895	H	−2.56108	2.85557	3.86128
C	−1.59447	3.36044	3.76254	H	−1.76882	4.34711	3.33658
C	−2.54830	1.35924	1.09880	H	−1.18283	3.50973	4.76110
C	0.48265	1.45881	-0.27127	H	−1.82227	0.60941	1.39496
C	1.29458	4.32413	1.62381	H	−3.00913	1.01000	0.17384
C	−2.72724	3.69960	0.52439	H	−3.32248	1.39973	1.87152
C	−0.79959	5.18241	0.77735	H	1.50501	1.66693	−0.59202
C	−0.26305	6.45524	0.71922	H	−0.18413	2.08610	−0.85894
C	−1.05957	7.54113	0.32413	H	0.25867	0.41762	−0.50876
C	−2.37636	7.38196	0.00186	H	1.77236	3.38824	1.89352
C	−2.96259	6.09916	0.06209	H	1.30380	4.97448	2.50346
C	−2.16531	4.99706	0.45105	H	1.90191	4.78859	0.84581
C	−4.32187	5.88443	−0.25222	H	0.76970	6.64143	0.97088
C	−4.84624	4.62678	−0.17098	H	−0.60817	8.52398	0.28281
C	−4.06514	3.52889	0.22155	H	−2.98151	8.22737	−0.29817
C	2.10140	−0.81505	1.18111	H	−4.93475	6.72380	−0.55346
C	3.12301	−0.08515	0.57891	H	−5.88896	4.45971	−0.40816
C	3.89590	−0.62840	−0.43491	H	−4.53474	2.55877	0.27527
C	3.63712	−1.91488	−0.87159	H	3.33750	0.91506	0.93256
C	2.63594	−2.66362	−0.26303	H	4.70629	−0.05975	−0.87036
C	1.88535	−2.12962	0.76598	H	1.12078	−2.72594	1.24718
S	4.59365	−2.56227	−2.22697	H	6.93728	−4.17186	−2.31051
C	4.89614	−4.23015	−1.66766	H	7.37536	−6.51241	−1.64652
C	6.15321	−4.77081	−1.86790	H	5.59901	−7.84827	−0.57059
C	6.39646	−6.08252	−1.48437	H	3.37718	−6.84954	−0.15182
C	5.40031	−6.83162	−0.88086				

Table S10: Coordinates of the calculated structure of **2-ms-BN** (B3LYP−D3/def2−TZVP).

	x	y	z		x	y	z
C	−6.30943	4.64055	−0.60497	C	−0.58024	1.09118	−0.10232
C	−6.72778	3.64217	−1.47079	C	0.7697	1.09214	−0.36792
C	−5.13906	4.45887	0.15613	C	1.6056	0.34872	0.4758
C	−4.45119	3.27483	0.03473	H	1.18146	1.65049	−1.19863
H	−4.80616	5.23575	0.83368	C	1.0904	−0.35218	1.54831
C	−4.88844	2.2687	−0.84023	H	2.66995	0.33359	0.28416
C	−6.01866	2.44463	−1.60522	C	−0.28348	−0.34138	1.82381
H	−6.35579	1.68612	−2.29925	H	1.75305	−0.91374	2.19275
H	−7.61521	3.7935	−2.07059	H	−0.67893	−0.88225	2.6737
N	−3.31163	2.81862	0.65183	C	−9.2246	4.72188	0.05276
N	−4.00894	1.21812	−0.7414	C	−8.81527	3.79231	1.00956
C	−2.43052	3.64279	1.42408	C	−9.572	2.66934	1.2752
H	−2.98178	4.13384	2.22757	H	−7.89386	3.95334	1.5522
H	−1.9354	4.41771	0.82888	C	−10.76118	2.44922	0.58002
H	−1.65973	3.02934	1.88777	H	−9.24644	1.95785	2.02161
C	−2.8507	1.5825	0.04068	C	−11.18335	3.37014	−0.37451
C	−3.96296	0.11455	−1.65385	C	−10.41888	4.4947	−0.62858
H	−3.65049	0.40511	−2.66194	H	−12.10304	3.20084	−0.9177
H	−4.94489	−0.3552	−1.7209	H	−10.74364	5.19859	−1.38423
H	−3.2648	−0.63153	−1.28014	C	−11.54117	1.28367	0.84556
N	−1.63481	1.70608	−0.73865	N	−12.16971	0.34238	1.05918
N	−2.46836	0.56983	0.99146	C	−7.06041	5.90854	−0.48491
C	−1.4873	2.71946	−1.73976	C	−8.43698	5.94545	−0.21273
H	−1.0307	3.63892	−1.35718	C	−9.08914	7.17679	−0.15719
H	−2.45996	2.97378	−2.15795	C	−6.38508	7.11491	−0.6673
H	−0.86853	2.35083	−2.55936	C	−7.04105	8.33181	−0.59776
C	−3.32331	0.23154	2.08961	C	−8.40378	8.36412	−0.34624
H	−3.19555	−0.81945	2.35142	H	−5.32725	7.08817	−0.89695
H	−4.36126	0.37285	1.79507	H	−6.49121	9.25121	−0.75024
H	−3.13305	0.8338	2.98404	H	−8.92971	9.30768	−0.28825
C	−1.10348	0.37892	0.98631	H	−10.1472	7.20119	0.07263

Table S11: Coordinates of the calculated structure of **1-ms-BN** (B3LYP−D3/def2−TZVP).

	x	y	z		x	y	z
C	−6.2091	4.64832	−0.46822	H	0.8534	2.42643	−1.45782
C	−6.59956	3.69549	−1.39602	C	2.15903	0.57817	1.04474
C	−5.06405	4.42975	0.32026	H	2.99226	1.74218	−0.52382
C	−4.3707	3.25196	0.16471	C	0.97367	0.12094	1.66024
H	−4.75592	5.17259	1.04579	H	3.11618	0.28361	1.45473
C	−4.77864	2.29388	−0.77629	C	−9.13066	4.68034	0.14942
C	−5.88405	2.50636	−1.56765	C	−8.72379	3.70837	1.06432
H	−6.19641	1.78238	−2.30866	C	−9.47379	2.5679	1.26666
H	−7.46938	3.87637	−2.01307	H	−7.81006	3.8502	1.62497
N	−3.26031	2.75997	0.80367	C	−10.65382	2.37268	0.54883
N	−3.89875	1.24232	−0.71032	H	−9.15046	1.82363	1.98146
C	−2.40314	3.52713	1.65755	C	−11.07407	3.33618	−0.36354
H	−2.99869	4.07133	2.39132	C	−10.31628	4.47768	−0.55438
H	−1.7881	4.25029	1.11155	H	−11.98697	3.18679	−0.92376
H	−1.73766	2.86659	2.21116	H	−10.63946	5.21523	−1.27791
C	−2.80437	1.51922	0.19031	C	−11.42577	1.18879	0.74859
C	−3.85982	0.14558	−1.62955	N	−12.04647	0.23196	0.90955
H	−3.56703	0.45061	−2.63951	C	−6.96946	5.90594	−0.30312
H	−4.83764	−0.33561	−1.68783	C	−8.3496	5.92082	−0.04821
H	−3.14601	−0.59517	−1.27599	C	−9.0109	7.1441	0.05576
N	−1.56599	1.67154	−0.54733	C	−6.29936	7.12298	−0.41998
N	−2.68392	0.4498	1.15526	C	−6.96476	8.33121	−0.30305
C	−1.59541	2.63701	−1.62242	C	−8.33089	8.34302	−0.06877
H	−1.15452	3.5967	−1.3333	H	−5.23811	7.11304	−0.63469
H	−2.61259	2.8249	−1.95014	H	−6.41939	9.26023	−0.40459
H	−1.04533	2.25687	−2.48424	H	−8.86388	9.2796	0.02612
C	−3.89089	0.14233	1.88954	H	−10.07193	7.15146	0.27258
H	−4.01089	−0.93782	1.98249	C	−1.48106	0.06889	1.71742
H	−4.76001	0.51951	1.36159	C	0.99822	−0.70669	2.80356
H	−3.88555	0.5765	2.89366	C	−0.17489	−1.11538	3.36944
C	−0.27474	0.50602	1.11718	H	1.95081	−1.0057	3.22085
C	−0.33007	1.33837	−0.02721	C	−1.41786	−0.73116	2.84349
C	0.8513	1.77928	−0.59423	H	−0.15964	−1.74657	4.24858
C	2.08502	1.38598	−0.05309	H	−2.31298	−1.07688	3.33718

Table S12: Coordinates of the calculated structure of **1-ms-DPOD** (B3LYP–D3/def2–TZVP).

	x	y	z		x	y	z
C	−7.91554	−2.76258	−1.58514	C	−14.061	2.02901	−0.20423
C	−8.5826	−3.09949	−2.75272	H	−13.41917	2.69282	−0.763
C	−9.95898	−2.82993	−2.87428	H	−17.02684	2.1689	1.45558
C	−10.60495	−2.22161	−1.8237	H	−15.48435	3.58603	0.16334
C	−9.91726	−1.88002	−0.648	H	−6.8602	−2.98111	−1.49288
C	−8.57401	−2.14894	−0.51508	C	−6.15004	−1.9082	−4.00983
H	−8.03373	−1.89434	0.38719	C	−4.80373	−1.78983	−3.67623
H	−10.48354	−3.07637	−3.78944	C	−6.94627	−0.76224	−3.96434
N	−10.8152	−1.29116	0.20102	C	−6.41624	0.4555	−3.59501
N	−11.908	−1.82845	−1.66847	C	−5.06729	0.56245	−3.2518
C	−12.14377	−1.21249	−0.37602	H	−7.04162	1.33762	−3.56906
C	−10.51544	−0.7867	1.50519	C	−4.26343	−0.57241	−3.29759
H	−11.41595	−0.36782	1.95017	H	−3.21577	−0.51708	−3.03406
H	−9.76173	0.00428	1.46807	H	−4.17188	−2.66901	−3.69194
H	−10.15102	−1.57554	2.16862	H	−7.99347	−0.82897	−4.22661
C	−12.94639	−2.00022	−2.63596	C	−4.54369	1.85484	−2.85679
H	−13.87981	−1.59965	−2.24513	O	−3.24622	1.98778	−2.52551
H	−13.10865	−3.05554	−2.87179	C	−3.13644	3.29397	−2.22328
H	−12.72171	−1.47326	−3.56723	N	−4.26267	3.90473	−2.35701
N	−12.5698	0.15868	−0.51962	N	−5.16982	2.97799	−2.76495
N	−13.09567	−1.97099	0.3998	C	−1.8588	3.84286	−1.80789
C	−12.76681	−3.36082	0.60998	C	−1.77572	5.20435	−1.51582
H	−11.82127	−3.61059	0.14009	C	−0.57156	5.75407	−1.11674
H	−13.53034	−4.01718	0.18499	H	−2.66012	5.82066	−1.6052
H	−12.67575	−3.58867	1.67481	C	0.55921	4.95575	−1.0046
C	−11.68532	1.01383	−1.27597	H	−0.51409	6.81064	−0.89217
H	−10.81327	0.46526	−1.61634	C	0.47917	3.60221	−1.29435
H	−11.33081	1.85104	−0.66999	H	1.5002	5.3886	−0.69222
H	−12.18591	1.41931	−2.15862	C	−0.7234	3.04331	−1.69508
C	−13.7287	0.69975	−0.01135	H	−0.77212	1.98601	−1.91791
C	−14.60614	−0.12738	0.73045	H	1.35714	2.97616	−1.20854
C	−15.80506	0.39946	1.26554	C	−7.87099	−3.75117	−3.8732
C	−16.11132	1.76027	1.04844	C	−8.36063	−4.95567	−4.37677
C	−15.25208	2.54214	0.33075	C	−7.71678	−5.62893	−5.40082

6. NMR Spectra

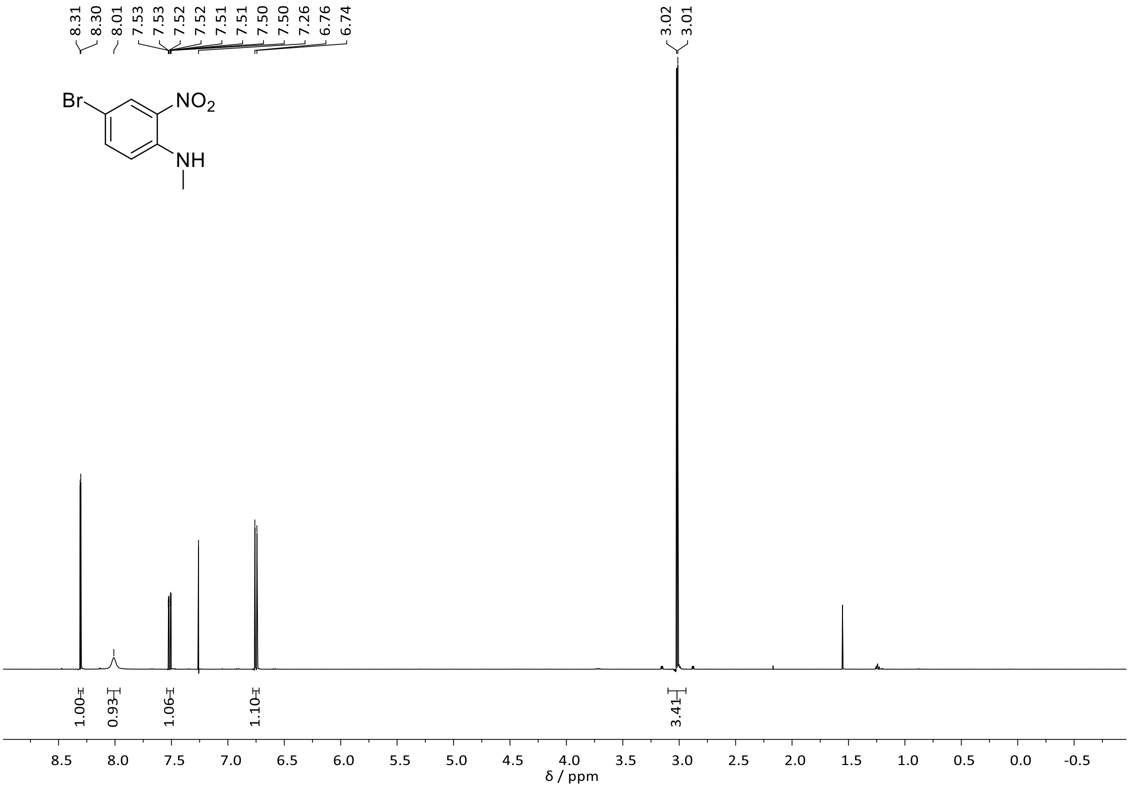

Figure S38: ^{1}H NMR spectrum (500 MHz) of **4a** in chloroform-*d*, contains traces of H_2O.

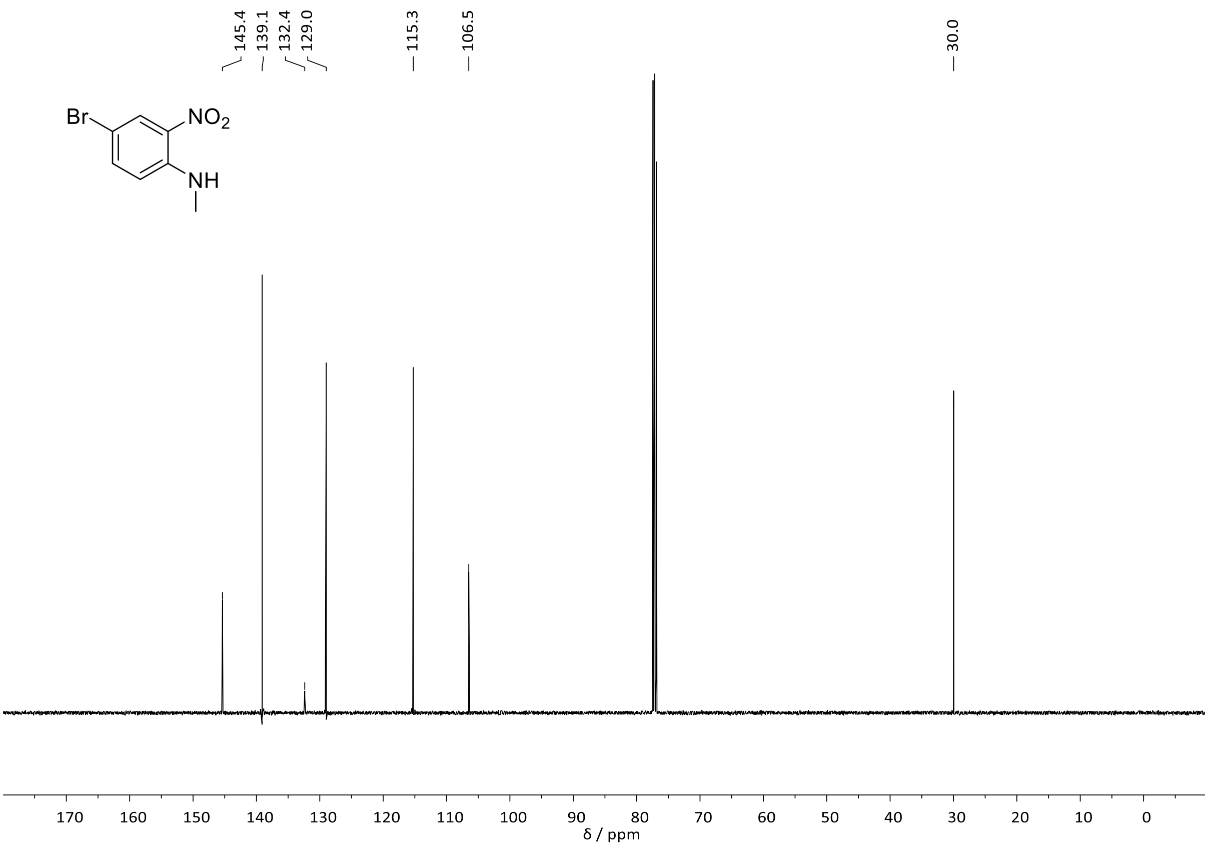

Figure S39: ^{13}C NMR spectrum (126 MHz) of **4a** in chloroform-*d*.

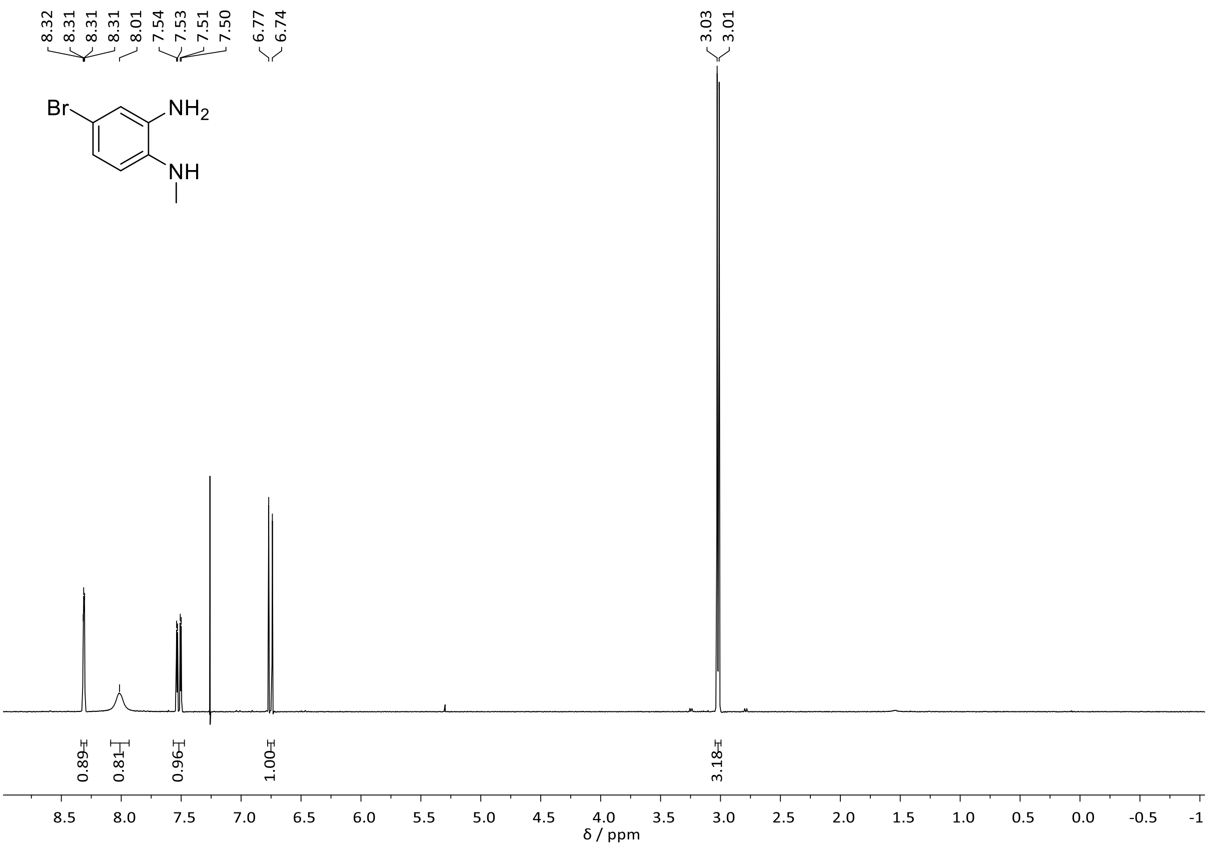

Figure S40: ^{1}H NMR spectrum (300 MHz) of **5a** in chloroform-*d*, contains traces of dichloromethane.

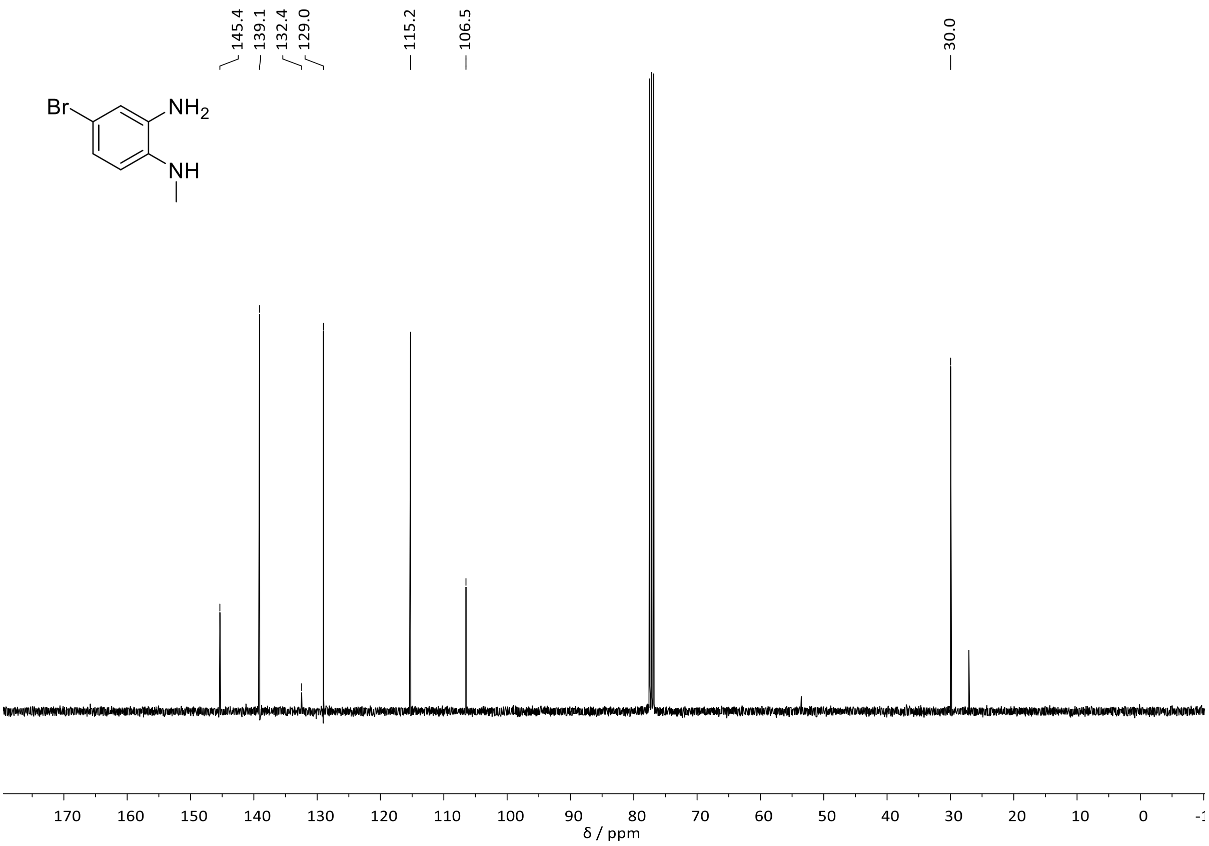

Figure S41: ^{13}C NMR spectrum (101 MHz) of **5a** in chloroform-*d*, contains traces of dichloromethane.

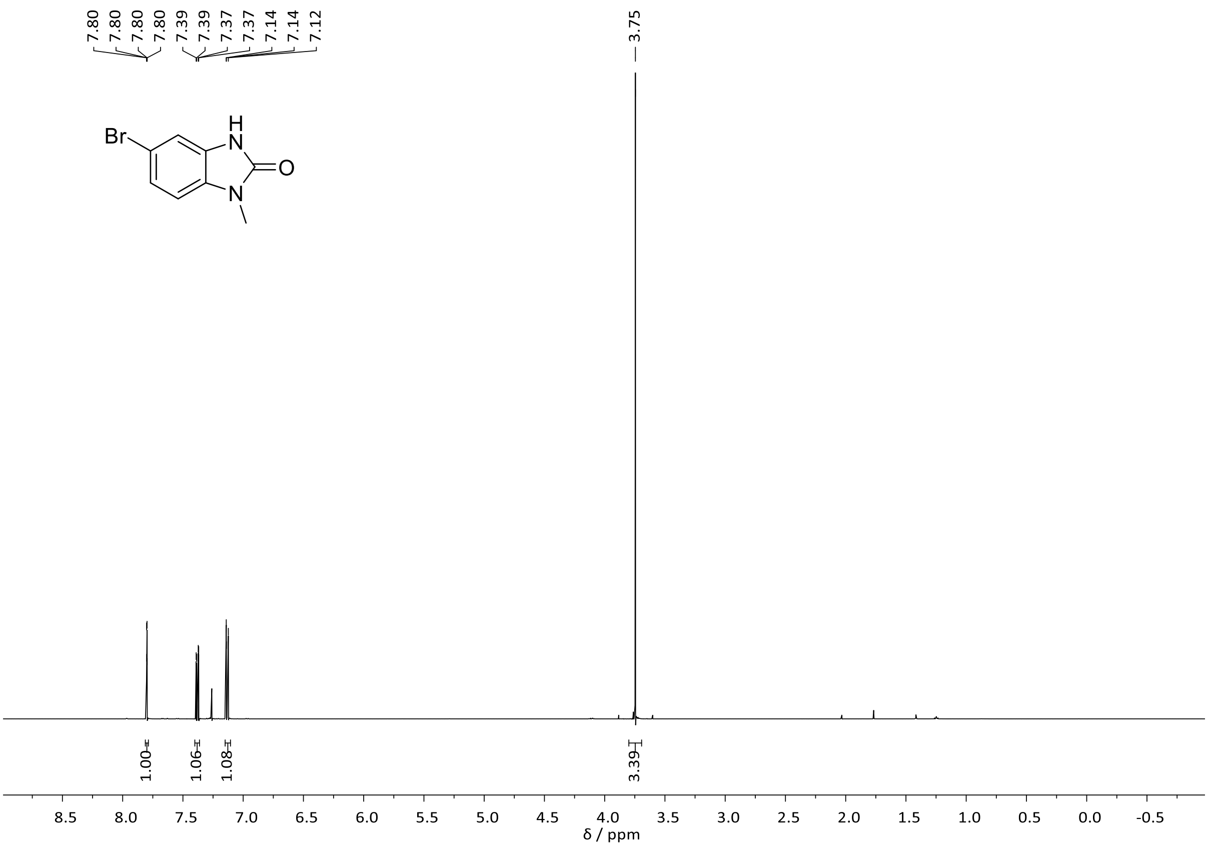

Figure S42: ^{1}H NMR spectrum (500 MHz) of **6a** in chloroform-*d*.

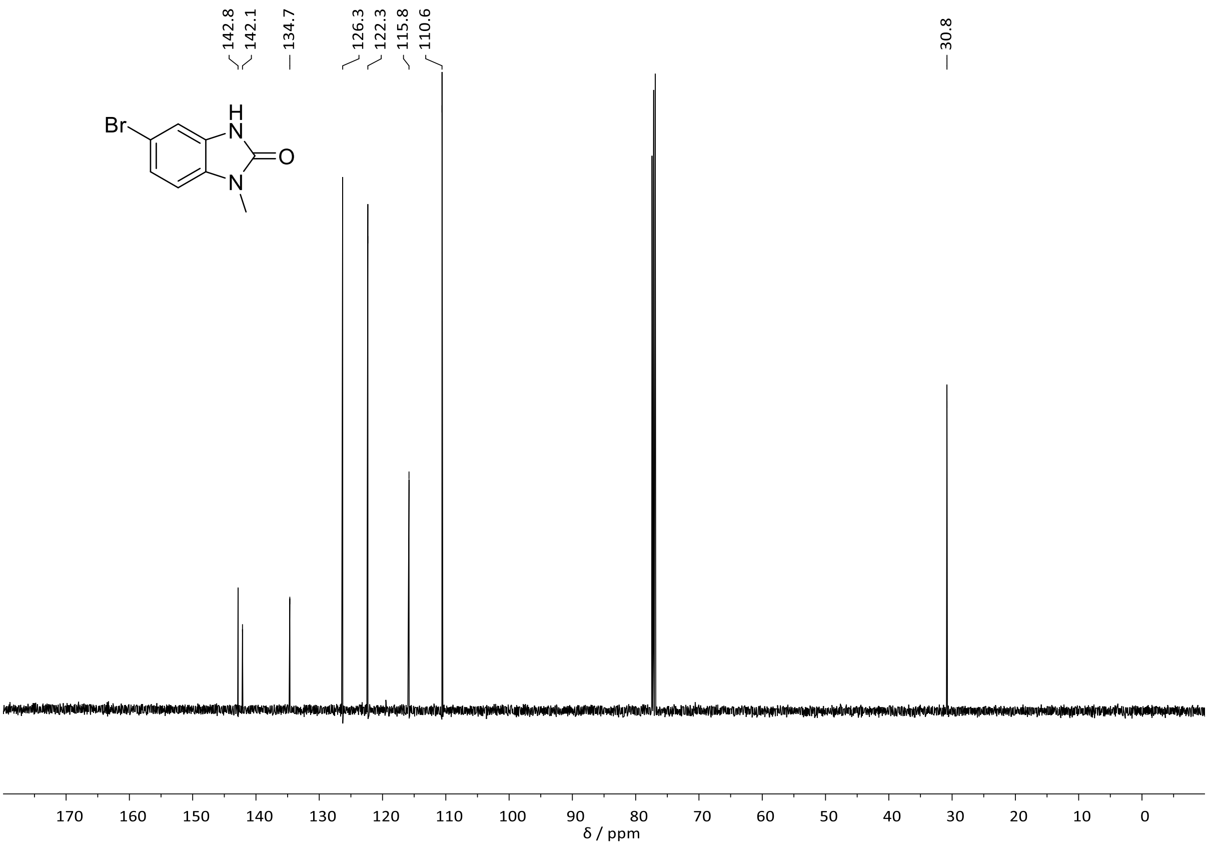

Figure S43: ^{13}C NMR spectrum (126 MHz) of **6a** in chloroform-*d*.

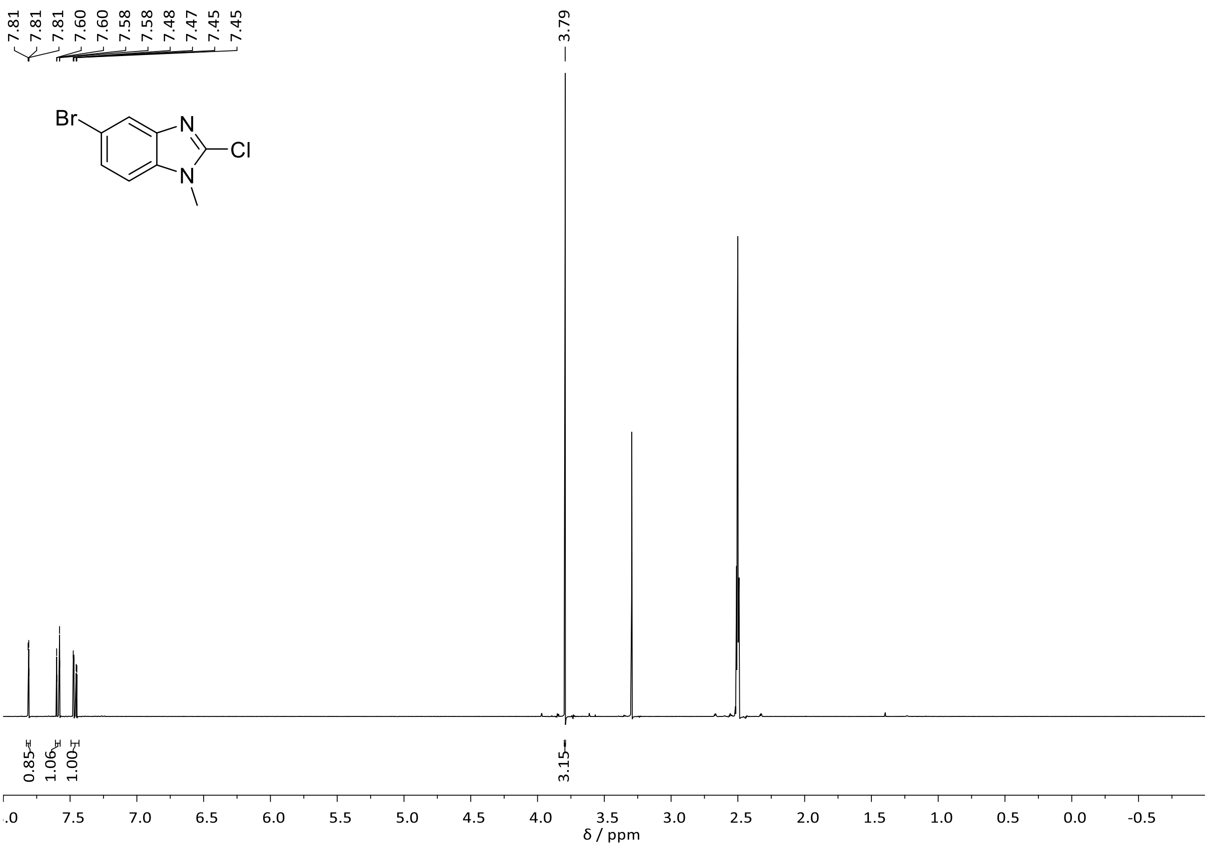

Figure S44: ^{1}H NMR spectrum (400 MHz, 303 K) of **7a** in DMSO-d_6, contains traces of H_2O.

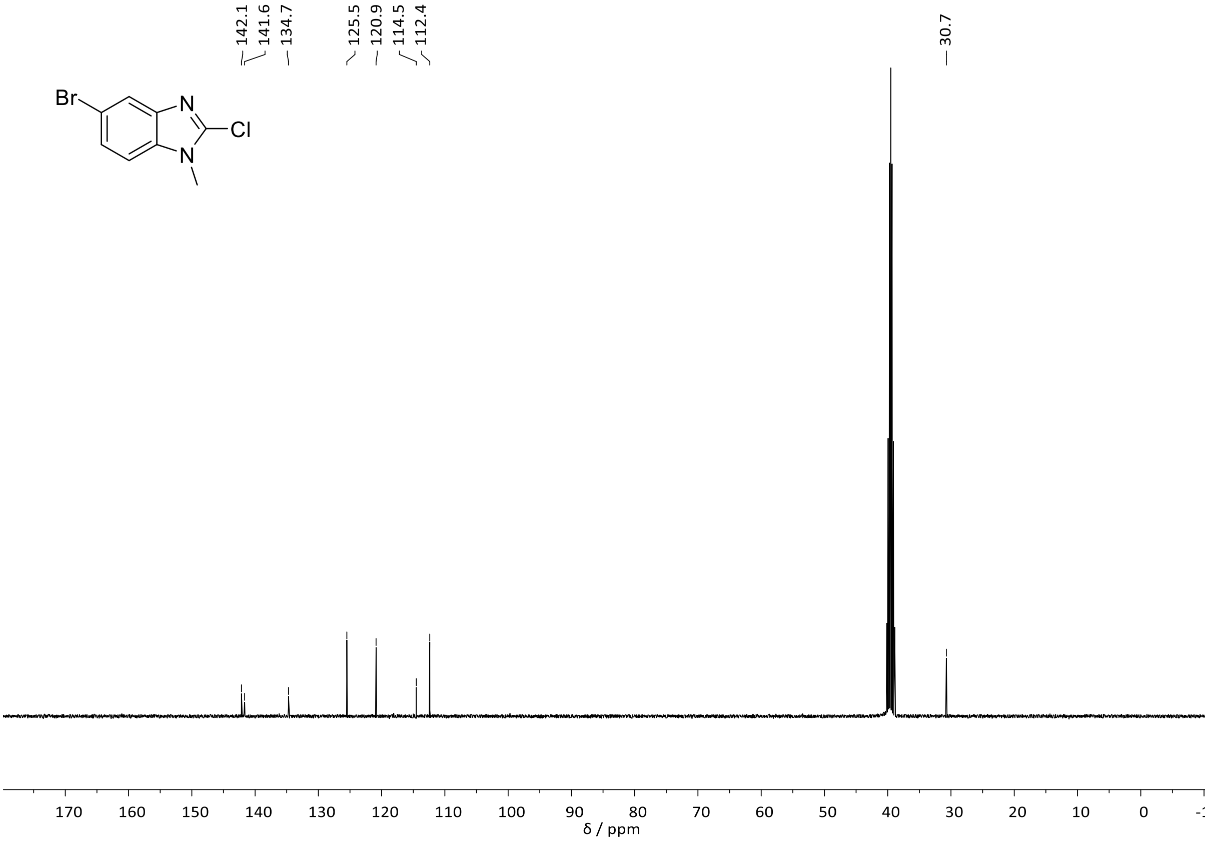

Figure S45: ^{13}C NMR spectrum (101 MHz, 303 K) of **7a** in DMSO-d_6.

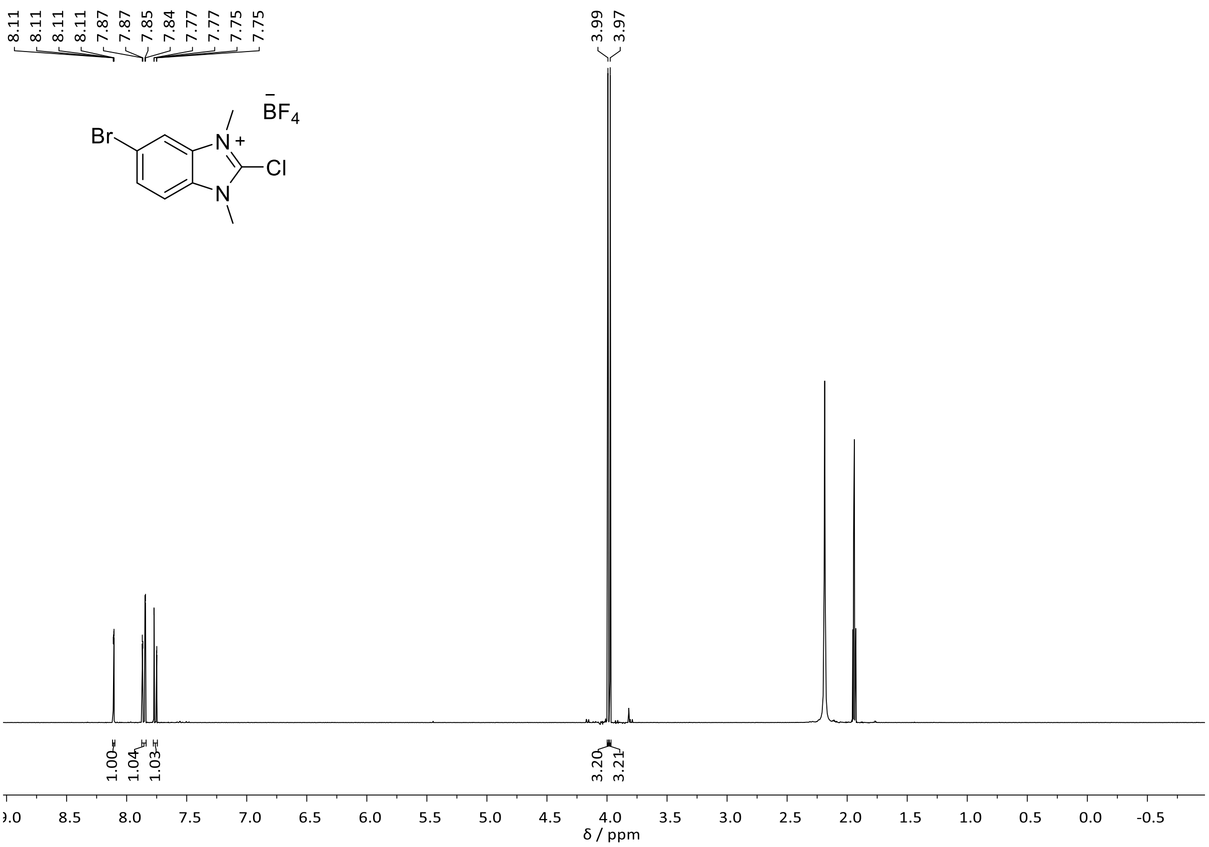

Figure S46: ^{1}H NMR spectrum (400 MHz, 303 K) of **3a** in acetonitrile-*d*$_3$, contains traces of H_2O.

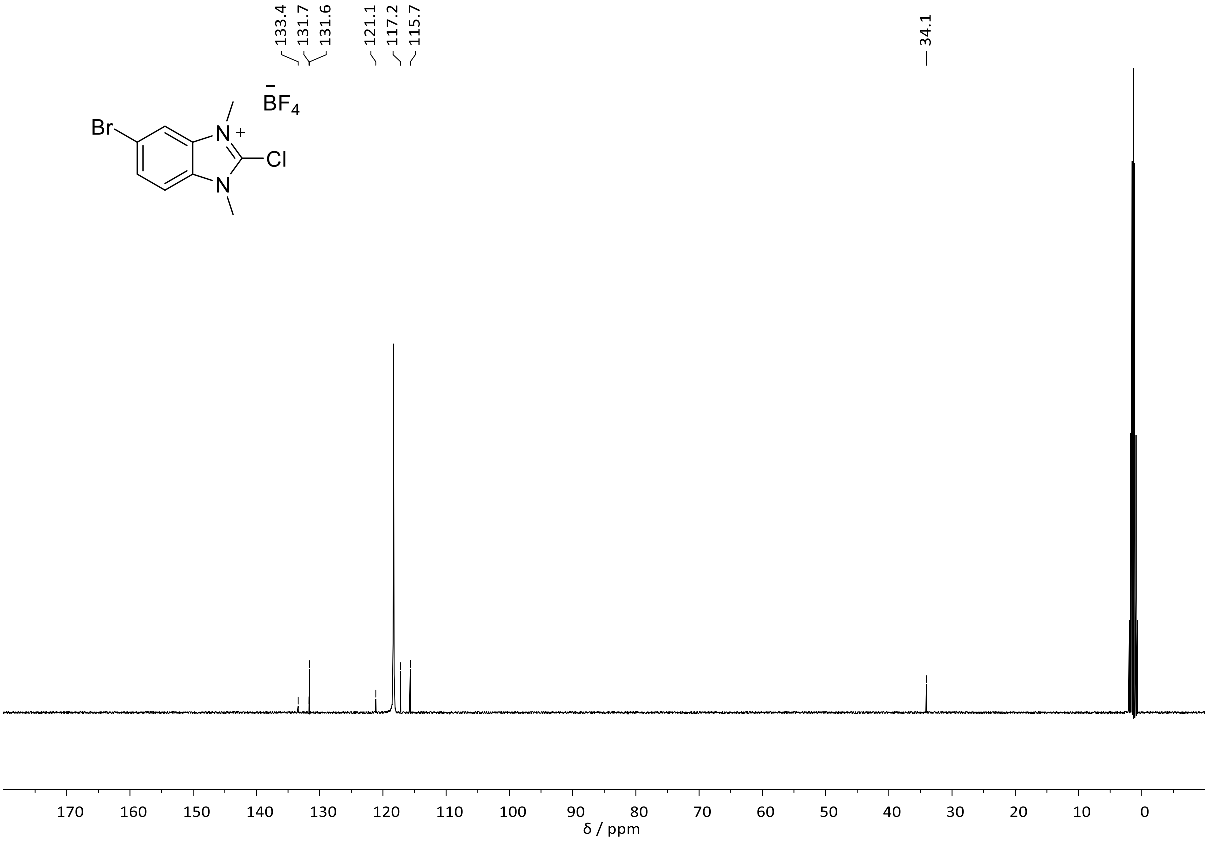

Figure S47: ^{13}C NMR spectrum (101 MHz, 303 K) of **3a** in acetonitrile-*d*$_3$.

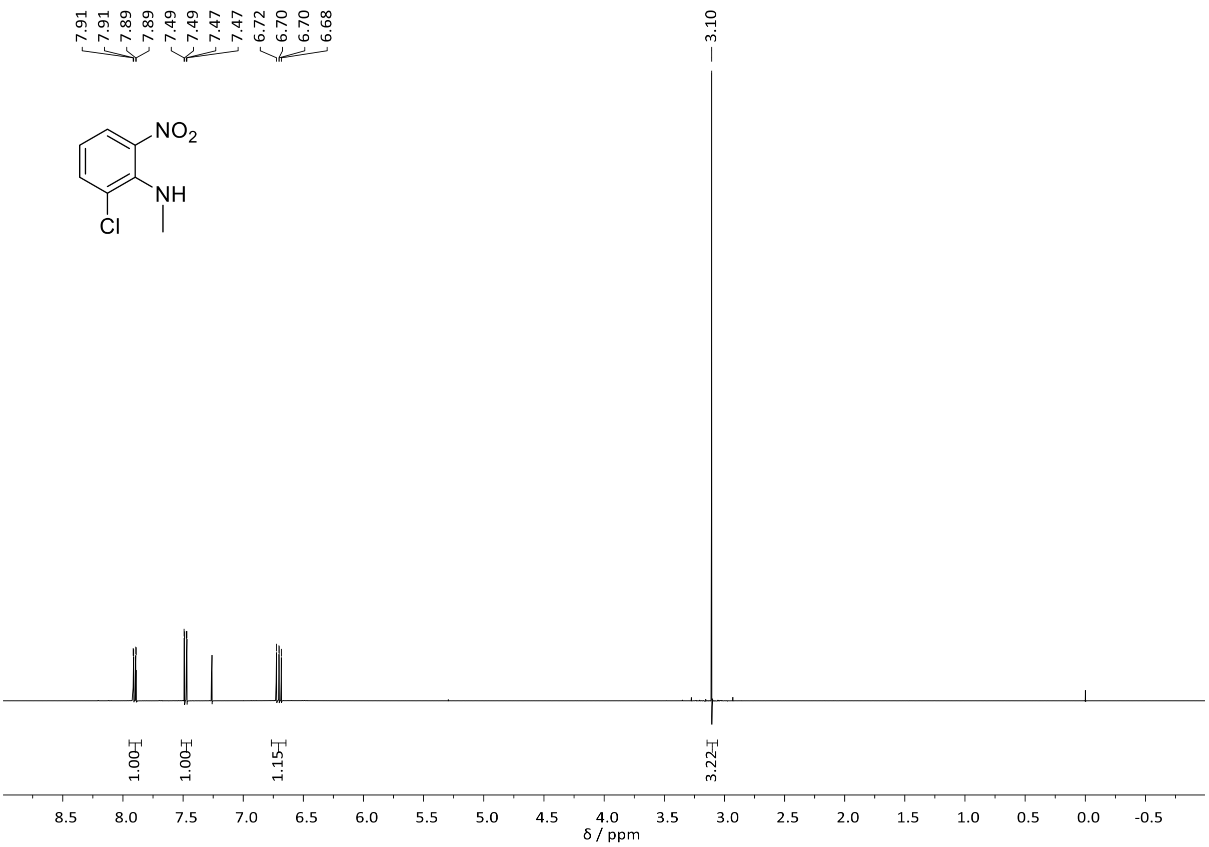

Figure S48: ^{1}H NMR spectrum (400 MHz, 303 K) of **4b** in chloroform-*d*.

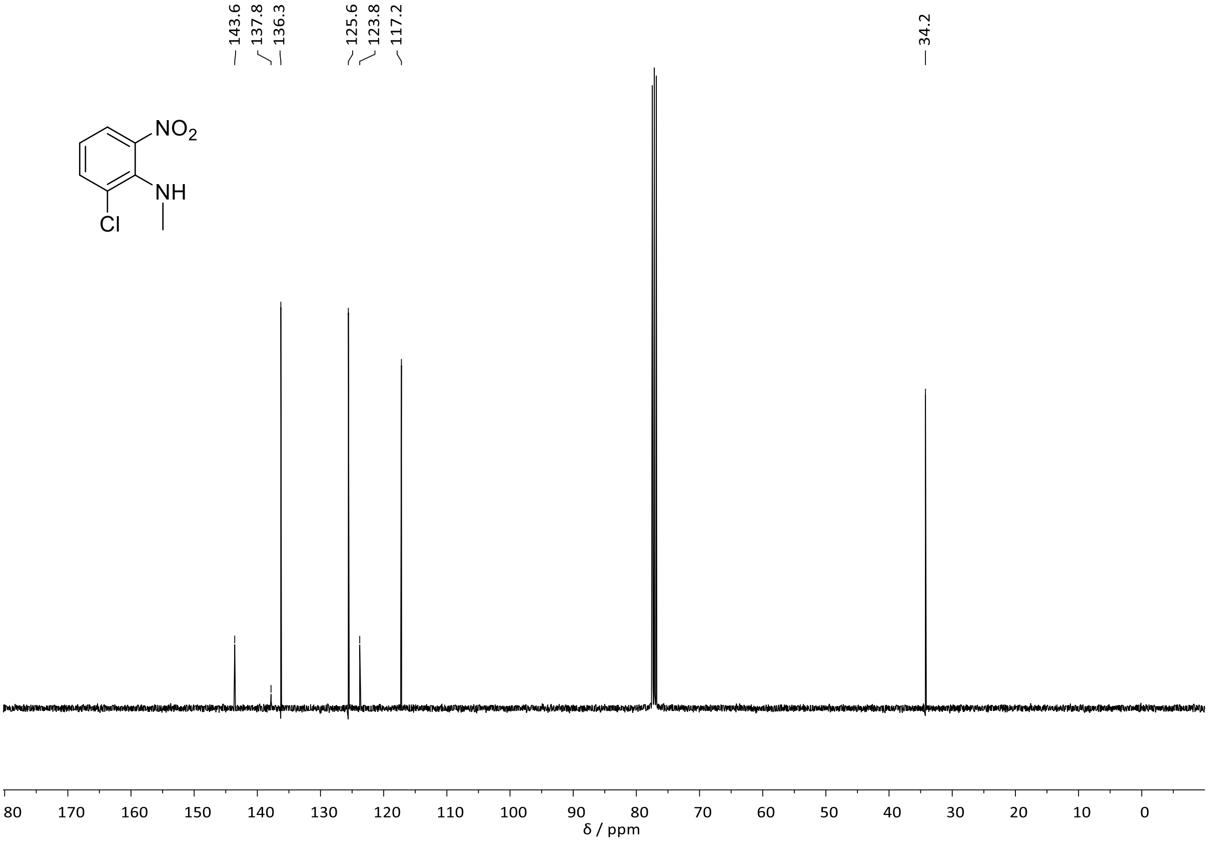

Figure S49: ^{13}C NMR spectrum (101 MHz, 303 K) of **4b** in chloroform-*d*.

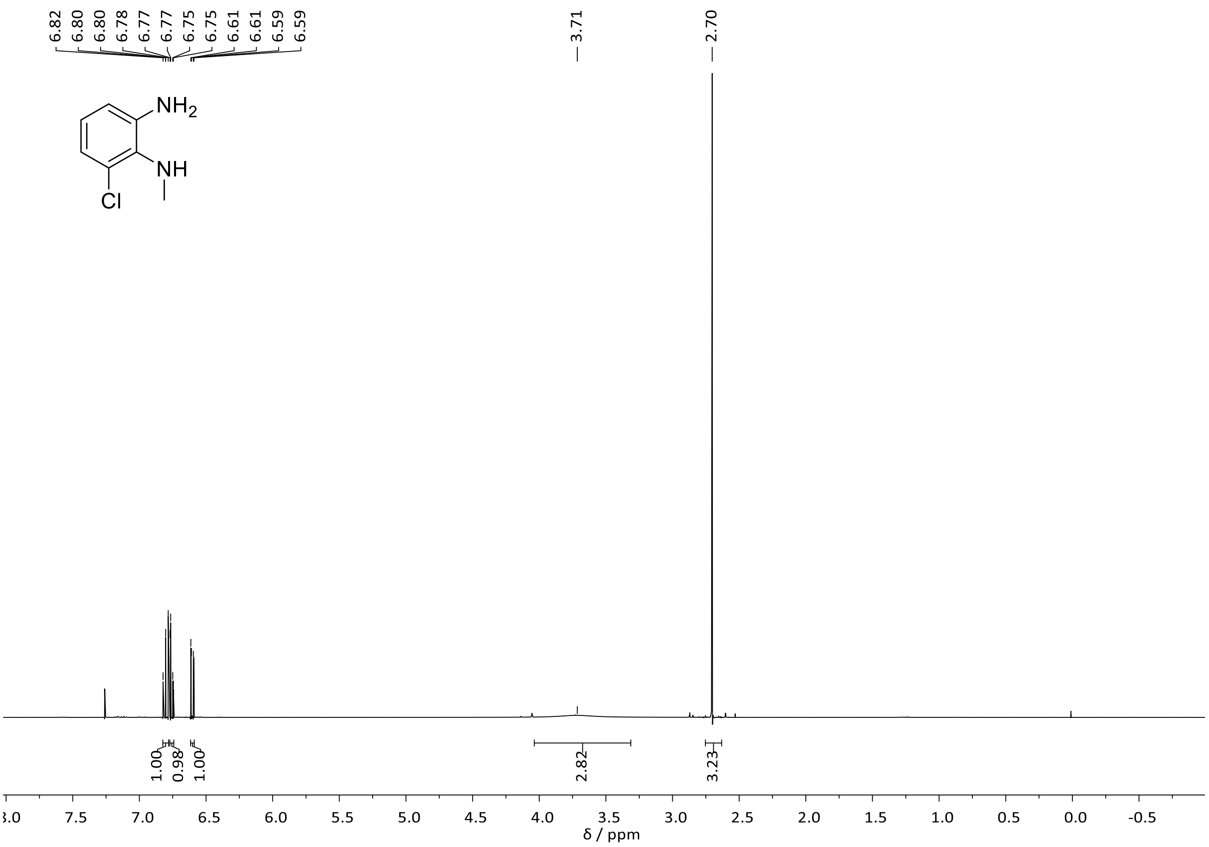

Figure S50: ^{1}H NMR spectrum (400 MHz, 303 K) of **5b** in chloroform-*d*.

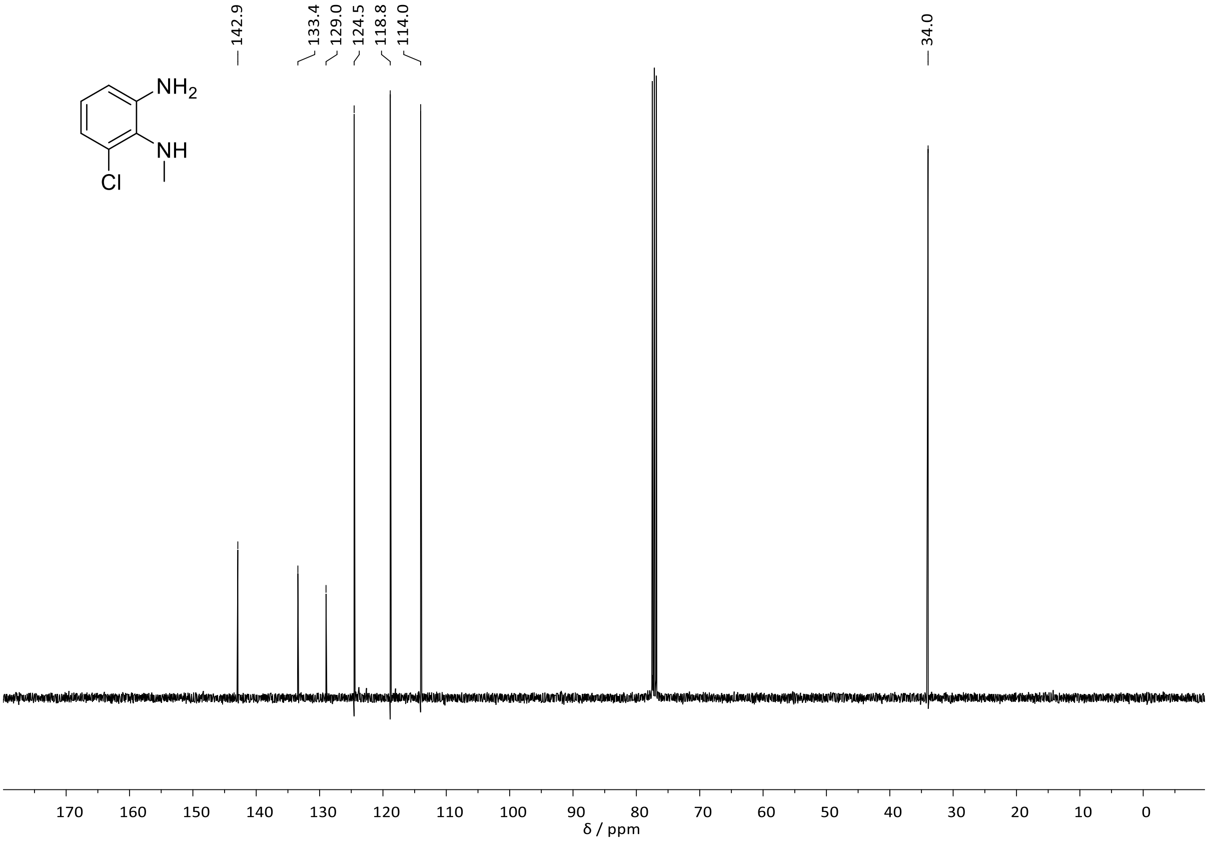

Figure S51: ^{13}C NMR spectrum (101 MHz, 303 K) of **5b** in chloroform-*d*.

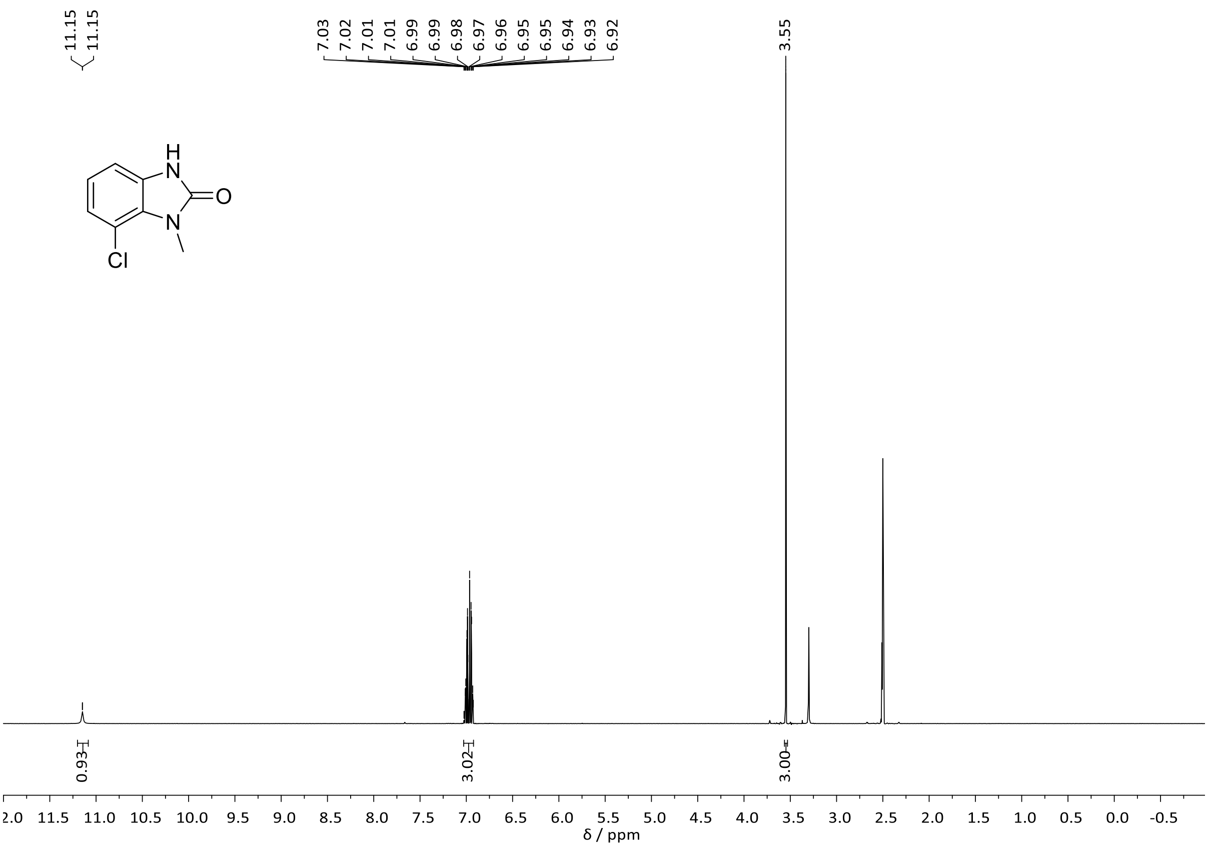

Figure S52: ^{1}H NMR spectrum (400 MHz, 303 K) of **6b** in DMSO-d_6, contains traces of H_2O.

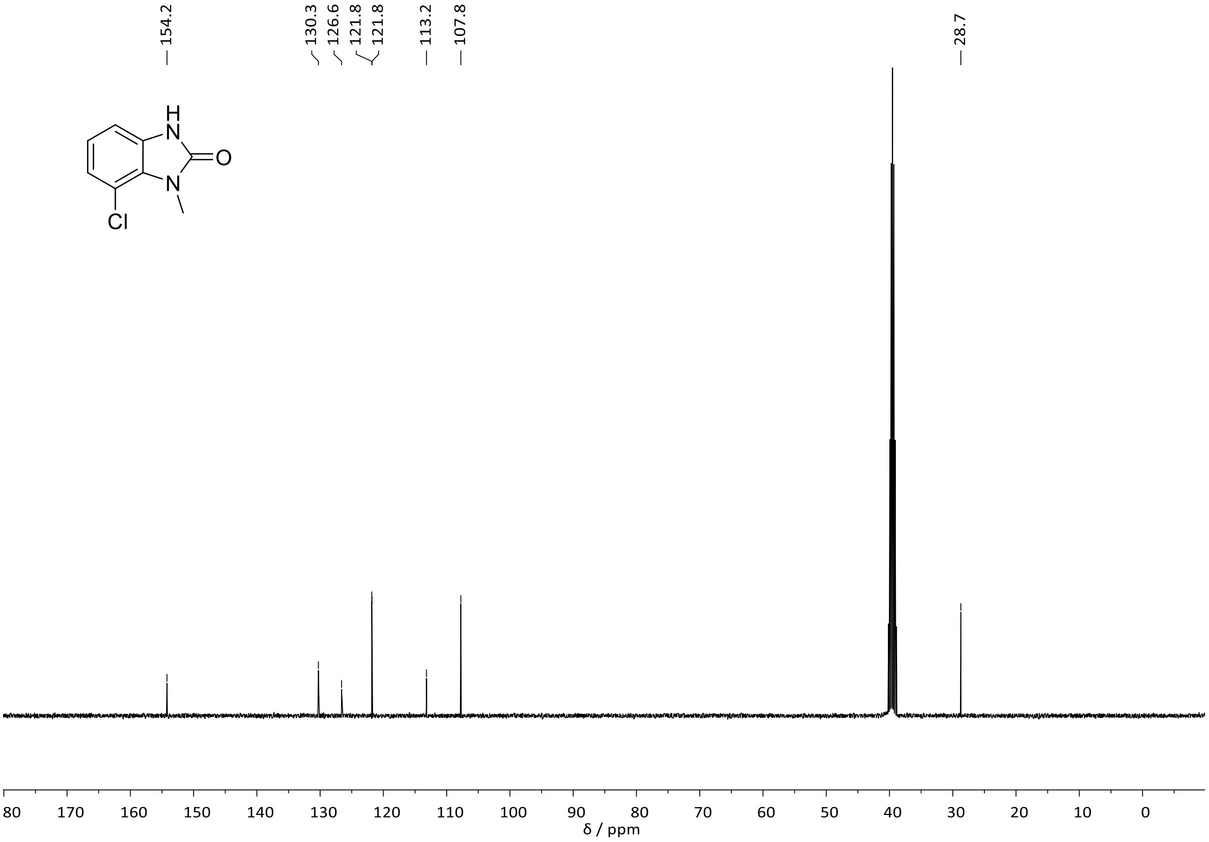

Figure S53: ^{13}C NMR spectrum (101 MHz, 303 K) of **6b** in DMSO-d_6.

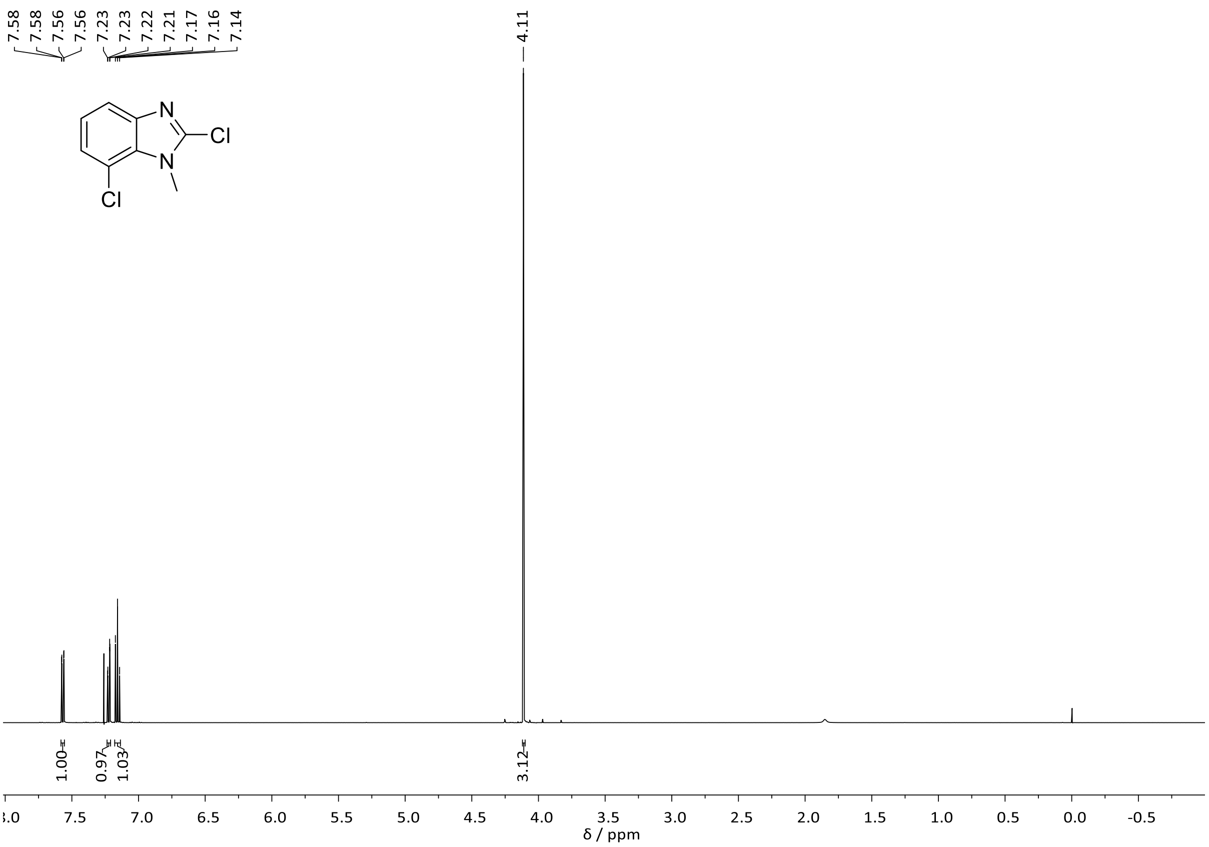

Figure S54: ^{1}H NMR spectrum (500 MHz) of **7b** in chloroform-*d*.

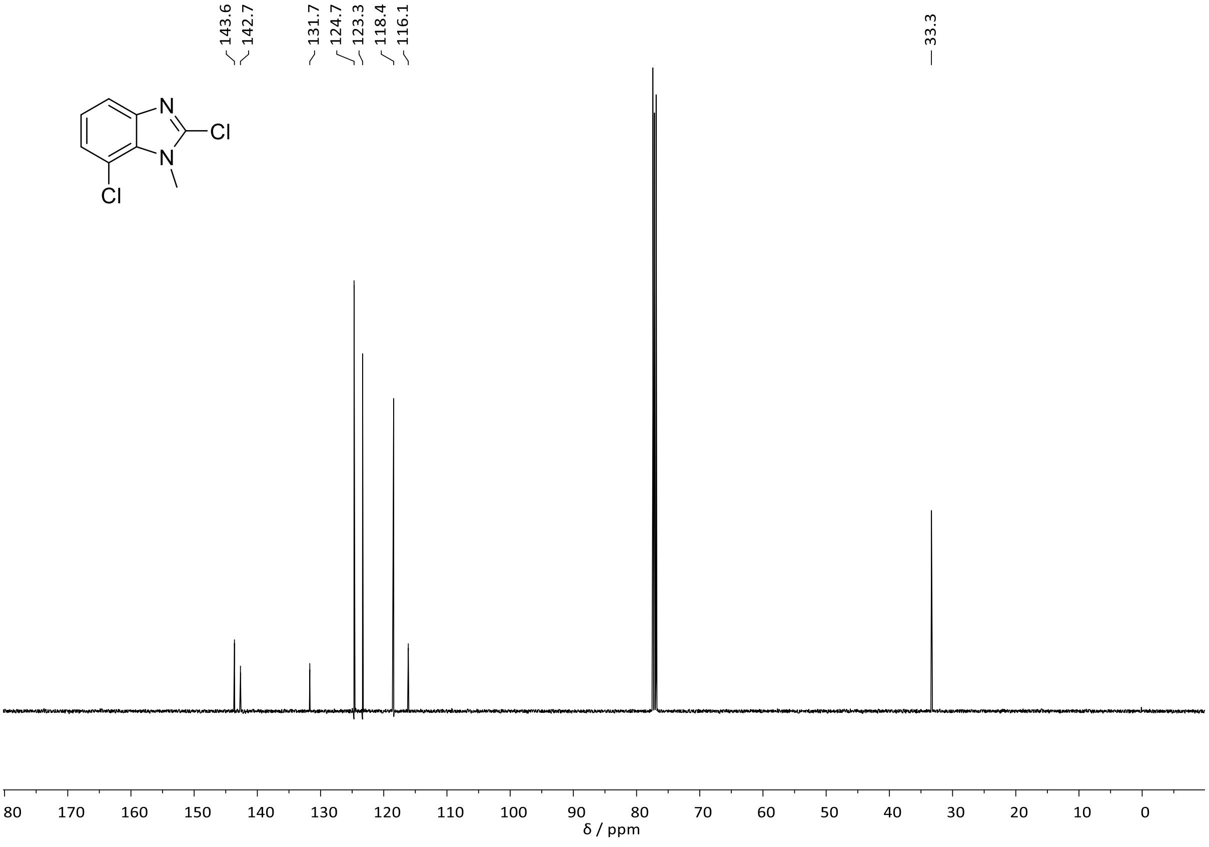

Figure S55: ^{13}C NMR spectrum (126 MHz) of **7b** in chloroform-*d*.

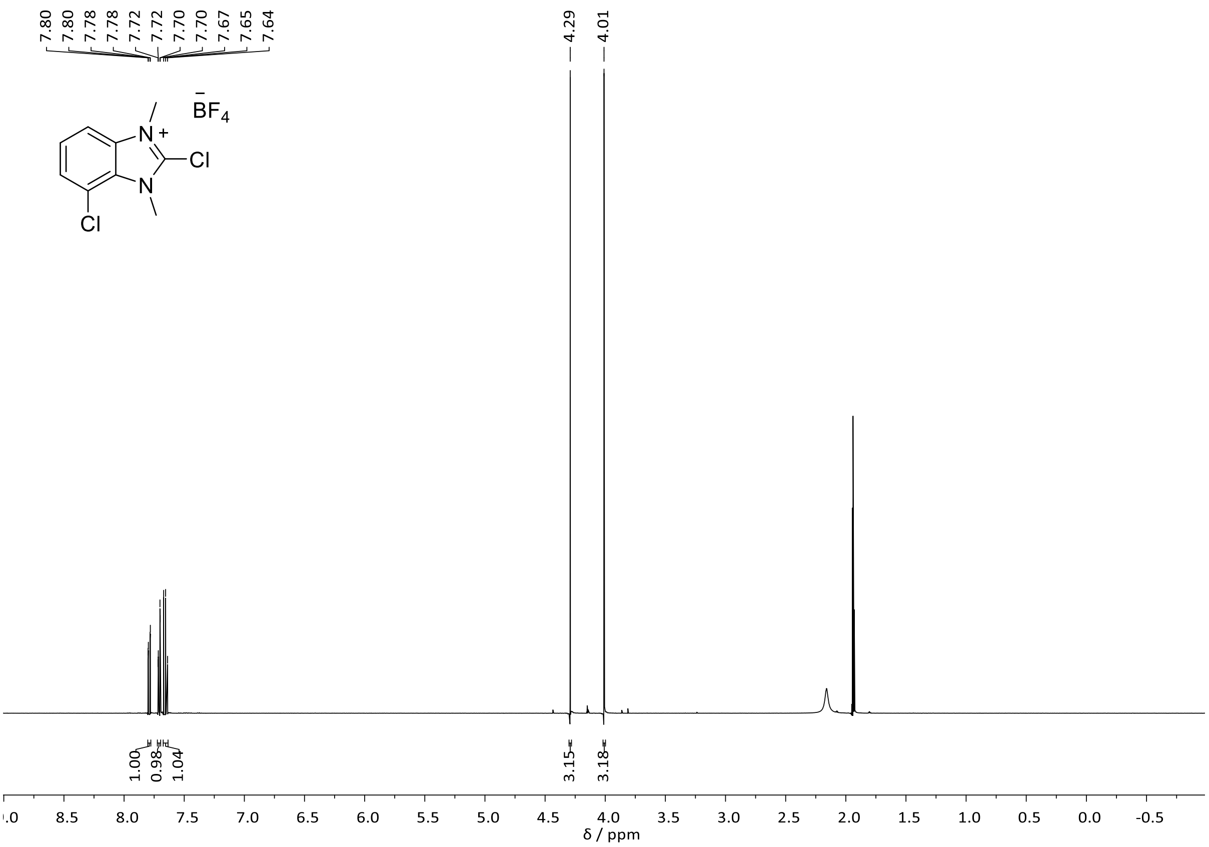

Figure S56: ^{1}H NMR spectrum (500 MHz) of **3b** in acetonitrile-*d*$_3$, contains traces of H_2O.

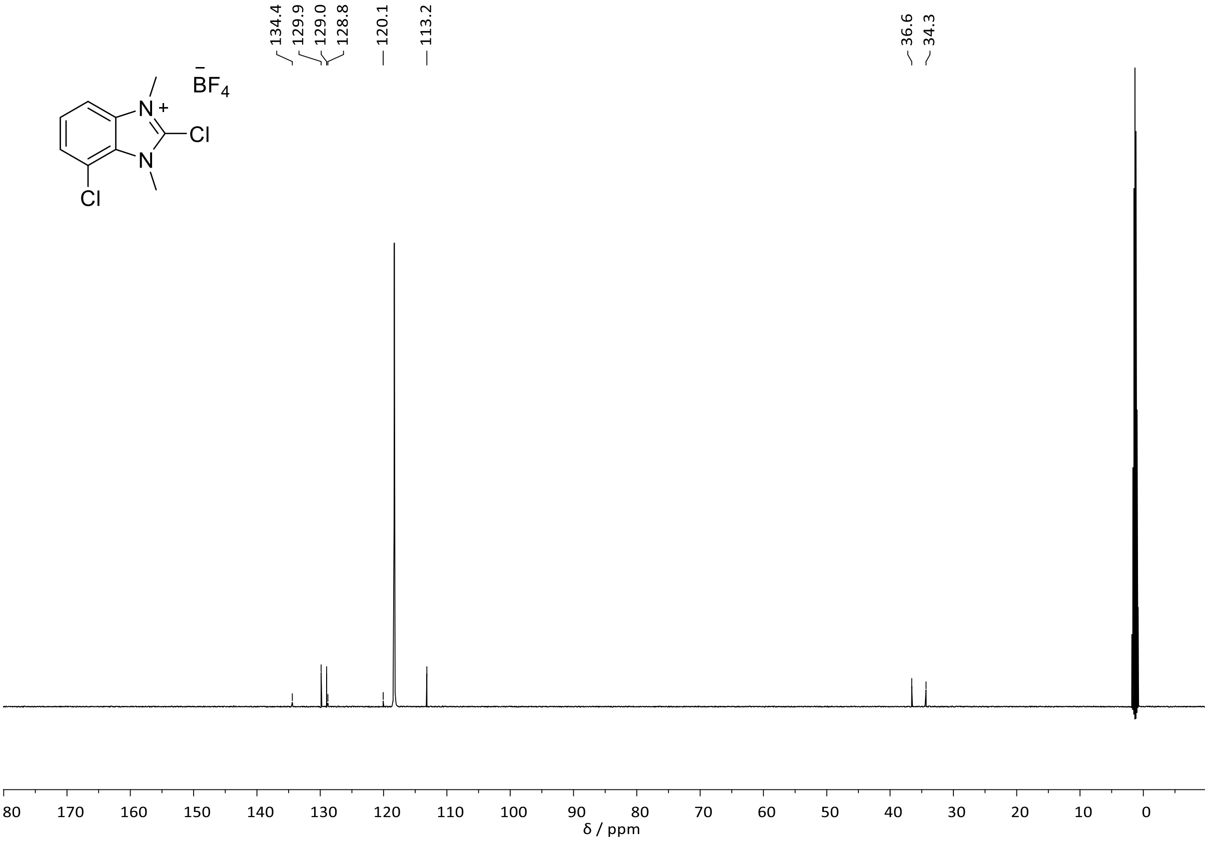

Figure S57: ^{13}C NMR spectrum (126 MHz) of **3b** in acetonitrile-*d*$_3$.

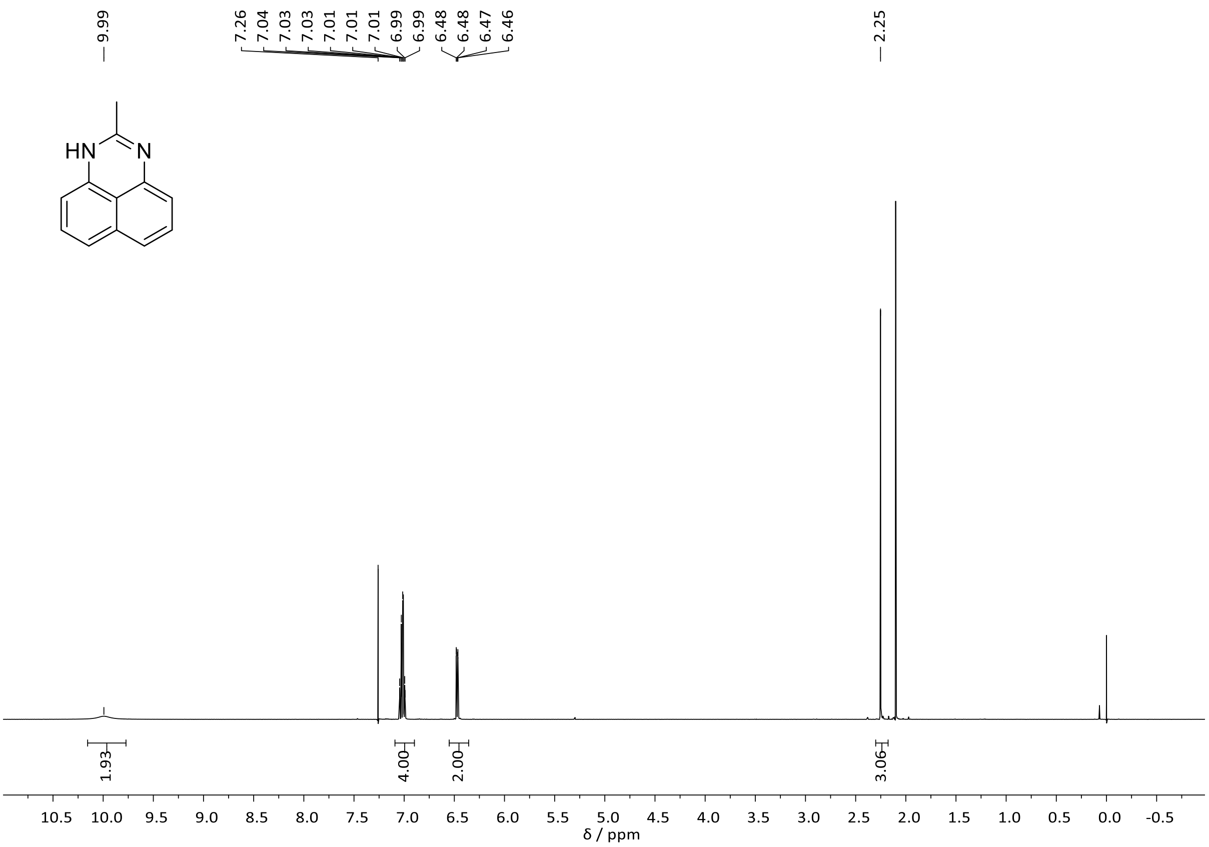

Figure S58: ^{1}H NMR spectrum (500 MHz) of **S3** in chloroform-*d*, contains traces of acetic acid and silion grease.

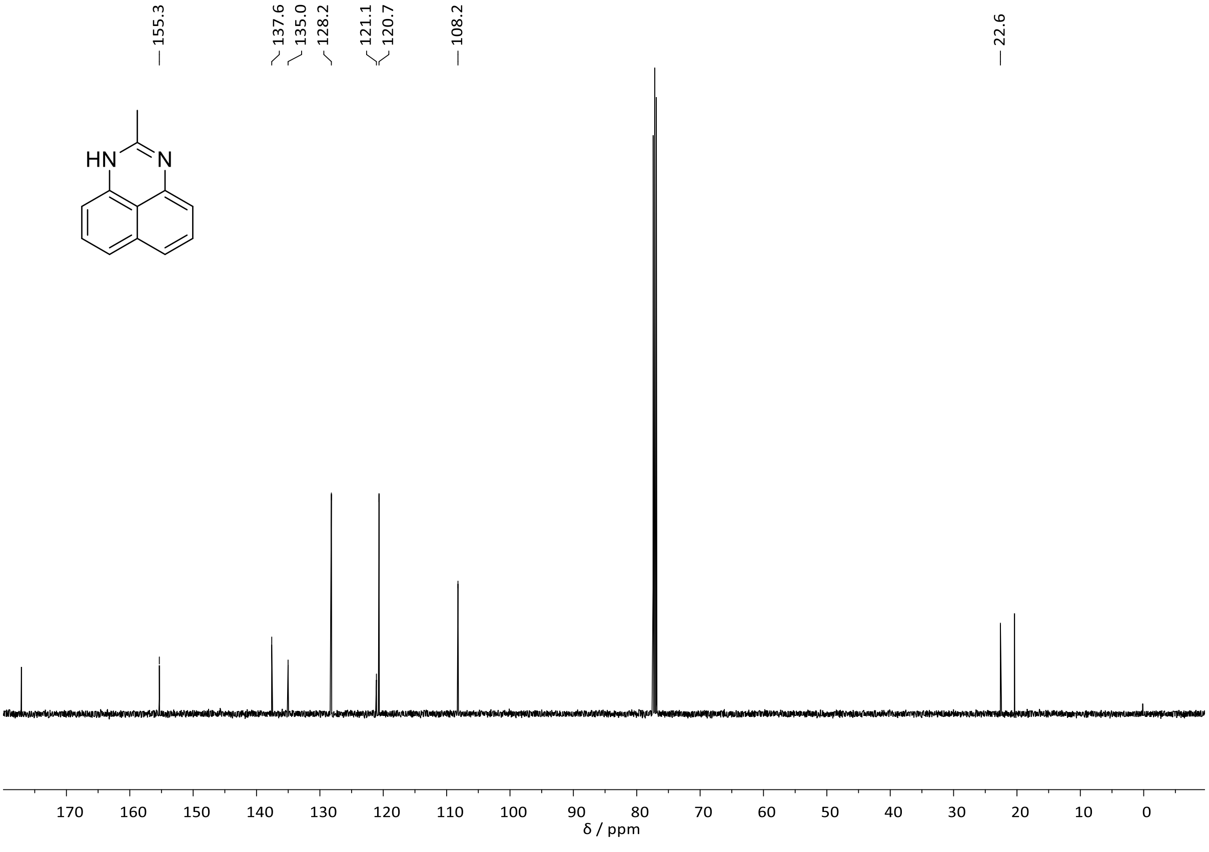

Figure S59: ^{13}C NMR spectrum (126 MHz) of **S3** in chloroform-*d*, contains traces of acetic acid and silicon grease.

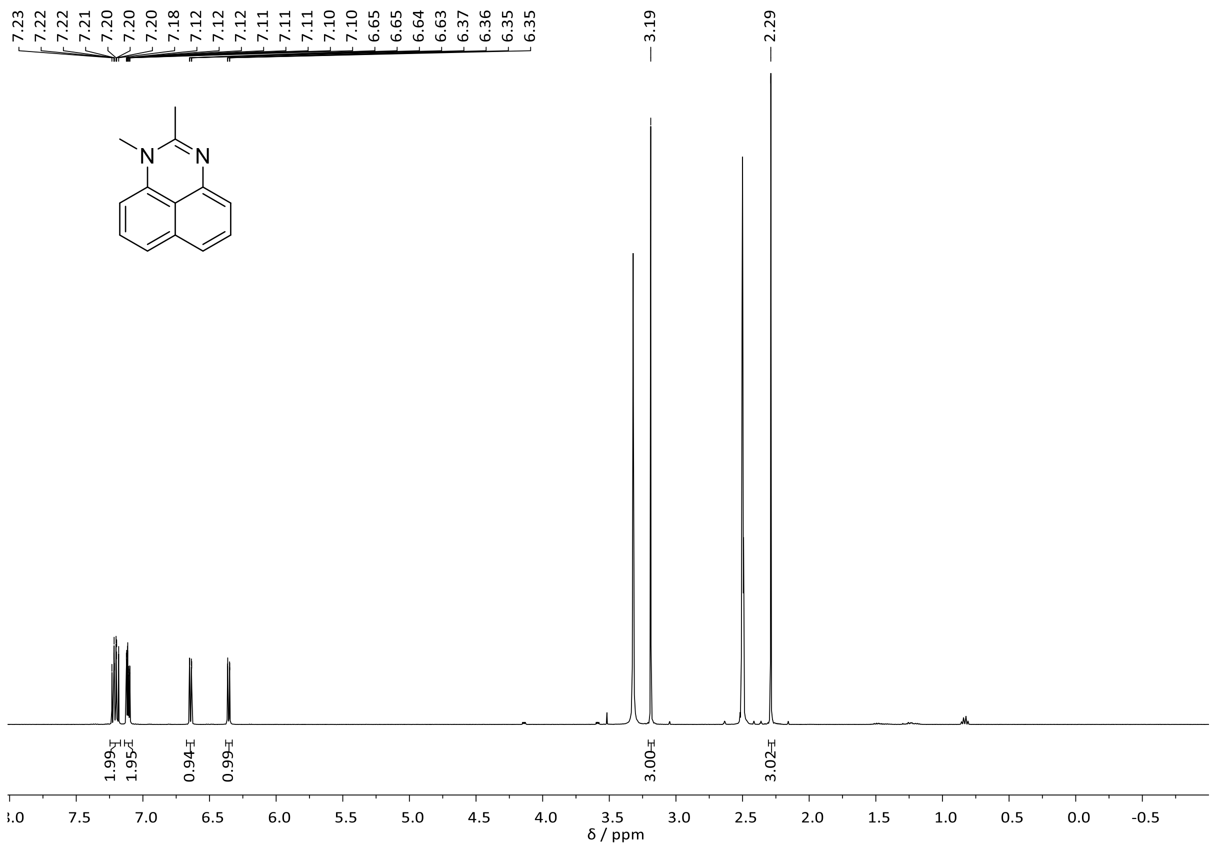

Figure S60: ^{1}H NMR spectrum (500 MHz) of **S4** in DMSO-*d*$_6$, contains traces of H_2O, grease and unknown impurity.

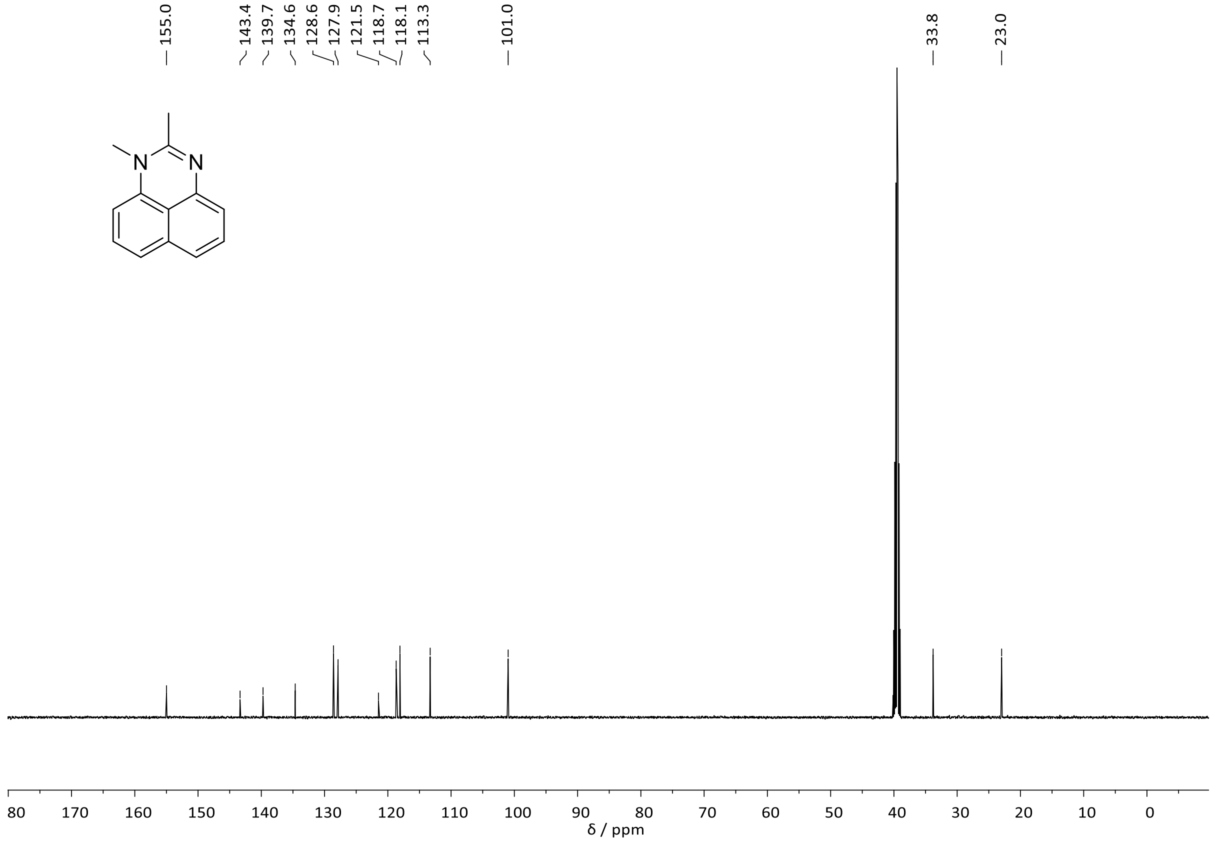

Figure S61: ^{13}C NMR spectrum (126 MHz) of **S4** in DMSO-*d*$_6$.

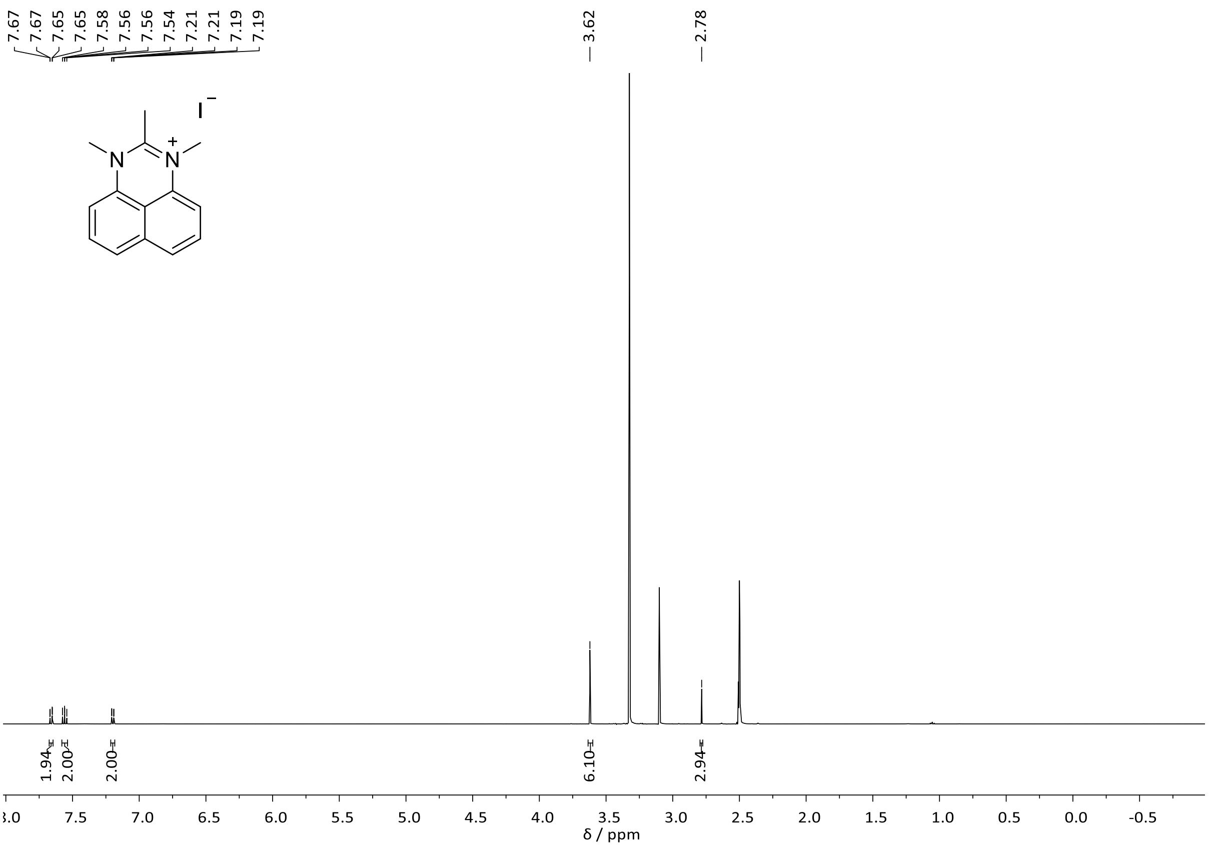

Figure S62: ^{1}H NMR spectrum (500 MHz) of **S5** in DMSO-d_6.

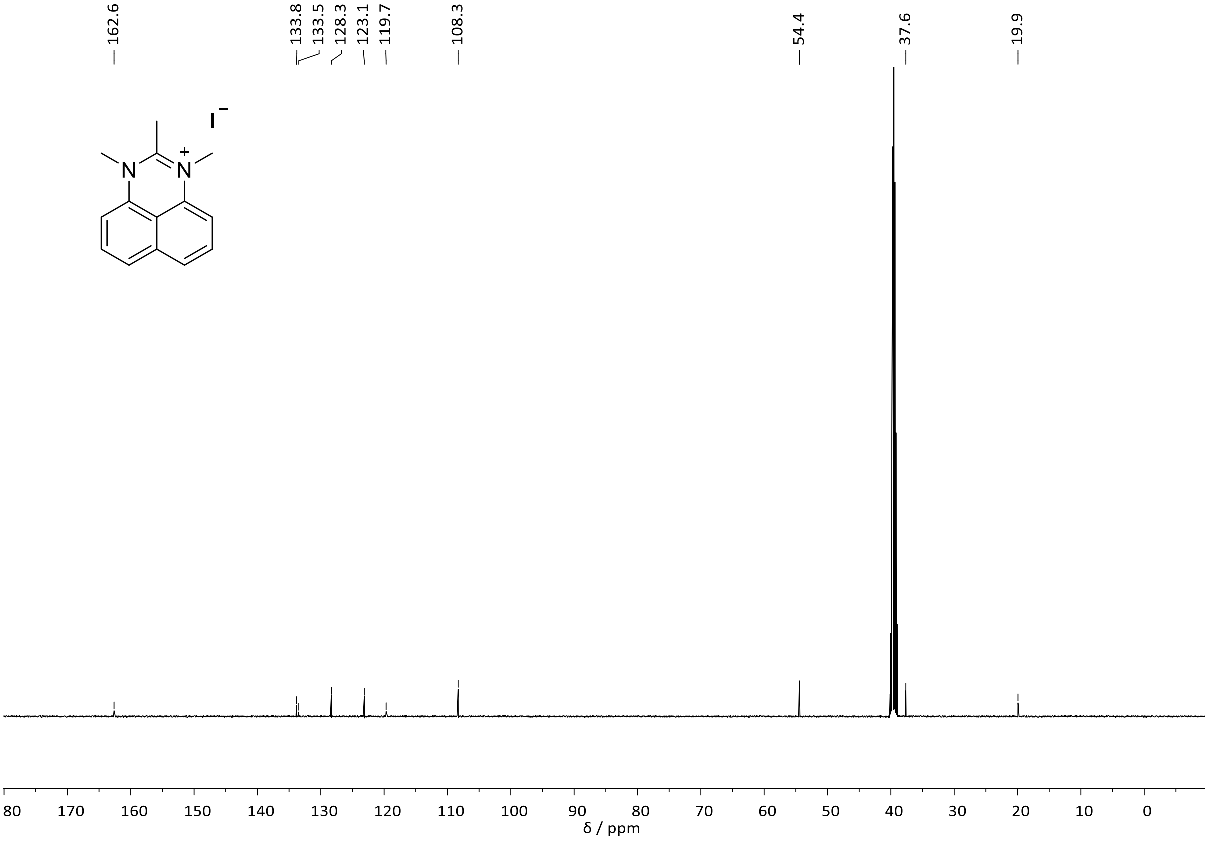

Figure S63: ^{13}C NMR spectrum (126 MHz) of **S5** in DMSO-d_6.

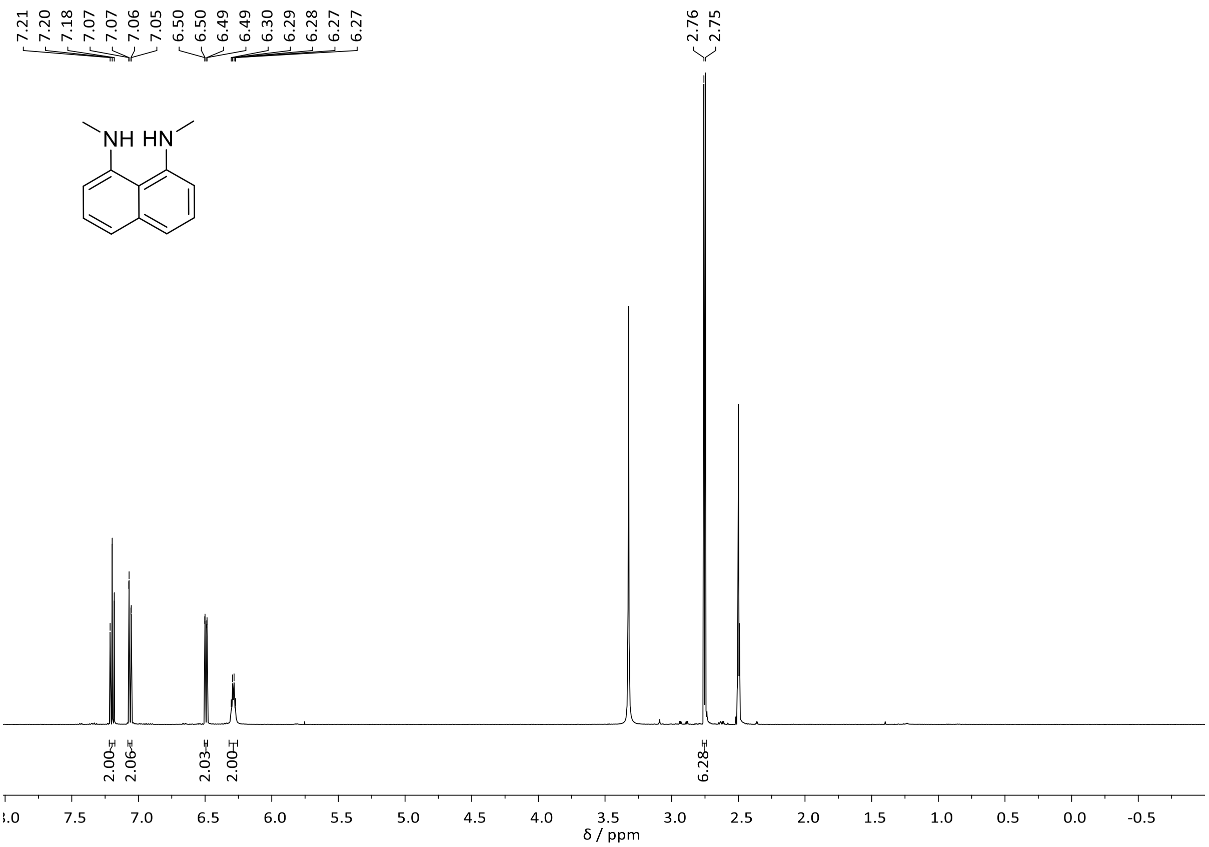

Figure S64: ^{1}H NMR spectrum (500 MHz) of **10** in DMSO-d_6, contains traces of H_2O.

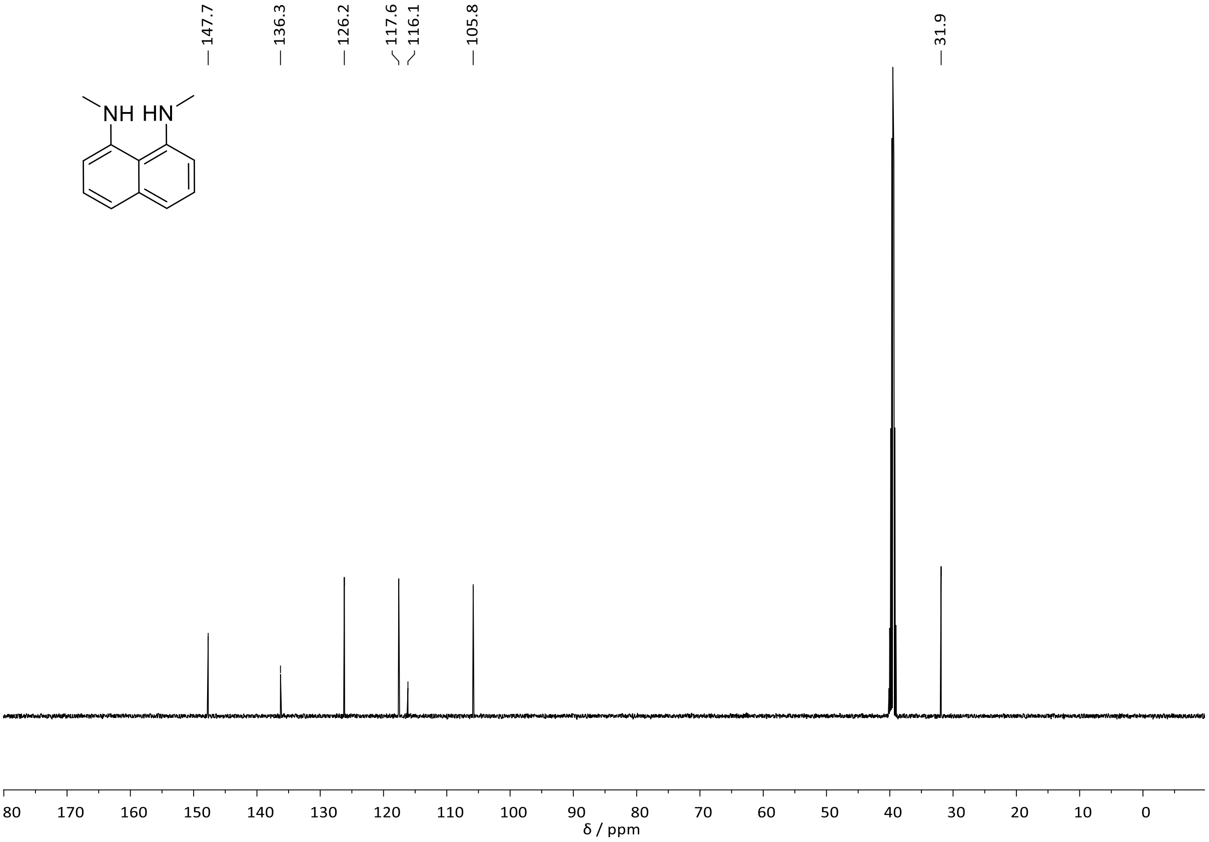

Figure S65: ^{13}C NMR spectrum (126 MHz) of **10** in DMSO-d_6.

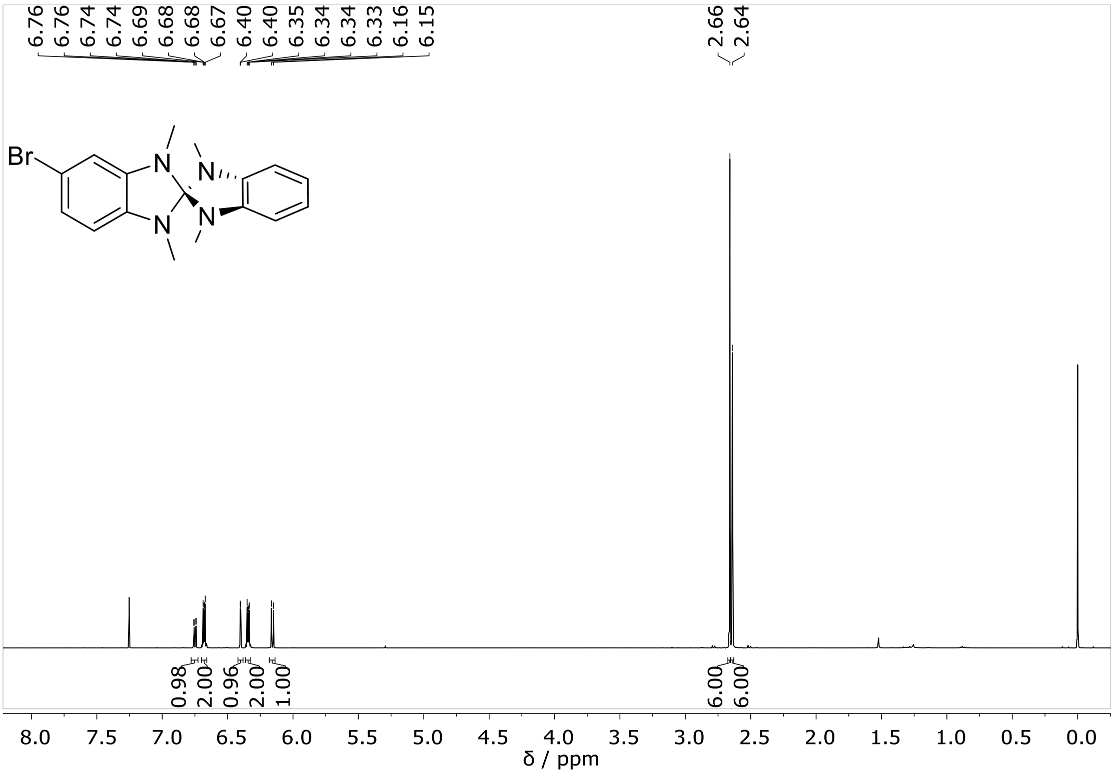

Figure S66: ^{1}H NMR spectrum (500 MHz) of **9** in chloroform-*d*.

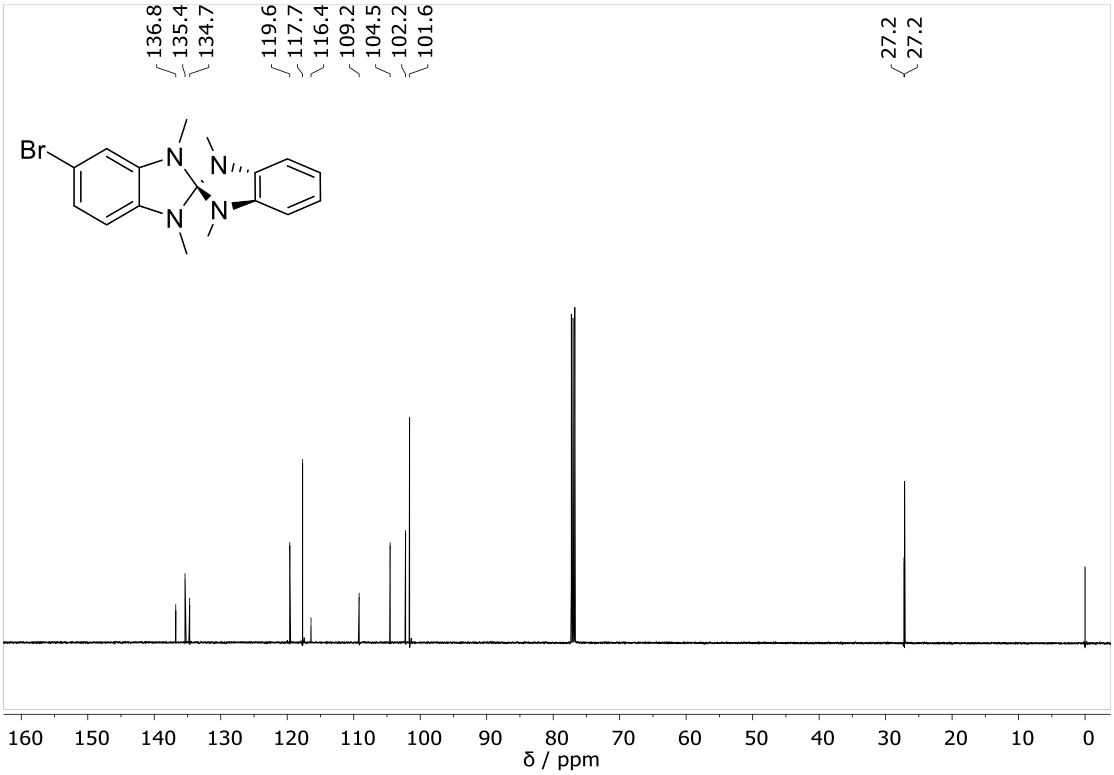

Figure S67: ^{13}C NMR spectrum (126 MHz) of **9** in chloroform-*d*.

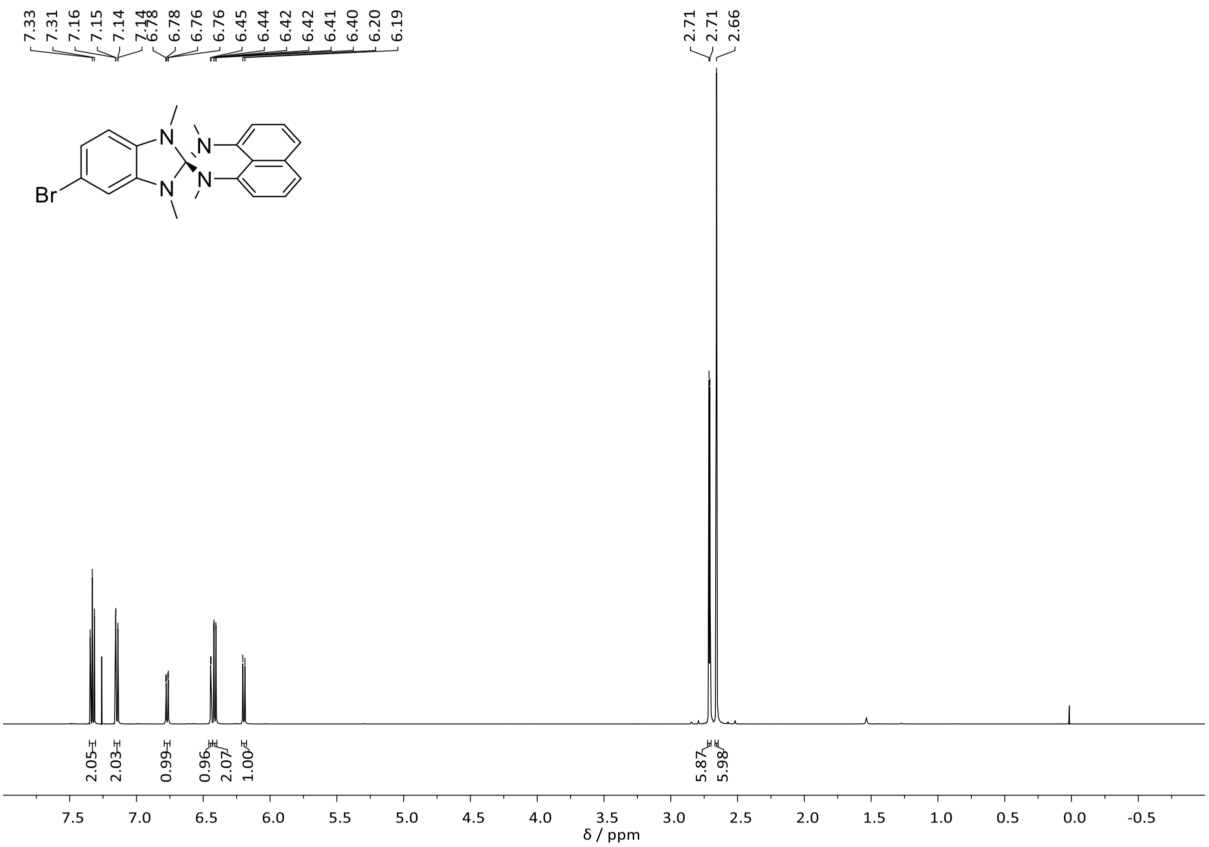

Figure S68: ^{1}H NMR spectrum (500 MHz) of **8a** in chloroform-*d*, contains traces of H_2O.

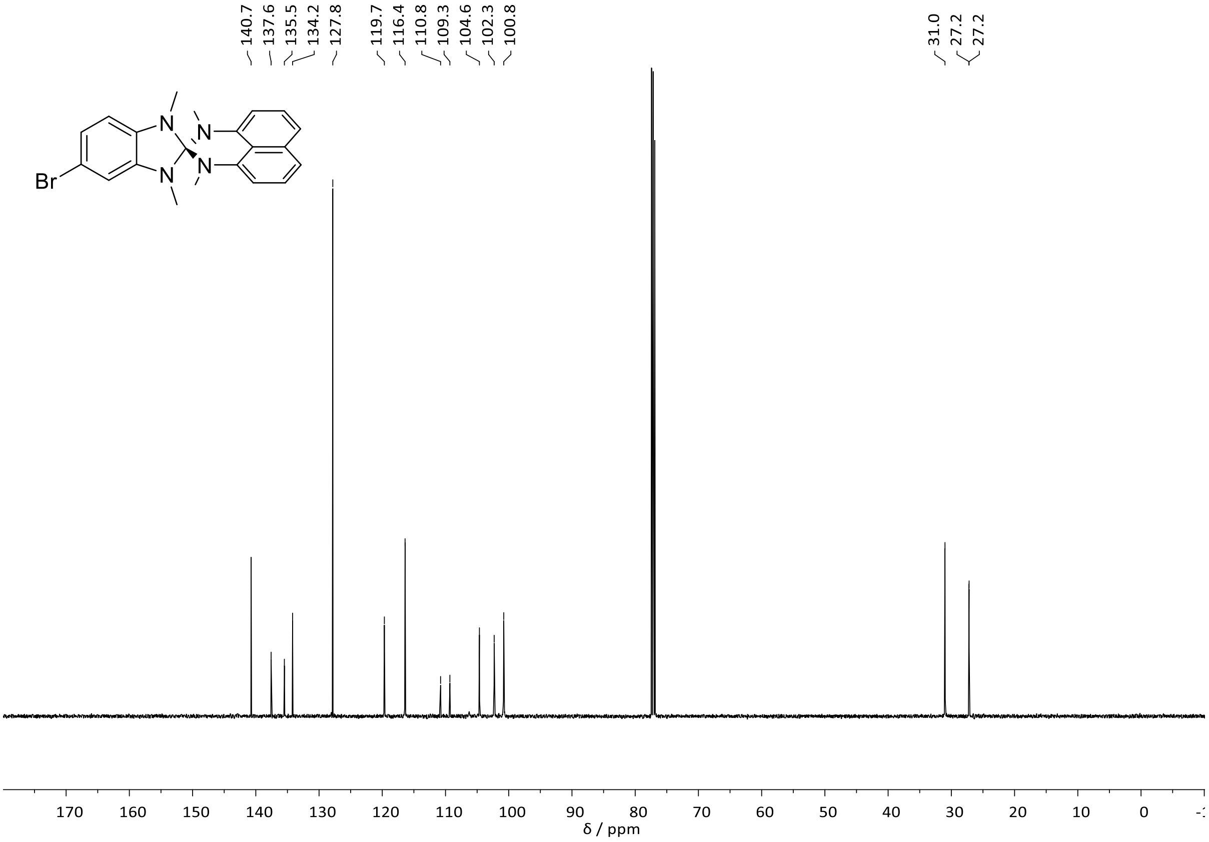

Figure S69: ^{13}C NMR spectrum (126 MHz) of **8a** in chloroform-*d*.

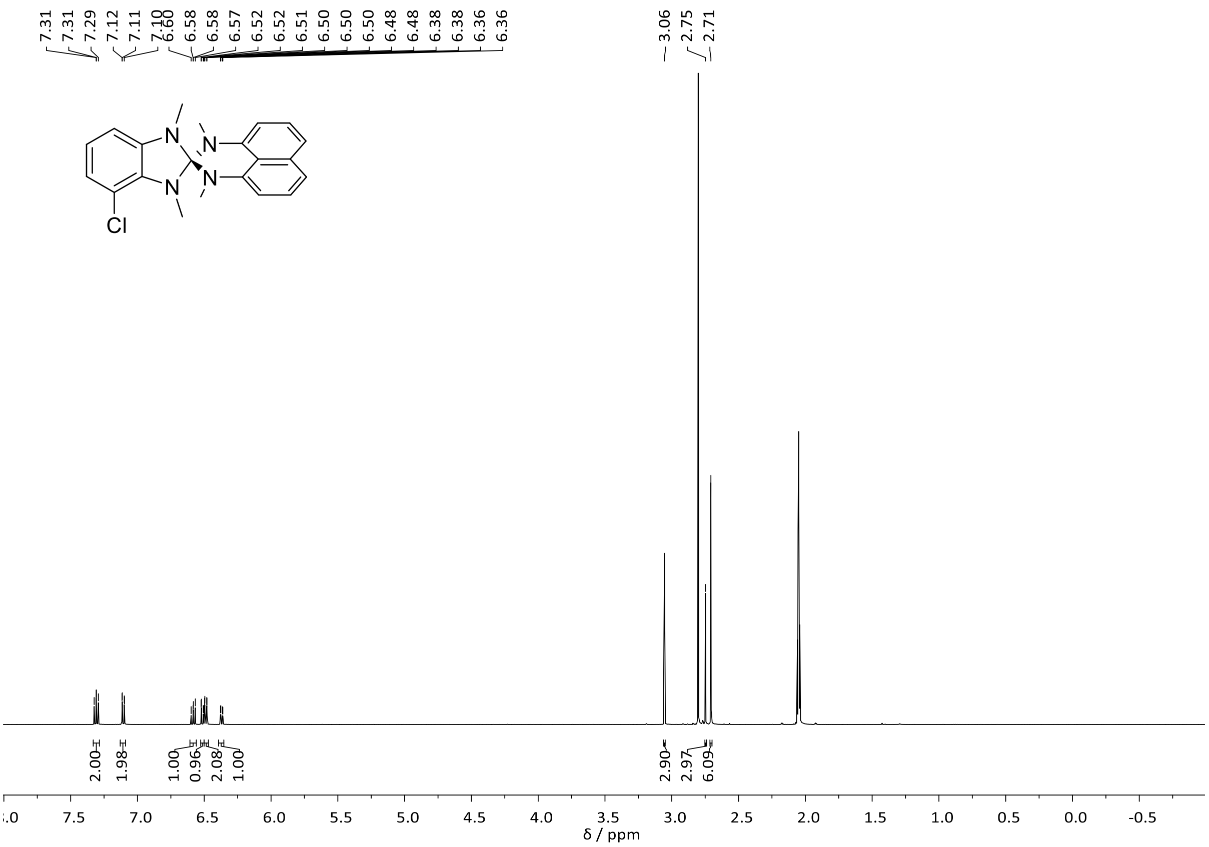

Figure S70: ^{1}H NMR spectrum (500 MHz) of **8b** in acetone-d_6, contains traces of H_2O.

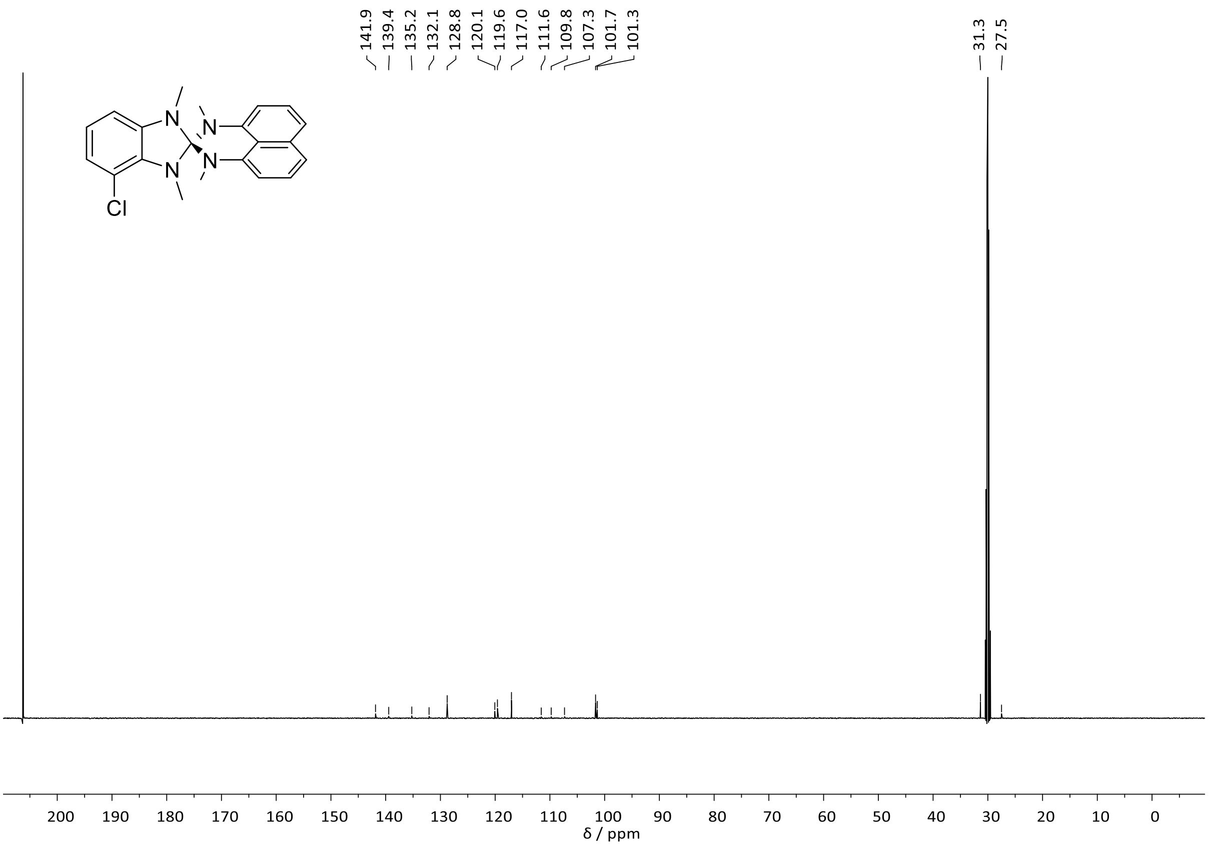

Figure S71: ^{13}C NMR spectrum (126 MHz) of **8b** in acetone-d_6.

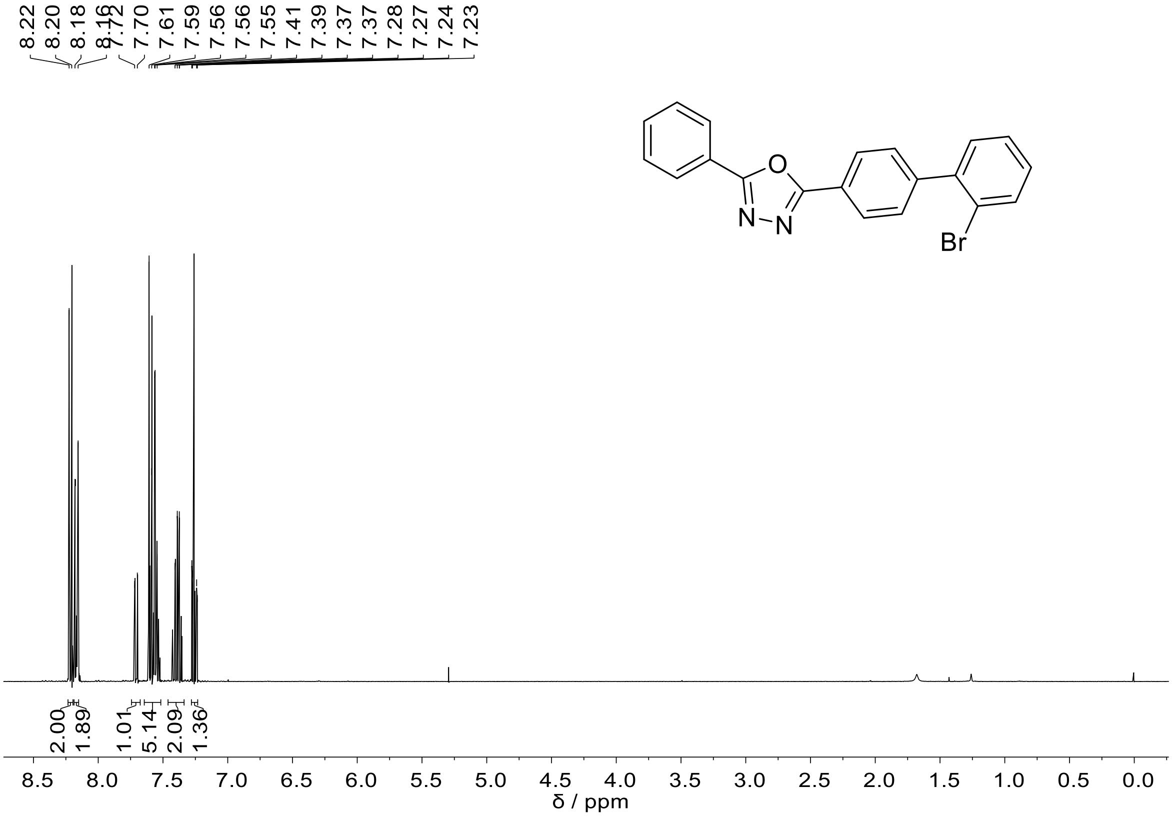

Figure S72: ^{1}H NMR spectrum (500 MHz) of **S8** in chloroform-*d*.

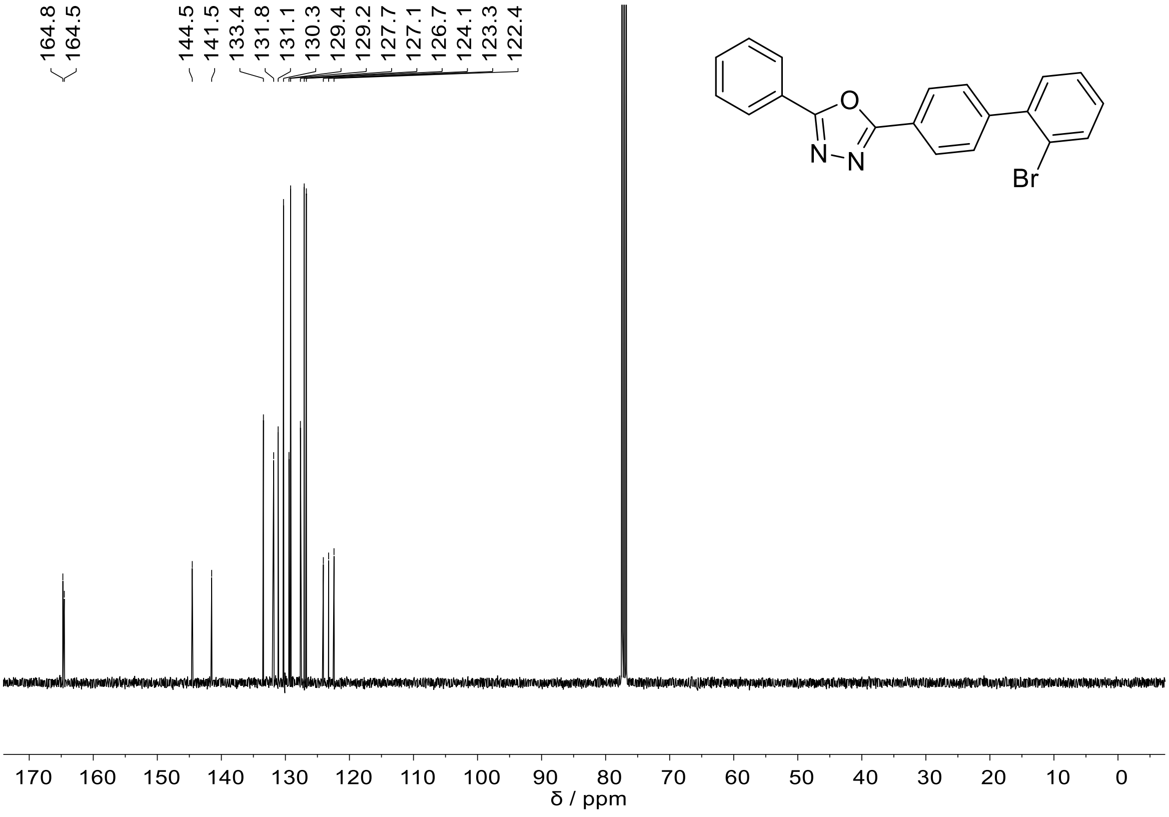

Figure S73: ^{13}C NMR spectrum (126 MHz) of **S8** in chloroform-*d*.

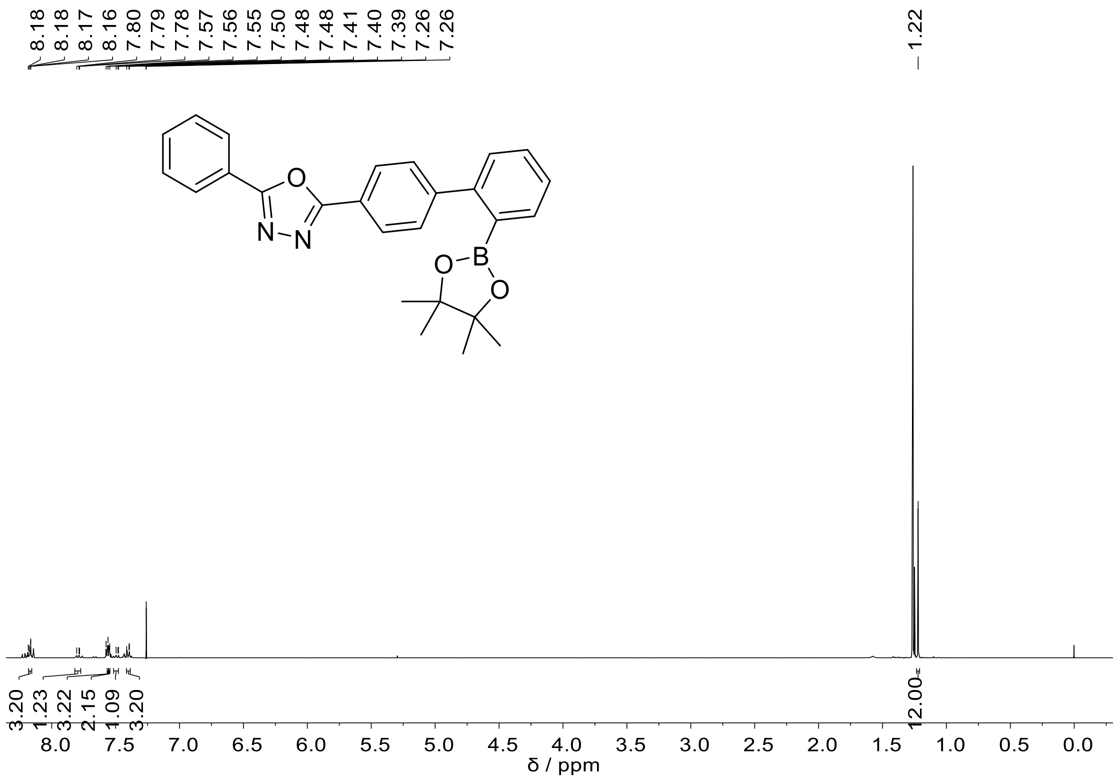

Figure S74: ^{1}H NMR spectrum (500 MHz) of **14** in chloroform-*d*.

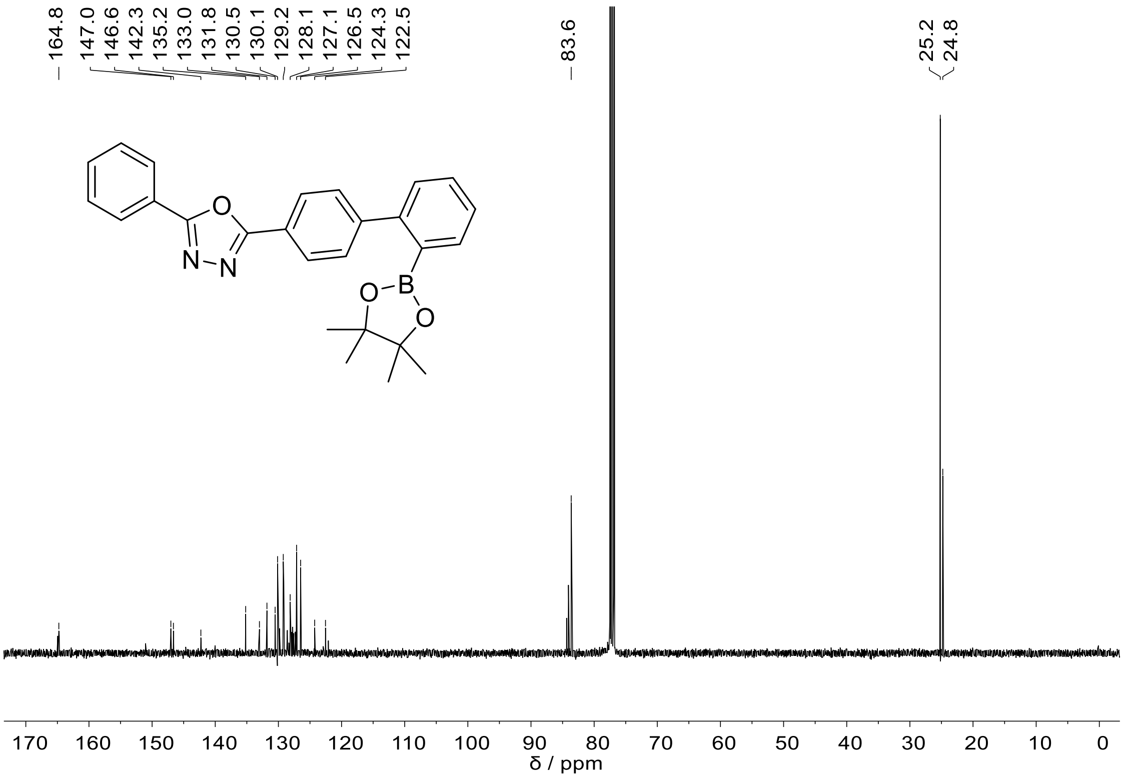

Figure S75: ^{13}C NMR spectrum (126 MHz) of **14** in chloroform-*d*.

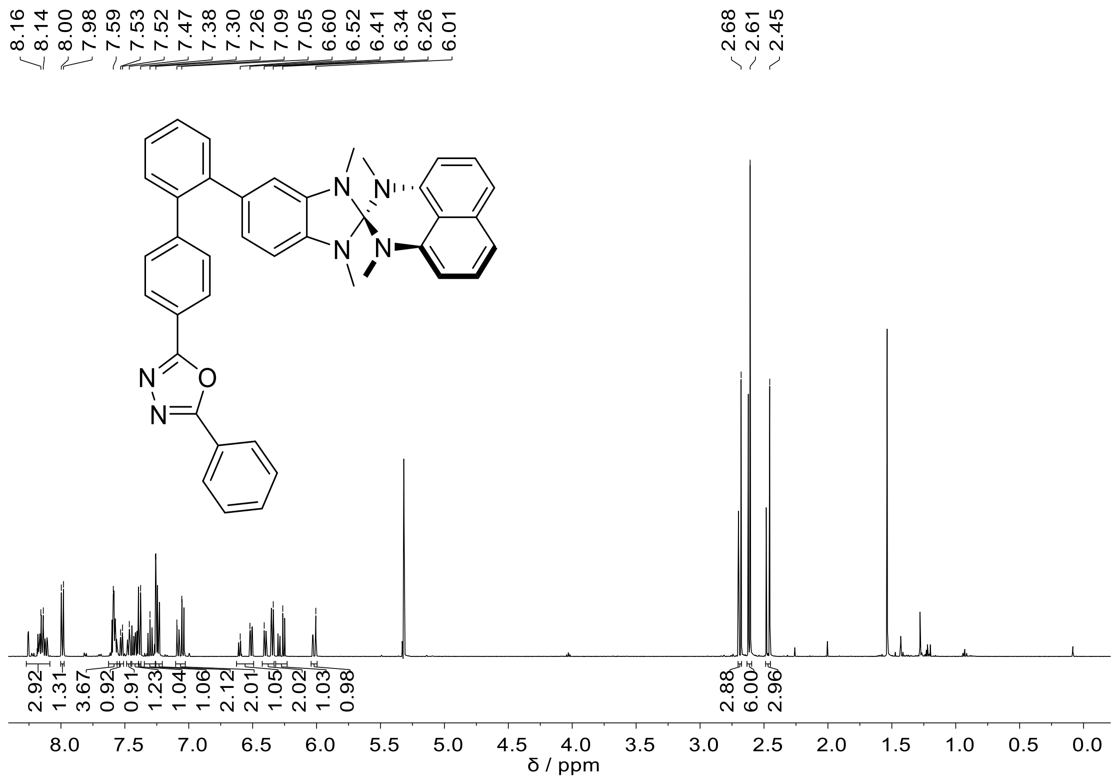

Figure S76: ^{1}H NMR spectrum (500 MHz) of **1-*ms*-DPOD** in dichloromethane-d_2.

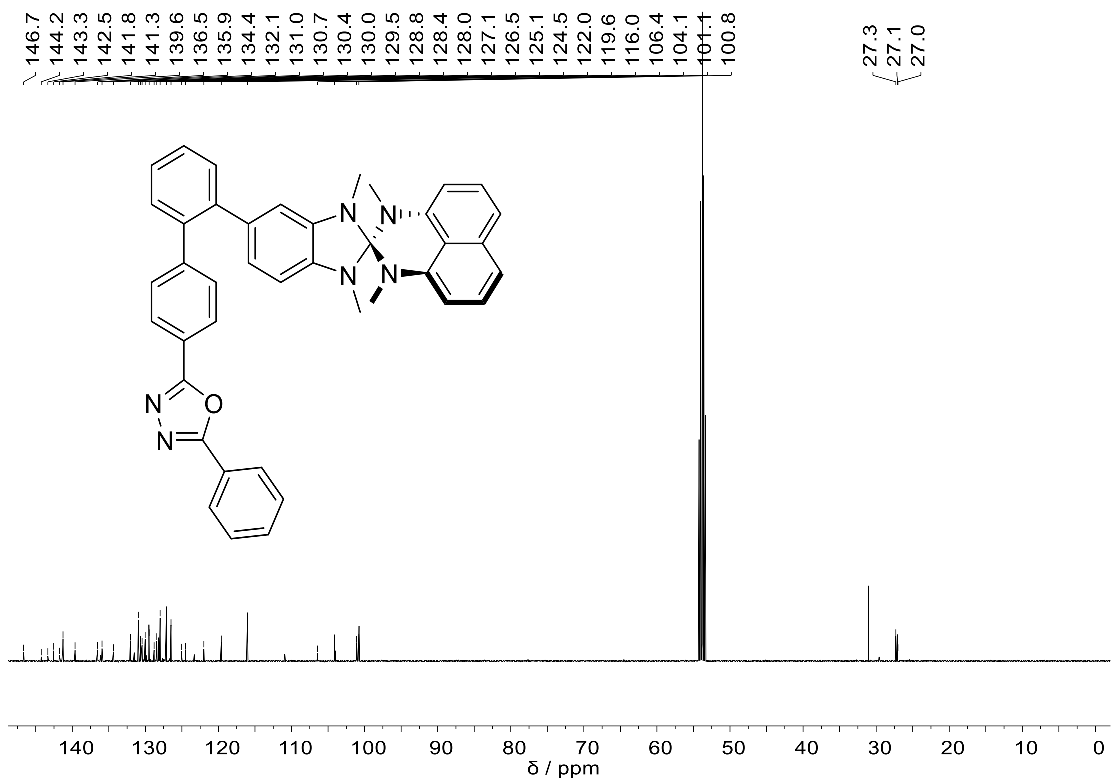

Figure S77: ^{13}C NMR spectrum (126 MHz) of **1-*ms*-DPOD** in dichloromethane-d_2.

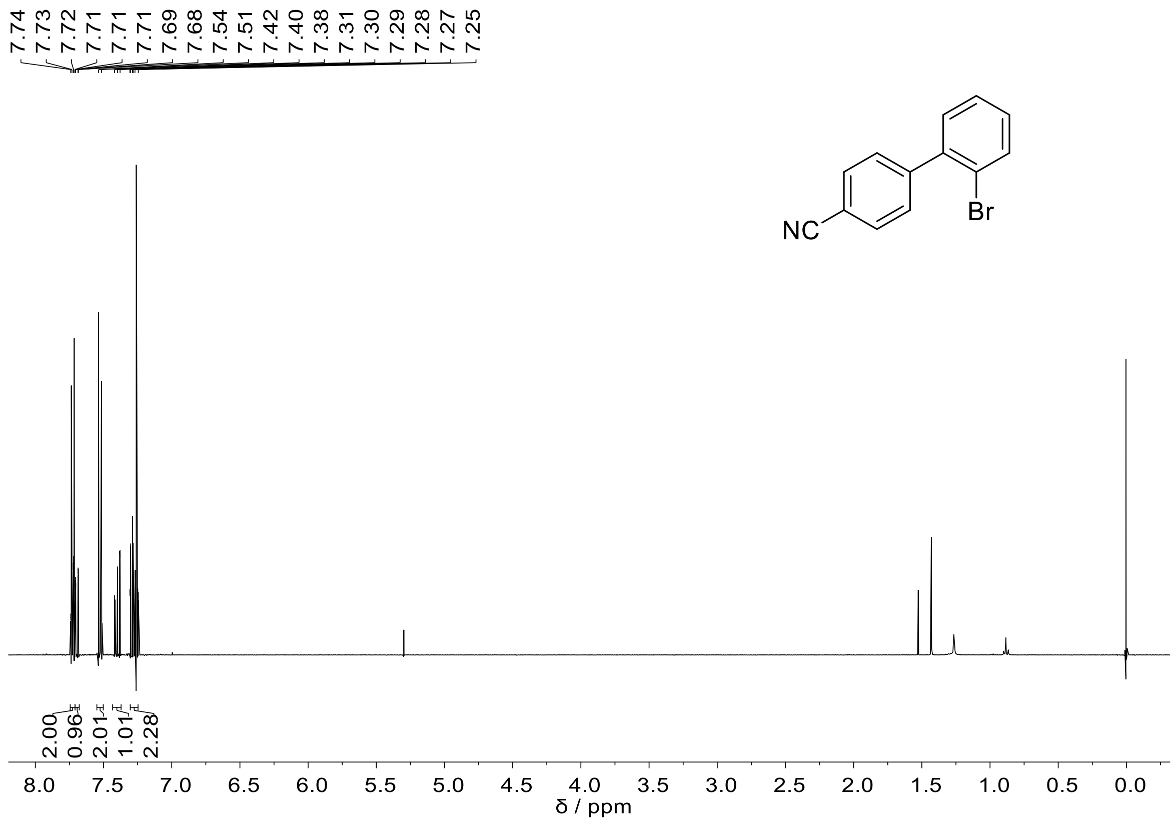

Figure S78: ^{1}H NMR spectrum (400 MHz) of **BN-Br** in chloroform-*d*.

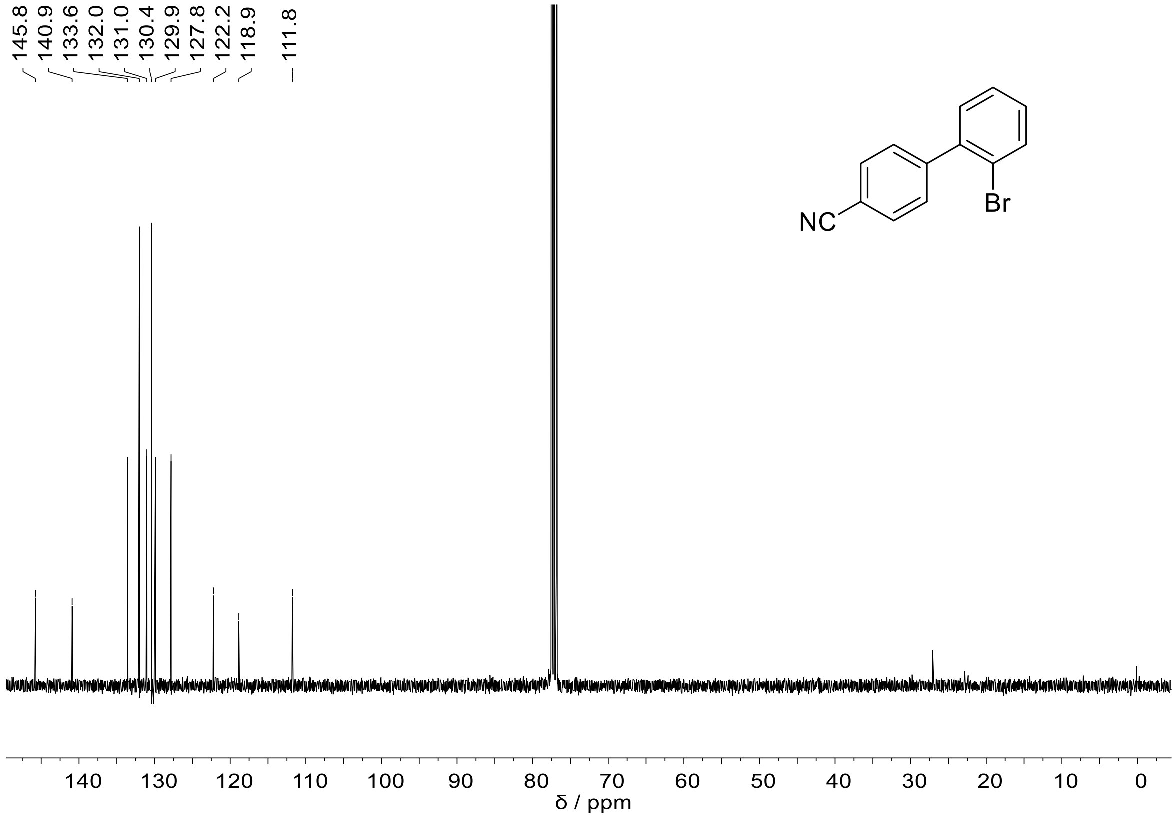

Figure S79: ^{13}C NMR spectrum (101 MHz) of **BN-Br** in chloroform-*d*.

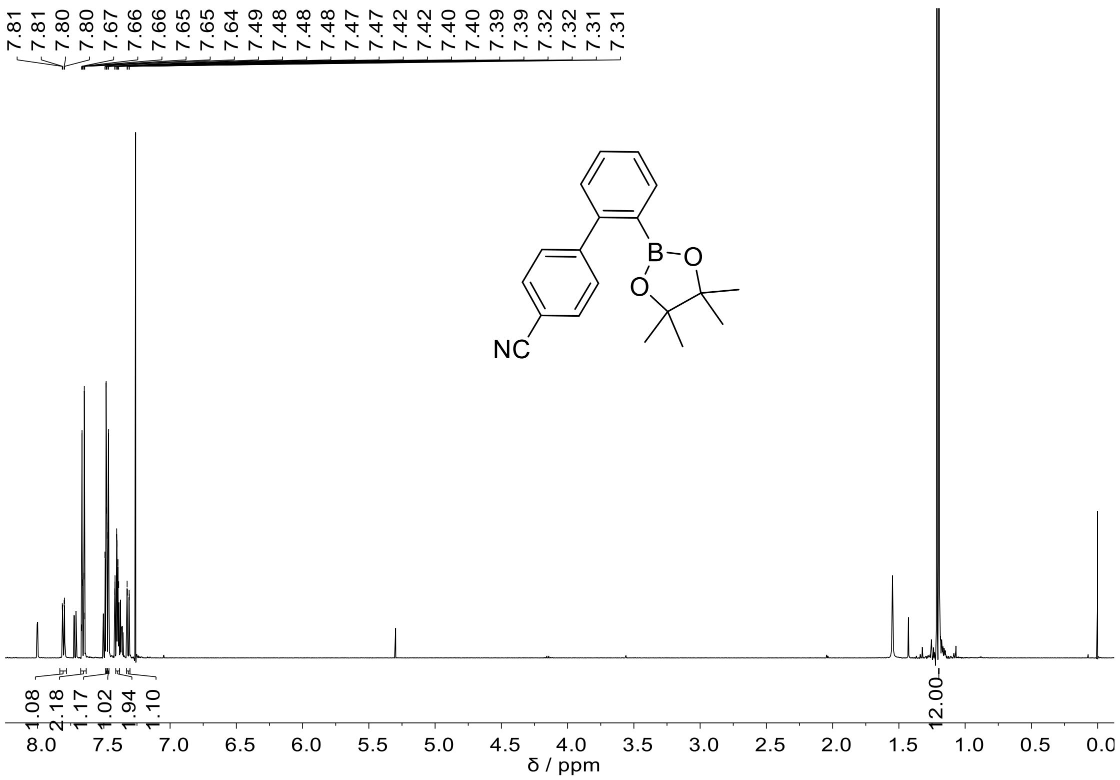

Figure S80: ^{1}H NMR spectrum (500 MHz) of **20** in chloroform-*d*.

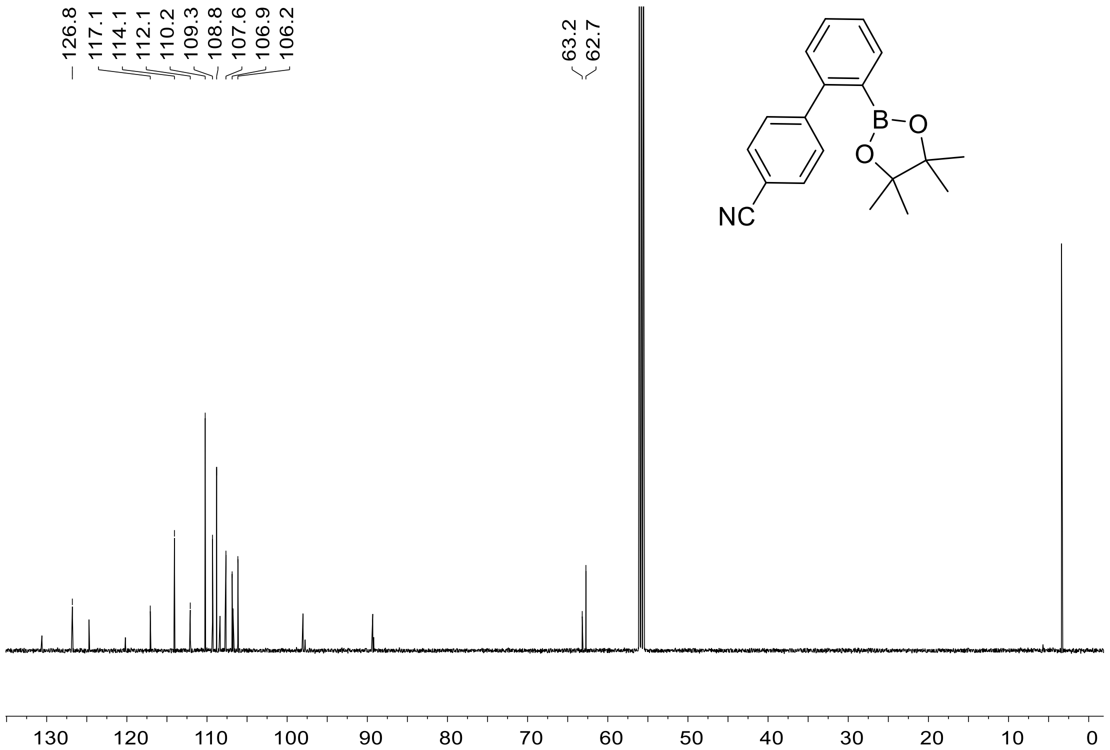

Figure S81: ^{13}C NMR spectrum (126 MHz) of **20** in chloroform-*d*.

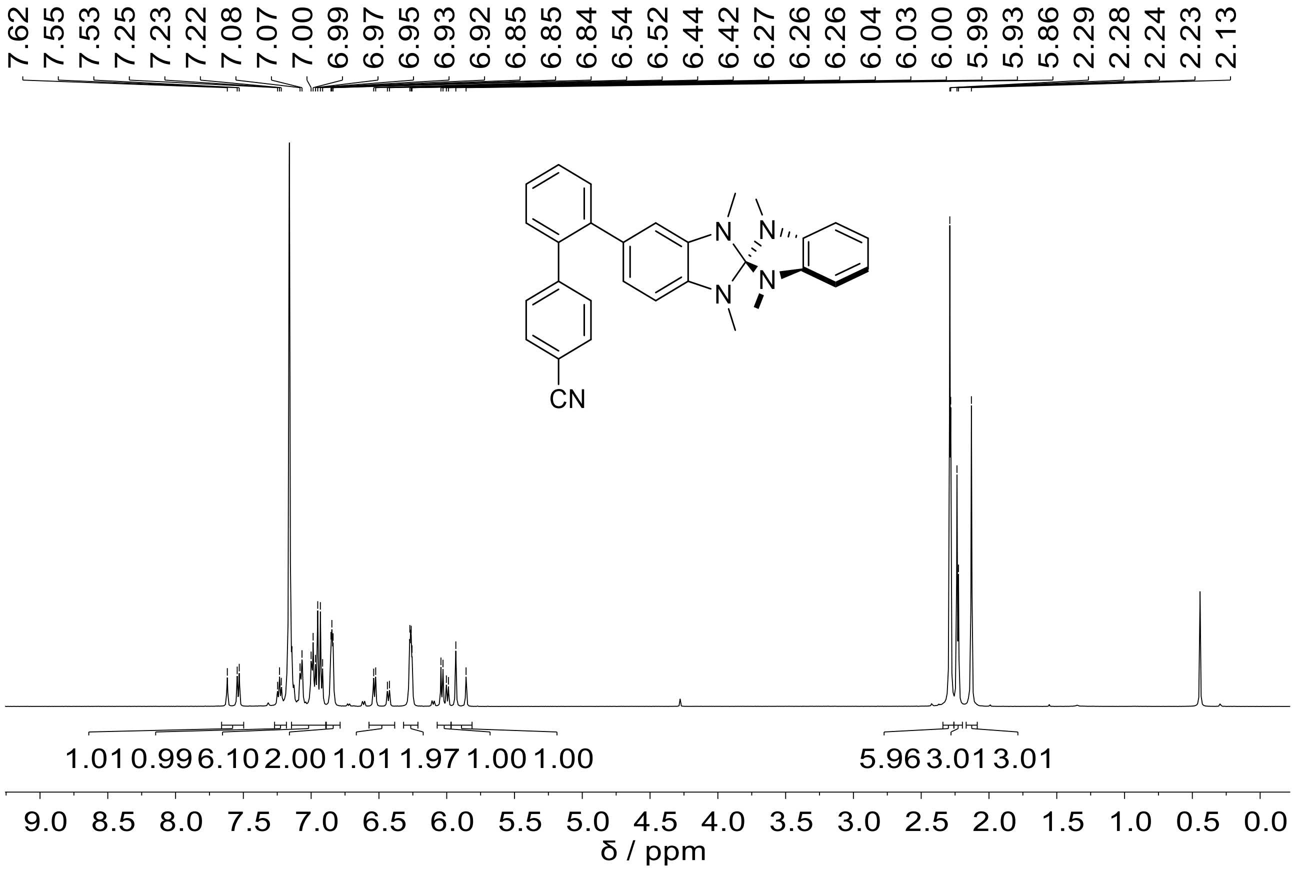

Figure S82: ^{1}H NMR spectrum (500 MHz) of **2-ms-BN** in C_6D_6.

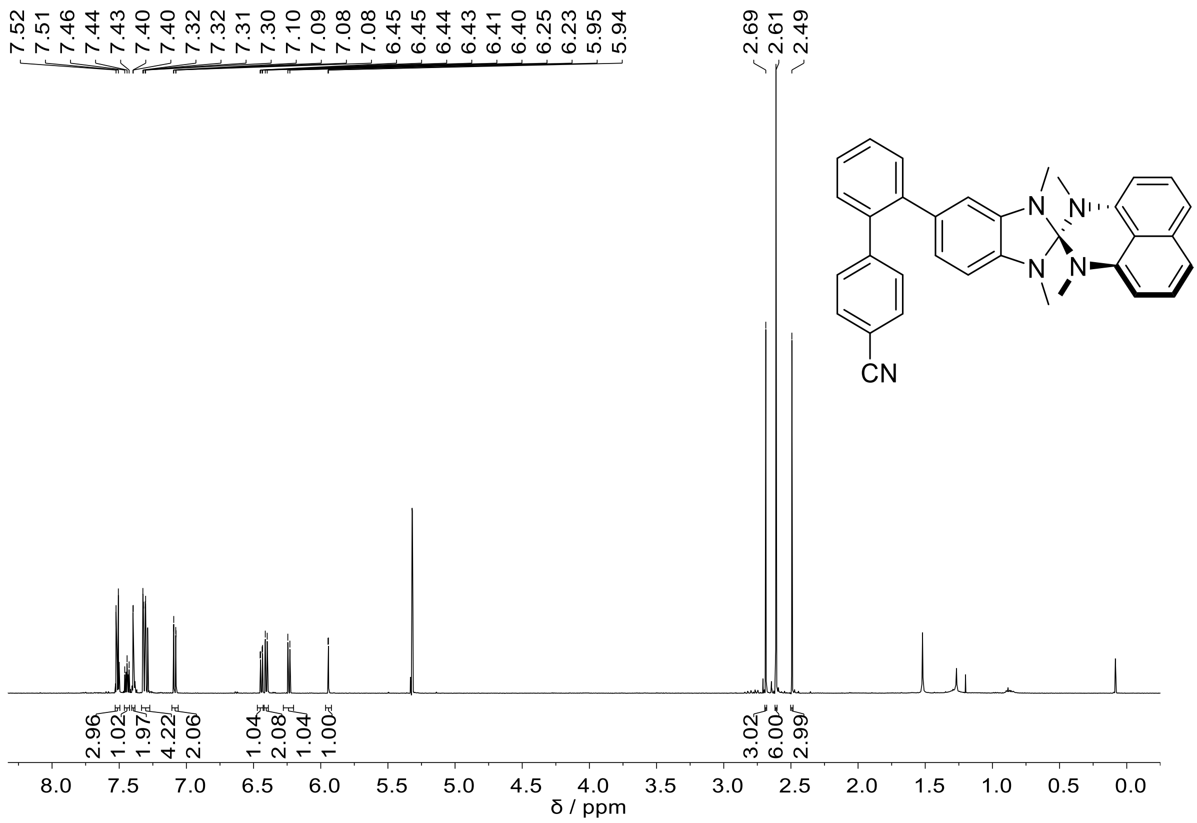

Figure S83: ^{1}H NMR spectrum (500 MHz, 303 K) of **1-*ms*-BN** in dichloromethane-d_2.

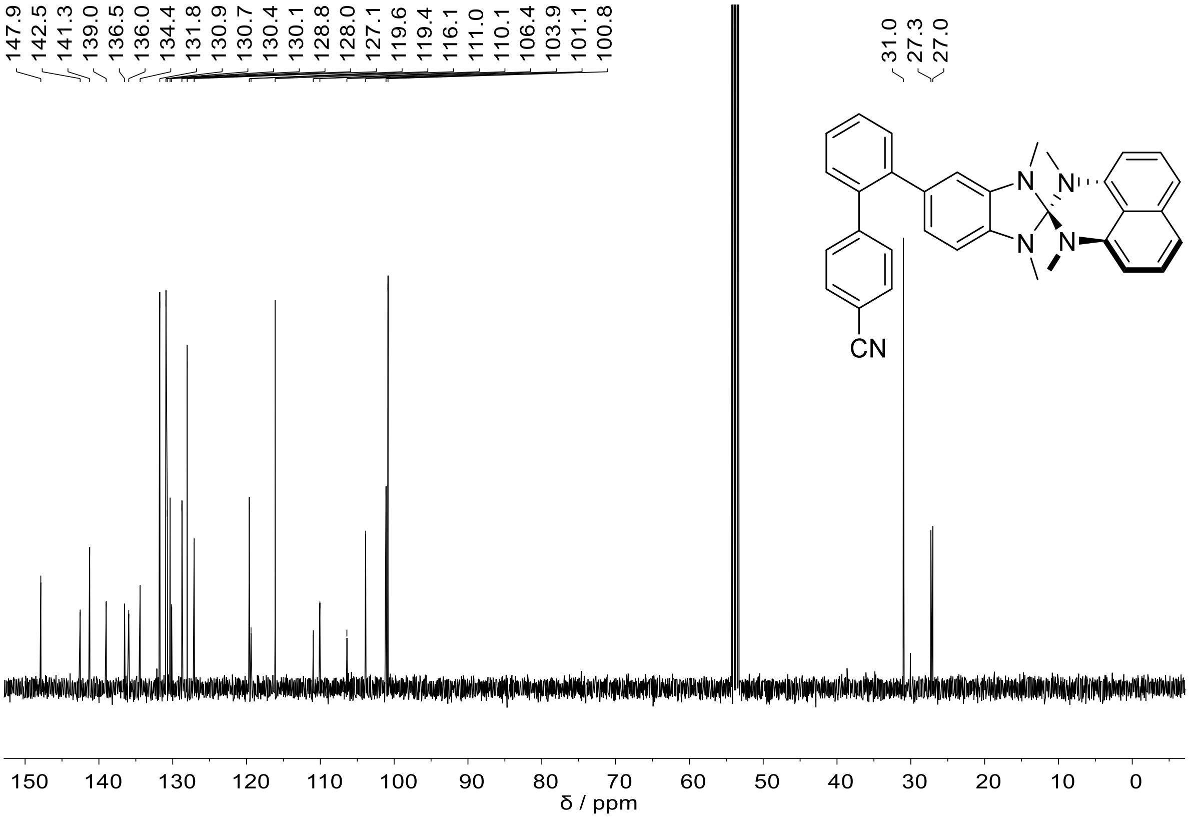

Figure S84: ^{13}C NMR spectrum (126 MHz) of **1-*ms*-BN** in dichloromethane-d_2.

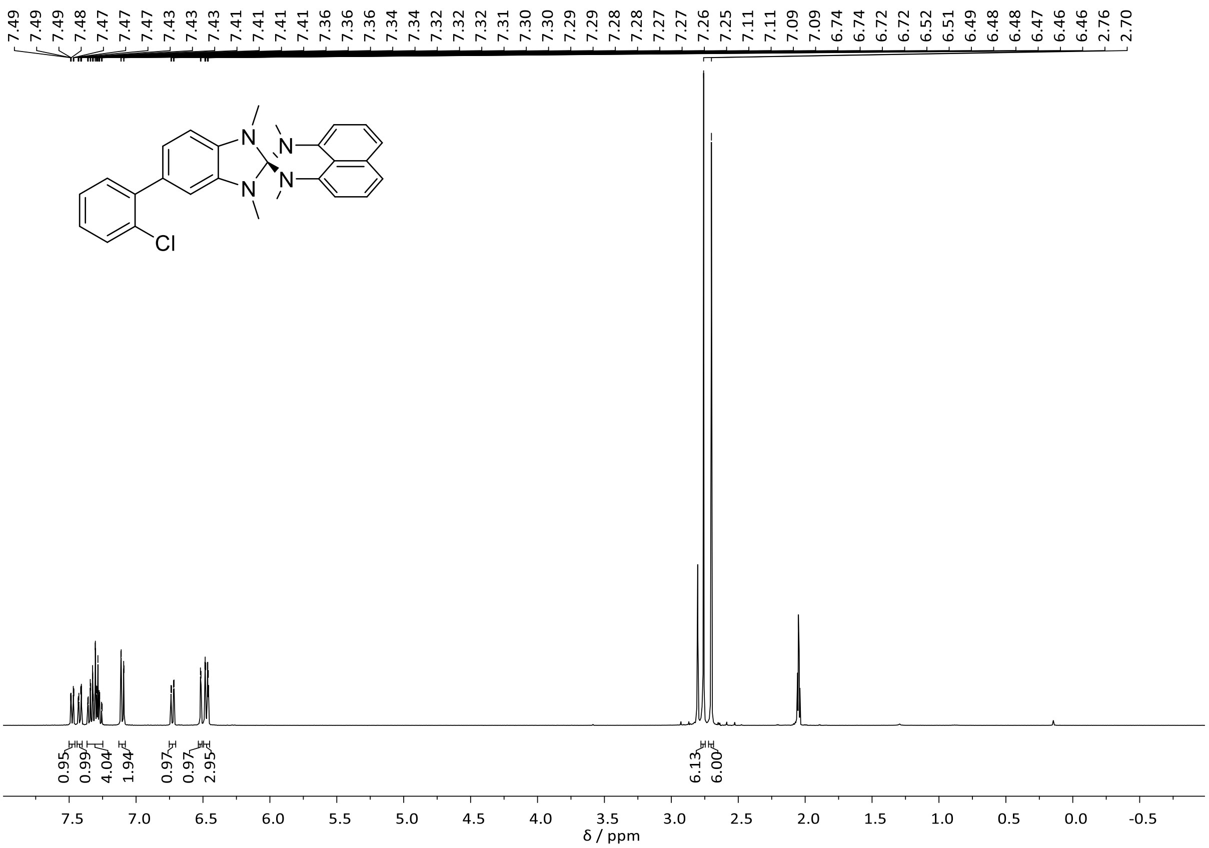

Figure S85: ^{1}H NMR spectrum (400 MHz, 303 K) of **12** in acetone-d_6, contains traces of H_2O.

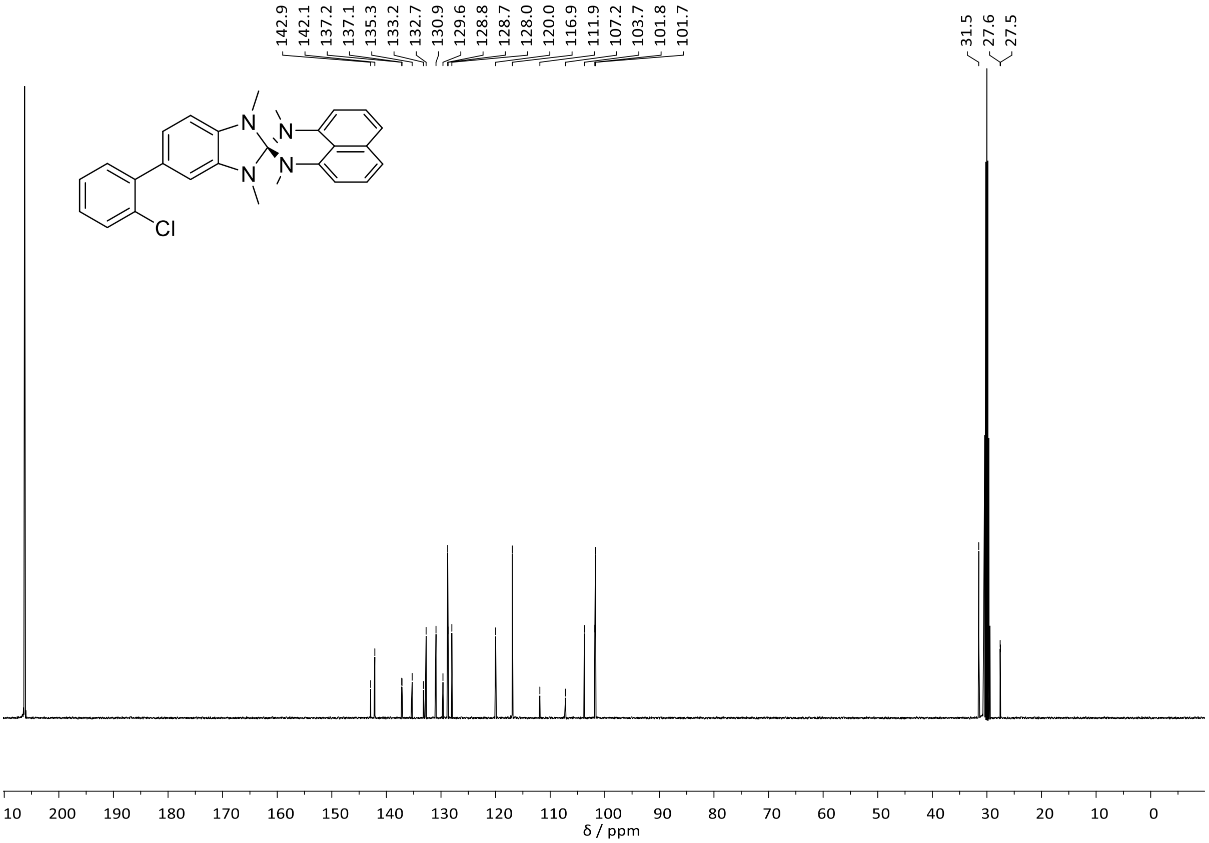

Figure S86: ^{13}C NMR spectrum (101 MHz, 303 K) of **12** in acetone-d_6.

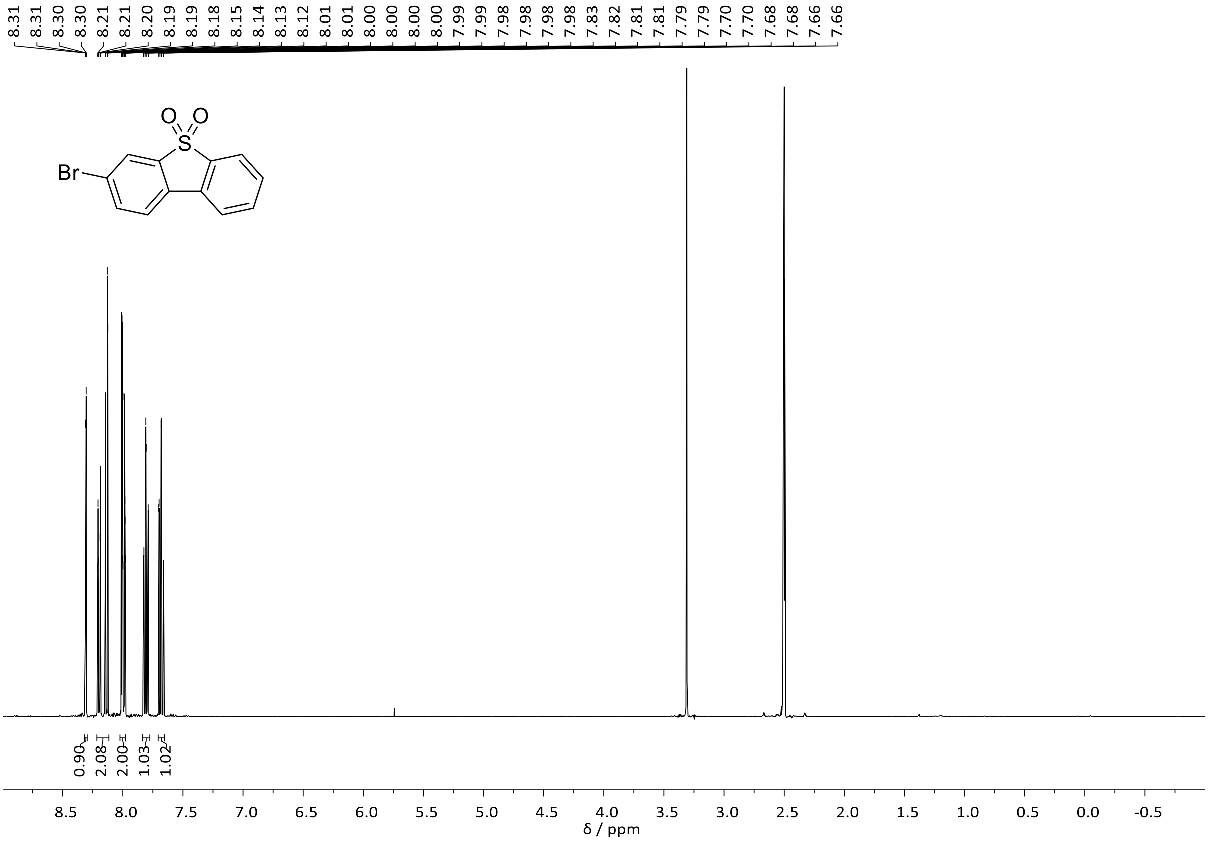

Figure S87: ^{1}H NMR spectrum (400 MHz, 303 K) of **DBTO-Br** in DMSO-d_6, contains traces of H_2O and dichloromethane.

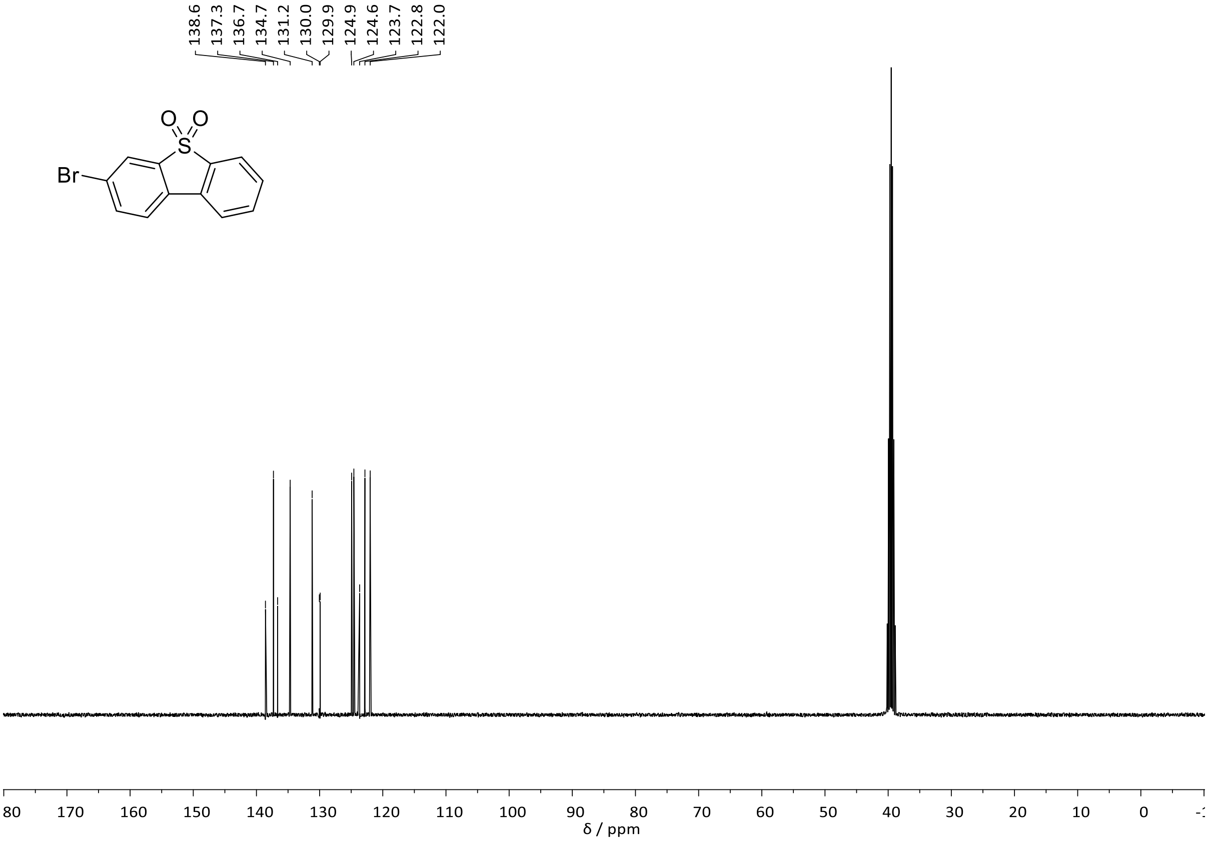

Figure S88: ^{13}C NMR spectrum (101 MHz, 303 K) of **DBTO-Br** in DMSO-d_6.

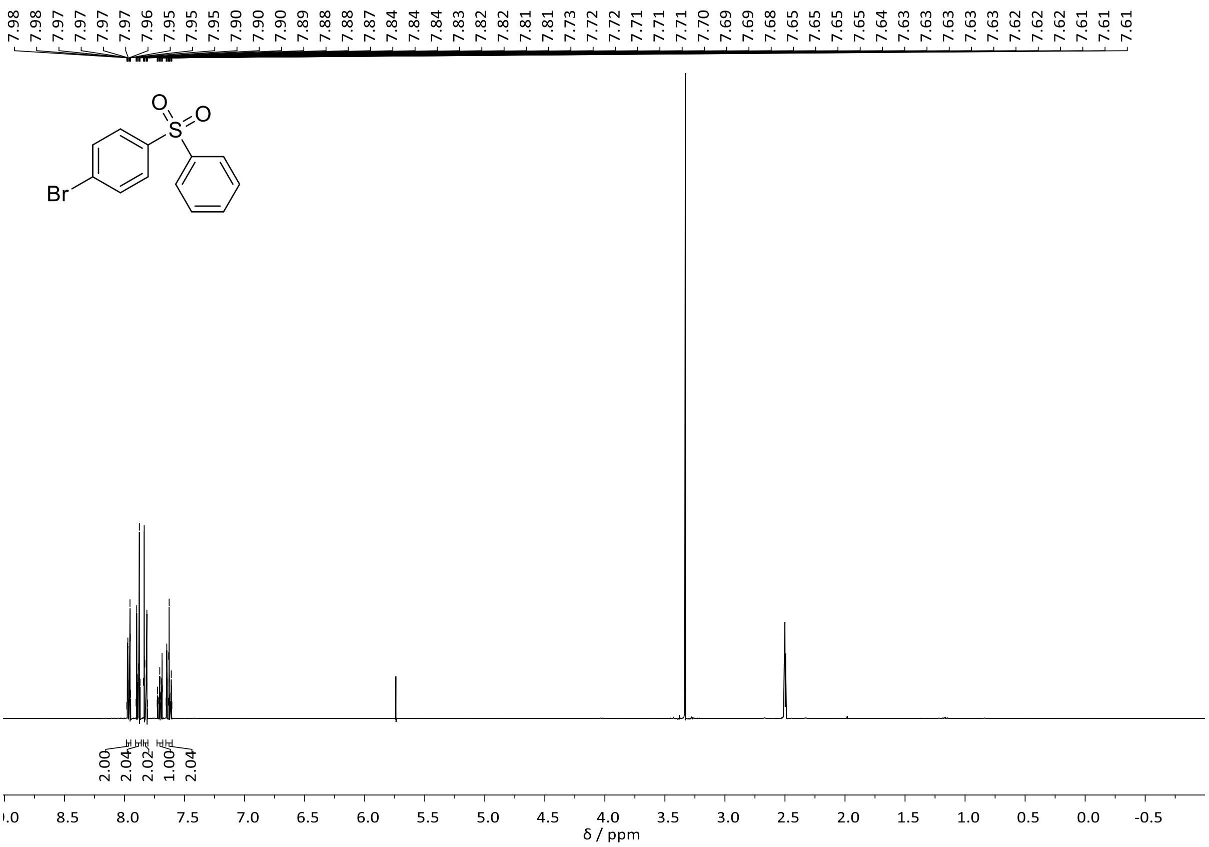

Figure S89: ^{1}H NMR spectrum (400 MHz, 303 K) of **DPS-Br** in DMSO-d_6, contains traces of H_2O and dichloromethane.

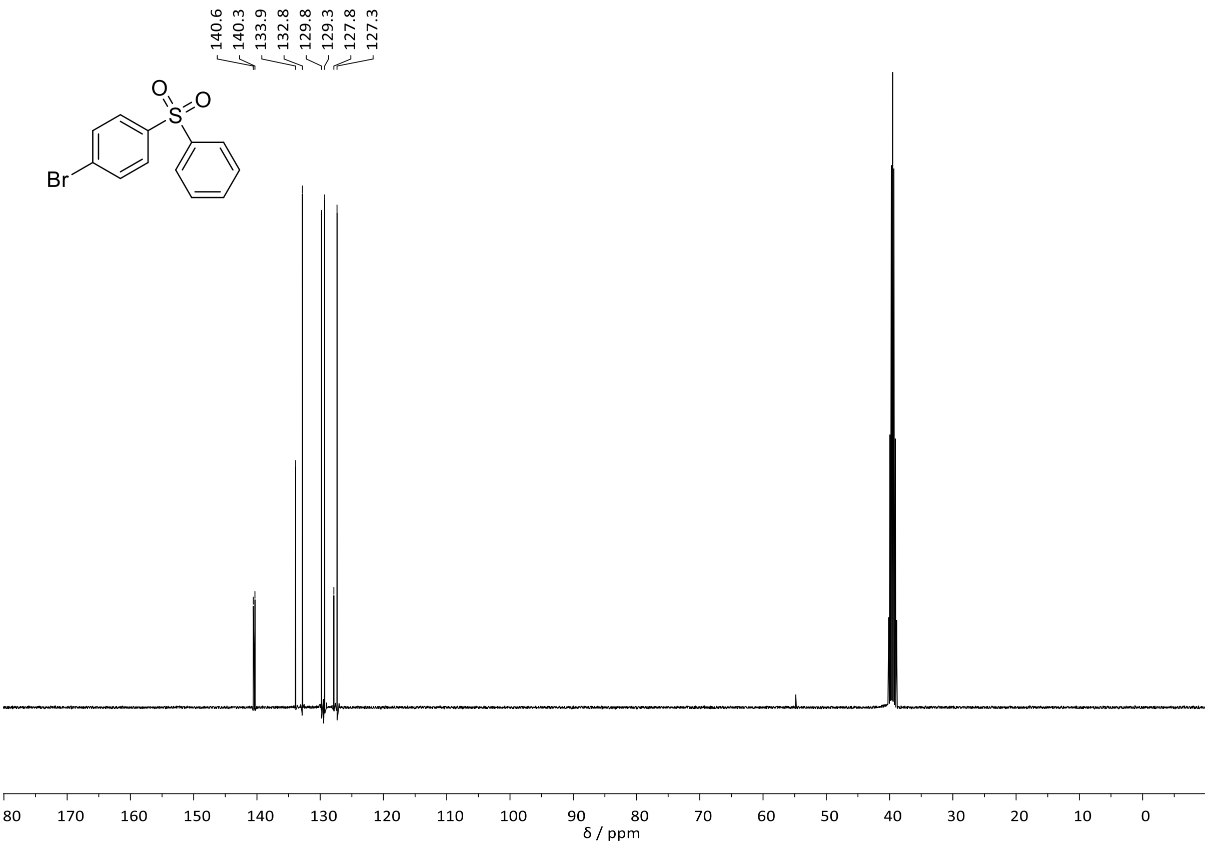

Figure S90: ^{13}C NMR spectrum (101 MHz, 303 K) of **DPS-Br** in DMSO-d_6, contains traces of dichloromethane.

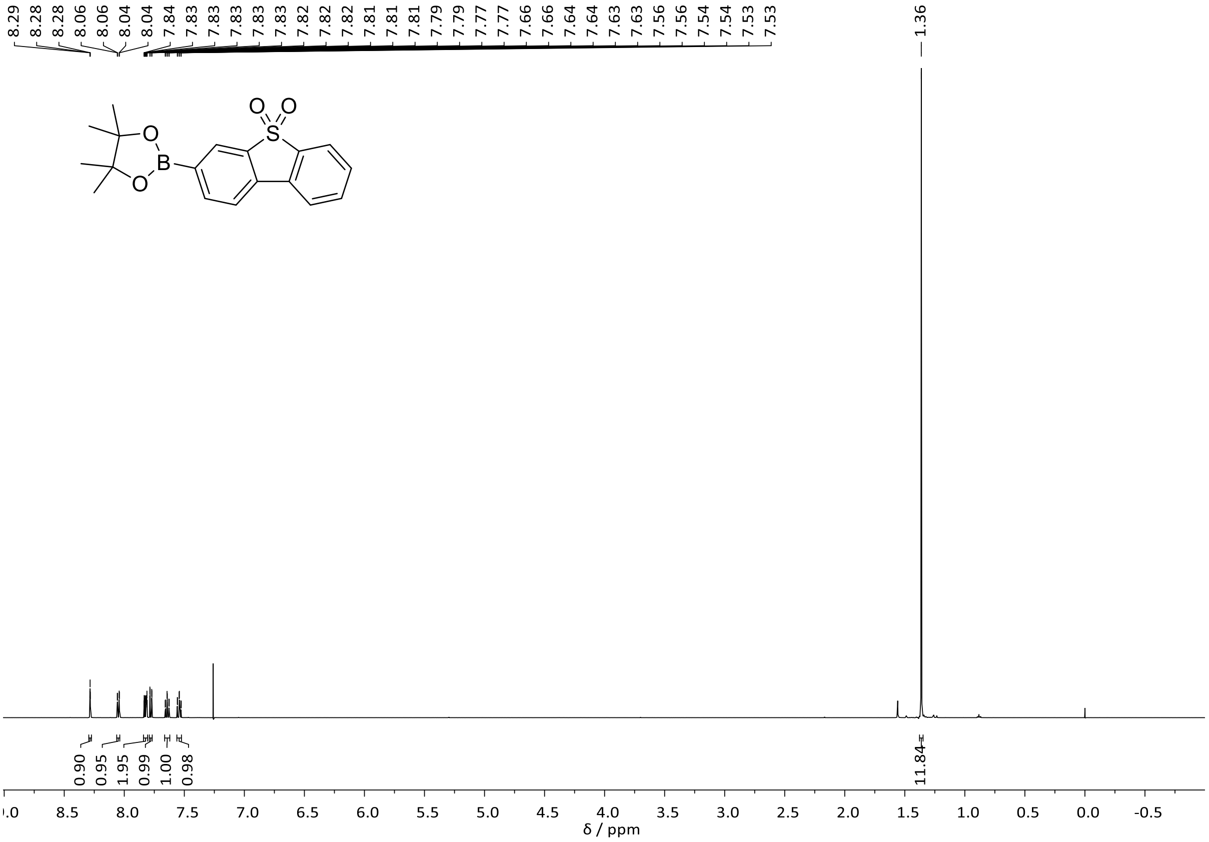

Figure S91: ^{1}H NMR spectrum (500 MHz) of **DBTO-Bpin** in chloroform-*d*, contains traces of H_2O and grease.

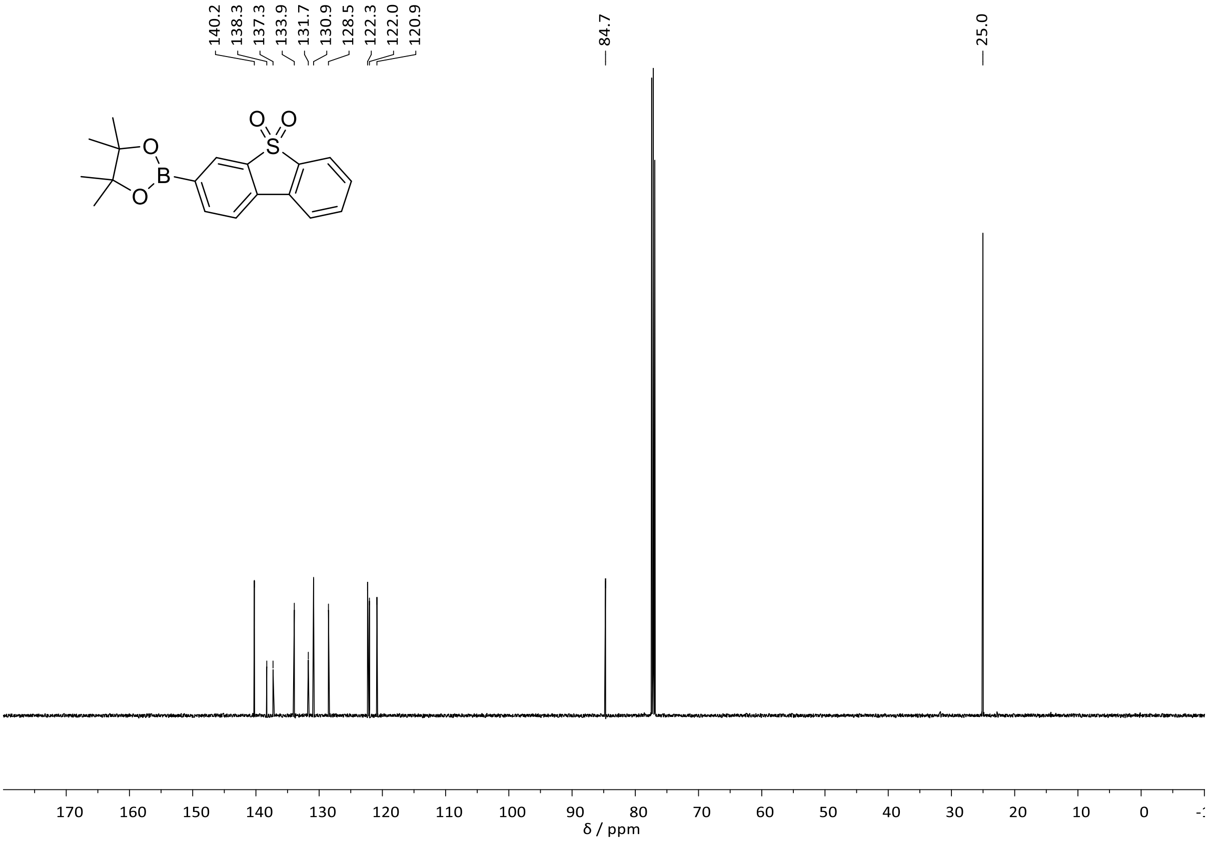

Figure S92: ^{13}C NMR spectrum (126 MHz) of **DBTO-Bpin** in chloroform-*d*.

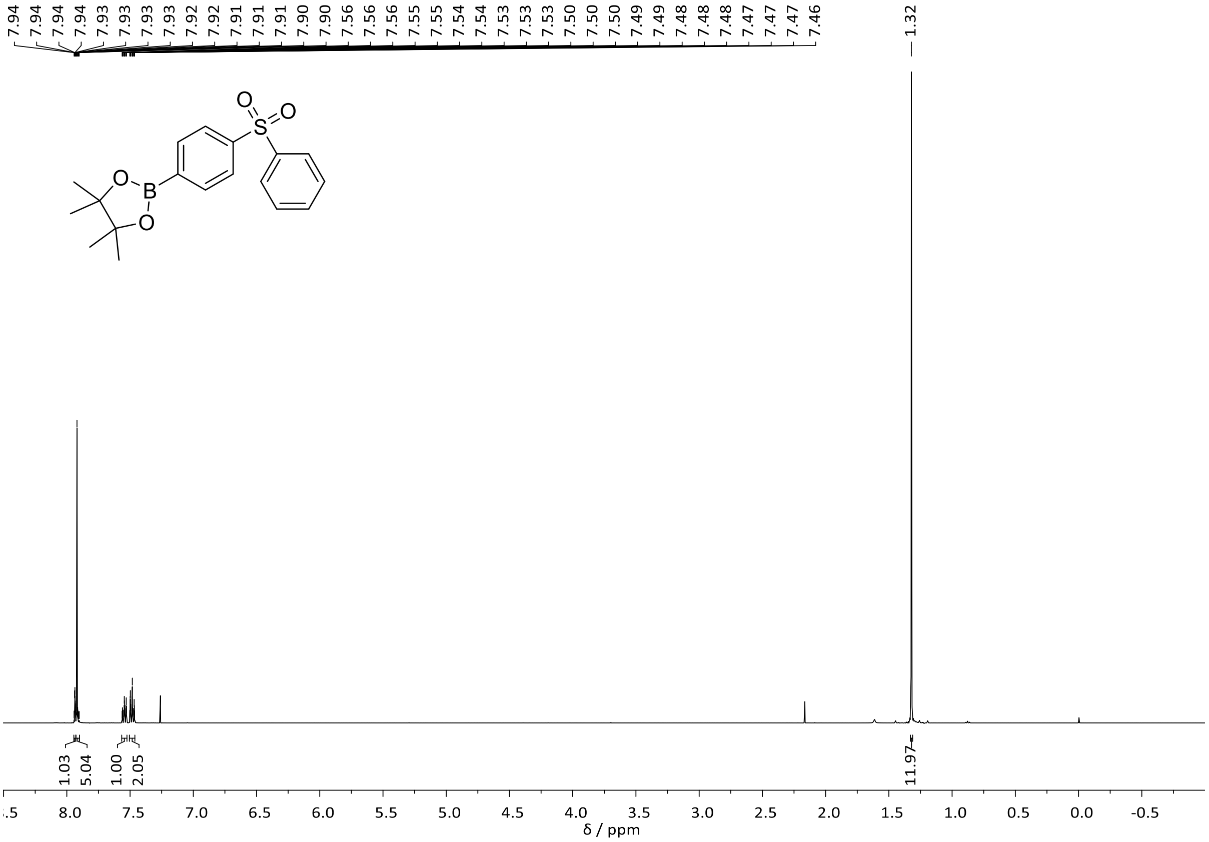

Figure S93: ^{1}H NMR spectrum (500 MHz) of **DPS-Bpin** in chloroform-*d*, contains traces of acetone.

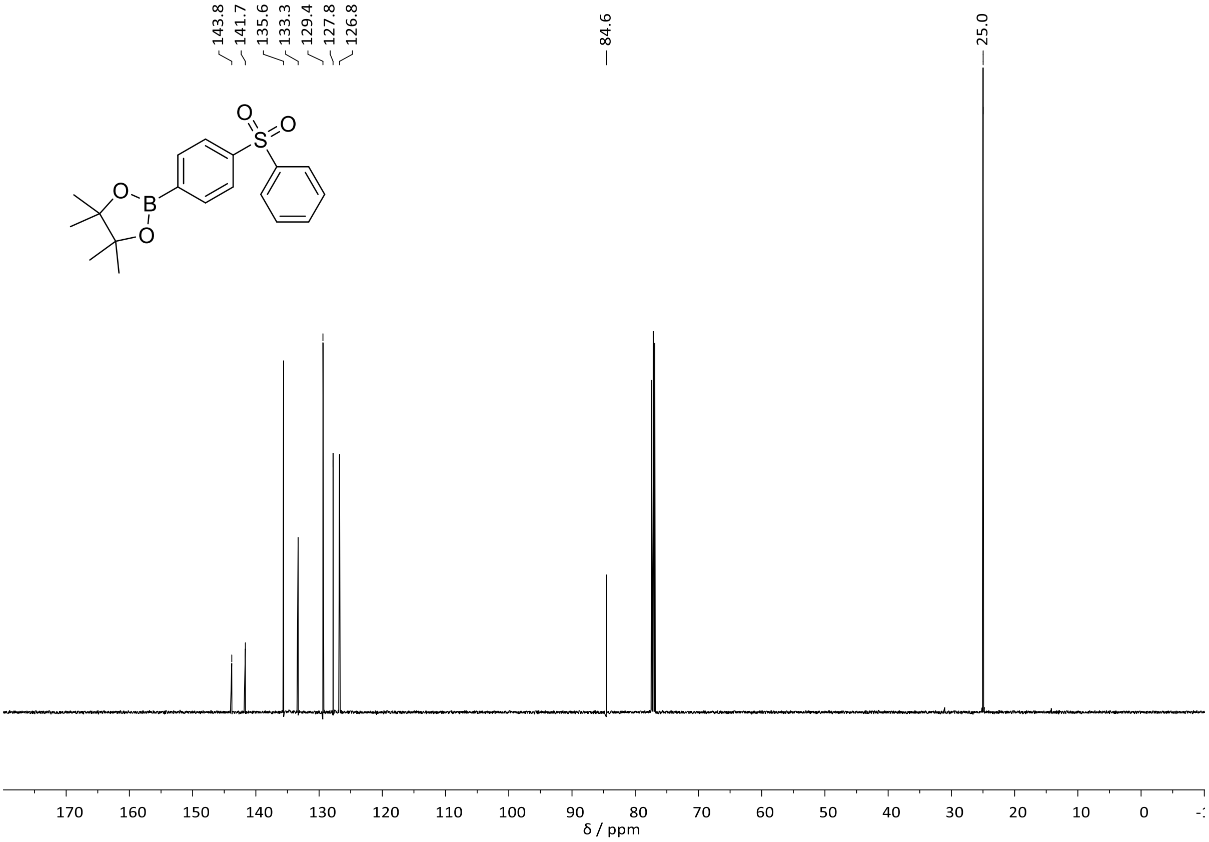

Figure S94: ^{13}C NMR spectrum (126 MHz) of **DPS-Bpin** in chloroform-*d*.

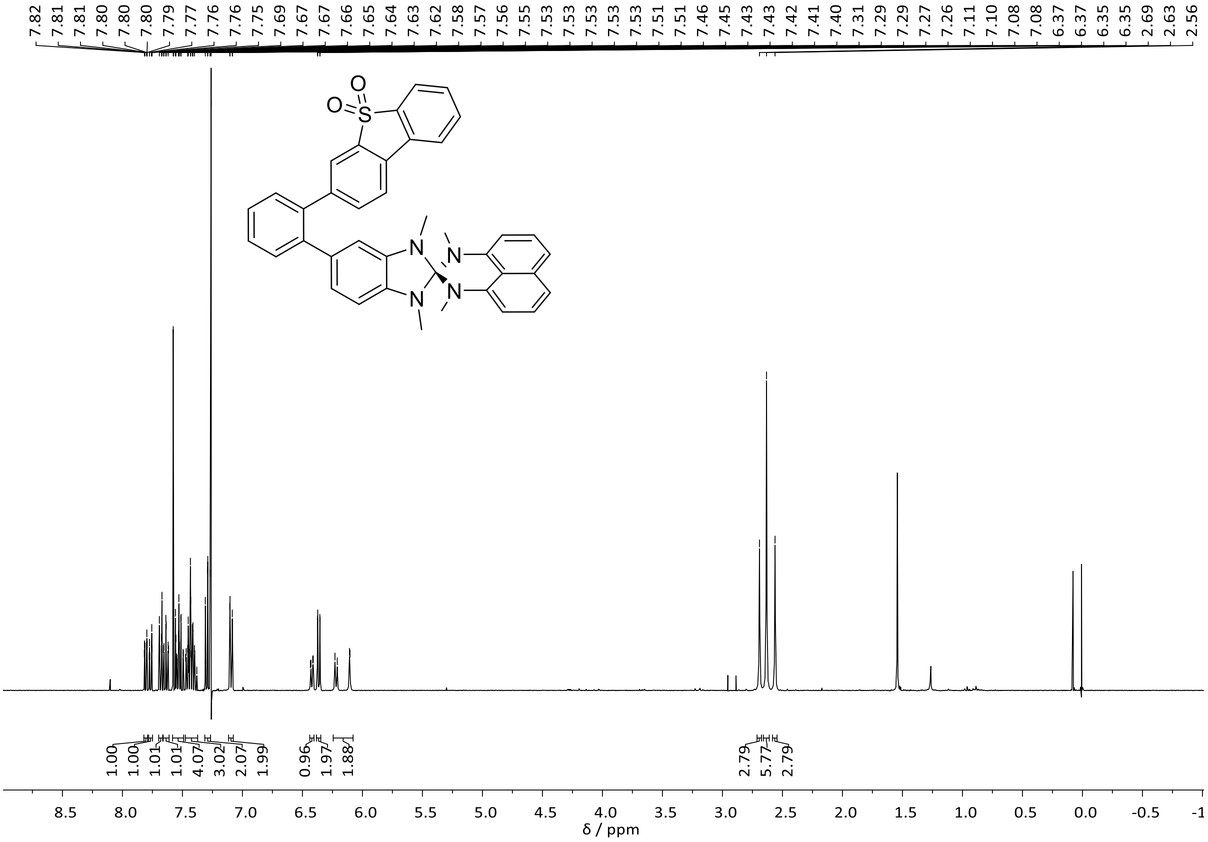

Figure S95: ^{1}H NMR spectrum (400 MHz, 303 K) of **1-ms-DBTO** in chloroform-*d*.

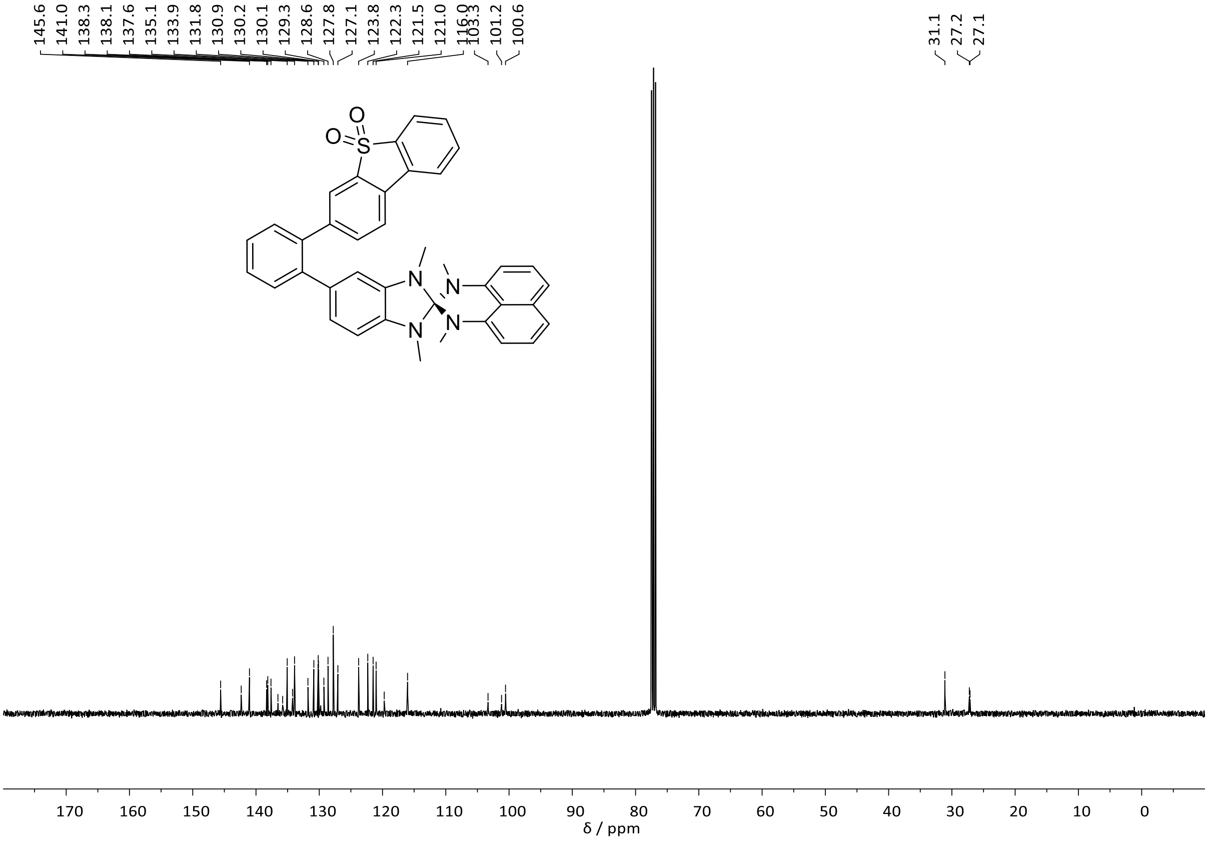

Figure S96: ^{13}C NMR spectrum (101 MHz, 303 K) of **1-ms-DBTO** in chloroform-*d*.

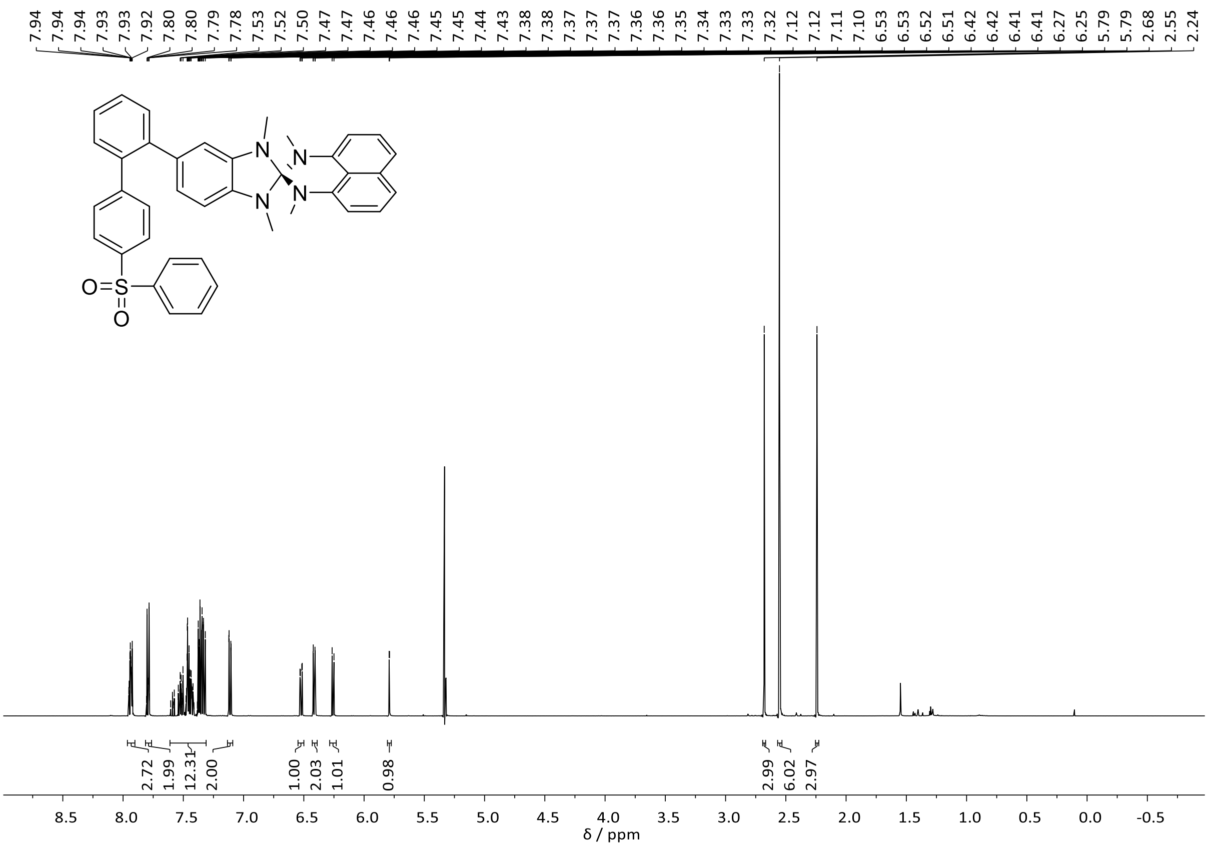

Figure S97: ^{1}H NMR spectrum (500 MHz) of **1-ms-DPS** in dichloromethane-d_2, contains traces of H_2O and grease.

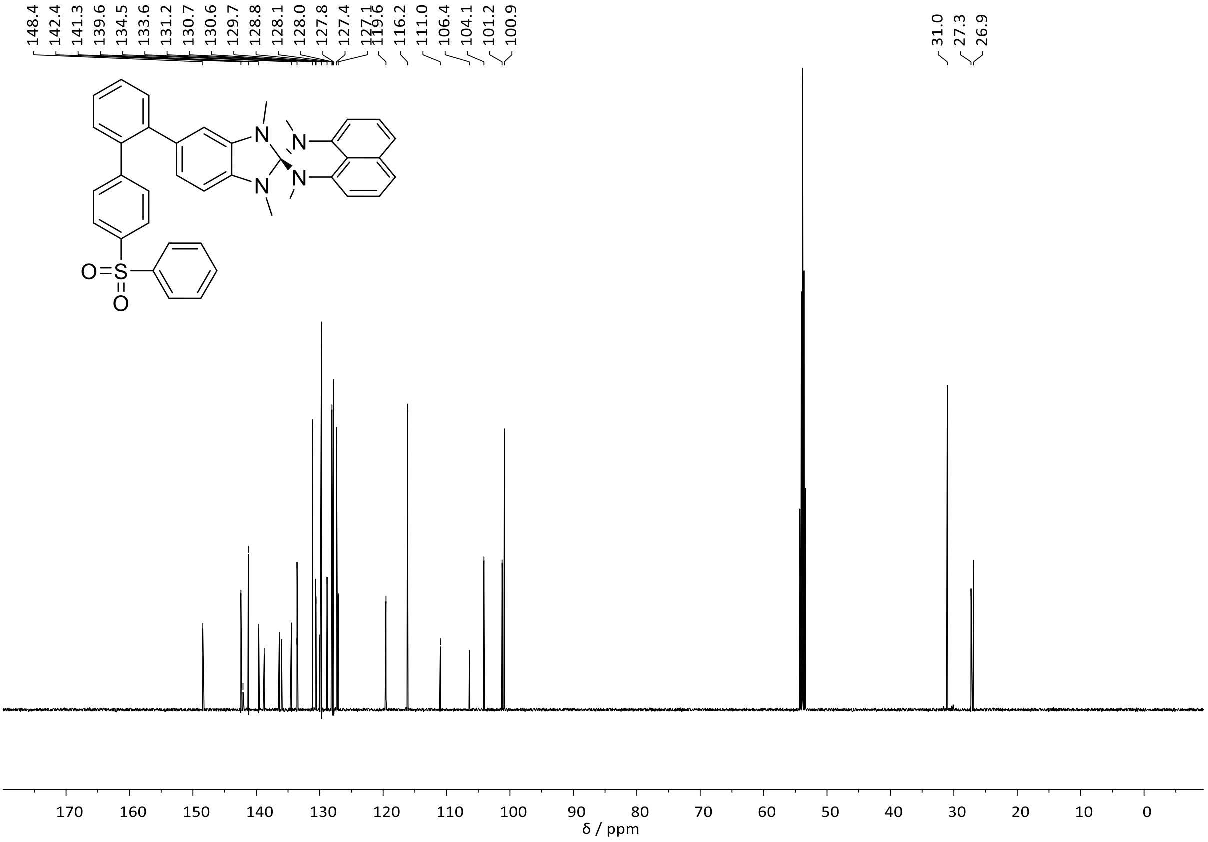

Figure S98: ^{13}C NMR spectrum (126 MHz) of **1-ms-DPS** in dichloromethane-d_2, contains traces of grease.

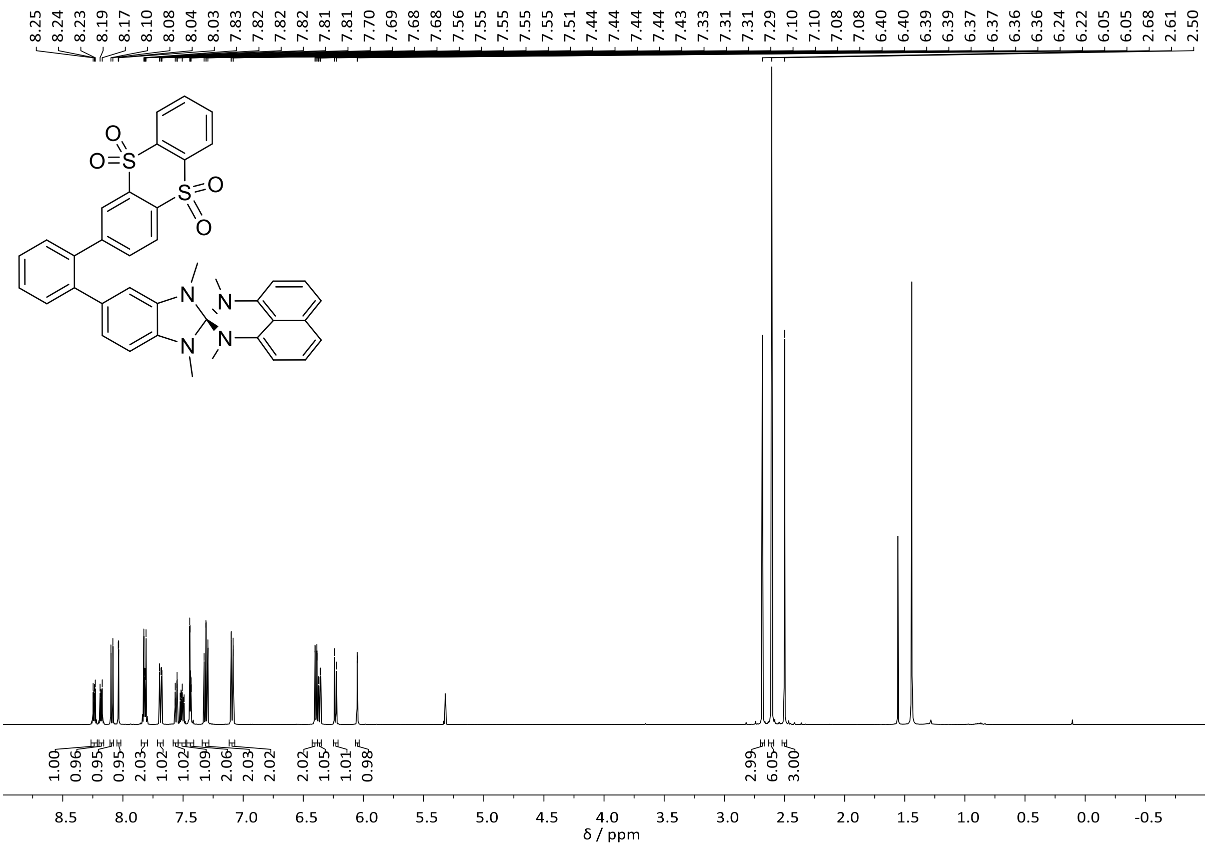

Figure S99: ^{1}H NMR spectrum (500 MHz) of **1-ms-TTO** in dichloromethane-*d*$_2$, contains traces of H_2O, grease and cyclohexane.

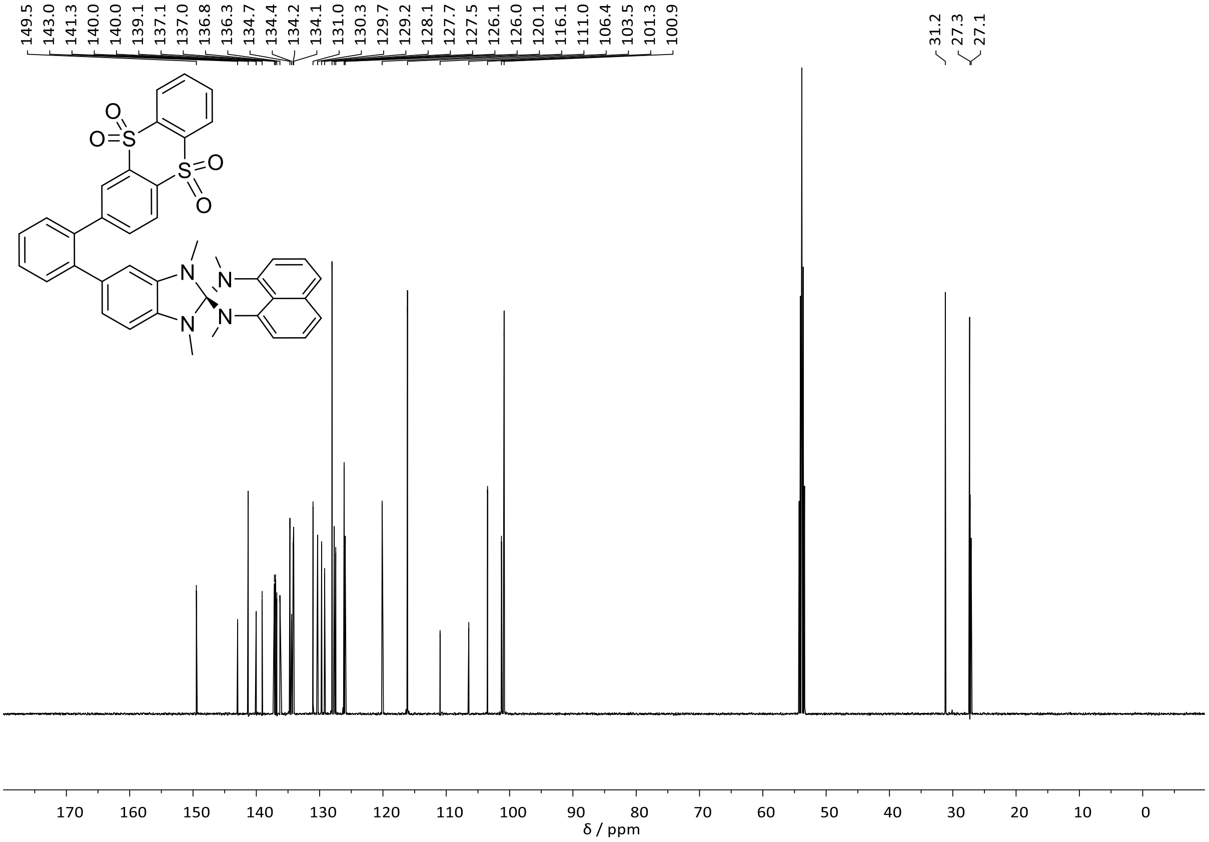

Figure S100: ^{13}C NMR spectrum (126 MHz) of **1-ms-TTO** in dichloromethane-*d*$_2$, contains traces of cyclohexane.

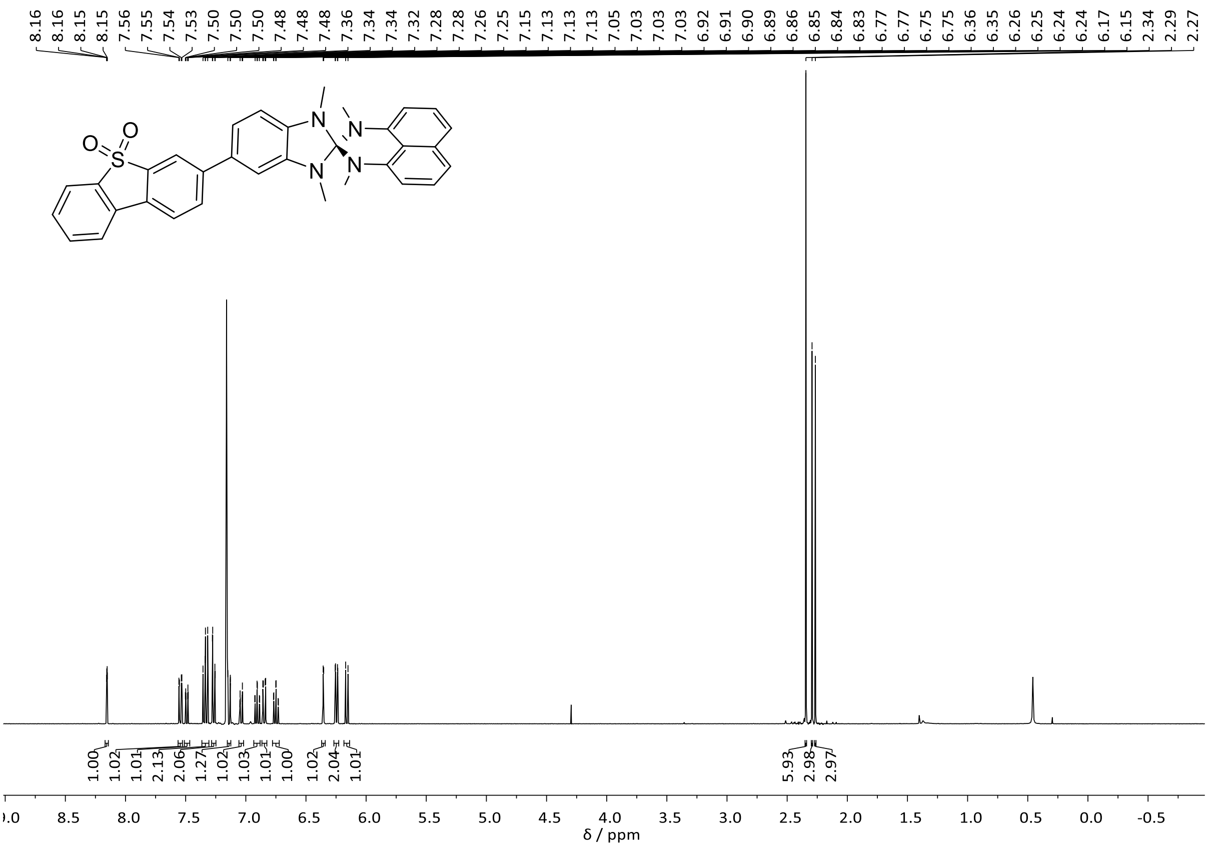

Figure S101: ^{1}H NMR spectrum (400 MHz, 303 K) of **1-m-DBTO** in benzene-d_6, contains traces of dichloromethane, cyclohexane, H_2O and silicon grease.

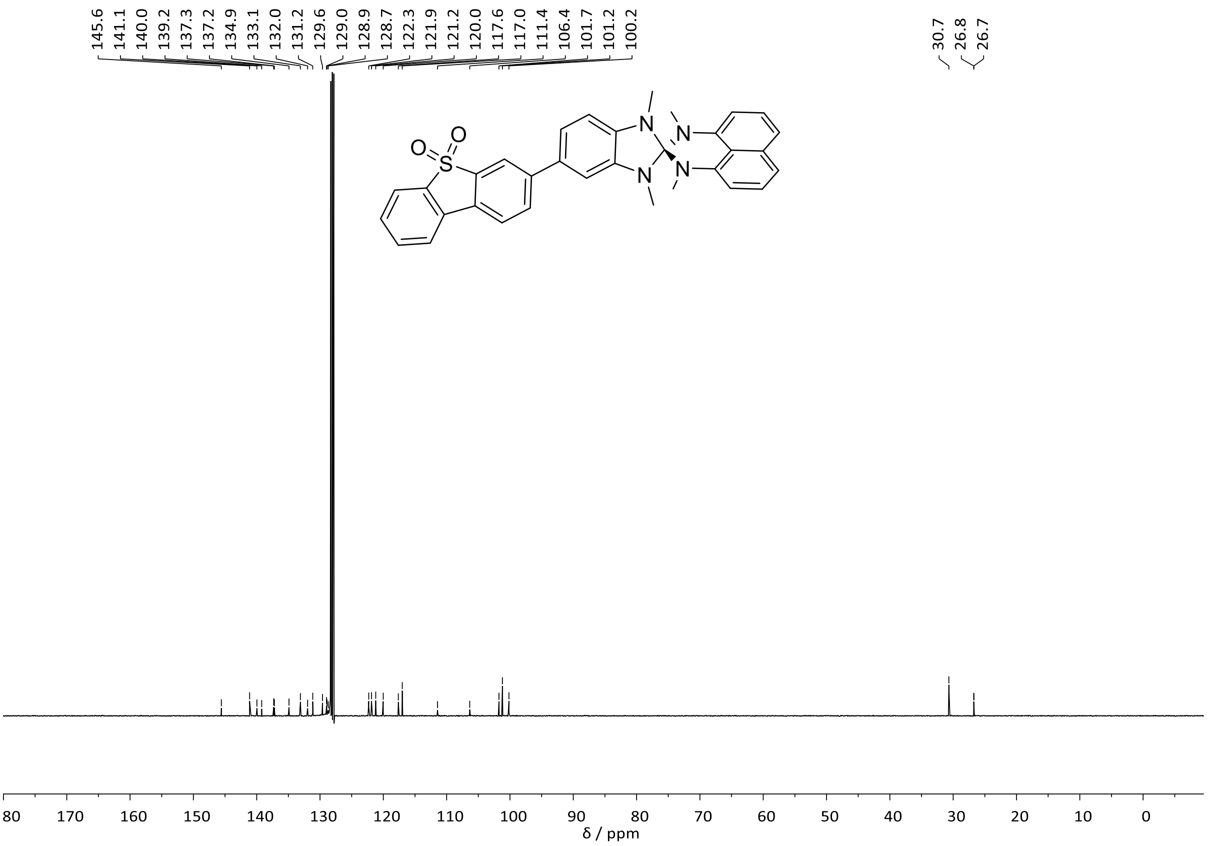

Figure S102: ^{13}C NMR spectrum (101 MHz, 303 K) of **1-m-DBTO** in benzene-d_6.

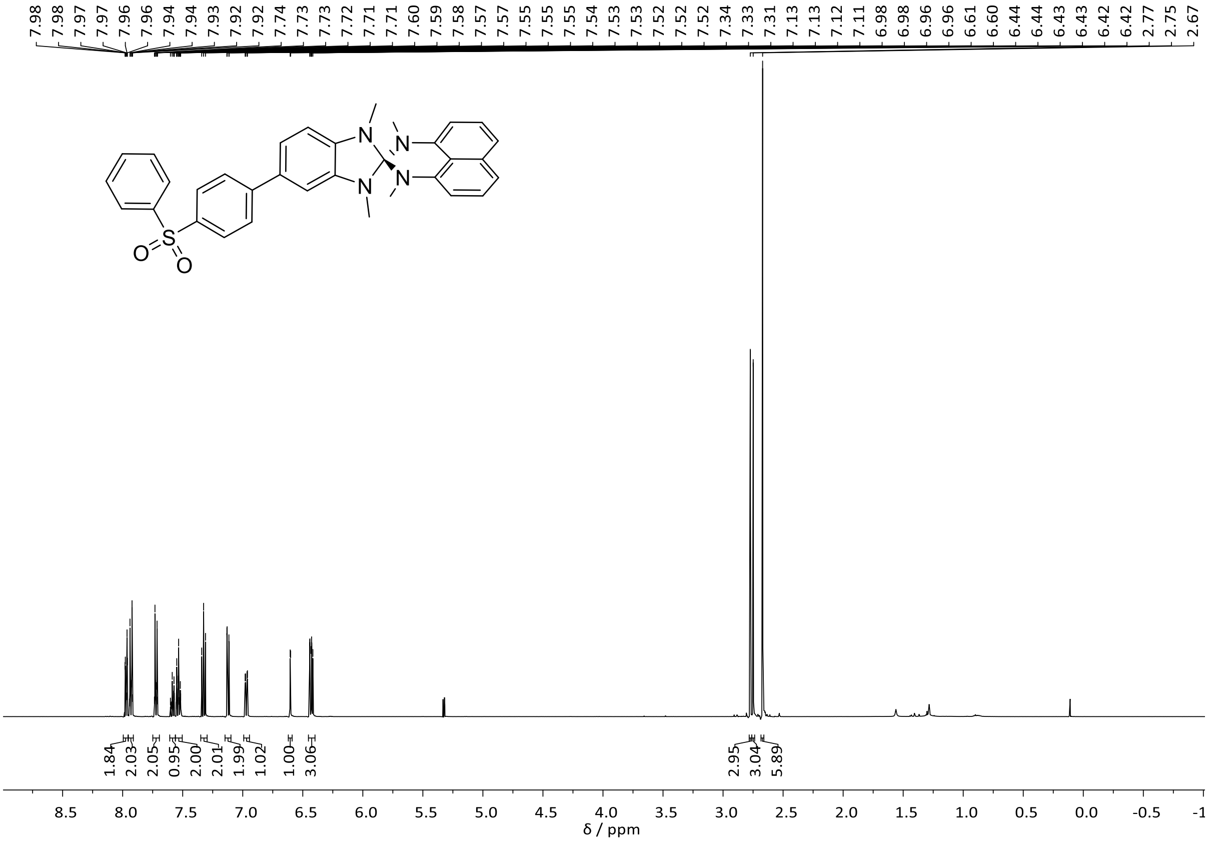

Figure S103: ^{1}H NMR spectrum (500 MHz) of **1-m-DPS** in dichloromethane-d_2, contains traces of H_2O, grease and silicon grease.

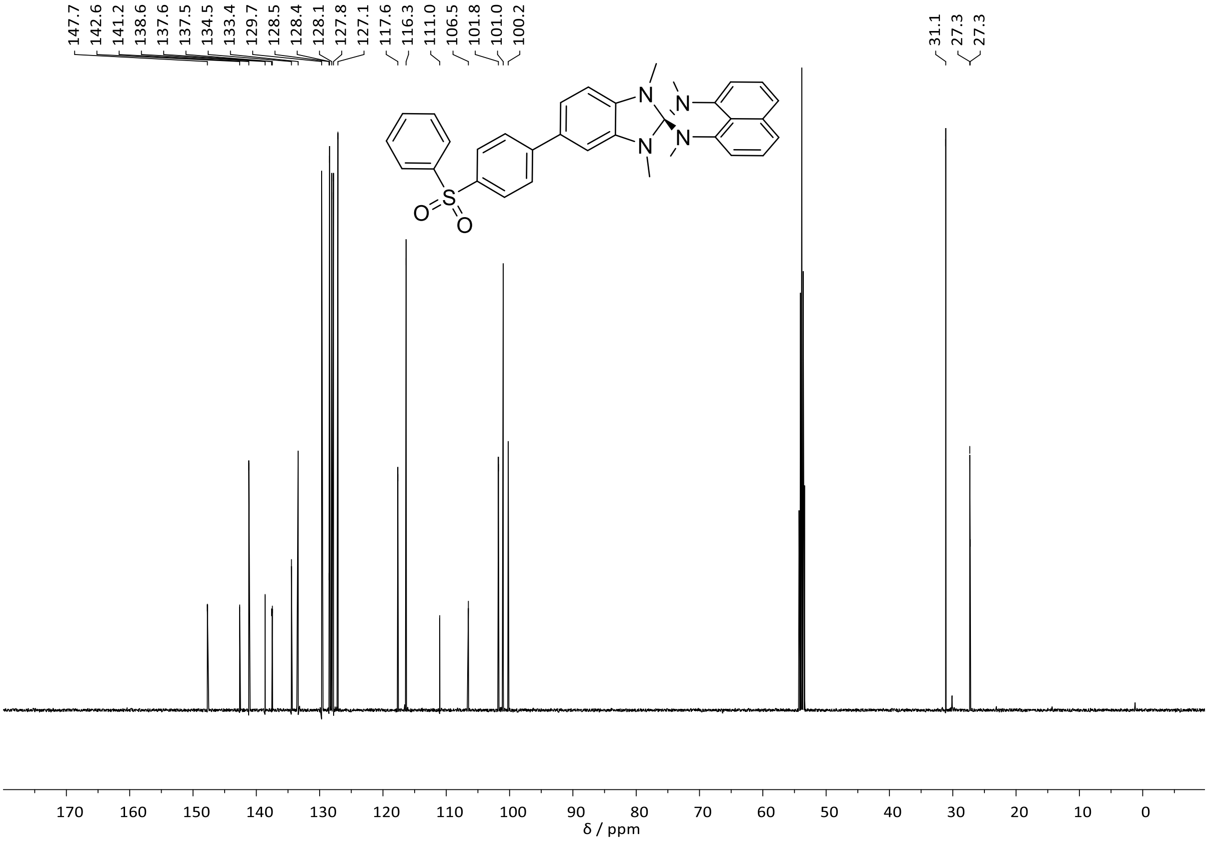

Figure S104: ^{13}C NMR spectrum (126 MHz) of **1-m-DPS** in dichloromethane-d_2, contains traces of grease and silicon grease.

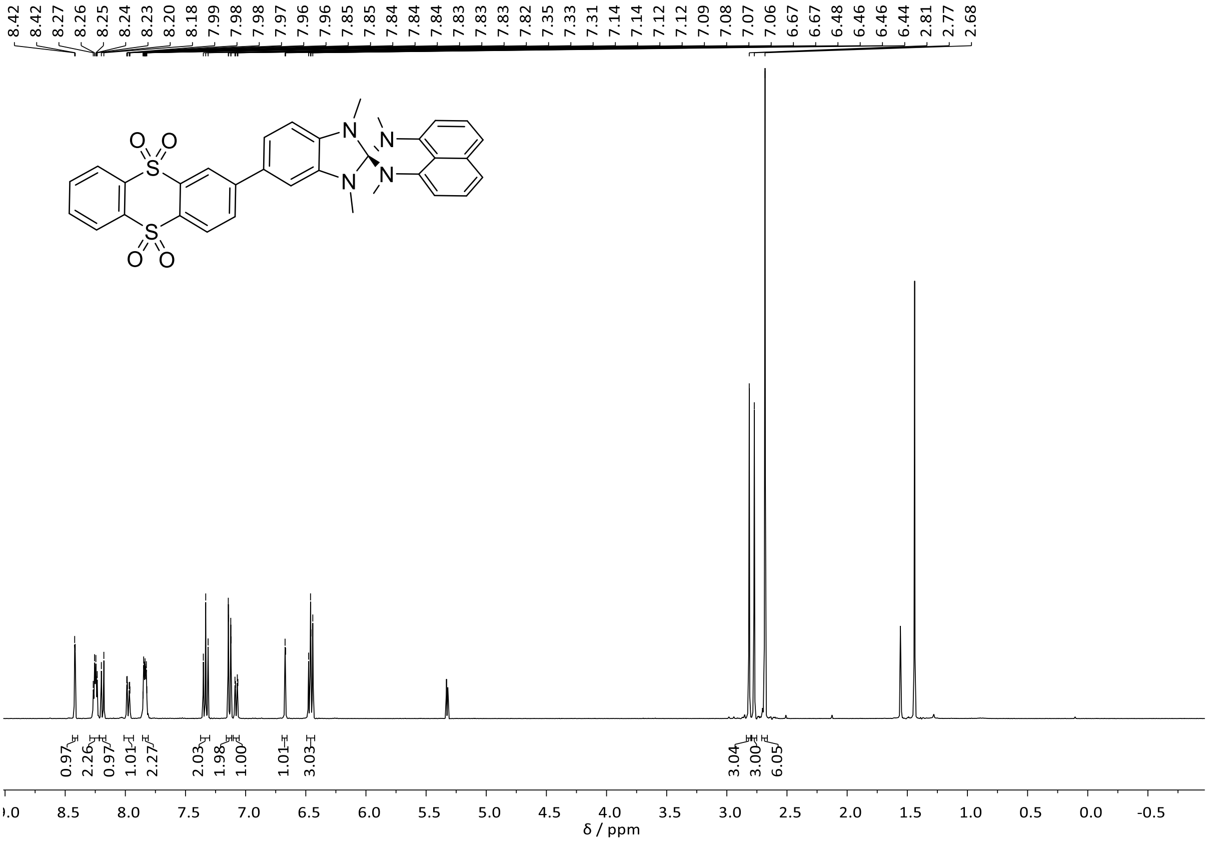

Figure S105: ^{1}H NMR spectrum (400 MHz, 303 K) of **1-m-TTO** in dichloromethane-d_2, contains traces of H_2O and cyclohexane.

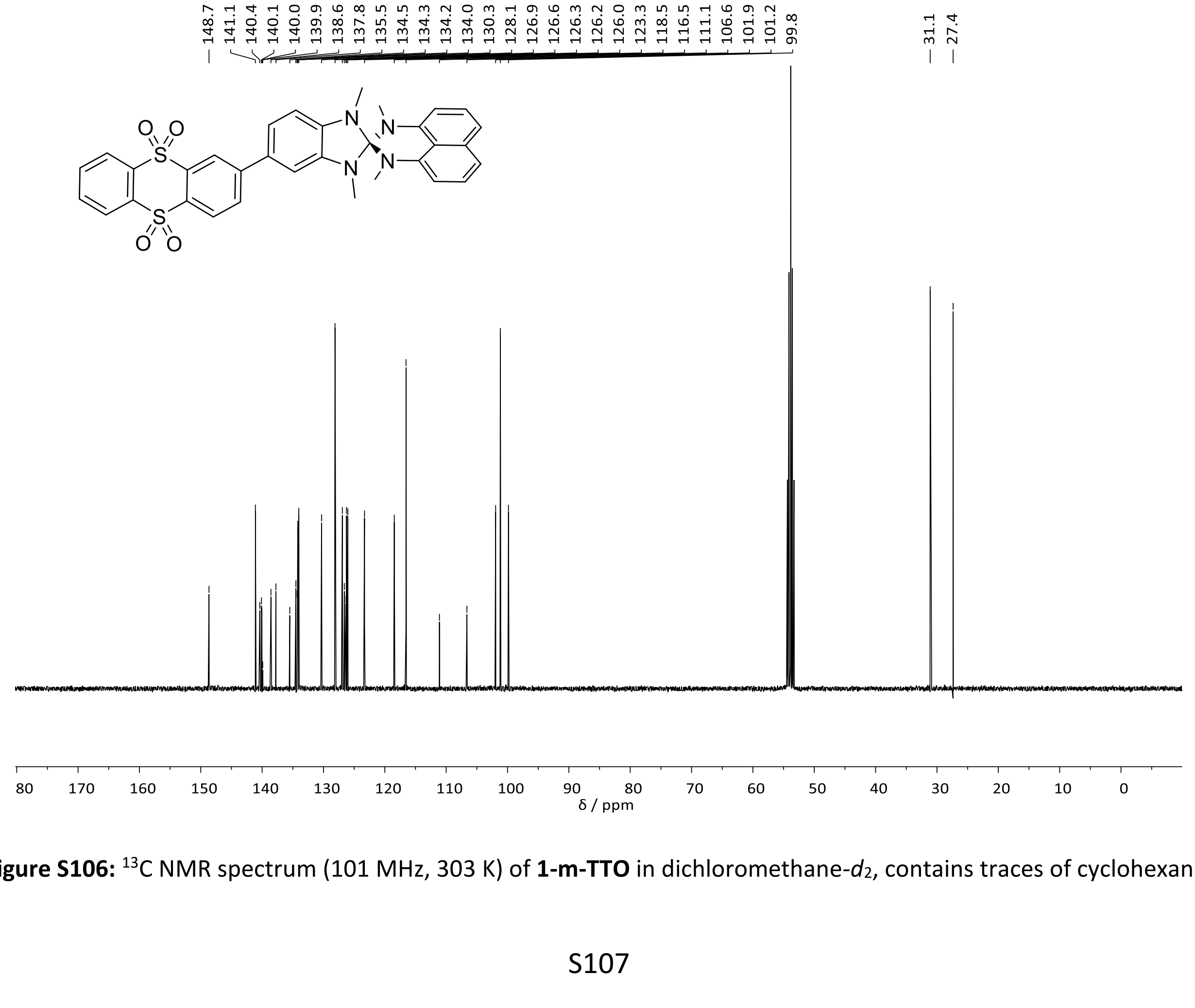

Figure S106: ^{13}C NMR spectrum (101 MHz, 303 K) of **1-m-TTO** in dichloromethane-d_2, contains traces of cyclohexane.

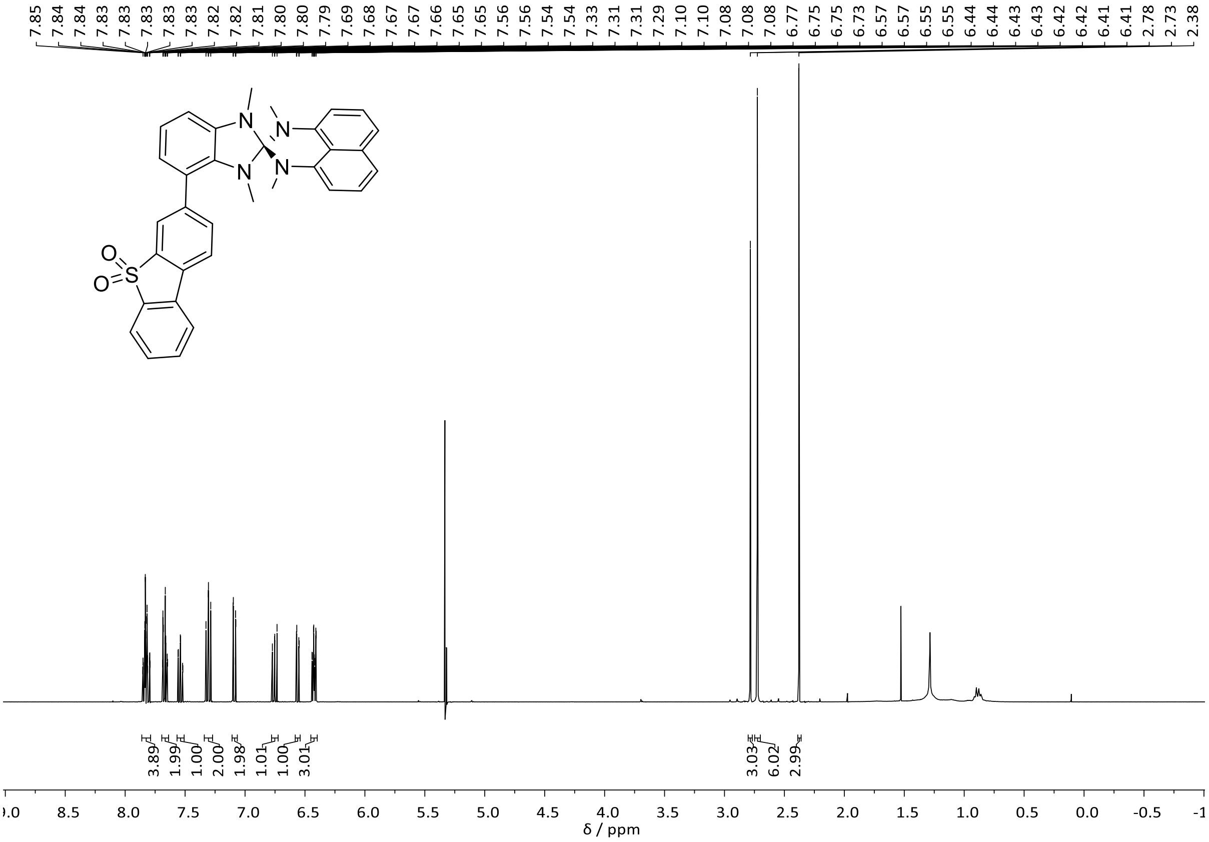

Figure S107: ^{1}H NMR spectrum (400 MHz, 303 K) of **1-o-DBTO** in dichloromethane-d_2, contains traces of H_2O and grease.

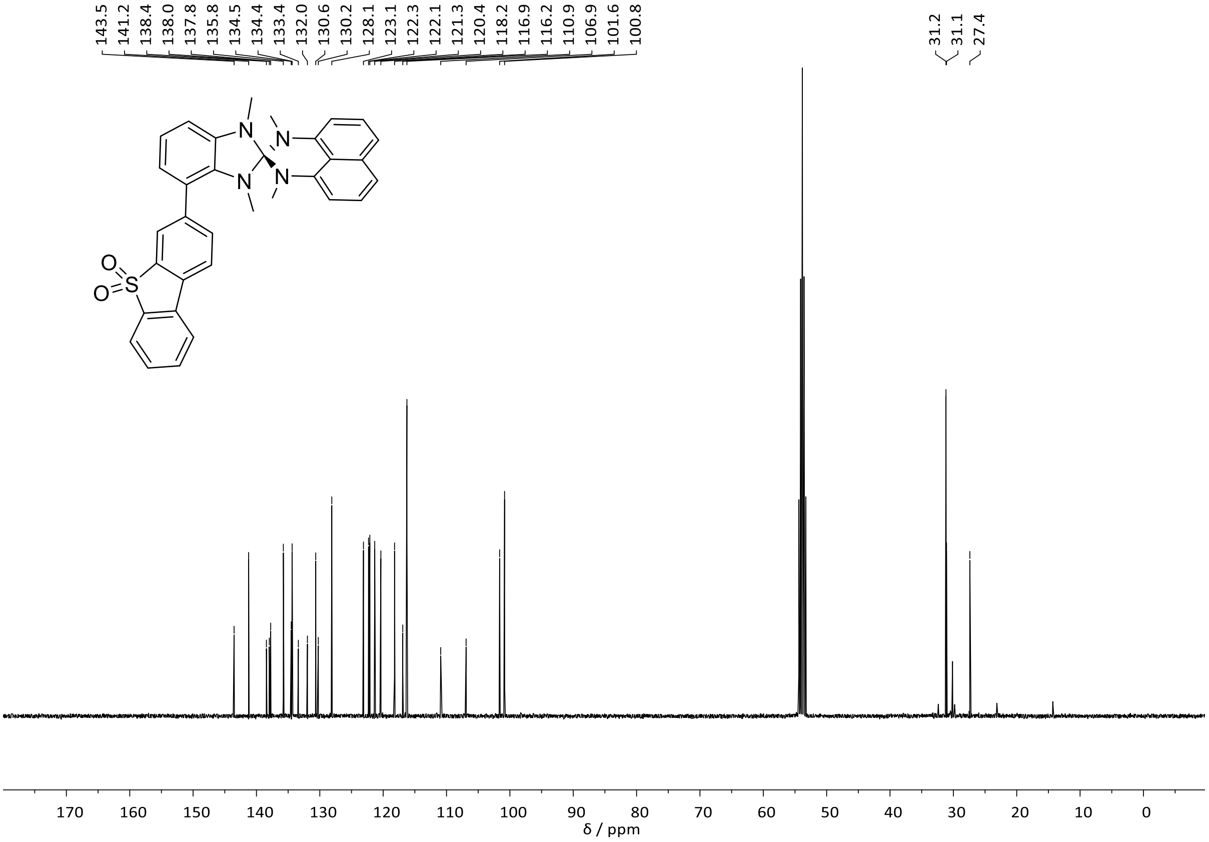

Figure S108: ^{13}C NMR spectrum (101 MHz, 303 K) of **1-o-DBTO** in dichloromethane-d_2, contains traces of grease.

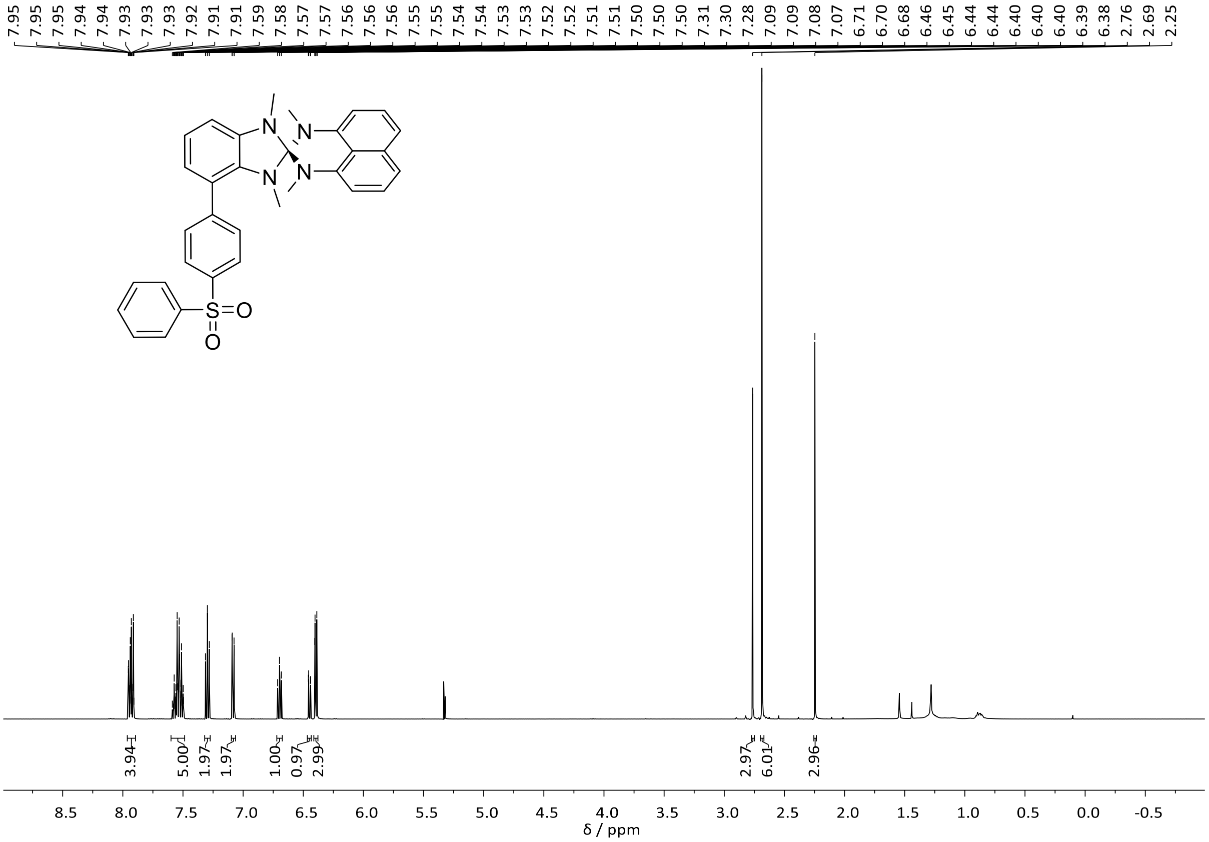

Figure S109: ^{1}H NMR spectrum (500 MHz) of **1-o-DPS** in dichloromethane-d_2, contains traces of H_2O, cyclohexane and grease.

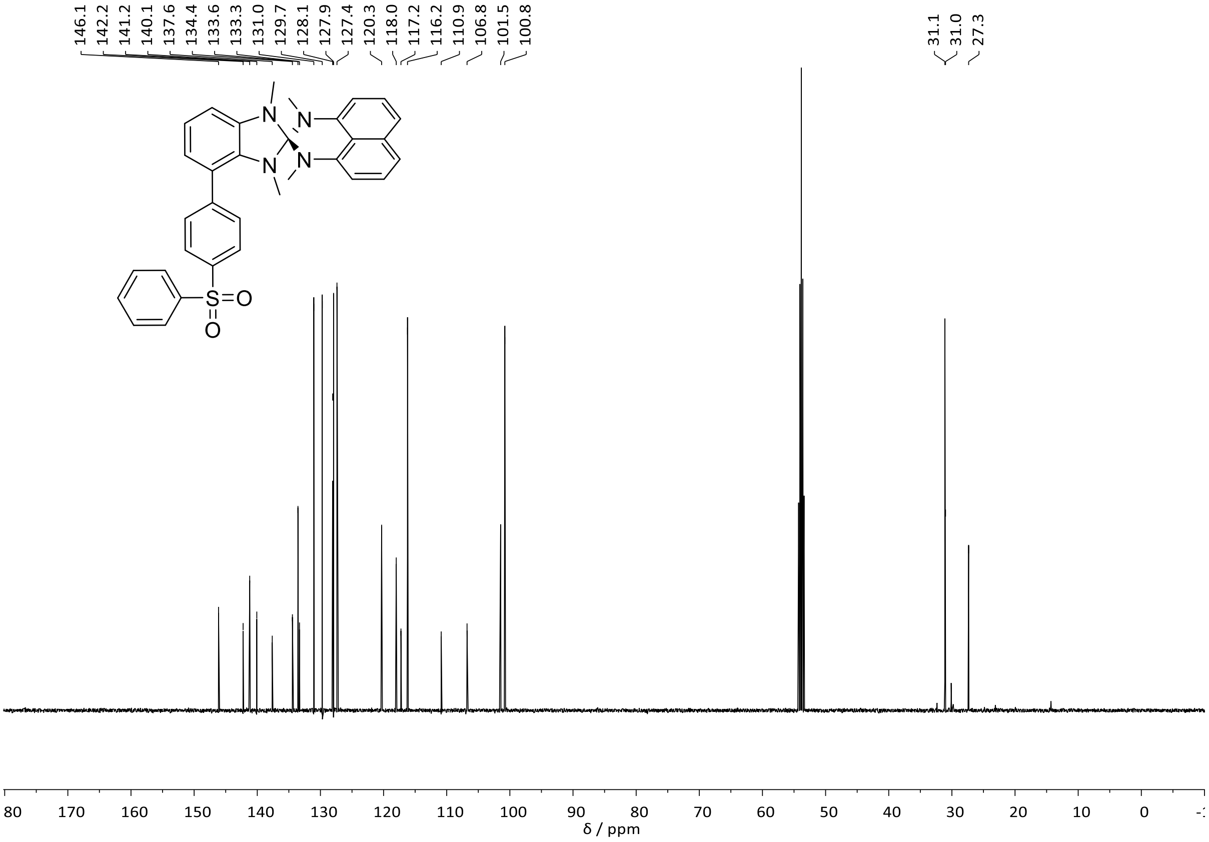

Figure S110: ^{13}C NMR spectrum (126 MHz) of **1-o-DPS** in dichloromethane-d_2, contains traces of cyclohexane and grease.

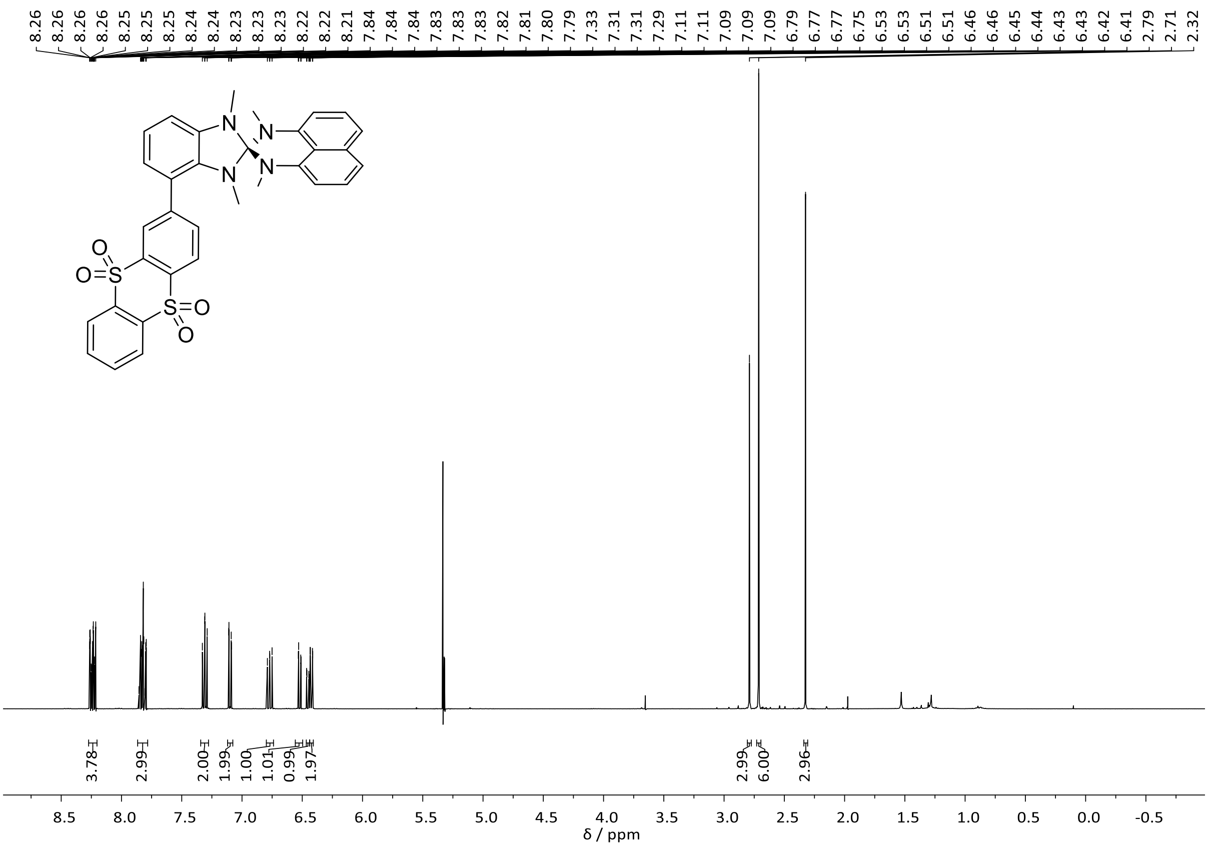

Figure S111: ^{1}H NMR spectrum (400 MHz, 303 K) of **1-o-TTO** in dichloromethane-d_2, contains traces of dioxane, H_2O and grease.

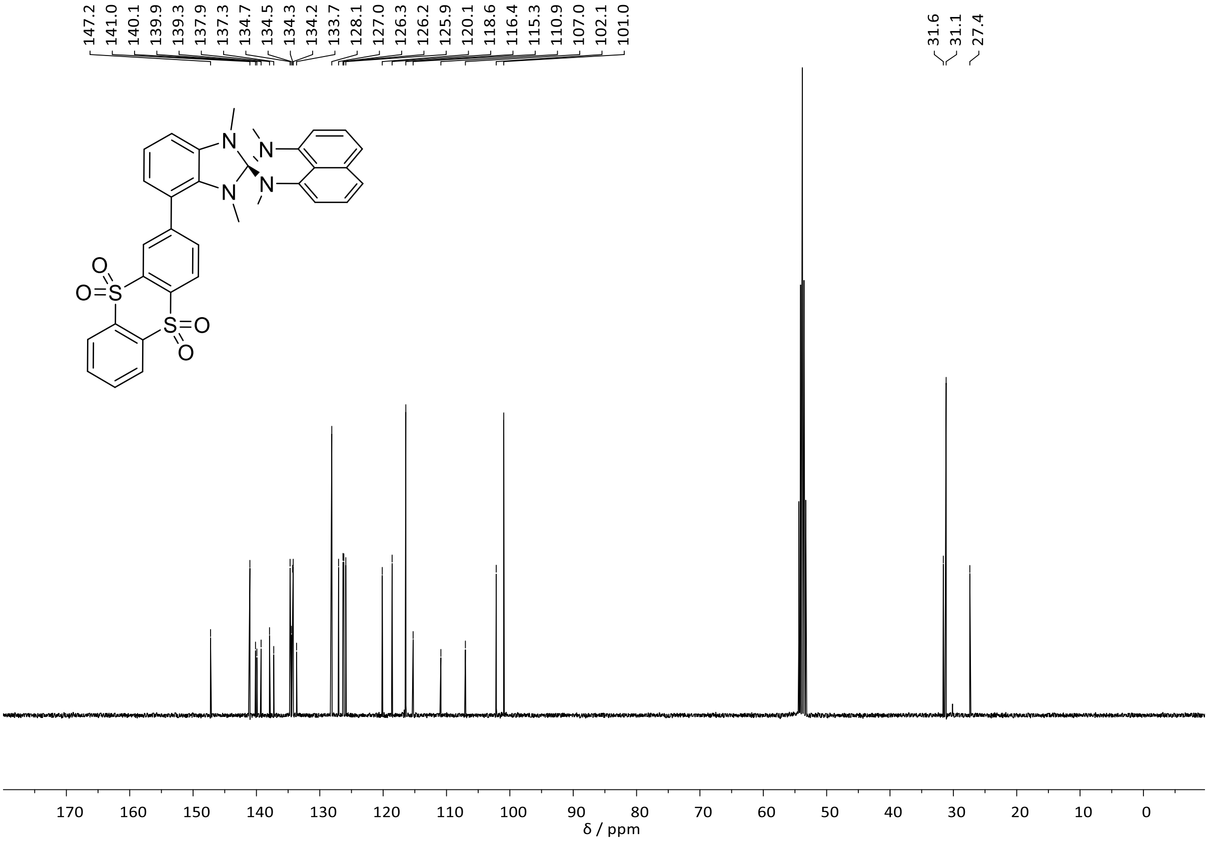

Figure S112: ^{13}C NMR spectrum (101 MHz, 303 K) of **1-o-TTO** in dichloromethane-d_2, contains traces of grease.

7. References

[1] D. B. G. Williams, M. Lawton, *J. Org. Chem.* **2010**, *75*, 8351–8354.

[2] H. E. Gottlieb, V. Kotlyar, A. Nudelman, *J. Org. Chem.* **1997**, *62*, 7512–7515.

[3] B. W. D'Andrade, S. Datta, S. R. Forrest, P. Djurovich, E. Polikarpov, M. E. Thompson, *Org. Electron.* **2005**, *6*, 11–20.

[4] H. Quast, E. Schmitt, *Chem. Ber.* **1968**, *101*, 1137–1139.

[5] R. Teodoro, M. Scheunemann, W. Deuther-Conrad, B. Wenzel, F. Fasoli, C. Gotti, M. Kranz, C. Donat, M. Patt, A. Hillmer, M.-Q. Zheng, D. Peters, J. Steinbach, O. Sabri, Y. Huang, P. Brust, *Molecules* **2015**, *20*, 18387–18421.

[6] *SAINT V8.37A, Bruker AXS*, Madison, Wisconsin, USA, **2015**.

[7] L. Krause, R. Herbst-Irmer, G. M. Sheldrick, D. Stalke, *J. Appl. Crystallogr.* **2015**, *48*, 3–10.

[8] G. M. Sheldrick, *Acta Crystallogr. Sect. A Found. Adv.* **2015**, *71*, 3–8.

[9] G. M. Sheldrick, *Acta Crystallogr. Sect. C Struct. Chem.* **2015**, *71*, 3–8.

[10] C. B. Hübschle, G. M. Sheldrick, B. Dittrich, *J. Appl. Crystallogr.* **2011**, *44*, 1281–1284.

[11] D. Kratzert, J. J. Holstein, I. Krossing, *J. Appl. Crystallogr.* **2015**, *48*, 933–938.

[12] D. Kratzert, I. Krossing, *J. Appl. Crystallogr.* **2018**, *51*, 928–934.

[13] C. R. Groom, I. J. Bruno, M. P. Lightfoot, S. C. Ward, *Acta Crystallogr. Sect. B Struct. Sci. Cryst. Eng. Mater.* **2016**, *72*, 171–179.

[14] D. Kratzert, "FinalCif," **2020**.

[15] *TURBOMOLE V7.3 2018, a Development of University of Karlsruhe and GmbH, Forschungszentrum Karlsruhe*, TURBOMOLE GmbH, Since 2007, **2018**.

[16] K. Eichkorn, O. Treutler, H. Öhm, M. Häser, R. Ahlrichs, *Chem. Phys. Lett.* **1995**, *242*, 283–289.

[17] R. Ahlrichs, K. May, *Phys. Chem. Chem. Phys.* **2000**, *2*, 943–945.

[18] H. Öhm, M. Häser, R. Ahlrichs, *Chem. Phys. Lett.* **1995**, *242*, 652.

[19] S. Grimme, J. Antony, S. Ehrlich, H. Krieg, *J. Chem. Phys.* **2010**, *132*, 154104.

[20] B. R. Brooks, C. L. Brooks, A. D. Mackerell, L. Nilsson, R. J. Petrella, B. Roux, Y. Won, G. Archontis, C. Bartels, S. Boresch, A. Caflisch, L. Caves, Q. Cui, A. R. Dinner, M. Feig, S. Fischer, J. Gao, … M. Karplus, *J. Comput. Chem.* **2009**, *30*, 1545–1614.

[21] A. D. Becke, E. R. Johnson, *J. Chem. Phys.* **2005**, *123*, 154101.

[22] E. R. Johnson, A. D. Becke, *J. Chem. Phys.* **2005**, *123*, 024101.

[23] E. R. Johnson, A. D. Becke, *J. Chem. Phys.* **2006**, *124*, 174104.

[24] S. Grimme, J. G. Brandenburg, C. Bannwarth, A. Hansen, *J. Chem. Phys.* **2015**, *054107*, 1–213.

[25] P. J. Stephens, F. J. Devlin, C. F. Chabalowski, M. J. Frisch, *J. Phys. Chem.* **1994**, *98*, 11623–11627.

[26] A. D. Becke, *J. Chem. Phys.* **1993**, *98*, 1372–1377.

[27] O. T. and K. Eichkorn, F. Weigend, R. Ahlrichs, *Theor. Chem. Acc.* **1997**, *97*, 119.
